# A Pull‐Out Mooring Wave Energy Converter: Design, Analysis, and Application

**DOI:** 10.1002/advs.202516945

**Published:** 2025-11-03

**Authors:** Weihan Xu, Junru Chen, Shanghao Gu, Xingya Feng, Fei Wang

**Affiliations:** ^1^ School of Microelectronics Southern University of Science and Technology Shenzhen 518055 China; ^2^ State Key Laboratory of Quantum Functional Materials Southern University of Science and Technology Shenzhen 518055 China; ^3^ Department of Ocean Science and Engineering Southern University of Science and Technology Shenzhen 518055 China

**Keywords:** electromagnetic transducers, hydrodynamic response, power take‐off systems, self‐powered sensing systems, wave energy converters

## Abstract

Wave energy converters (WEC), as alternative power sources for marine equipment, play a crucial role in promoting the development and utilization of ocean resources. However, the harsh marine environment and the low‐frequency nature of ocean waves pose substantial challenges to the lifespan and energy conversion efficiency of WECs. This paper proposes a pull‐out mooring wave energy converter (POM‐WEC) integrating a high‐performance electromagnetic power take‐off (EPTO) system. The EPTO system can convert the low‐frequency and low‐speed wave excitation into high‐speed inertial rotational motion of the rotor. Under an excitation velocity of 0.5 m s^−1^, the EPTO system achieves an average output power of 9.1 mW. A comprehensive methodology based on response amplitude operators and Cummins equations is developed to analyze and predict the motion response of the POM‐WEC under various wave conditions. By comparing the numerical simulation with experimental data, the validity and applicability of the methodology are further verified. The influences of wave height and frequency on both the motion response of the POM‐WEC and the output performance of the EPTO system are also systematically tested and evaluated. Furthermore, a self‐powered wireless sensing node based on the POM‐WEC is successfully developed, featuring non‐volatile data storage and three distinct operation modes.

## Graphical abstract

A pull‐out mooring wave energy converter (POM‐WEC) with a high‐performance electromagnetic power take‐off system efficiently converts low‐frequency wave excitation into high‐speed rotor motion, enabling reliable energy harvesting in real marine environments. A self‐powered wireless sensing node based on the POM‐WEC features three distinct operation modes, offering new opportunities for sustainable marine equipment and oceanographic research applications.

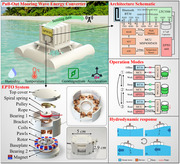

## 1 Introduction

The ocean plays an increasingly critical role in energy resources, climate regulation, biodiversity, and global economy with the development and utilization of ocean resources.^[^ ^1^, ^2^, ^3^ ^]^ Marine equipment is indispensable in the oceanographic research and development, such as monitoring buoys, remote operated vehicles, offshore platforms, and subsea pipelines.^[^ ^4^, ^5^, ^6^, ^7^ ^]^ The power supply for marine equipment mainly relies on batteries, fuel, or cables, which compromises their sustainability and poses potential risks of marine pollution.^[^ ^8^, ^9^, ^10^ ^]^ Therefore, an increasing number of renewable energy converters are used in marine equipment as additional power sources, significantly enhancing the sustainability of marine equipment.^[^ ^11^ ^]^ Compared with other renewable energy sources, such as solar and wind energy, ocean wave energy offers distinct advantages, including high energy density, low intermittency and high predictability.^[^ ^12^, ^13^ ^]^

Wave energy converters (WEC) are commonly used to convert ocean wave energy into electricity. The power take‐off (PTO) system is the core of a WEC, which determines the energy transmission method and conversion mechanism.^[^ ^14^ ^]^ Currently, much of the research on WECs focuses on large‐scale ocean wave energy harvesting as an alternative to fossil fuels.^[^ ^15^ ^]^ The PTO systems of these WECs are typically large and bulky in structure, with output power ranging from kW to MW. However, for WECs used in small‐scale marine equipment, compact, integrated, and high‐performance PTO systems are required to power electronic devices. In PTO systems, wave energy can be transmitted through various methods, such as mechanical transmission, pneumatic compression, or water flow, with mechanical transmission being the most common.^[^ ^16^, ^17^, ^18^, ^19^, ^20^, ^21^, ^22^, ^23^, ^24^, ^25^ ^]^ For PTO systems using mechanical transmission, linear‐to‐rotary drives typically exhibit higher conversion efficiency than direct linear drives owing to the low‐frequency nature of ocean waves.^[^ ^26^, ^27^ ^]^ The energy conversion mechanisms employed by PTO systems are primarily electromagnetic, electrostatic, piezoelectric, and triboelectric.^[^ ^28^, ^29^, ^30^, ^31^, ^32^, ^33^, ^34^, ^35^, ^36^, ^37^, ^38^, ^39^, ^40^ ^]^ Electrostatic, piezoelectric, and triboelectric PTO systems generally rely on dielectric material deformation, charge transfer, and contact electrification effects, making their output performance susceptible to environmental conditions and material aging.^[^ ^41^, ^42^, ^43^, ^44^ ^]^ Due to the structural maturity, high stability, and superior conversion efficiency, electromagnetic PTO systems are widely employed in WECs.^[^ ^45^ ^]^ Built‐in wave energy converters (BI‐WEC), whose PTO systems are completely isolated from seawater, have attracted increasing attention in recent years. Compared with other WECs, BI‐WECs typically have a longer lifespan and can be more easily integrated into marine equipment.^[^ ^46^, ^47^ ^]^ However, since the PTO system in BI‐WECs does not directly interact with ocean waves, wave energy can only be indirectly transmitted to the PTO system through the floater, posing challenges in terms of conversion efficiency and adaptability.^[^ ^48^, ^49^ ^]^ Mooring wave energy converters (MWEC) offer a reasonable trade‐off among lifespan, system integration, and conversion efficiency.^[^ ^50^ ^]^ The main components of the PTO system in MWECs are not directly exposed to seawater, but wave energy can still be directly transmitted to the PTO system via mooring ropes, chains, or rods. Therefore, developing compact, integrated, and high‐performance electromagnetic PTO systems for MWECs could provide a potential solution for improving the sustainability of marine equipment, advancing human understanding and exploration of the ocean.

To characterize the performance of PTO systems, some studies utilize mechanically actuated platforms to simulate wave motion and excite WECs.^[^ ^28^, ^29^ ^]^ The motion amplitude and period of these platforms can be adjusted to replicate various sea conditions. However, mechanically actuated platforms often struggle to accurately replicate the motion response of WECs in real ocean wave environments, as this response is closely related to the intrinsic properties of WECs, such as their size, shape, and mass distribution.^[^ ^51^ ^]^ Alternatively, some studies employ wave tanks to generate physical waves for exciting WECs, offering a rapid and cost‐effective testing environment for prototype validation under realistic wave conditions.^[^ ^52^, ^53^, ^54^ ^]^ However, the limited dimensions of wave tanks constrain the achievable wave height, and frequent wave reflections from the tank walls impede precise control of the wave profile. The actual motion response of WECs under different wave conditions is not only crucial for ensuring structural integrity and survivability but also plays a critical role in the design and optimization of PTO systems. By analyzing the motion response of WECs, researchers can gain insights into how wave characteristics influence the operation of PTO systems. Therefore, accurately predicting and measuring the motion response of WECs in ocean wave environments is crucial in marine engineering, as it supports mooring design, fatigue assessment, and the prediction of extreme responses during severe sea states. One of the primary prediction tools is the calculation of response amplitude operators (RAO), which represent the frequency‐dependent motion response of a floating structure to incident waves. These RAOs serve as the foundation for time‐domain simulations and performance evaluations under different wave conditions. Numerical simulations of RAOs are typically based on linear potential flow theory, which assumes an incompressible, irrotational, and inviscid fluid. Boundary element methods (BEM) have been widely employed to numerically solve the boundary‐value problems associated with potential flow theory. However, frequency‐domain methods fail to capture nonlinear effects introduced by components such as PTO systems. In the motion response of MWECs, PTO systems could introduce additional stiffness and damping effects, which may vary throughout motion process. To address these limitations, time‐domain modeling approaches such as the Cummins equation have been developed.^[^ ^55^ ^]^ The Cummins equation extends the linear potential flow theory by incorporating a convolution integral, enabling the inclusion of viscous damping, nonlinear forces, and external interactions.^[^ ^56^ ^]^

At present, the function of self‐powered beacons has been extensively demonstrated in numerous studies by using WECs to power lights.^[^ ^57^, ^58^ ^]^ Furthermore, some self‐powered sensing systems based on WECs have also been developed to enable real‐time monitoring of the ocean environment.^[^ ^59^, ^60^ ^]^ However, most self‐powered systems based on WECs still face challenges in adapting to the variability of the ocean environment and fulfilling the requirements for unmanned operation of marine equipment, such as switching between offline and online modes, non‐volatile data storage, and wireless data transmission. For marine equipment utilizing WECs as power sources, there remains substantial potential for further advancement.

To address these issues, we proposed a pull‐out mooring wave energy converter (POM‐WEC) integrating a high‐performance electromagnetic power take‐off (EPTO) system. The EPTO system can convert the low‐frequency and low‐speed wave excitation into high‐speed inertial rotational motion of the rotor. Through an electromagnetic transducer, the kinetic energy of the rotor is then converted into electrical power. The output performance of the EPTO system was characterized through an excitation‐response testing system and various influencing factors were analyzed, including the magnet‐to‐coil distance, excitation velocity, amplitude, and frequency. A comprehensive methodology was developed to analyze and predict the motion response of the POM‐WEC under different wave conditions. Starting from the linear frequency‐domain approach for calculating RAOs, the analysis was further extended into the time‐domain using the Cummins equation, thereby incorporating the influence of the EPTO system. A visual marker tracking technique was employed to capture the actual motion response of the POM‐WEC under various wave conditions. By comparing the numerical simulation with experimental data, the validity and applicability of the proposed methodology were fully verified. The effects of wave height and frequency on both the motion response of the POM‐WEC and the output performance of the EPTO system were also systematically tested and evaluated. Moreover, a self‐powered wireless sensing node (SPWSN) based on the POM‐WEC was successfully developed, featuring non‐volatile data storage and three distinct operation modes. This SPWSN can dynamically switch between modes to accommodate different application scenarios.

## 2 Results and Discussion

### 2.1 Design of the POM‐WEC

**Figure 1:**
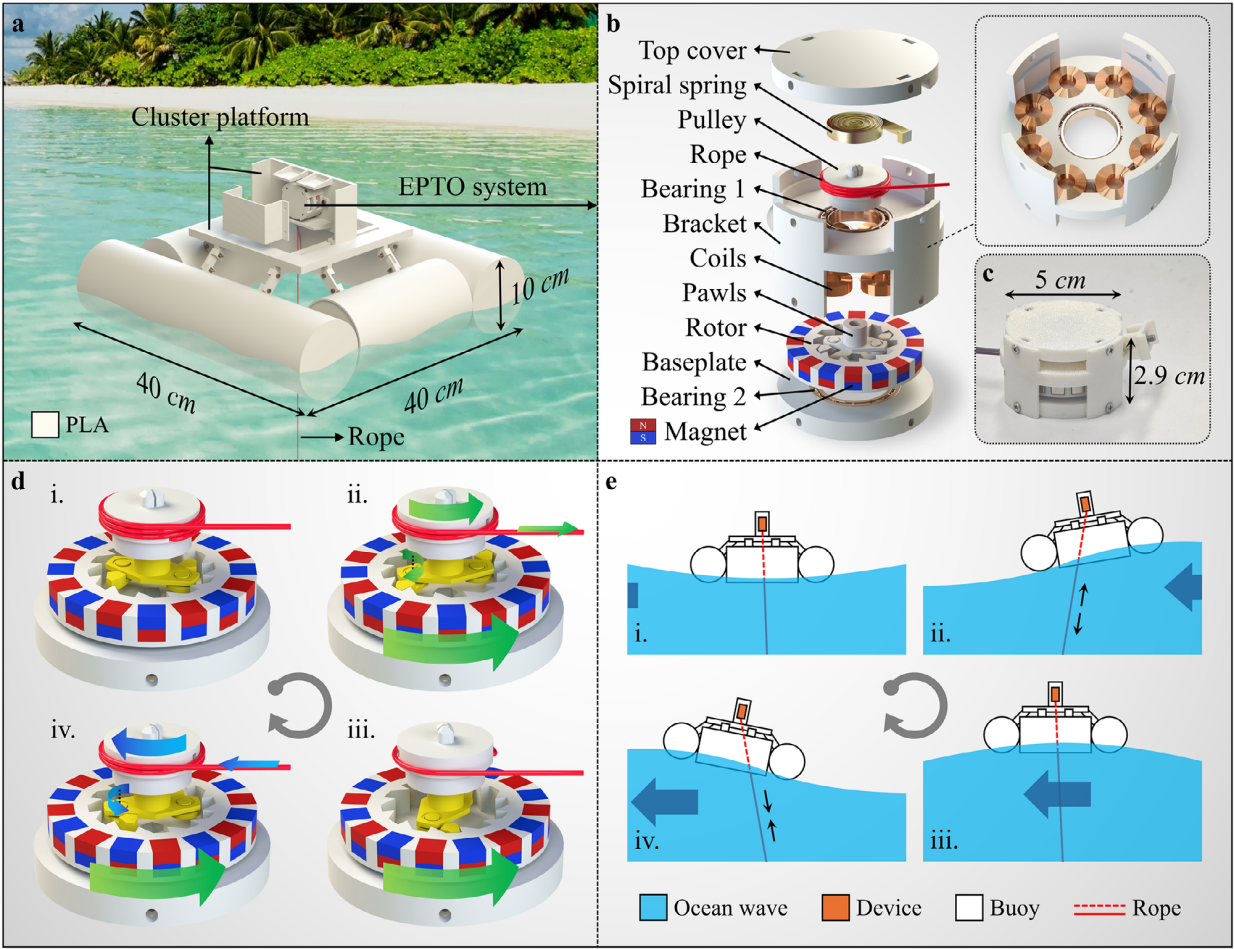
Structural overview and operating principle of the POM‐WEC. a) The structure and the nearshore application scenario of the POM‐WEC. b) The schematic view of the EPTO system. c) The physical photograph of the EPTO system. d) The operating principle of the EPTO system. e) The motion schematic of the POM‐WEC under wave excitation.

**Figure** **1**a illustrates the structure and a typical nearshore application scenario of the proposed POM‐WEC. Figure S1 (Supporting Information) shows its physical photograph. The POM‐WEC derives its primary buoyancy from four hollow cylindrical buoys and can be equipped with one or more EPTO systems. The wave kinetic energy is transmitted to the EPTO system via a flexible tension rope, which also constrains the planar drift of the POM‐WEC. The EPTO system can convert kinetic energy into electrical energy to drive low‐power sensing systems. Figure 1b,c presents the schematic view and the physical photograph of the EPTO system, respectively. The EPTO system primarily comprises three components, which are a rope‐driven actuator, a pawl‐ratchet clutch, and an electromagnetic transducer, respectively. The rope‐driven actuator includes a spiral spring, a pulley and a flexible tension rope. The pawl‐ratchet clutch features a dual‐pawl mechanism and a rotor with a ratchet structure. The electromagnetic transducer consists of 16 cubic magnets arranged in an alternating polarity configuration and 8 coils embedded within the bracket. A simulation using COMSOL Multiphysics is conducted to analyze the magnetic flux density distribution for alternate polarity (AP) arrangement and the same polarity (SP) arrangement. To balance computational efficiency with model accuracy, we simplified the 3D annular arrangement into a 2D linear model. Figure S2 (Supporting Information) shows the distribution of the magnetic flux density component normal to the coil plane. For a 1 mm air gaps, the AP arrangement achieves a peak‐to‐peak flux density change of approximately 0.88 T with an average change rate of 73 mT mm^−1^. This is significantly higher than the 0.16 T amplitude and 27 mT mm^−1^ change rate of the SP arrangement. The corresponding plot of magnetic flux density versus displacement quantitatively confirms the superiority of the AP arrangement. Figure S3 (Supporting Information) shows the flux density variation under different air gaps for the AP arrangement. Both the amplitude and the change rate of flux density decrease significantly as the air gap increases. The air gap size of our EPTO system is set to 1 mm to achieve higher output performance. Figure 1d shows the operating principle of the EPTO system. When the rope is stretched, the pulley rotates accordingly, and the spiral spring is twisted until the stretching stops. When the rope is released, the spiral spring drives the pulley to rotate in the opposite direction until returning to initial state. Rotation of the pulley in the forward direction causes the pawls to engage with the ratchets, thereby driving the rotor to rotate. Conversely, reverse rotation leads to disengagement of the pawls, allowing the rotor to continue rotating due to inertia. Wave excitation is thus converted into high‐speed, unidirectional, and inertial rotational motion of the rotor through the combined action of the rope‐driven actuator and the pawl‐ratchet clutch. Subsequently, the kinetic energy of the rotor is transformed into electrical energy via the electromagnetic transducer. Figure 1e presents the motion schematic of the POM‐WEC under wave excitation. The rope is periodically stretched and released as it moves with the POM‐WEC, enabling continuous power generation by the EPTO system. Relevant parameters of the POM‐WEC and the EPTO system are provided in Table S1 (Supporting Information).

### 2.2 High‐Performance EPTO System

**Figure 2:**
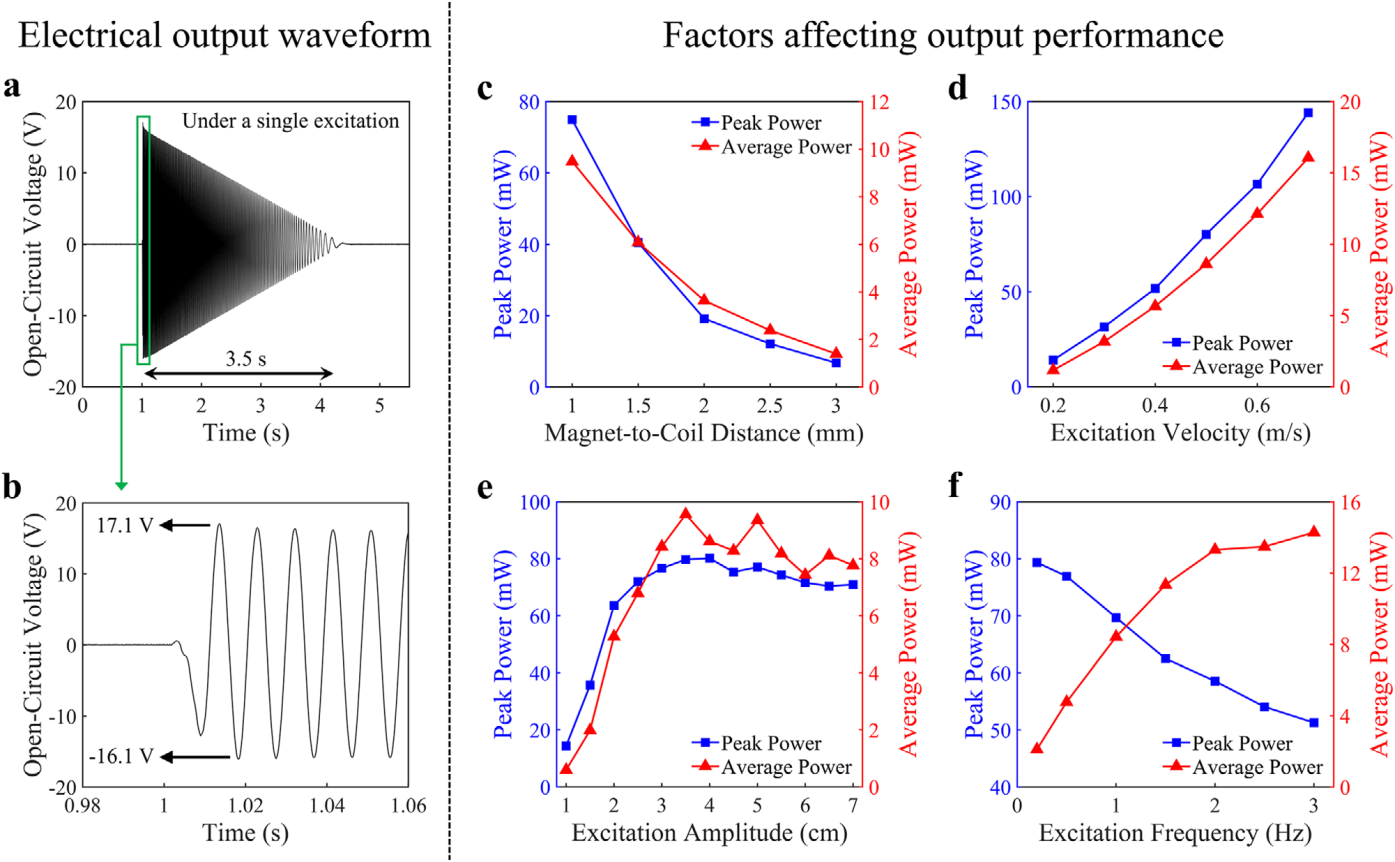
Output performance and affecting factors of the EPTO system. a) The open‐circuit voltage waveform of the EPTO system under a single excitation with 0.5 m s^−1^ velocity and 4 cm amplitude and b) its partial enlarged view. c) The effect of the magnet‐to‐coil distance, d) the effect of the excitation velocity, e) the effect of the excitation amplitude, and f) the effect of the excitation frequency when the magnet‐to‐coil distance is 1 mm, the excitation velocity is 0.5 m s^−1^, the excitation amplitude is 4 cm, and the excitation frequency is 1 Hz.

The output performance of the EPTO system is characterized through a series of tests. The following experimental conditions are specified as default in this section: the load resistance is 2.5 kΩ, the magnet‐to‐coil distance is 1 mm, the excitation velocity is 0.5 m s^−1^, the excitation amplitude is 4 cm, and the excitation frequency is 1 Hz. **Figure** **2**a,b displays the open‐circuit voltage waveform of the EPTO system under a single excitation. The EPTO system can continuously output electrical energy for approximately 3.5 s, with a peak‐to‐peak voltage of around 33.2 V. As the rotor speed decreases, both the amplitude and frequency of the output voltage diminish. Figure S4 (Supporting Information) illustrates output voltage waveforms of the EPTO system at different load resistances under a single excitation. Due to Lenz's law, the magnetic field generated by induced current always prevents the change in magnetic flux that generates the induced current, which retards the movement of magnets in the EPTO system. As the load resistance decreases, the induced current increases, which shortens the output duration of the EPTO system, as shown in Figure S5 (Supporting Information). The output duration directly affects the average power of the EPTO system. Consequently, the average and peak power of the EPTO system reach their optimal values at different load resistances. Figure S6 (Supporting Information) presents the matching resistance test results for the EPTO system, achieving a peak power of 191.2 mW at a load resistance of 0.18 kΩ and an average power of 9.1 mW at a load resistance of 2 kΩ. Figure S7 (Supporting Information) shows the output current for the EPTO system under different loads. For a constant load, the output current of the EPTO system is directly proportional to the output voltage, which itself is determined by the POM‐WEC hydrodynamic response. Since the hydrodynamic response is a function of the wave conditions (wave height and period), the output current is consequently affected by wave conditions in the same manner as the output power.

Figure 2c shows the effect of the magnet‐to‐coil distance on output performance. As the distance increases, the change in magnetic flux within the coils slows down, resulting in reduced output power. Figure 2d shows the effect of the excitation velocity on output performance. As the excitation velocity increases, the magnets rotate faster, which boosts up the rate of magnetic flux change, thereby improving the output power. Figure 2e shows the effect of the excitation amplitude on output performance. When the excitation amplitude is less than 3 cm, the rotor in the EPTO system cannot be effectively driven, and its rotational speed fails to match the excitation velocity. This occurs because a minimum excitation amplitude is required to ensure engagement between the pawls and ratchets prior to effective energy transfer to the rotor. At a constant excitation velocity, once the rotor can be effectively driven, further increases in excitation amplitude primarily extend the excitation process without significantly enhancing the rotor speed, leading to saturation in the output power. Figure 2f shows the effect of the excitation frequency on output performance. Figure S8 (Supporting Information) displays output voltage waveforms of the EPTO system under different frequency of excitation. At higher excitation frequencies, the rotor is still in rotation when the new excitation arrives, which means a larger minimum excitation amplitude is required to ensure engagement between the pawls and ratchets. The interaction mechanism between the dual‐pawl structure and the ratchets was analyzed in detail in our previous work.^[^ ^61^ ^]^ In short, the EPTO system requires a larger excitation amplitude at higher excitation frequencies to be effectively driven. Therefore, the peak power is reduced with the increase of excitation frequency. In contrast, the prolonged duration of high‐speed rotor rotation contributes to an increase in average power.

Furthermore, the long‐term durability of the PTO system is a critical factor for its practical application. To assess the impact of internal friction losses on long‐term performance and durability of our PTO system, a durability test have been conducted. A brand‐new PTO system was continuously subjected to about 10000 excitation cycles with an excitation velocity of 0.5 m/s and an excitation amplitude of 7 cm. Figure S9 (Supporting Information) presents the voltage output waveform of the PTO system under a 2.5 kΩ load throughout the durability test. It can be observed that the output performance shows almost no degradation after about 10 000 excitation cycles. Figure S10 (Supporting Information) shows a morphological comparison of the tensile rope before and after the durability test. The rope's morphology remains intact, with no signs of breakage or aging due to wear, indicating excellent wear resistance. These durability test results demonstrate that the influence of internal friction losses on fatigue life and overall performance degradation of the PTO system is minimal under the tested conditions.

### 2.3 Hydrodynamic Response Prediction of the POM‐WEC

The motion response of the POM‐WEC under wave excitation can be analyzed using linear potential flow theory in the frequency domain. Assuming the fluid is inviscid, incompressible, and irrotational, and that the structure undergoes small‐amplitude oscillatory motions, the governing equation of motion in the frequency domain is given by:

(1) $$\left[-\left[M+M^{a}\left(\omega \right)\right]\omega^{2}-i\omega B\left(\omega \right)+\left[C+C_{\mathrm{add}}\right]\right]\xi \left(\omega \right)=F_{\mathrm{exc}}\left(\omega \right)$$

where *M* is the structural mass and inertia properties of the floating body, ω is the wave angular frequency, *M^a^* (ω) is the added mass matrix that accounts for the inertia of the fluid surrounding the structure, *B*(ω) is the radiation damping matrix, representing energy lost to radiated waves due to body oscillation, *C* is the hydrostatic restoring stiffness matrix, representing the hydrostatic restoring forces and moments acting on a floating body due to its displacement from equilibrium, *C_add_* is the moored stiffness matrix related to the EPTO system, ξ(ω) is the complex motion amplitude vector, and *F_exc_* (ω) is the complex wave excitation force vector, incorporating both Froude–Krylov and diffraction effects. The hydrodynamic coefficients, including the added mass, radiation damping, and excitation force, are computed using Nemoh, an open‐source BEM solver. The equivalent mooring stiffness is experimentally obtained by applying known displacements and measuring the corresponding restoring forces.

By incorporating the structural properties, hydrodynamic coefficients, and moored stiffness into the theoretical model, the motion response of the POM‐WEC can be described using the frequency‐domain motion equation. Solving this equation for each degree of freedom yields the RAOs, defined as the ratio of the response amplitude to the wave amplitude at each frequency. The RAOs characterize the frequency‐dependent dynamic behavior of the structure and are fundamental in estimating motions under different sea states via spectral analysis. After the RAOs are calculated in the frequency domain, they can be further used to reconstruct the time‐domain motion response. The motion response of the POM‐WEC in each degree of freedom is computed as:

(2) $$\xi^{\left(1\right)}\left(\omega \right)=\left|\mathrm{RAO}\left(\omega \right)\right|\cdot a\cdot \cos\left(\omega t+\varphi +\arg\left(\mathrm{RAO}\left(\omega \right)\right)\right)$$

where the magnitude of the RAO determines the motion amplitude, while the phase of the RAO introduces a phase shift between the wave and the motion response. The theoretical prediction from the RAO will be compared with the linear component of the experimental motion response, minimizing the influence of higher‐order wave effects and nonlinear interaction that cannot be captured by the linear model. Additional details regarding the theoretical prediction based on RAOs are provided in Text S1 (Supporting Information).

To account for nonlinear and transient effects beyond the limitations of the frequency‐domain linear potential flow model, the motion of the POM‐WEC is further analyzed using the Cummins equation, a time‐domain formulation derived from linear potential flow theory with memory effects. This equation is derived by applying the convolution theorem to the frequency‐domain representation of radiation forces and allows time‐domain simulation of hydrodynamic response under arbitrary wave inputs, including regular and irregular waves. The Cummins equation is expressed as:

(3) $$\begin{array}{ccc} & & \left(M+M_{\infty }^{a}\right)\ddot{\xi }\left(t\right)+\int_{0}^{t}K\left(t-\tau \right)\dot{\xi }\left(t\right)d\tau +B_{\mathrm{add}}\dot{\xi }\left(t\right)+C\xi \left(t\right)=\\ & & F_{\mathrm{wave}}^{\left(1\right)}\left(t\right)+F_{\mathrm{wave}}^{\left(2\right)}\left(t\right)+F_{\mathrm{EPTO}}\left(t\right) \end{array}$$

where $M_{\infty }^{a}$ is the infinite frequency added mass matrix, *K*(*t*) is the retardation function, τ represents a dummy time variable used in the convolution integral, allowing the retardation function *K*(*t* − τ) to model the memory effects of wave radiation forces resulting from the past motions, *B_add_* is the viscous damping coefficient, representing additional energy dissipation caused by viscous effects that cannot be captured by linear inviscid models, $F_{wave}^{\left(1\right)}\left(t\right)$ and $F_{wave}^{\left(2\right)}\left(t\right)$ are the time‐dependent first‐order and second‐order wave excitation force, respectively, *F_EPTO_* (*t*) is the force exerted by the EPTO system, and $\ddot{\xi }\left(t\right)$, $\dot{\xi }\left(t\right)$, and ξ(*t*) are the time‐dependent acceleration, velocity, and amplitude vectors of the POM‐WEC. Additional details regarding the theoretical prediction based on Cummins equation are provided in Text S2 (Supporting Information).

### 2.4 Hydrodynamic Response Measurement of the POM‐WEC

#### 2.4.1 POM‐WEC in Regular Wave

**Figure 3:**
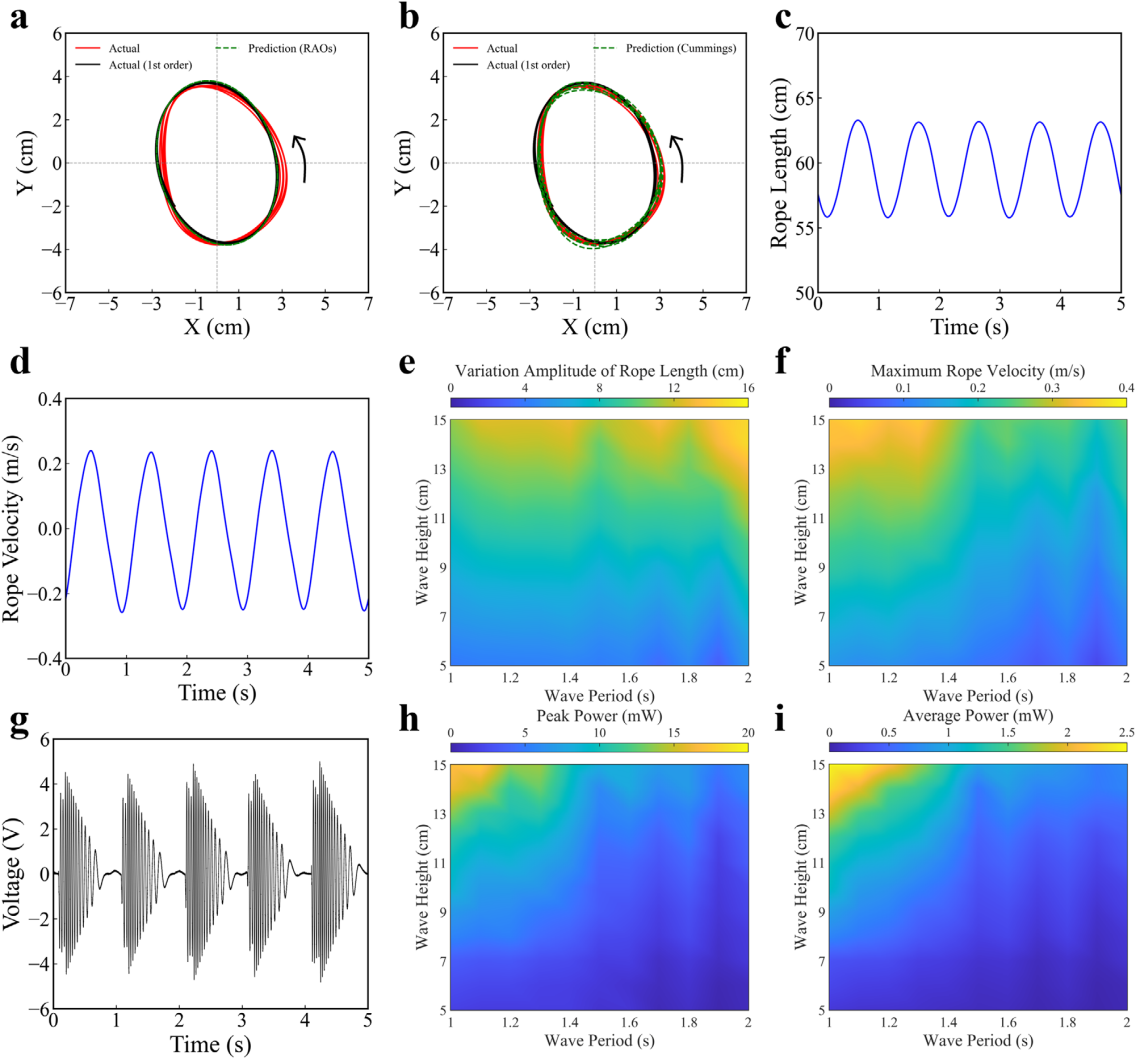
Motion and output performance of the POM‐WEC under regular waves with 1 s period and 10 cm height. a) The comparison between the theoretical prediction trajectory using RAOs and the actual motion trajectory. b) The comparison between the theoretical prediction trajectory using Cummins equation and the actual motion trajectory. c) The variations in rope length. d) The variations in velocity along the rope direction. e) The rope length amplitude of the POM‐WEC under regular waves with different heights and periods. f) The maximum rope pulling velocity of the POM‐WEC under regular waves with different heights and periods. g) The output voltage waveform of the EPTO system. h) The peak power of the POM‐WEC under regular waves with different heights and periods. i) The average power of the POM‐WEC under regular waves with different heights and periods.

In this study, unidirectional incident waves were used to excite the POM‐WEC. Figure S11 (Supporting Information) shows the profile of the regular wave with a period of 1 s and a height of 10 cm. Video S1 (Supporting Information) shows the motion response of the POM‐WEC under the regular wave. The motion trajectory of the POM‐WEC was tracked using a visual marker tracking method based on OpenCV within the surge‐heave plane. To further assess the accuracy of the theoretical models, we performed a detailed comparison between the experimental results and the predictions based on both the RAO method and the Cummins equation, as shown in  **Figure** 3a,b. The red line represents the actual motion trajectory of the POM‐WEC, while the black line corresponds to the dominant frequency component of the actual trajectory, extracted via Fourier transform. The green dashed line represents the theoretical prediction trajectory. Figure 3a compares the RAO‐based theoretical prediction with the actual motion trajectory of the POM‐WEC under the regular wave, demonstrating that the prediction align well with the dominant frequency component of the experimental data. On the other hand, Figure 3b shows the theoretical prediction based on Cummins equation, which can effectively capture the nonlinear behaviors of the POM‐WEC, including the EPTO response. As a linear model, the RAO method provides a first‐order approximation of the motion response and is effective within the linear regime. In comparison, the Cummins equation offers a more comprehensive representation, especially under conditions involving nonlinear interactions, such as those introduced by the EPTO system. Overall, the theoretical prediction based on the Cummins equation exhibits better agreement with the actual motion trajectory than that derived from the RAO method. These results indicate that, while the RAO method offers a quick and reasonable estimate in the linear regime, the Cummins equation provides a more accurate motion response prediction of the POM‐WEC under real wave conditions, effectively capturing both the linear and nonlinear effects that are crucial for optimizing WECs and PTO systems.

Performance characterization of the EPTO system reveals that the excitation velocity, amplitude, and frequency are the primary influencing factors. Specifically, the excitation velocity, amplitude, and frequency are determined by the rope pulling velocity, rope length amplitude, and wave period, respectively. Based on the motion trajectory, the variations in rope length and the velocity along the rope direction can be obtained, as shown in Figure 3c,d. Under the regular wave, the rope length amplitude can reach about 7.2 cm and the maximum rope pulling velocity can reach about 0.24 m s^−1^. Moreover, Figure S12 (Supporting Information) shows the variations in the displacement and velocity of the POM‐WEC in both the surge and heave directions. It can be observed that the rope length amplitude and rope pulling velocity are more determined by the displacement and velocity of the POM‐WEC in heave direction. Figure 3e,f shows the rope length amplitude and maximum rope pulling velocity of the POM‐WEC under regular waves with different heights and periods, respectively. As the wave height grows, both the rope length amplitude and the maximum rope pulling velocity are enhanced. This is attributed to that greater wave height directly expands the motion range and elevates the velocity of the POM‐WEC in heave direction. When prolonging wave period, the rope length amplitude is enhanced while the maximum rope pulling velocity is declined. The associated increase in wavelength expands the motion range of the POM‐WEC in surge direction, thereby contributing to the enhancement of the rope length amplitude. However, the prolonged wave period declines the velocity of the POM‐WEC in heave direction. Dataset S1 (Supporting Information) provides detailed motion trajectories of the POM‐WEC, the variations in rope length and rope pulling velocity, as well as displacement and velocity in both the surge and heave directions under regular waves with various heights and periods.

Figure 3g presents the output voltage waveform of the EPTO system under the regular wave, at a load resistance of 2.5 kΩ. The peak power can reach up to 10 mW, while the average power is approximately 1.2 mW. Figure 3h,i shows the peak power and average power of the EPTO system under regular waves with different heights and periods, respectively. As the wave height grows, both the peak power and average power increase. When prolonging wave period, both the peak power and average power decrease. Based on the performance characterization of the EPTO system, further increases in excitation amplitude cannot significantly improve output performance once the EPTO system can be effectively driven. Therefore, the decline of the rope pulling velocity caused by the prolonged wave period directly reduces output power. Moreover, the increasing excitation frequency caused by the shortened wave period does not reduce peak power due to the concurrent enhancement of rope pulling velocity. Dataset S2 (Supporting Information) includes output voltage waveforms of the EPTO system under regular waves with various heights and periods, measured at a load resistance of 2.5 kΩ.

#### 2.4.2 POM‐WEC in Focused Wave

**Figure 4:**
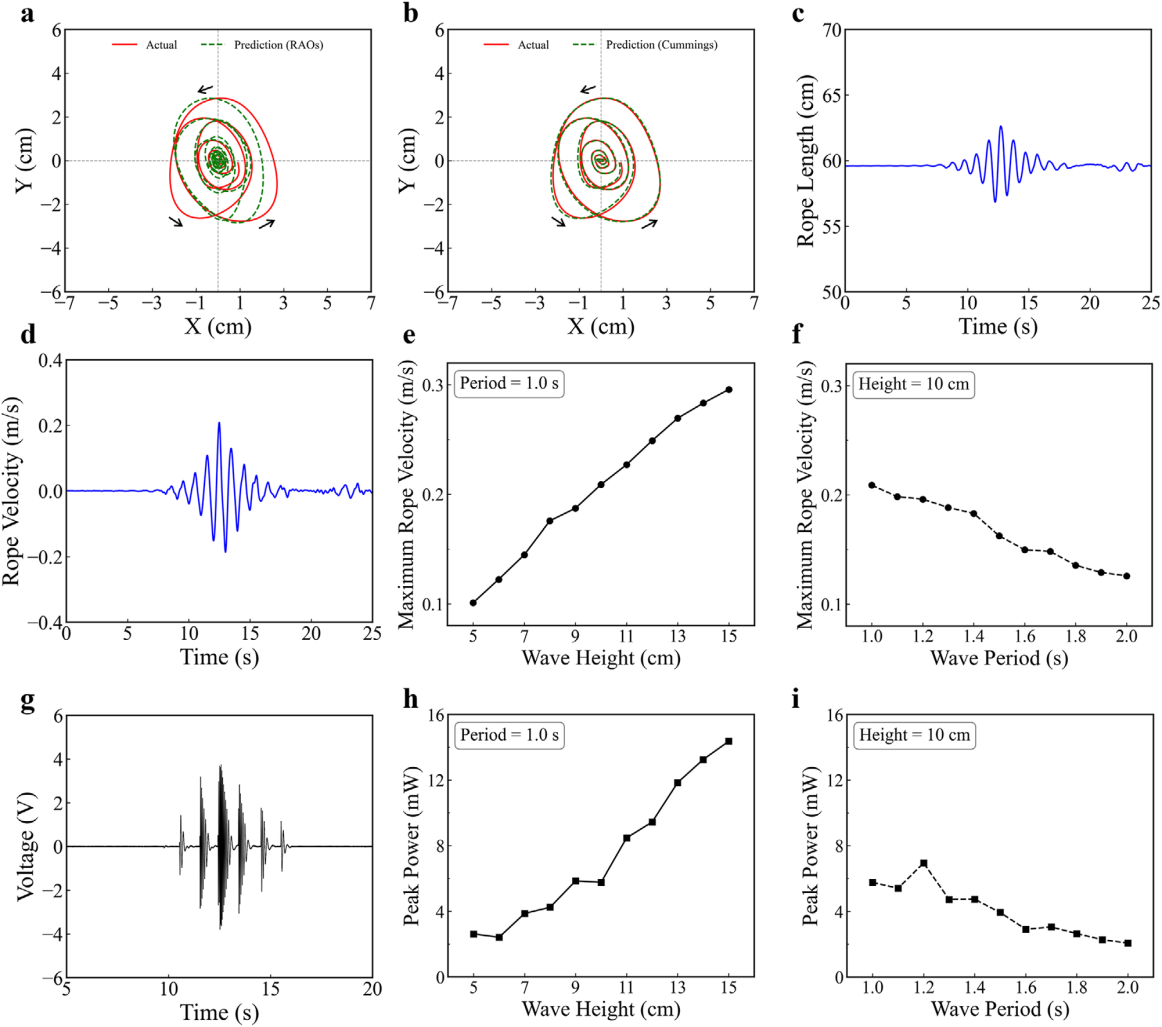
Motion and output performance of the POM‐WEC under focused waves with 1 s period and 10 cm height. a) The comparison between the theoretical prediction trajectory using RAOs and the actual motion trajectory. b) The comparison between the theoretical prediction trajectory using Cummins equation and the actual motion trajectory. c) The variations in rope length. d) The variations in velocity along the rope direction. e) The maximum rope pulling velocity of the POM‐WEC under focused waves with different heights. f) The maximum rope pulling velocity of the POM‐WEC under focused waves with different periods. g) The output voltage waveform of the EPTO system. h) The peak power of the POM‐WEC under focused waves with different heights. i) The peak power of the POM‐WEC under focused waves with different periods.

Recently, focused wave groups have been used as a practical tool to study nonlinear effects in a controlled laboratory environment. These wave groups are constructed by linearly superimposing multiple frequency components with a predefined phase relationship to concentrate energy at a specific time and location. Although the construction is based on linear theory, the resulting wave exhibits significant nonlinear characteristics near the focus point, making them an ideal testbed for evaluating the limits of linear models and the necessity of time‐domain simulation. Figure S13 (Supporting Information) shows the profile of the focused wave with a period of 1 s and a height of 10 cm. Video S2 (Supporting Information) shows the motion response of the POM‐WEC under the focused wave. Figure S14 (Supporting Information) shows the actual motion trajectory of the POM‐WEC under the focused wave. Figure S15 (Supporting Information) shows the variations in the displacement and velocity of the POM‐WEC in both the surge and heave directions. It can be observed that the POM‐WEC exhibits additional drift in surge direction under the focused wave conditions. To better analyze the motion response, the additional drift is removed from the actual motion trajectory. **Figure** **4**a displays the RAO‐based theoretical prediction under the focused wave and the prediction error is relatively large due to strong nonlinear wave‐body interactions and the higher‐order wave effects. On the other hand, Figure 4b shows the theoretical prediction based on Cummins equation under the focused wave, which shows excellent agreement with the actual motion trajectory. This improved accuracy is attributed to the Cummins equation's ability to incorporate nonlinearities, capturing the time‐varying EPTO response and accounting for the complex interactions introduced by the focused wave. The capability of the Cummins equation to model nonlinear behaviors makes it a more suitable approach for accurately predicting the motion response of WECs in real ocean environments.

Based on the motion trajectory, the variations in rope length and the velocity along the rope direction can be obtained, as shown in Figure 4c,d. Under the focused wave, the rope length amplitude can reach about 5.8 cm and the maximum rope pulling velocity can reach about 0.21 m s^−1^. Figure 4e,f shows maximum rope pulling velocity of the POM‐WEC under focused waves with different heights and periods. The maximum rope pulling velocity rises with increasing wave height but declines as the wave period becomes longer. Dataset S3 (Supporting Information) provides detailed motion trajectories of the POM‐WEC, the variations in rope length and rope pulling velocity, as well as displacement and velocity in both the surge and heave directions under focused waves with various heights and periods. Figure 4g shows the output voltage waveform of the EPTO system under the focused wave, at a load resistance of 2.5 kΩ. The shape of the voltage waveform corresponds closely to the focused wave profile shown in Figure S13 (Supporting Information). The peak power can reach 5.7 mW. Figure 4h,i displays the peak power of the EPTO system under focused waves with different heights and periods. As the wave height grows, the peak power increases due to the enhancement of the rope pulling velocity. When the wave period is prolonged, the peak power decreases due to the decline of the rope pulling velocity. Dataset S4 (Supporting Information) includes output voltage waveforms of the EPTO system under focused waves with various heights and periods, measured at a load resistance of 2.5 kΩ.

### 2.5 Self‐Powered Wireless Sensing Application Based on the POM‐WEC

**Figure 5:**
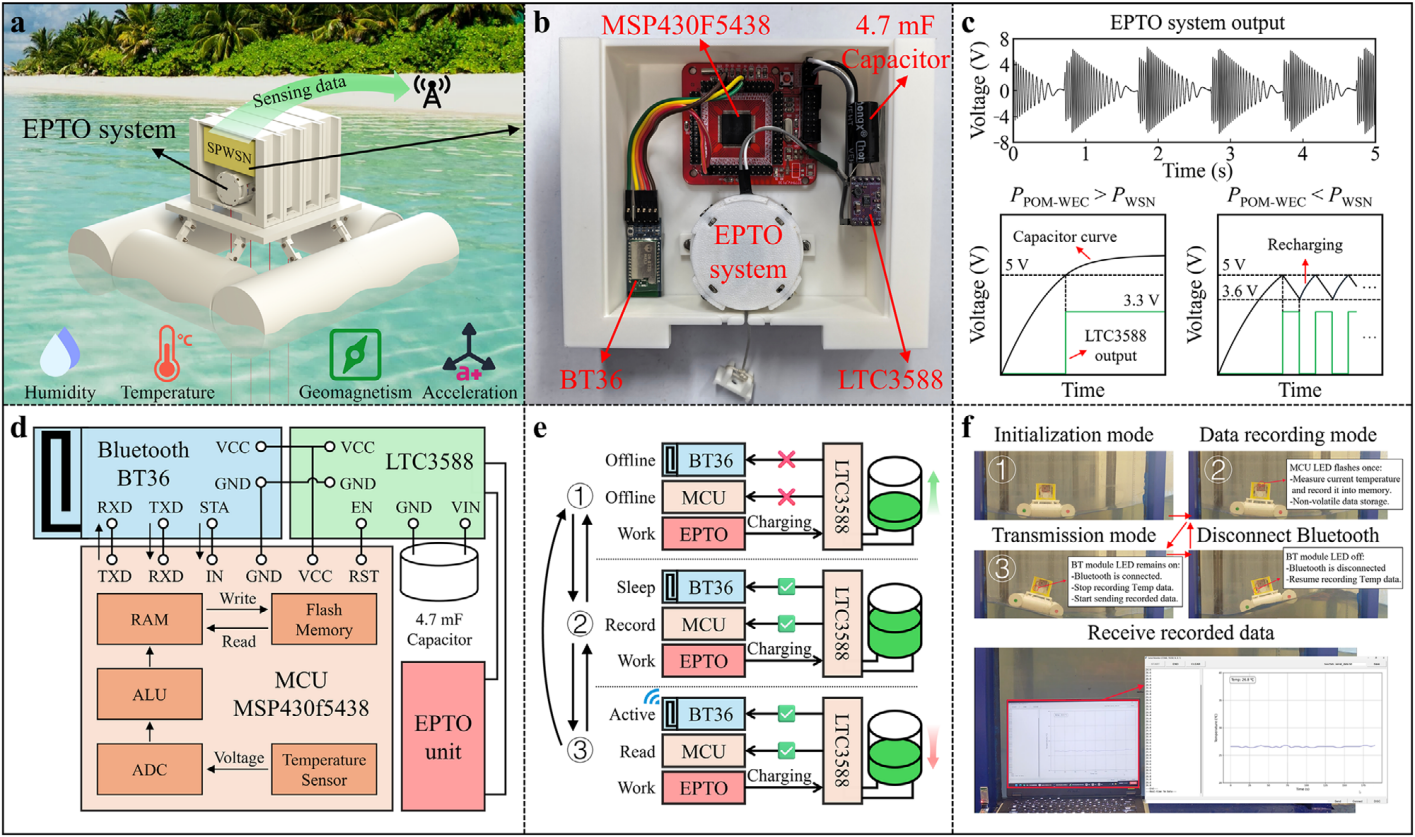
Self‐powered wireless sensing application based on the POM‐WEC. a) The nearshore application scenario of SPWSNs based on the POM‐WEC. b) The physical photograph of the SPWSN. c) The voltage waveform of the EPTO system, input capacitor, and LTC3588 output. d) The architecture schematic of the SPWSN. e) Three operation modes of the SPWSN. f) The flexible switching of operating modes according to different requirements.

As illustrated in **Figure** **5**a, each POM‐WEC can be equipped with one or more self‐powered wireless sensing nodes (SPWSN) as needed to achieve various monitoring functions, such as temperature, humidity, UV intensity, acceleration, and geomagnetism. Taking temperature monitoring as an example, we developed a SPWSN based on the EPTO system and the physical photograph is shown in Figure 5b. The EPTO system is fully integrated within the POM‐WEC to power the internal SPWSN and transmit sensing data wirelessly, eliminating the need for external cables. Furthermore, the entire PTO system (excluding the tensile rope) and SPWSN are packaged within a sealed compartment protected by a cover plate, as illustrated in Figure S16 (Supporting Information). This design effectively shields the internal components from direct seawater splash and immersion, ensuring the long‐term operational reliability of the POM‐WEC. The SPWSN is composed of an energy management module LTC3588, a 4.7 mF input capacitor, an MCU MSP430F5438 and a Bluetooth module BT36. Figure 5c displays the output voltage waveform of the EPTO system when the POM‐WEC is under the regular wave with a period of 1 s and a height of 15 cm, demonstrating an almost continuous power generation. The electrical energy generated by the EPTO system is accumulated in the input capacitor through a full‐wave bridge rectifier integrated into the LTC3588. When the voltage across the input capacitor exceeds a predefined threshold value of 5 V, the LTC3588 will transfer a portion of the stored charge to the output at a regulated voltage of 3.3 V. If the voltage across the input capacitor drops below 3.6 V during this process, the LTC3588 will disable the output. Depending on the power output of the POM‐WEC, the SPWSN can operate either continuously without interruption or intermittently, as illustrated in Figure 5c.

Figure 5d shows the schematic architecture of the SPWSN. The SPWSN can work at three operation modes: initialization mode (Mode 1), data recording mode (Mode 2), and data transmission mode (Mode 3), as illustrated in Figure 5e. In Mode 1, the voltage across the input capacitor is below the threshold value, and the EPTO system continuously charges the input capacitor. When the input voltage exceeds the threshold value, the output is enabled and the SPWSN switches from Mode 1 to Mode 2. In Mode 2, MCU measures current temperature data through a built‐in integrated sensor and saves the data in flash memory to achieve non‐volatile data storage. Since Bluetooth is disconnected, the BT36 module remains in sleep mode. The SPWSN in Mode 2 features an ultra‐low average power consumption of less than 1 mW. When Bluetooth connection is established, the SPWSN switches from Mode 2 to Mode 3. In Mode 3, the BT36 module switches to active mode and wirelessly transmits the incoming data to the receiving terminal. MCU pauses measuring and saving the temperature data but starts reading the saved data and sending them to the BT36 module. After sending all saved data, the MCU switches to standby mode. Throughout the entire process, if the input voltage drops below 3.6 V, the SPWSN will immediately switch to Mode 1. The POM‐WEC can flexibly switch operating modes according to different requirements, as shown in Figure 5f. Video S3 (Supporting Information) demonstrates the self‐powered wireless sensing application of the POM‐WEC under regular waves with a period of 1 s and a height of 15 cm. Every 5 seconds, the POM‐WEC‐based SPWSN collects temperature data and stores it in flash memory. When a Bluetooth connection is established, all previously stored data are wirelessly transmitted to the receiver. After the connection is terminated, the SPWSN resumes data collection and storage.

## 3 Conclusion

To efficiently extract ocean wave energy, this paper proposed a POM‐WEC integrating a high‐performance EPTO system. The EPTO system can convert the low‐frequency and low‐speed wave excitation into high‐speed inertial rotational motion of the rotor. Under an excitation velocity of 0.5 m s^−1^, the EPTO system can continuously output for approximately 3.5 s, with a peak‐to‐peak open‐circuit voltage of around 33.2 V. its peak power can reach 191.2 mW at a load resistance of 0.18 kΩ and average power can reach 9.1 mW at a load resistance of 2 kΩ, demonstrating the high performance of the EPTO system. Various factors influencing the output performance were also analyzed. Decreasing the magnet‐to‐coil distance or increasing the excitation velocity will increase the output performance. At a constant excitation velocity, once the rotor can be effectively driven, further increasing the excitation amplitude cannot significantly improve the rotor speed, leading to saturation in the output power. As the excitation frequency increases, the peak power decreases and average power increases.

A comprehensive methodology was presented to analyze and predict the motion response of the POM‐WEC under different wave conditions. Starting from the linear frequency‐domain formula for calculating RAOs, the analysis was further extended to the time‐domain using the Cummins equation to incorporate the influence of the EPTO system. Experimental calibration, including damping identification and the EPTO system characterization, was incorporated to improve model accuracy. Focused wave tests were employed to assess the influence of nonlinear effects. The proposed methodology aims to provide a robust platform for modelling the POM‐WEC under realistic wave conditions, with implications for both hydrodynamic analysis and PTO system optimization. A visual marker tracking method was used to capture the actual motion response of the POM‐WEC under both regular and focused wave conditions. By comparing the numerical simulation with the actual motion response, the validity and applicability of the proposed methodology was fully verified. The prediction results based on the Cummins equation show excellent agreement with experimental data.

The effects of wave height and frequency on the motion response of the POM‐WEC and the output performance of the EPTO system under both regular and focused waves were also tested and evaluated. As the wave height increases, the rope length amplitude, maximum rope pulling velocity, and output performance all increase. As the wave period increases, the rope length amplitude increases, but both maximum rope pulling velocity and output performance decrease. Although the experiments were conducted under moderate wave conditions (wave height 5–15 cm and period 1–2 s) to ensure good measurement control and repeatability, the results provide insight into understanding system hydrodynamic behavior. When scaled to practical ocean environments with higher waves and longer periods, the performance and stability of the POM‐WEC will strongly depend on the mooring configuration and PTO settings. Under such conditions, increased wave energy may cause larger mooring tension fluctuations, which could lead to rope fatigue and affect long‐term durability. Scaling up the size of the POM‐WEC and PTO system, and using thicker mooring rope, may be potential solutions. Further studies involving scaling‐up experiments and fully coupled numerical simulations will be carried out to assess these effects and optimize the design for realistic ocean conditions.

Moreover, a SPWSN based on the POM‐WEC was successfully developed, featuring non‐volatile data storage and three distinct operation modes. When Bluetooth is disconnected, the SPWSN switches to data recording mode to measure and record current temperature data. Once a Bluetooth connection is established, the SPWSN switches to data transmission mode to wirelessly transmit all saved data. This SPWSN can dynamically switch between modes to accommodate different application scenarios, providing an innovative strategy for self‐powered marine equipment and thereby motivating its further applications in oceanographic research and development.

## 4 Experimental Section

### Characterization of the EPTO System

Both the main structures of the POM‐WEC and the EPTO system were fabricated using PLA material through 3D printer. Figure S17 (Supporting Information) shows the schematic diagram and physical photograph of the excitation‐response testing system used to characterize the EPTO system. A motor controller CL‐01A converts the control program into multiple pulse signal segments with different numbers and frequencies of pulse signals. A motor driver DM542 is used to drive the stepper motor to rotate according to pulse signal segments, where the rotation direction, angle, and speed are determined by pulse polarity, number, and frequency, respectively. The rotation of the stepper motor is converted into the linear motion of a slider. Thus, the EPTO system can be driven by controllable linear excitation at a predefined velocity, amplitude, and frequency. The electric output signal of the EPTO system is measured by oscilloscope MSO21BL.

### Incident Wave Generation

Figure S18 (Supporting Information) shows the schematic diagram and physical photograph of the piston wave maker. By controlling the motion of the wave paddle, the wave maker can generate unidirectional regular and irregular waves with predefined spectra, frequency, and heights. The wave‐making section is 20 m long and 0.8 m wide, with a still water depth of 0.5 m. The POM‐WEC is anchored at a position 6 m away from the wave paddle. In the case of a regular wave, the incident wave is assumed to be monochromatic and harmonic, represented by a single frequency ω. The first order wave elevation at a fixed location is typically written as:

(4) $$\eta^{\left(1\right)}\left(t\right)=a\cos\left(\omega t+\varphi \right)$$

where *t* is the time variable, *a* is the amplitude, and φ is the phase.

In realistic ocean environments, waves are not monochromatic but rather a random superposition of multiple wave components with varying frequencies and amplitudes. These waves are typically modeled as irregular waves, described statistically using a wave energy spectrum. In this study, the JONSWAP spectrum is adopted to characterize the frequency distribution of sea states. Focused waves are employed as a special and controlled realization of irregular waves to investigate the transient response of the POM‐WEC. Typical irregular waves are stochastic in nature, composed of wave components with random phases, while focusing waves are constructed from the same spectral content but with carefully chosen phases to achieve constructive interference at a predefined location and time. The first order focused wave group surface elevation can be constructed by discretizing the spectrum into *N* components and summing sinusoidal waves:

(5) $$\eta^{\left(1\right)}\left(x,t\right)=\sum_{n=1}^{N}a_{n}\cos\left(k_{n}\left(x-x_{f}\right)-\omega_{n}\left(t-t_{f}\right)\right)$$

where *x* denotes the longitudinal position along the wave flume, measured from the wave maker at *x* = 0, and *t* is the time variable. The parameters *x_f_* and *t_f_* represent the focus location and focus time, respectively. For the *n*‐order wave component from the wave spectrum, *a_n_* is the amplitude, *k_n_* is the wavenumber, and ω_*n*_ is the corresponding angular frequency. This approach retains the realistic frequency content of a natural sea state while concentrates energy into a single, large‐amplitude wave crest. Through the approach, we combine the benefits of the typical broadband spectral energy distribution of real seas, and the repeatability and controllability of deterministic wave fields.

### Hydrodynamic Response Tracking Method of the POM‐WEC

Under unidirectional waves, the POM‐WEC primarily moves within the plane defined by the surge and heave directions. We use a visual marker tracking method based on OpenCV to track the motion trajectory of the POM‐WEC in waves. Figure S19 (Supporting Information) shows the number, positions, and colors of the markers on the POM‐WEC. Two circular markers of different colors (green and red), each with a diameter of 2.5 cm, are placed 30 cm apart. Using two markers can effectively eliminate interference caused by pitch oscillation of the POM‐WEC and reduce measurement error. The motion trajectories of the POM‐WEC were recorded in video using 4K resolution at 60 fps. By identifying the pixel distance between the centers of the markers in video and mapping it to the actual physical distance of 30 cm, the mapped physical length per pixel can be determined. This allows the pixel displacement of the markers to be mapped to their actual motion trajectories. Video S4 (Supporting Information) demonstrates the effectiveness of the visual marker tracking method. The center of each marker is accurately identified, and the motion trajectory is clearly displayed.

### Data Processing and Statistical Analysis

The filtering and processing of the experimental data were performed using MATLAB and Python. Regarding peak power data, the top‐10 mean method was employed: the average was calculated from the ten highest peak power values generated by the EPTO system over 40 excitation cycles. As for the average power data, the value was calculated across the entire set of 40 excitation cycles. These methods effectively mitigate the impact of performance fluctuations in the EPTO output under continuous excitation, thereby ensuring that the resulting data are more representative and generalizable. For hydrodynamic response tracking of the POM‐WEC, the dilation‐erosion algorithm was applied to effectively suppress image noise and repair discontinuities in the markers, ensuring precise identification of the center of the markers. Furthermore, the raw motion trajectories of the POM‐WEC were processed using a Savitzky‐Golay filter followed by cubic spline interpolation to enhance their smoothness and continuity.

## Conflict of Interest

The authors declare no conflict of interest.

## Supporting information



Supporting Information

Supplemental Video 1

Supplemental Video 2

Supplemental Video 3

Supplemental Video 5

Supplemental Data

## Data Availability

The data that support the findings of this study are available in the supplementary material of this article.
