# Supplementary material for: A Pull‐Out Mooring Wave Energy Converter: Design, Analysis, and Application
Source: Adv Sci (Weinh). 2025 Nov 3;13(4):e16945. doi: 10.1002/advs.202516945 (PMC12822436; doi:10.1002/advs.202516945)
Supplement: Supplementary file 1 — Supporting Information [file ADVS-13-e16945-s003.docx]

***Supporting Figures***

Figure S1. The physical photograph of the POM-WEC.

Figure S2. The distribution of the magnetic flux density component normal to the coil plane (y-direction) for (a) AP arrangement and (b) SP arrangement.

Figure S3. The magnetic flux density variation under different air gaps for AP arrangement.

Figure S4. The output voltage waveform of the EPTO system with different load resistance under a single excitation.

Figure S5. The output duration of the EPTO system with different load resistance under a single excitation.

Figure S6. The matching resistance test of the EPTO system based on peak power and average power.

Figure S7. The peak current and average current of the EPTO system under different load.

Figure S8. The output voltage waveform of the EPTO system under different frequency of excitation.

Figure S9. The voltage output waveform of the PTO system under a 2.5 kΩ load throughout the durability test.

Figure S10. The morphological comparison of the tensile rope before and after the durability test.

Figure S11. The profile of the regular wave with a period of 1 s and a height of 10 cm.

Figure S12. The variations in the displacement and velocity of the POM-WEC in both the surge and heave directions under the regular wave with a period of 1 s and a height of 10 cm.

Figure S13. The profile and spectrum of the focused wave with a period of 1 s and a height of 10 cm.

Figure S14. The motion trajectory of the POM-WEC under the focused wave with a period of 1 s and a height of 10 cm.

Figure S15. The variations in the displacement and velocity of the POM-WEC in both the surge and heave directions under the focused wave with a period of 1 s and a height of 10 cm.

Figure S16. The EPTO system and the SPWSN in a sealed compartment.

Figure S17. The schematic diagram and physical photograph of the excitation-response testing system used to characterize the EPTO system.

Figure S18. The schematic diagram and physical photograph of the piston wave maker.

Figure S19. The markers used to track motion trajectory of the POM-WEC.


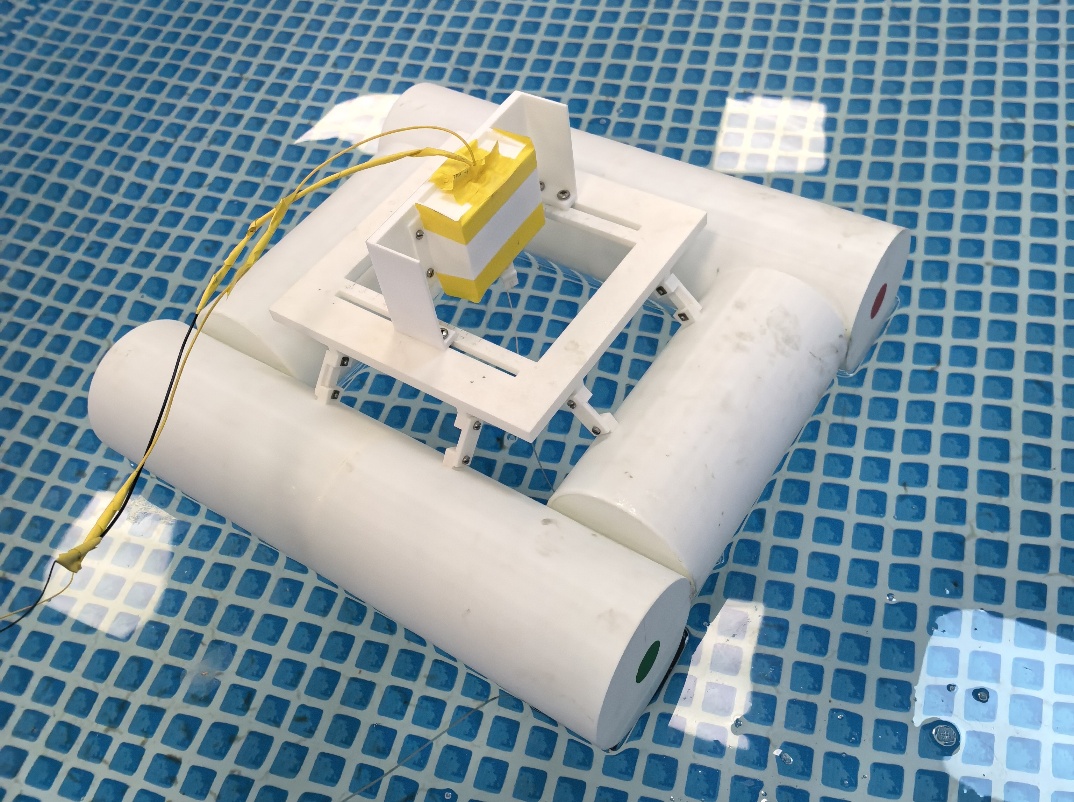


**Figure S1.** The physical photograph of the POM-WEC.


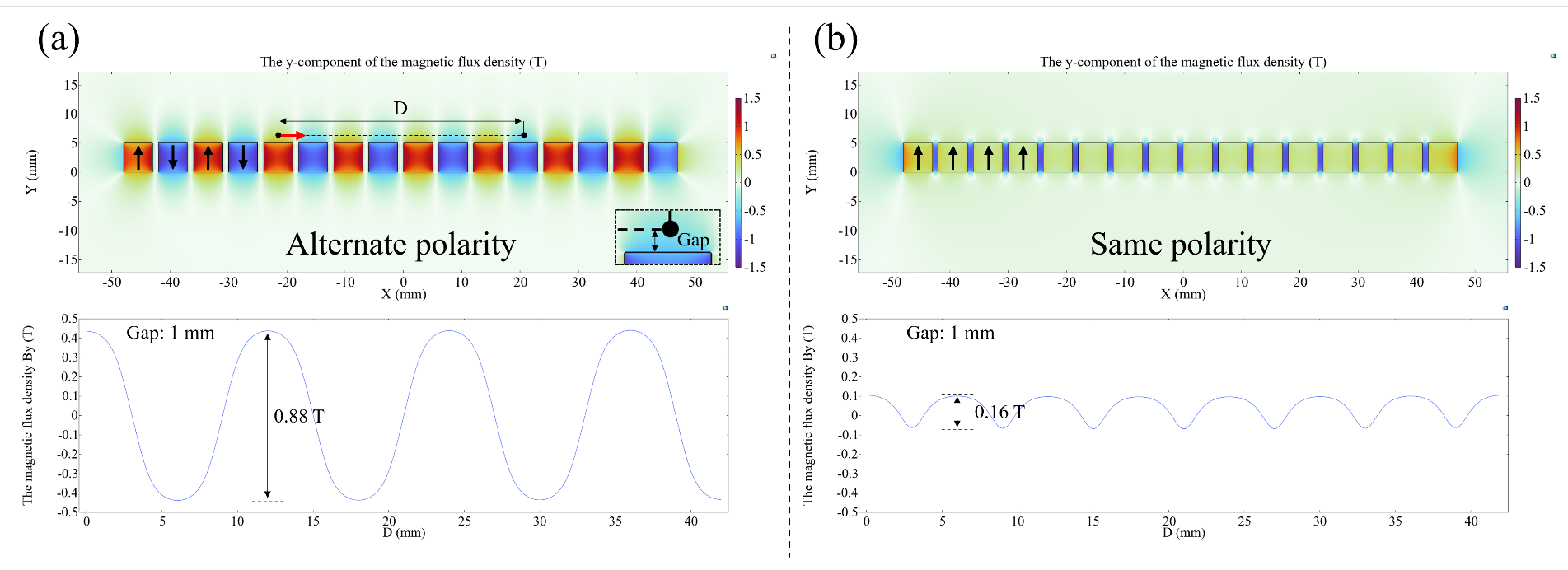


**Figure S2.** The distribution of the magnetic flux density component normal to the coil plane (y-direction) for (a) AP arrangement and (b) SP arrangement.


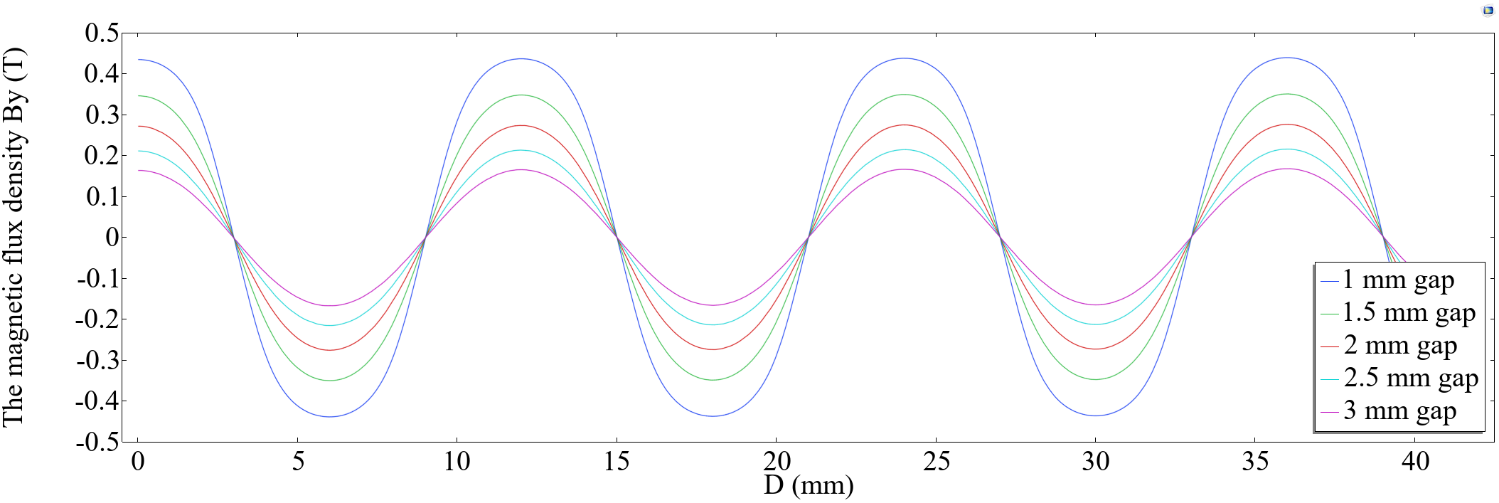


**Figure S3.** The magnetic flux density variation under different air gaps for AP arrangement.


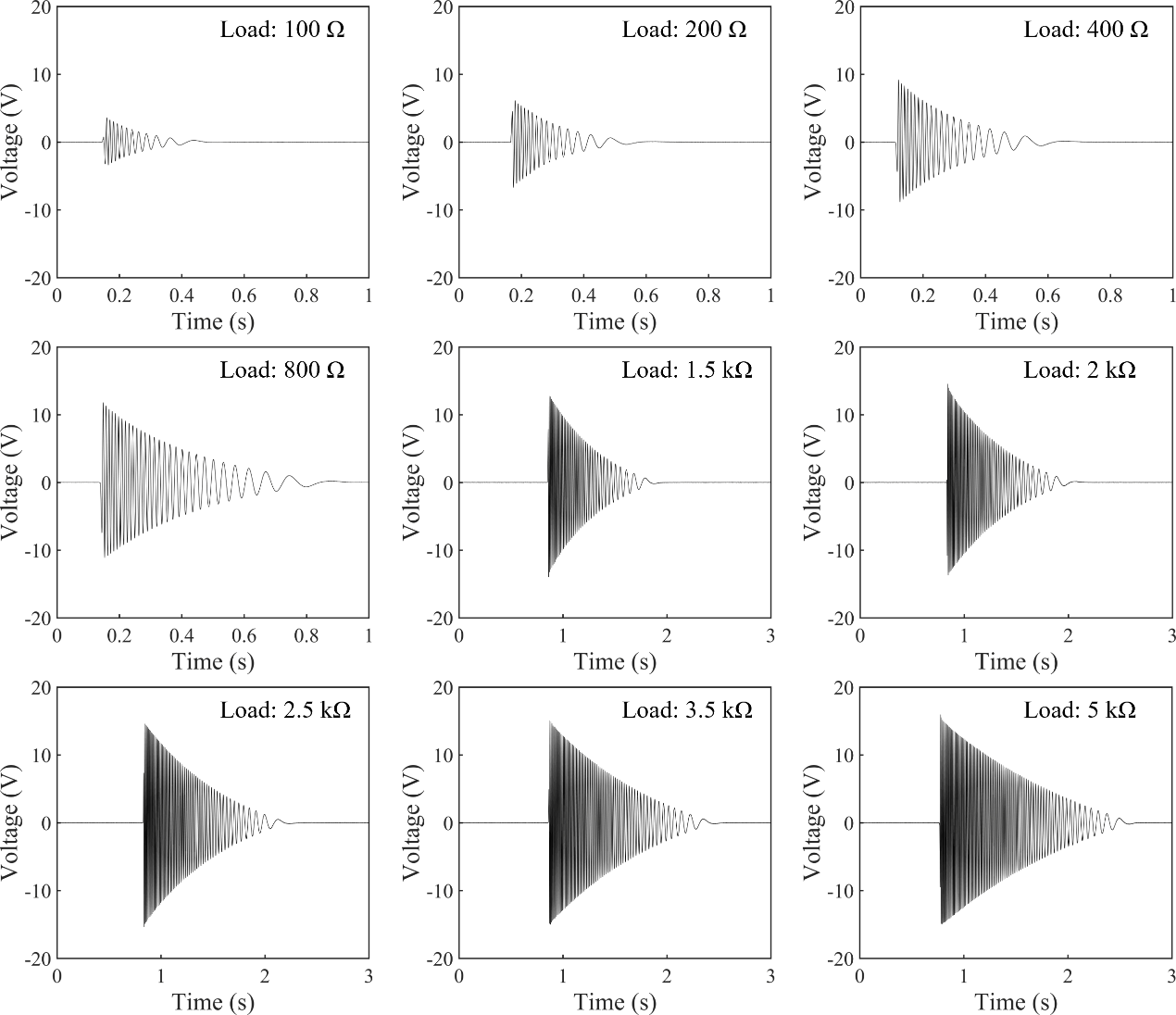


**Figure S4.** The output voltage waveform of the EPTO system with different load resistance under a single excitation.


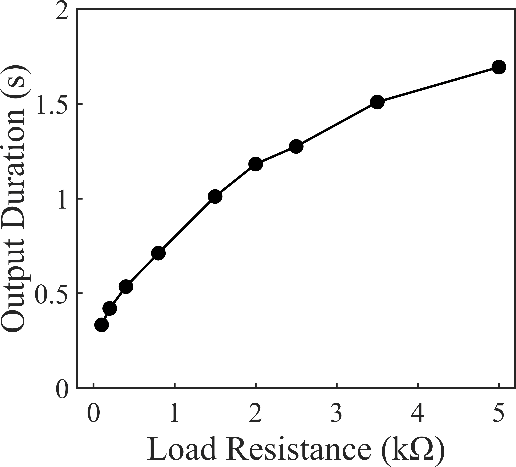


**Figure S5.** The output duration of the EPTO system with different load resistance under a single excitation.


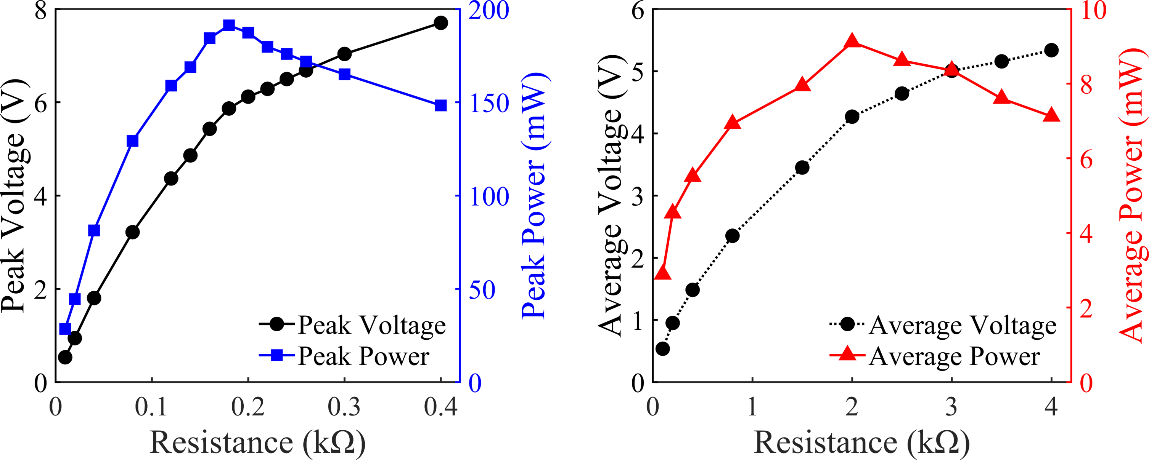


**Figure S6.** The matching resistance test of the EPTO system based on peak power and average power.


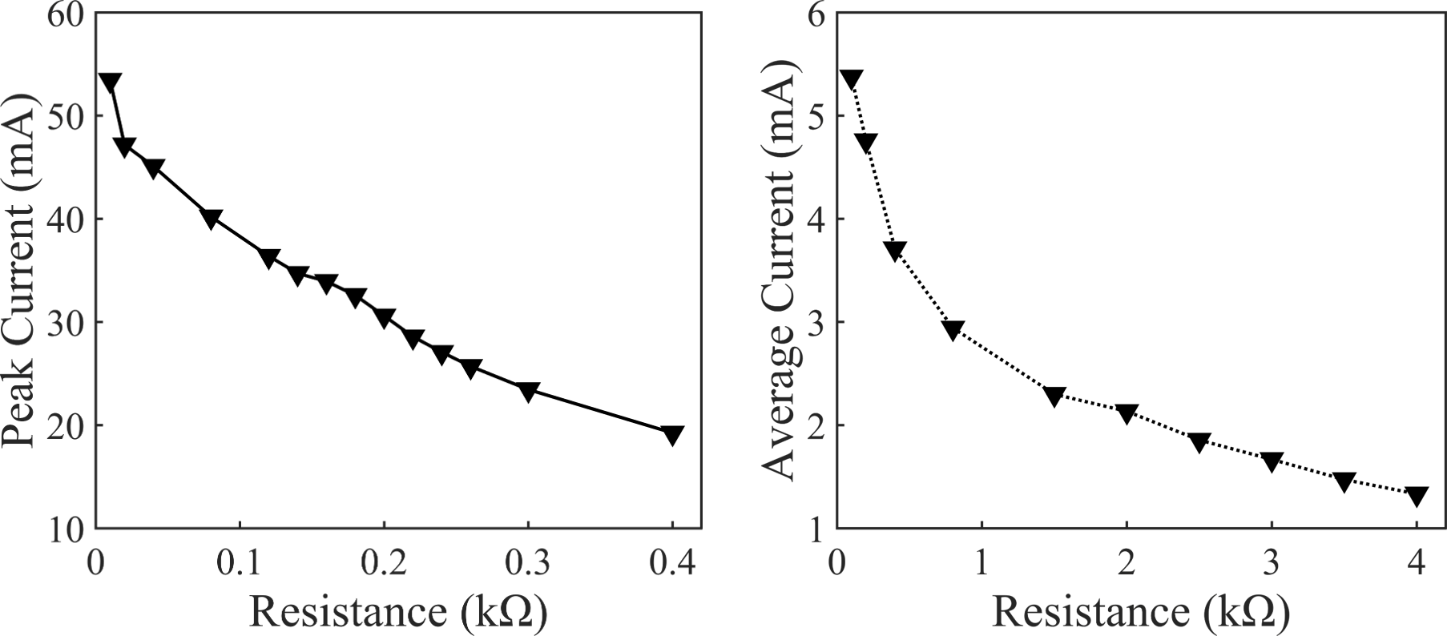


**Figure S7.** The peak current and average current of the EPTO system under different load.


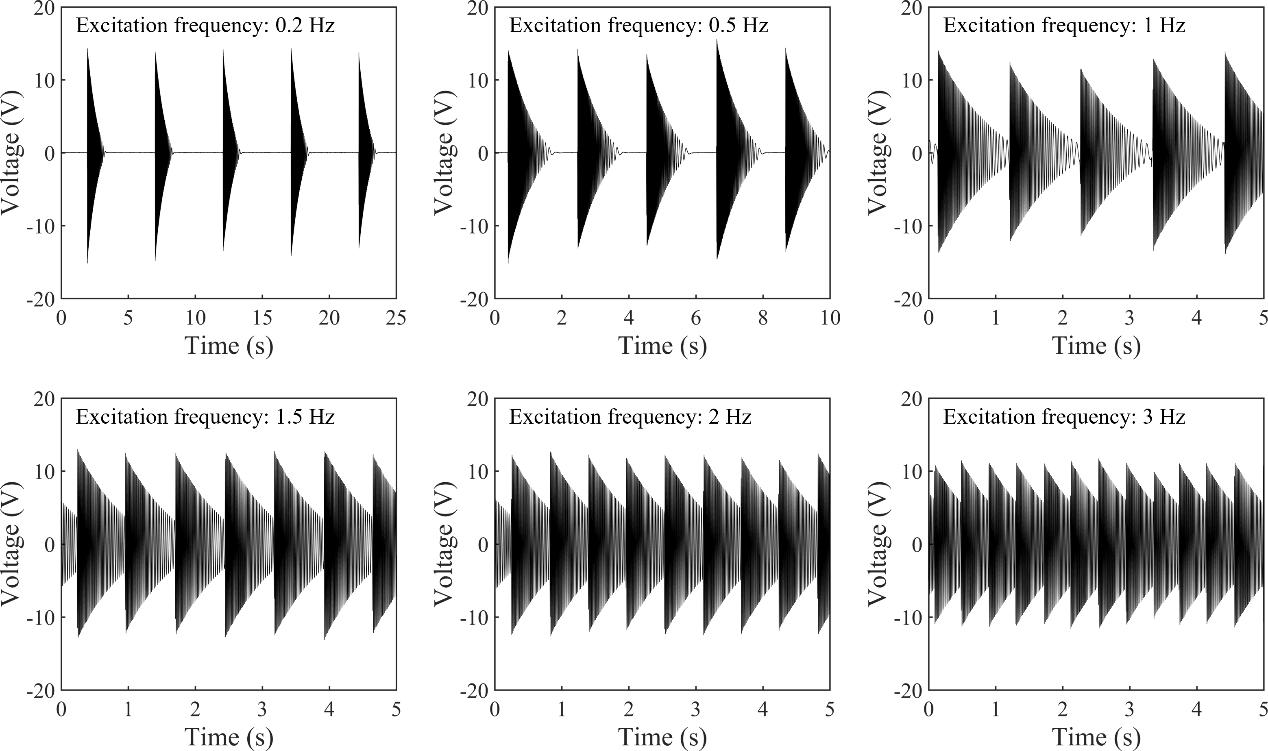


**Figure S8.** The output voltage waveform of the EPTO system under different frequency of excitation.


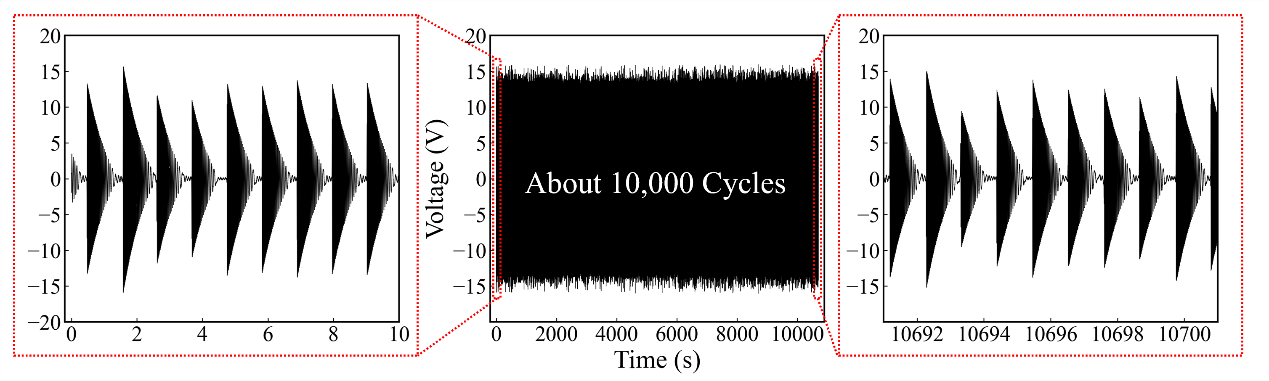


**Figure S9.** The voltage output waveform of the PTO system under a 2.5 kΩ load throughout the durability test.


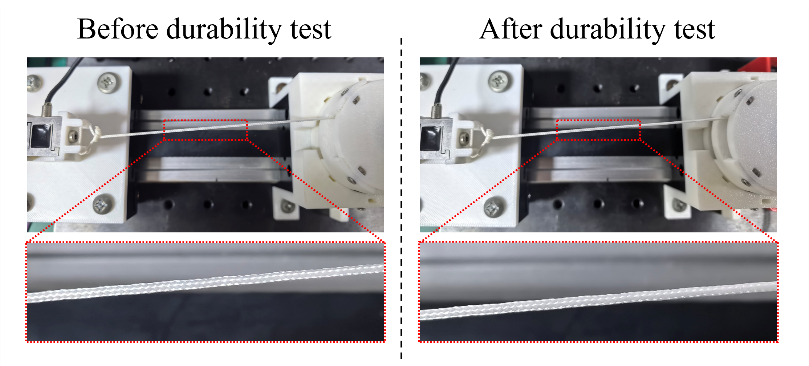


**Figure S10.** The morphological comparison of the tensile rope before and after the durability test.


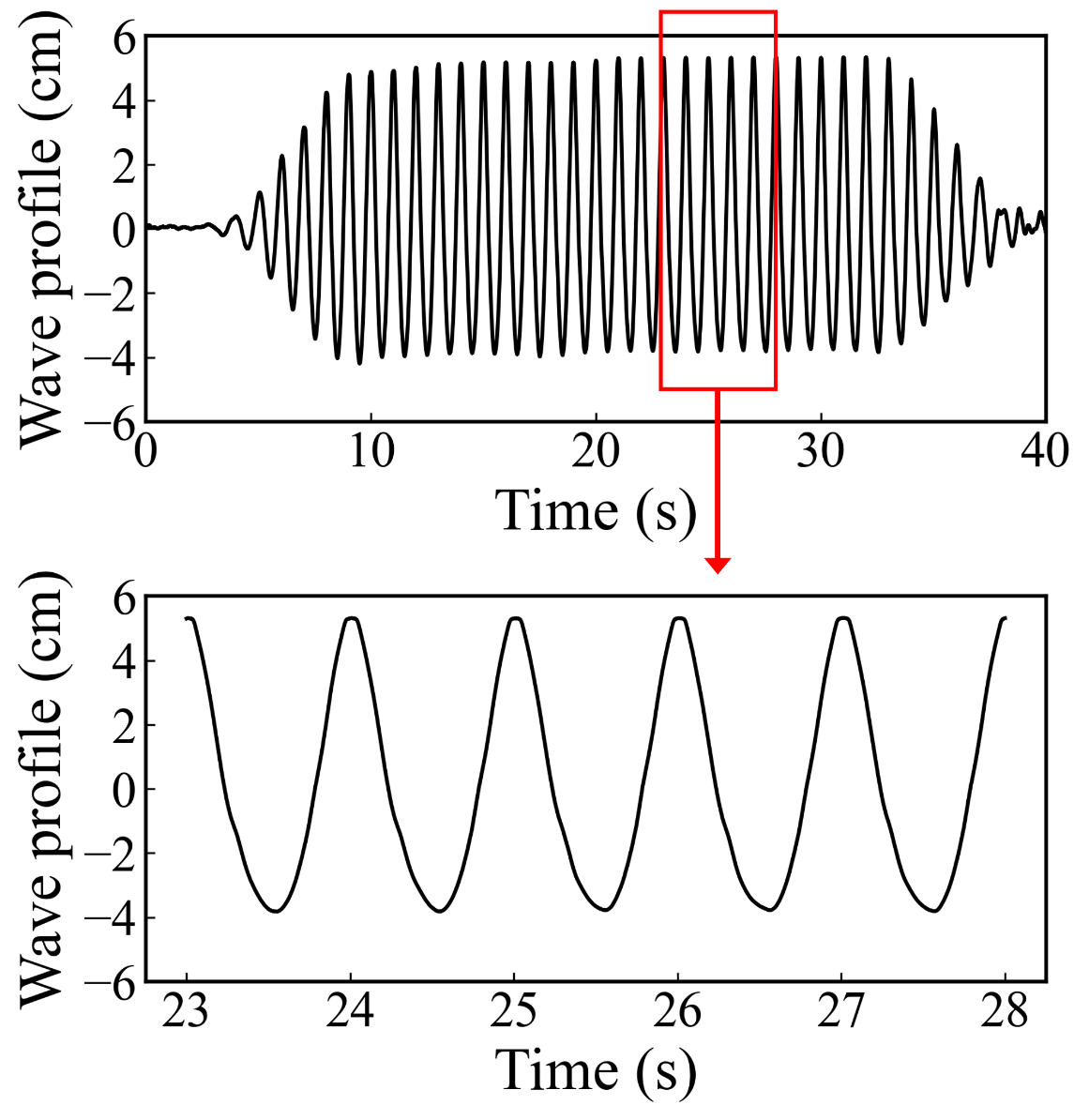


**Figure S11.** The profile of the regular wave with a period of 1 s and a height of 10 cm.


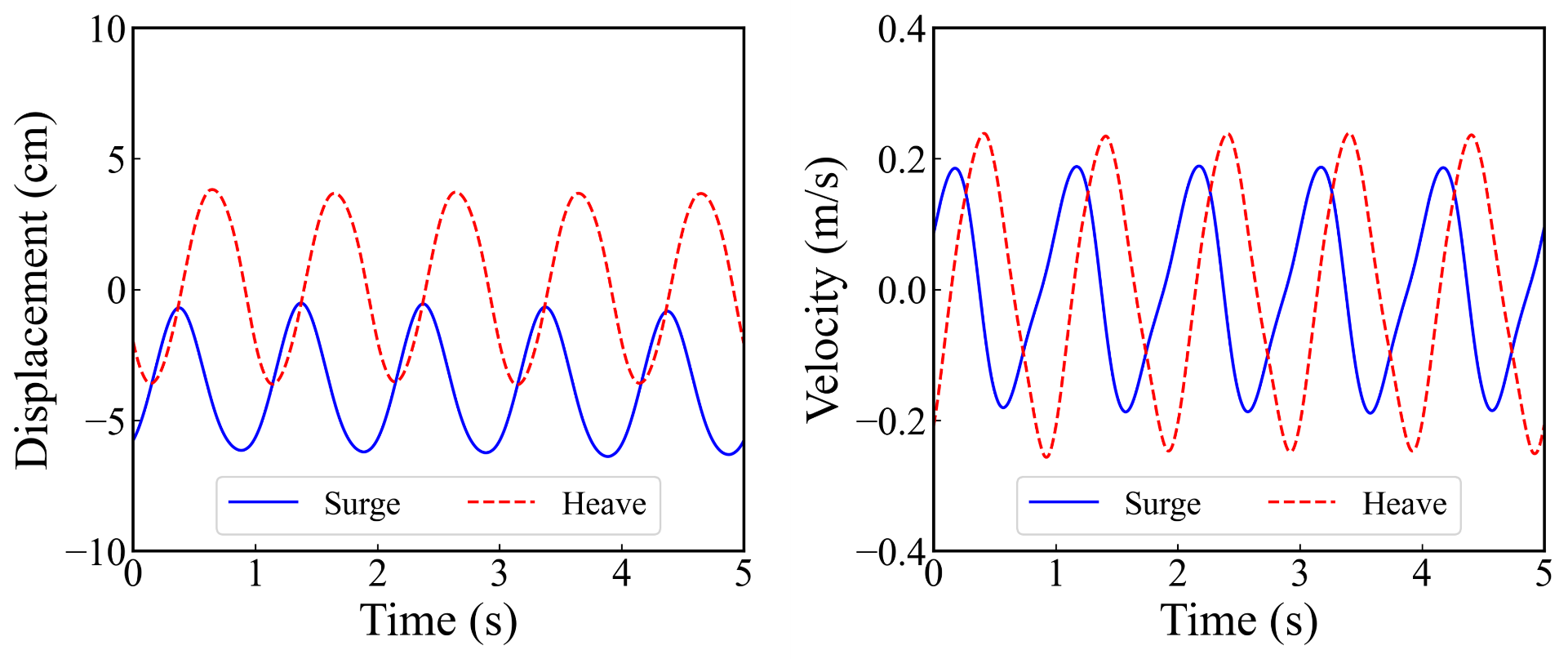


**Figure S12.** The variations in the displacement and velocity of the POM-WEC in both the surge and heave directions under the regular wave with a period of 1 s and a height of 10 cm.


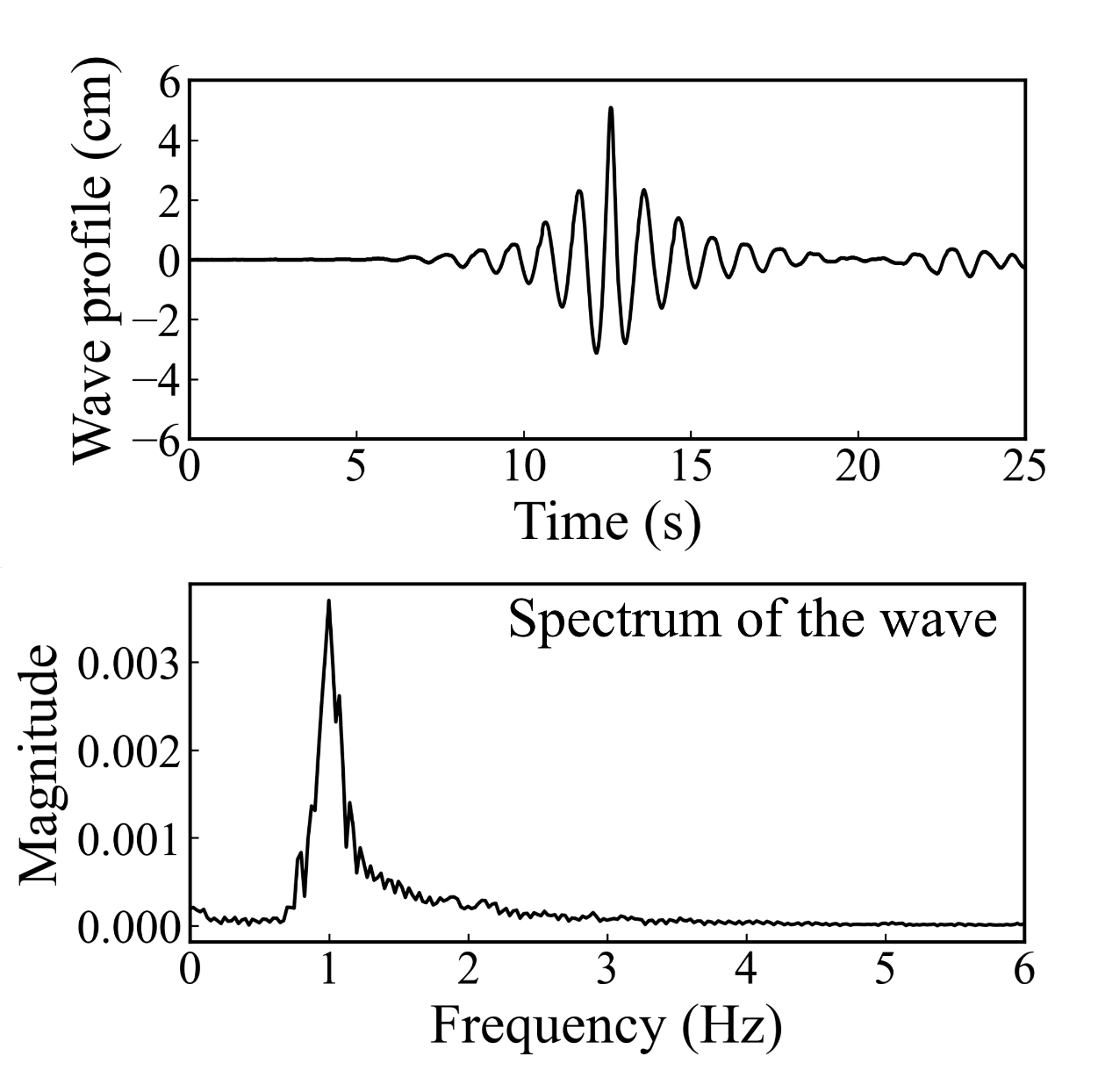


**Figure S13.** The profile and spectrum of the focused wave with a period of 1 s and a height of 10 cm.


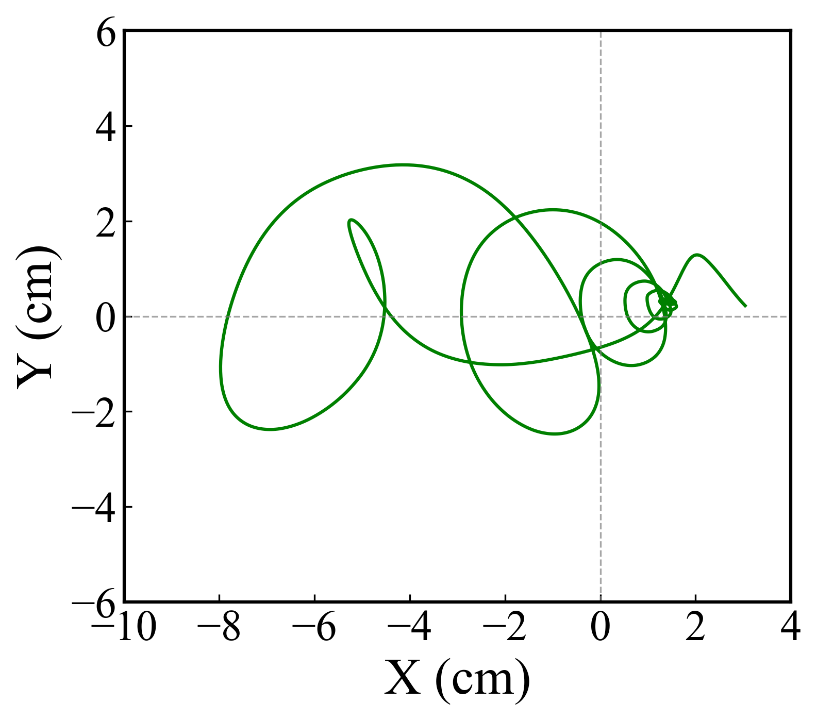


**Figure S14.** The motion trajectory of the POM-WEC under the focused wave with a period of 1 s and a height of 10 cm.


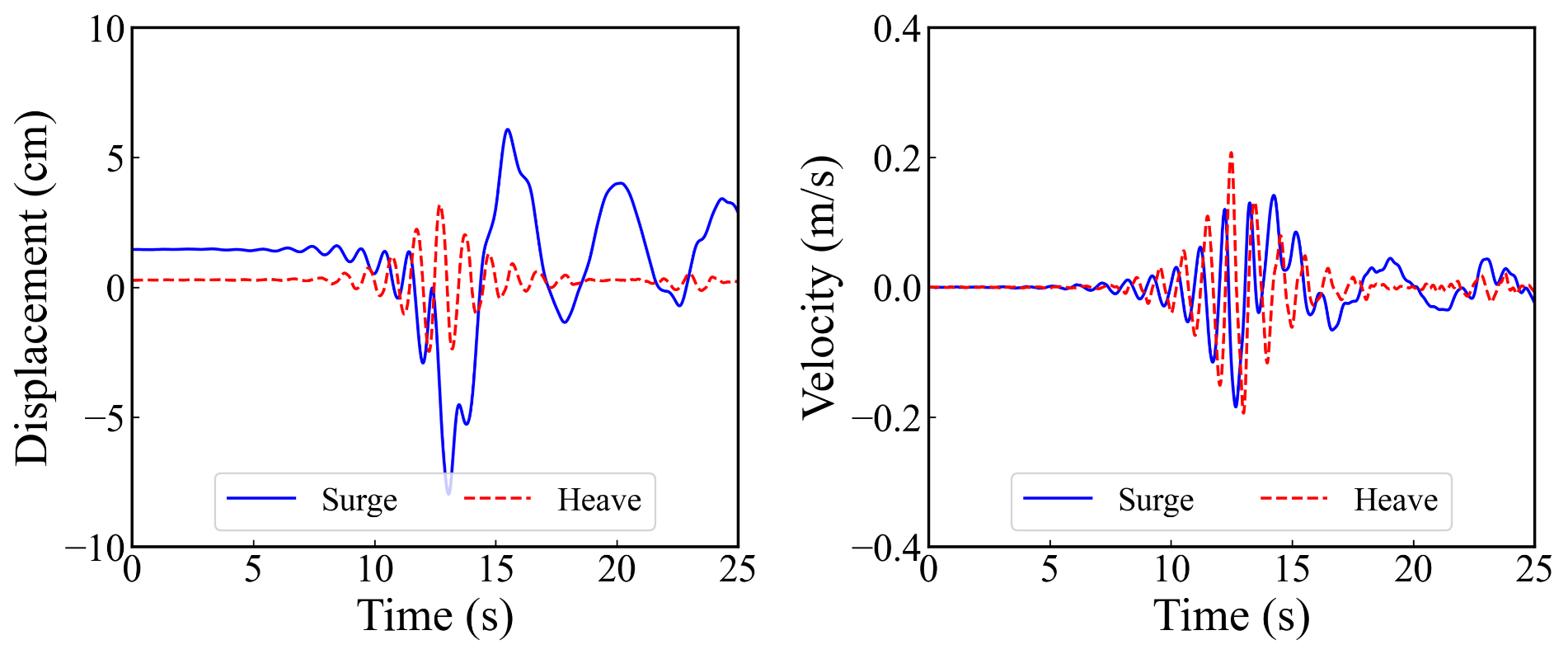


**Figure S15.** The variations in the displacement and velocity of the POM-WEC in both the surge and heave directions under the focused wave with a period of 1 s and a height of 10 cm.


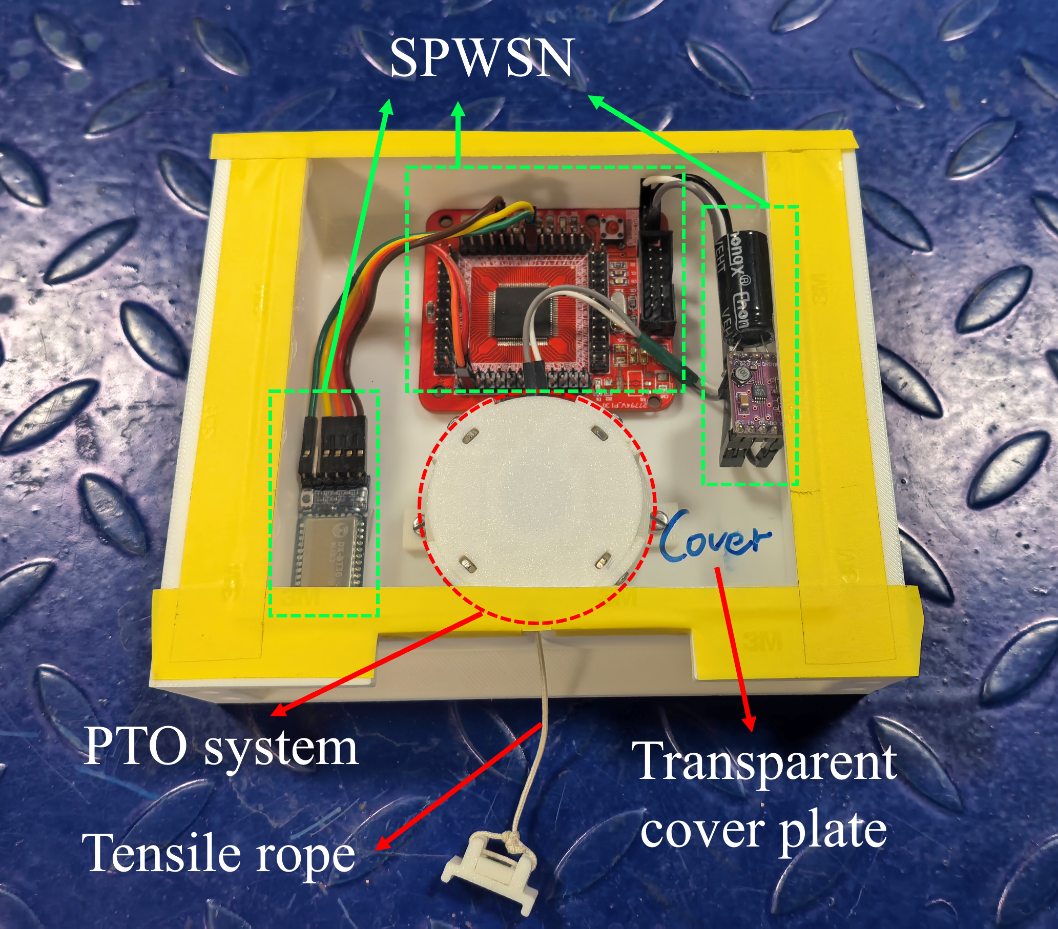


**Figure S16.** The EPTO system and the SPWSN in a sealed compartment.


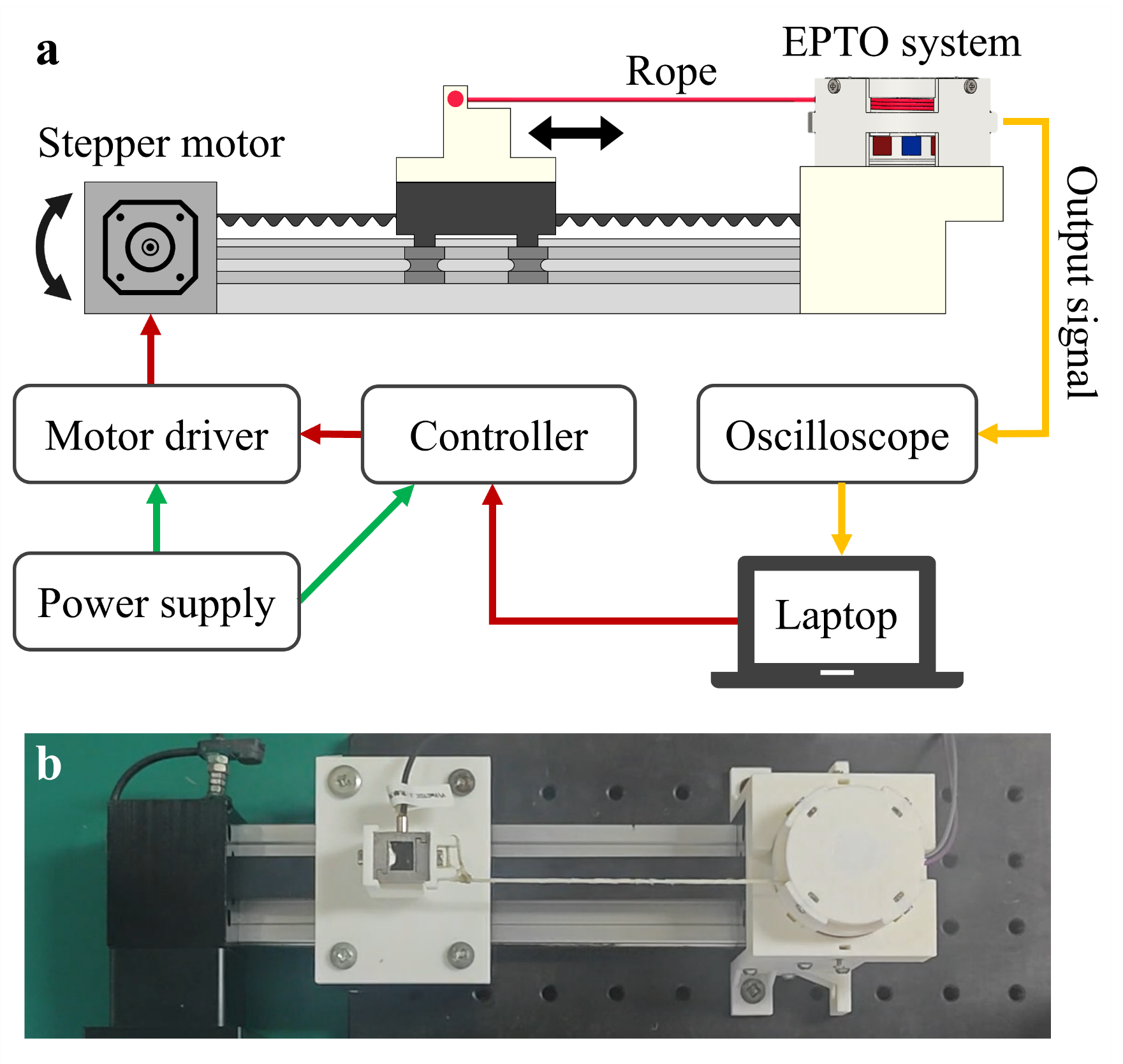


**Figure S17.** The schematic diagram and physical photograph of the excitation-response testing system used to characterize the EPTO system.


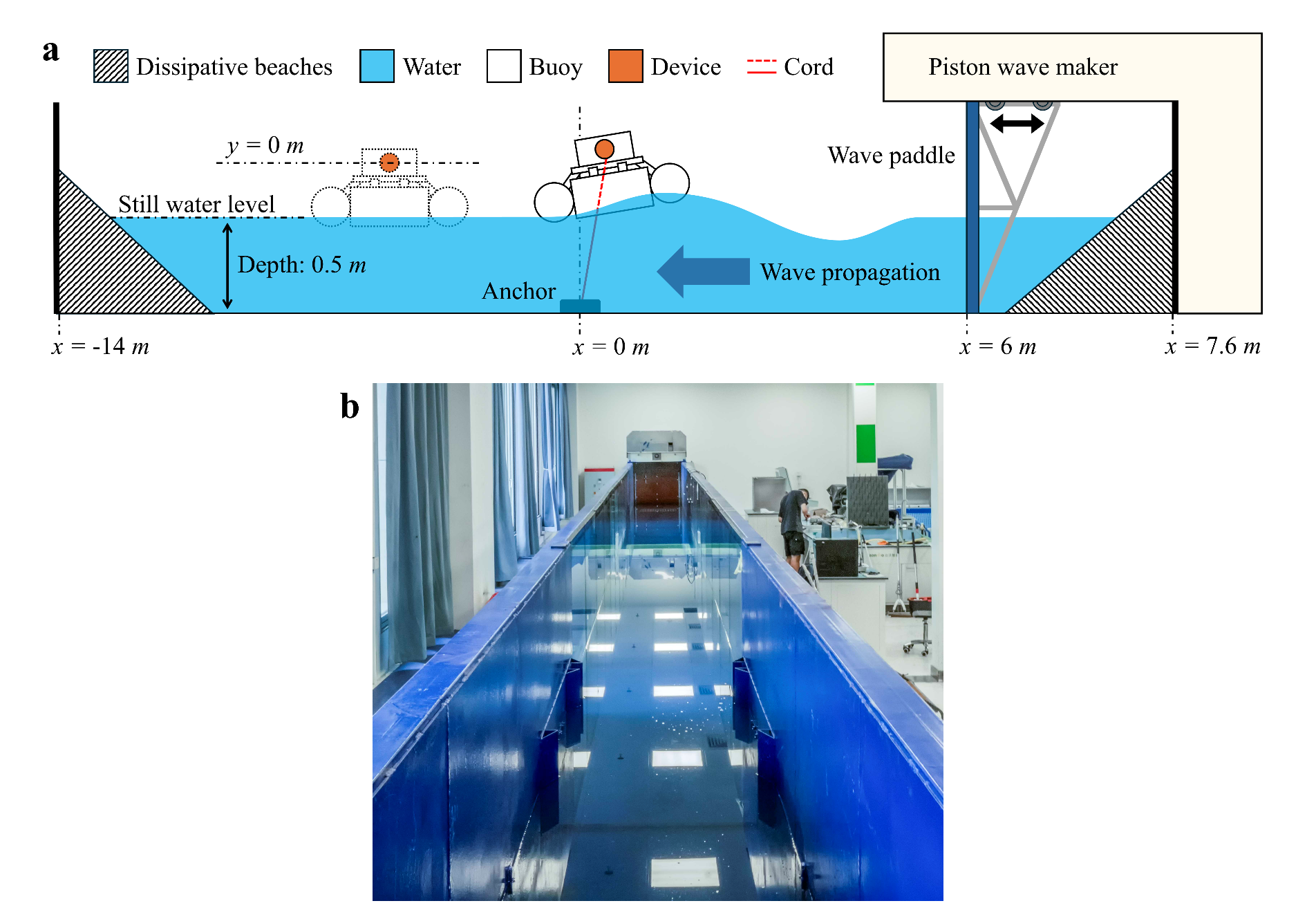


**Figure S18.** The schematic diagram and physical photograph of the piston wave maker.


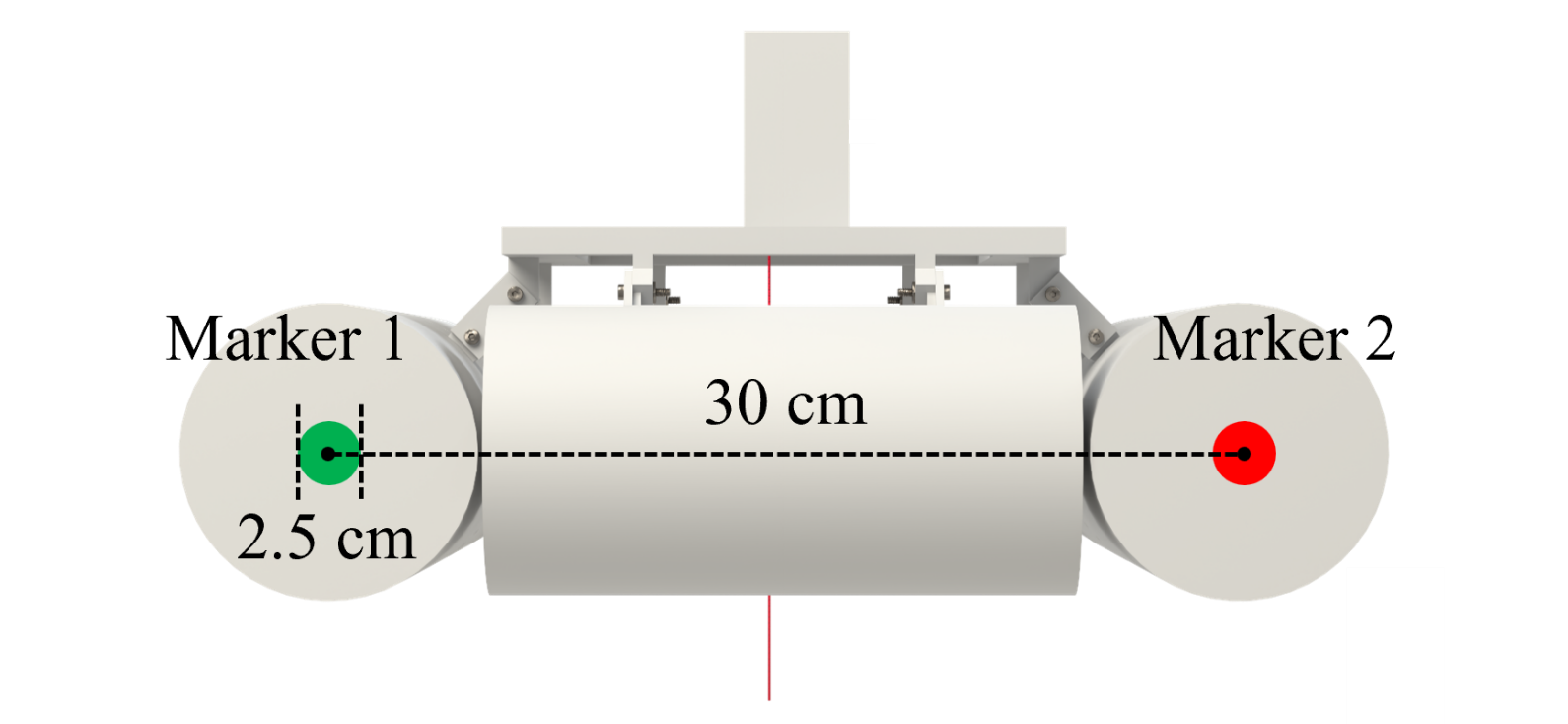


**Figure S19.** The markers used to track motion trajectory of the POM-WEC.

***Supporting Videos***

Video S1. The motion of the POM-WEC under the regular wave.

Video S2. The motion of the POM-WEC under the focused wave.

Video S3. The self-powered wireless sensing application of the POM-WEC.

Video S4. The demonstration of the visual marker tracking method.

***Supporting Datasets***

Dataset S1. The motion trajectories of the POM-WEC, the variations in rope length and rope pulling velocity, as well as displacement and velocity in both the surge and heave directions under regular waves with various heights and periods.

Dataset S2. The output voltage waveforms of the EPTO system under regular waves with various heights and periods, measured at a load resistance of 2.5 kΩ.

Dataset S3. The motion trajectories of the POM-WEC, the variations in rope length and rope pulling velocity, as well as displacement and velocity in both the surge and heave directions under focused waves with various heights and periods.

Dataset S4. The output voltage waveforms of the EPTO system under focused waves with various heights and periods, measured at a load resistance of 2.5 kΩ.
