# Supplementary material for: A Pull‐Out Mooring Wave Energy Converter: Design, Analysis, and Application
Source: Adv Sci (Weinh). 2025 Nov 3;13(4):e16945. doi: 10.1002/advs.202516945 (PMC12822436; doi:10.1002/advs.202516945)

Regular wave: Period1.0s_Height5cm

Trajectory

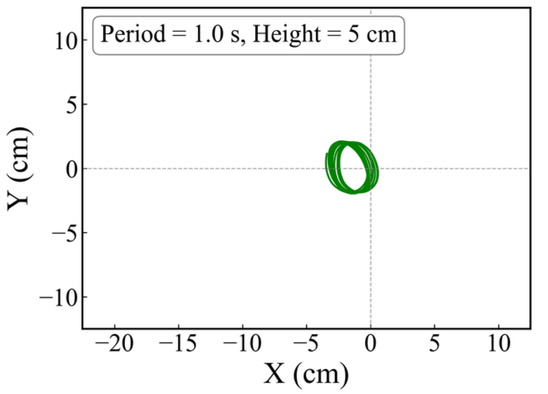

Displacement

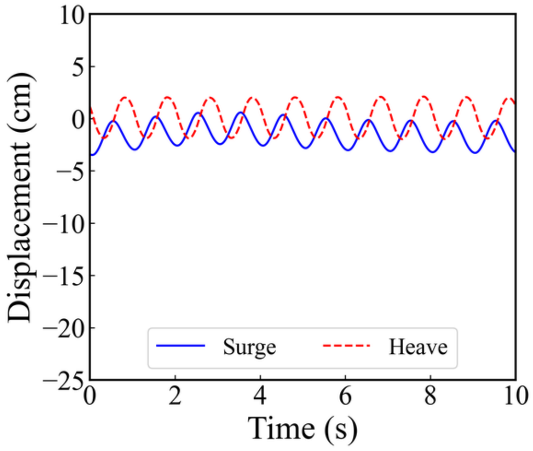

Velocity

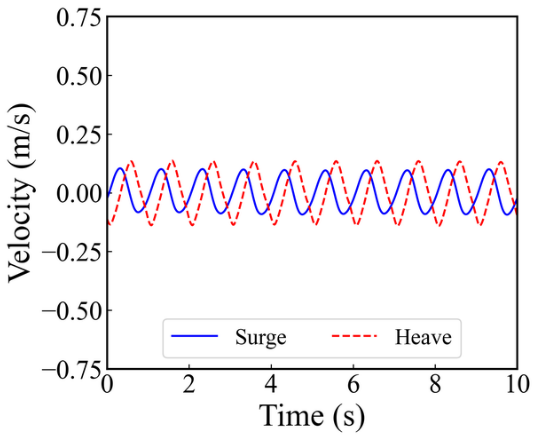

Rope Length

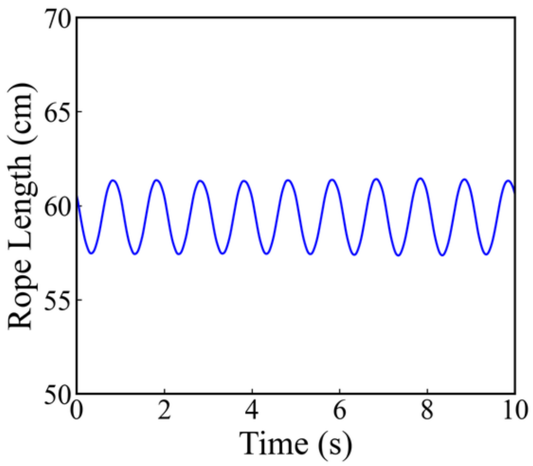

Rope Velocity

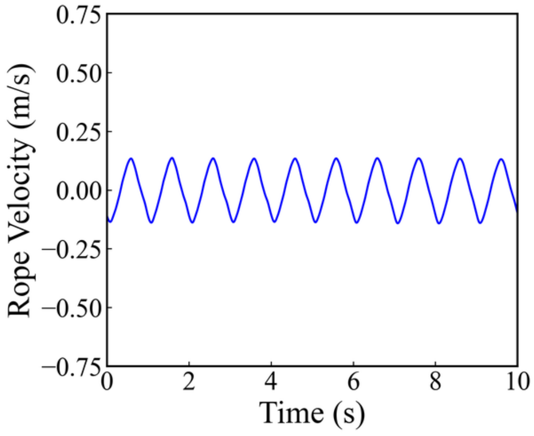

Regular wave: Period1.0s_Height6cm

Trajectory

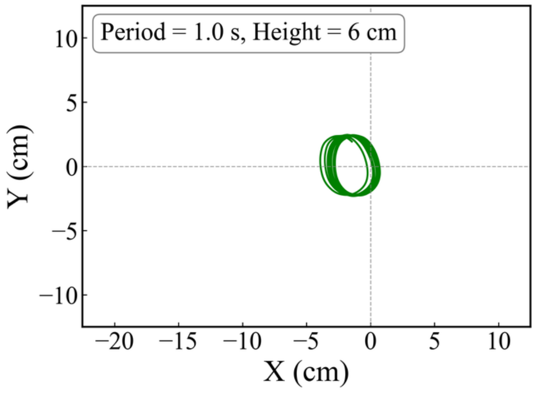

Displacement

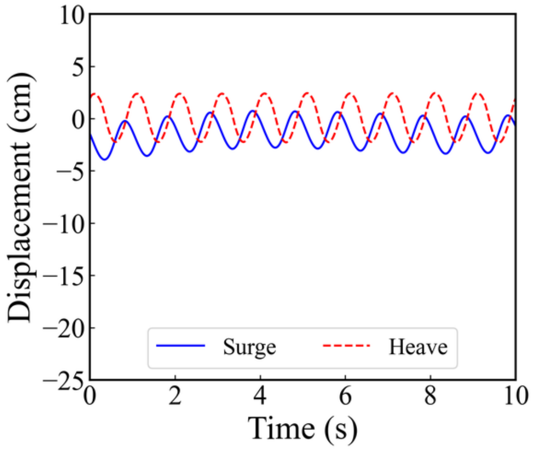

Velocity

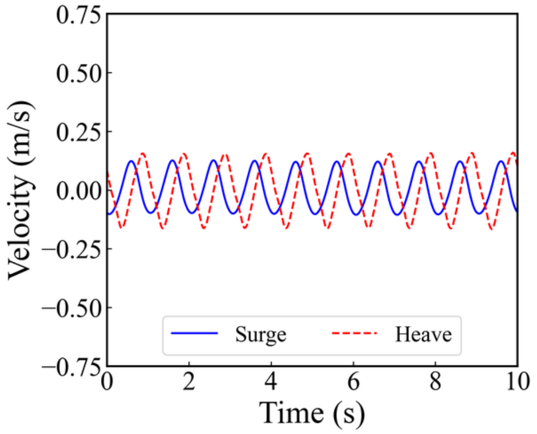

Rope Length

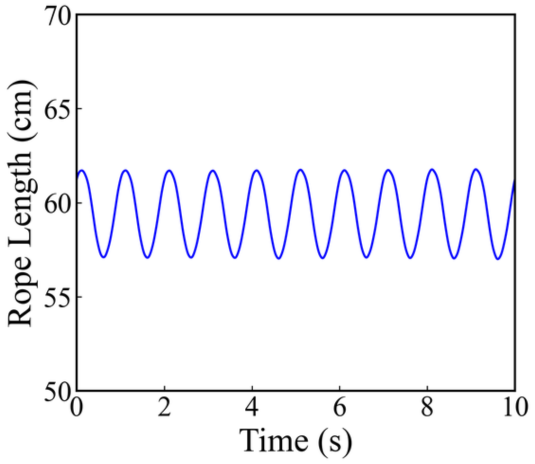

Rope Velocity

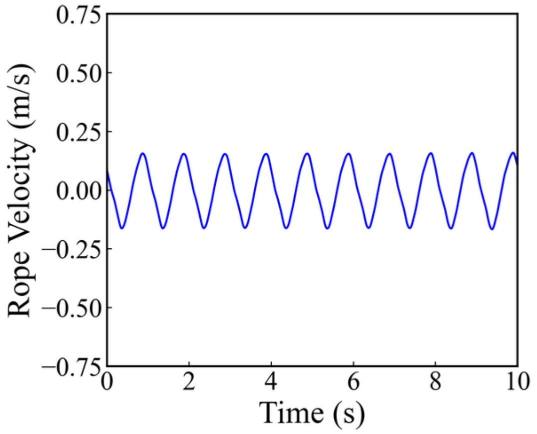

Regular wave: Period1.0s_Height7cm

Trajectory

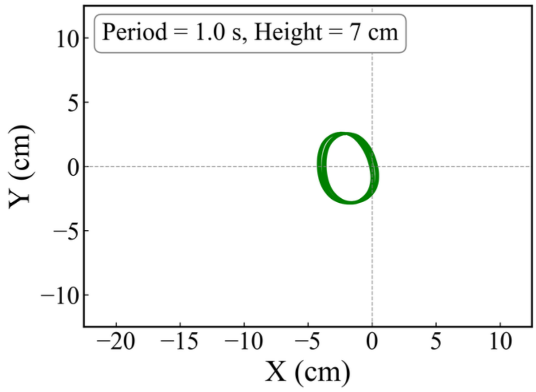

Displacement

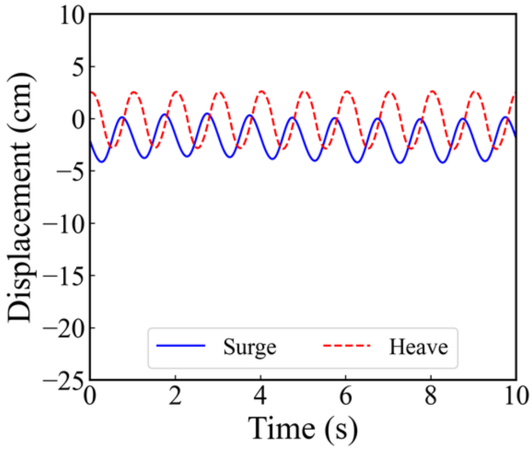

Velocity

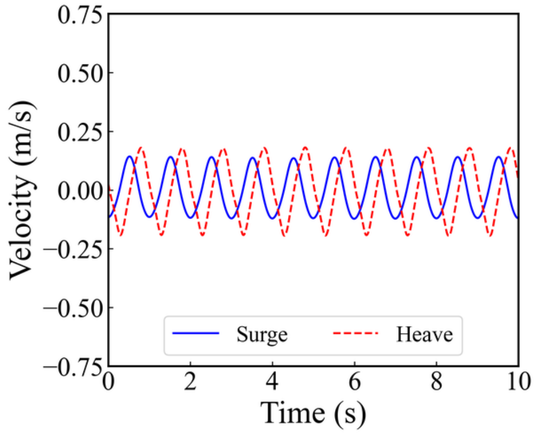

Rope Length

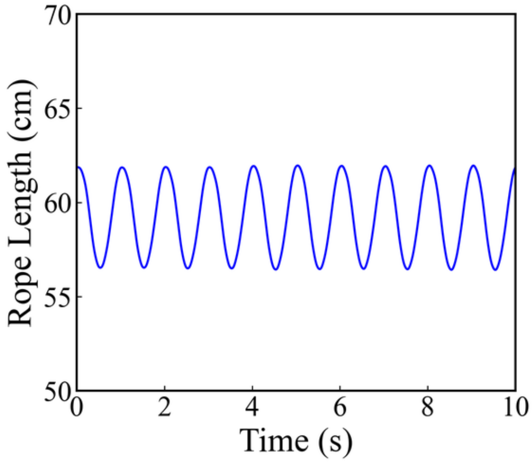

Rope Velocity

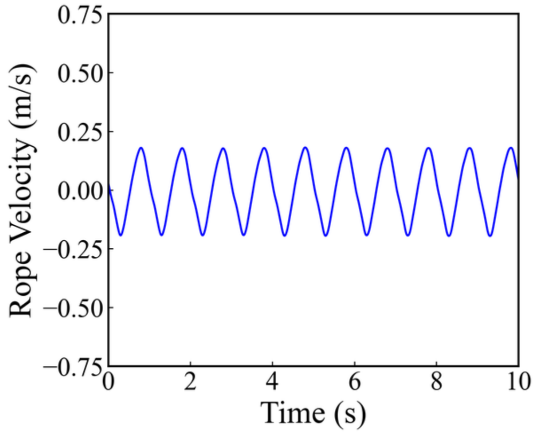

Regular wave: Period1.0s_Height8cm

Trajectory

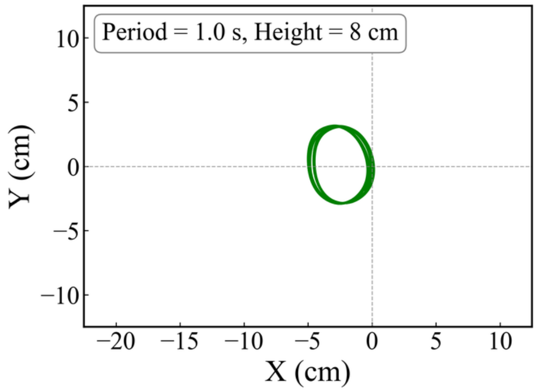

Displacement

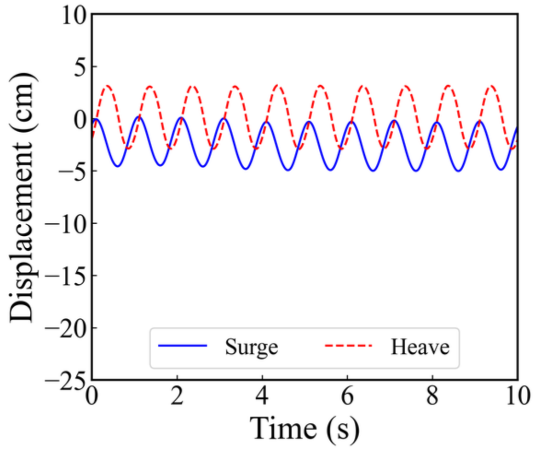

Velocity

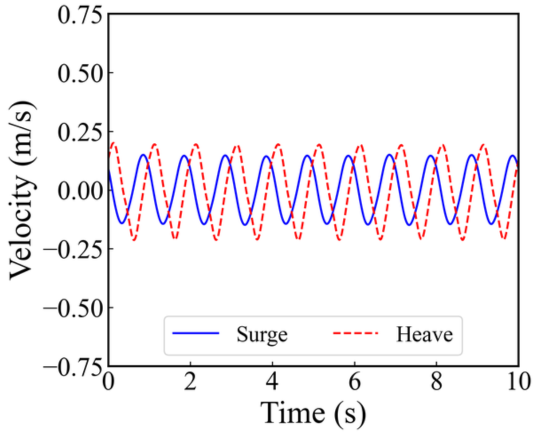

Rope Length

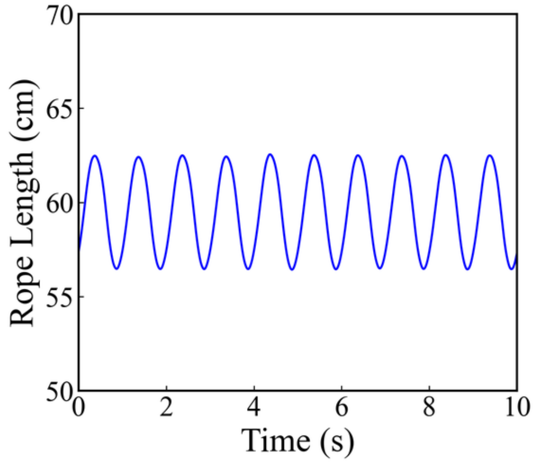

Rope Velocity

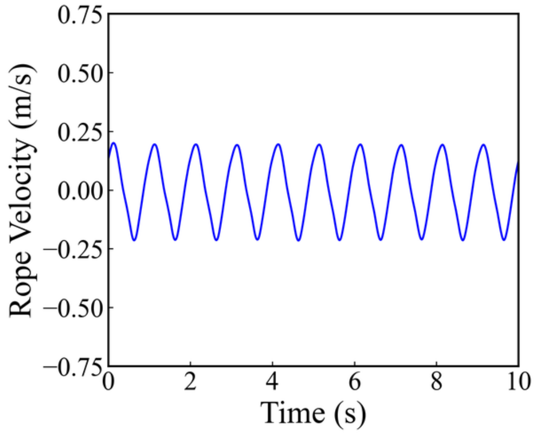

Regular wave: Period1.0s_Height9cm

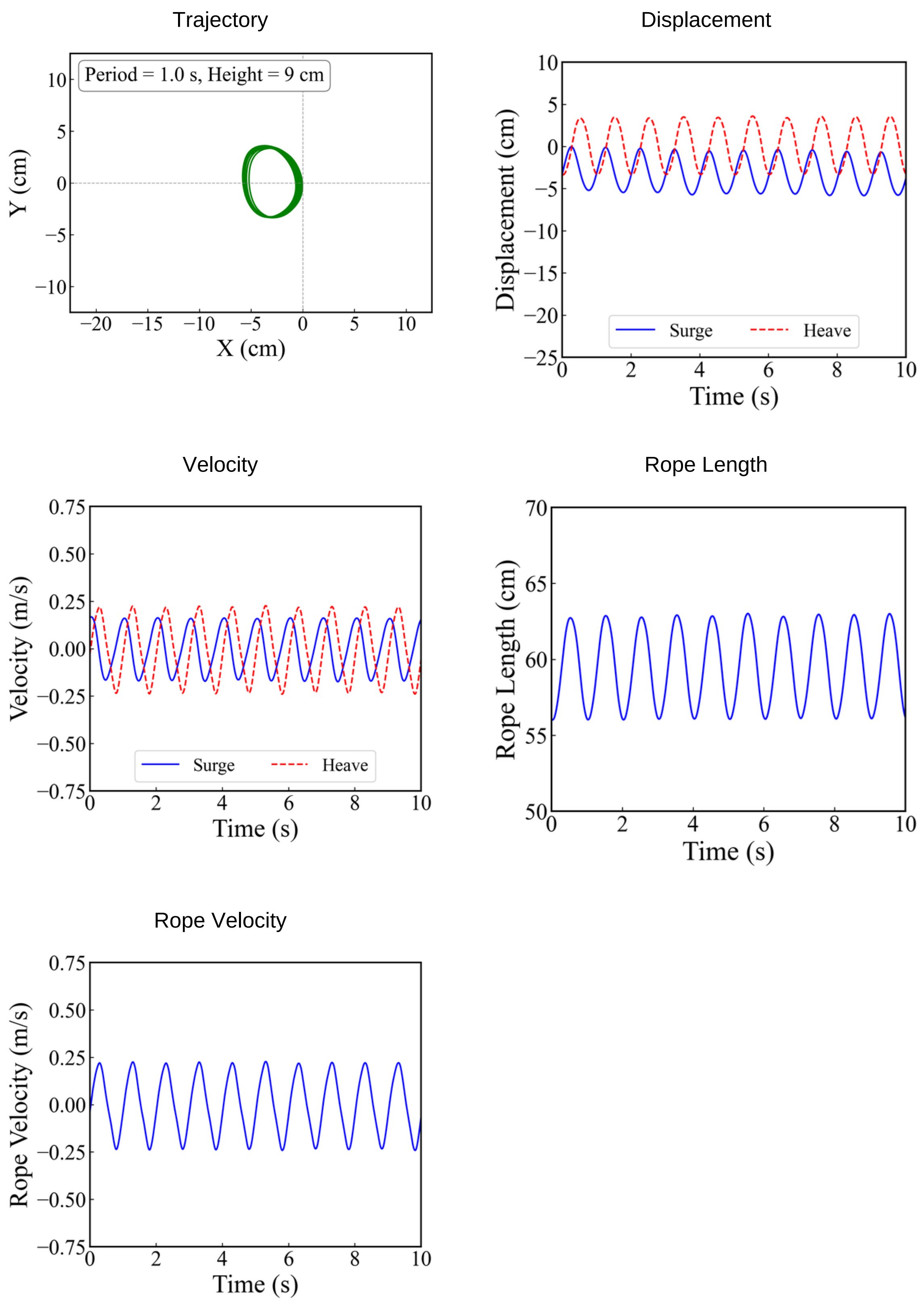

Regular wave: Period1.0s_Height10cm

Trajectory

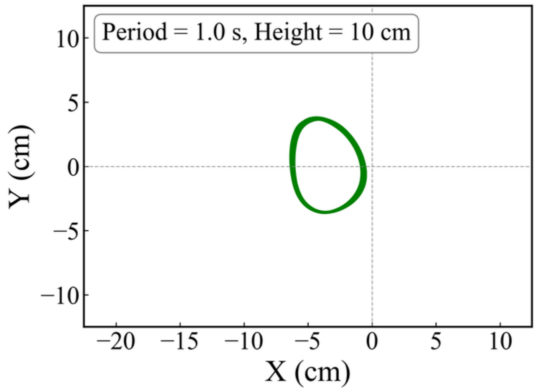

Displacement

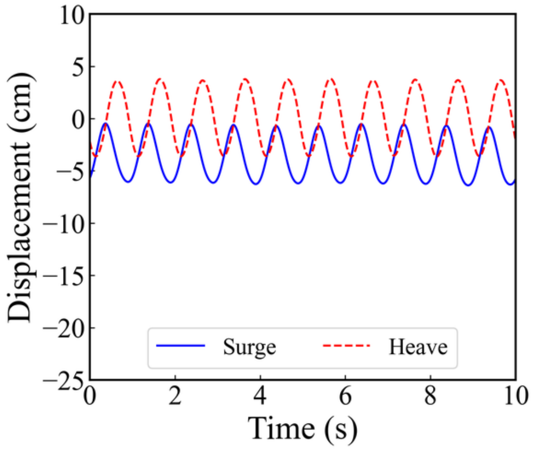

Velocity

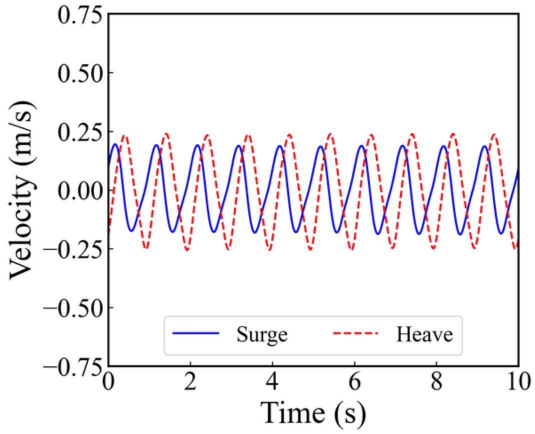

Rope Length

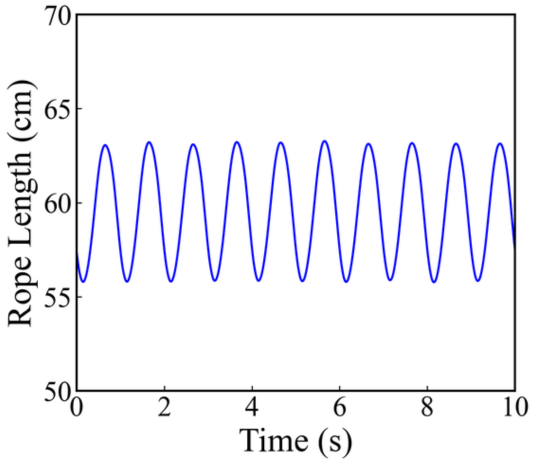

Rope Velocity

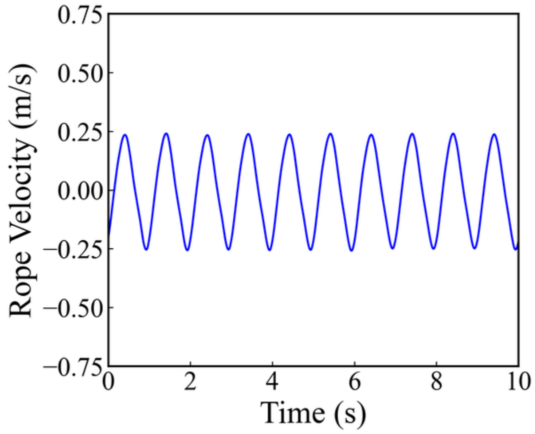

Regular wave: Period1.0s_Height11cm

Trajectory

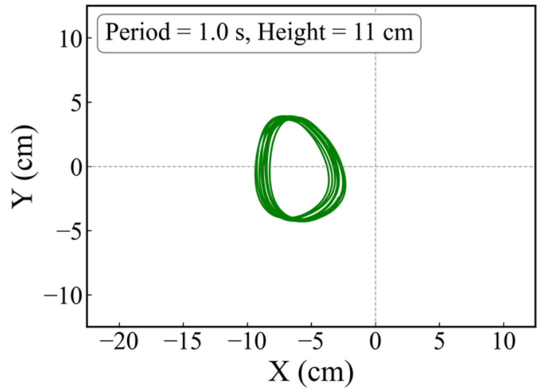

Displacement

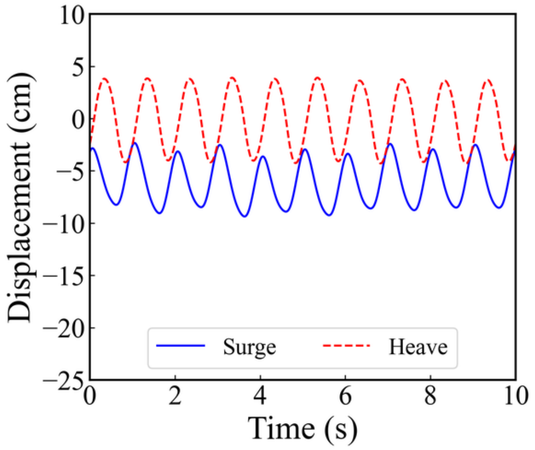

Velocity

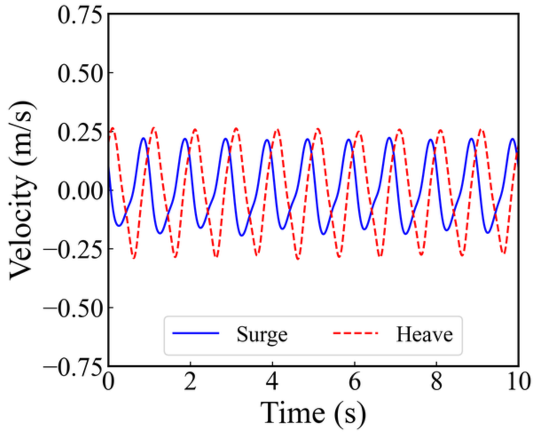

Rope Length

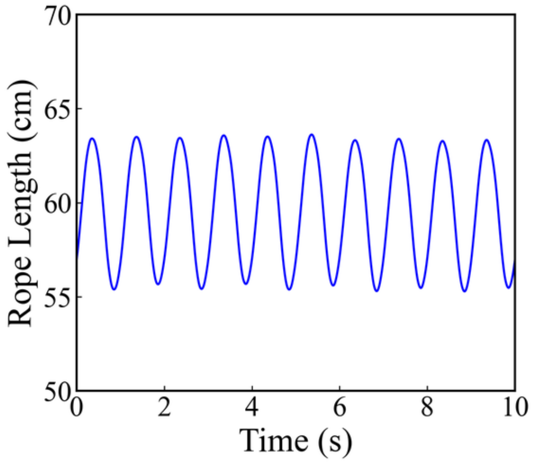

Rope Velocity

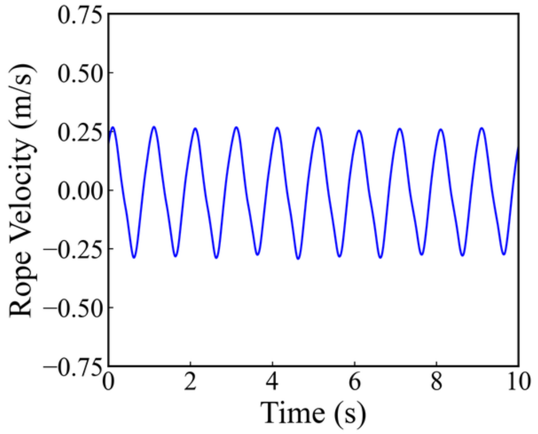

Regular wave: Period1.0s_Height12cm

Trajectory

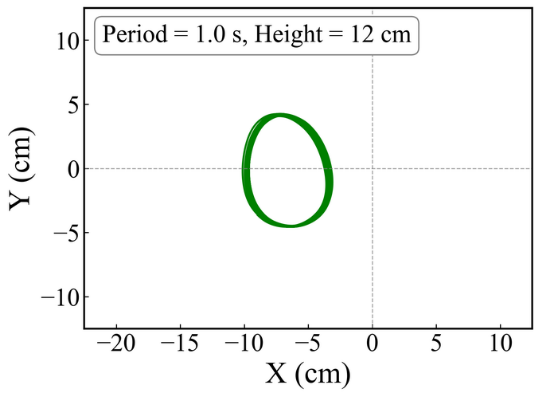

Displacement

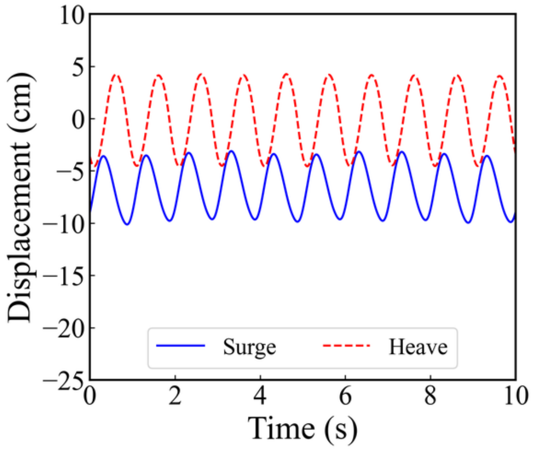

Velocity

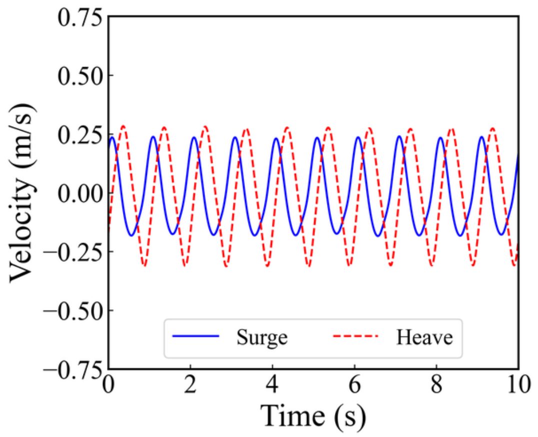

Rope Length

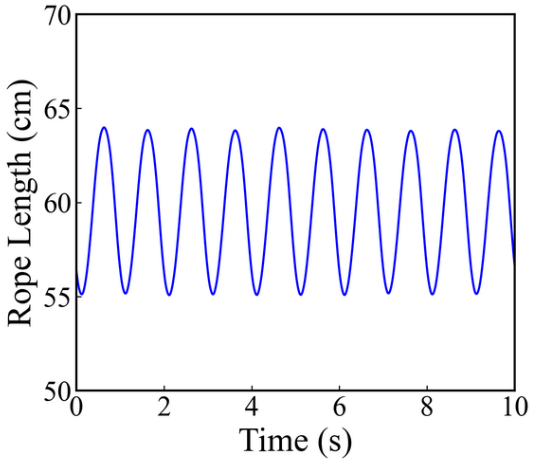

Rope Velocity

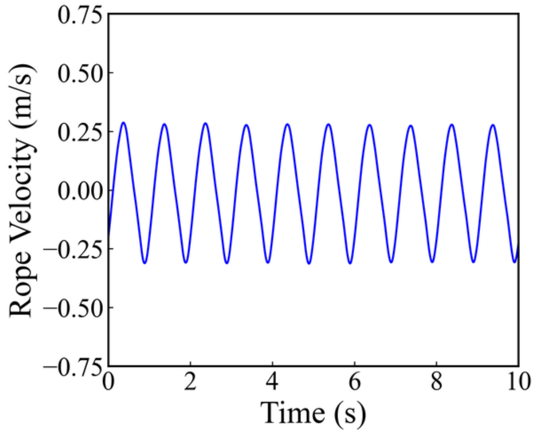

Regular wave: Period1.0s_Height13cm

Trajectory

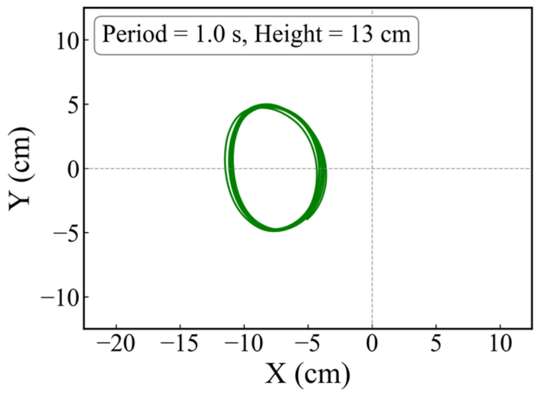

Displacement

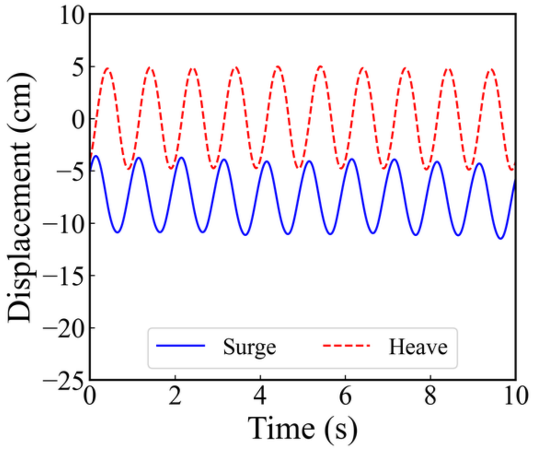

Velocity

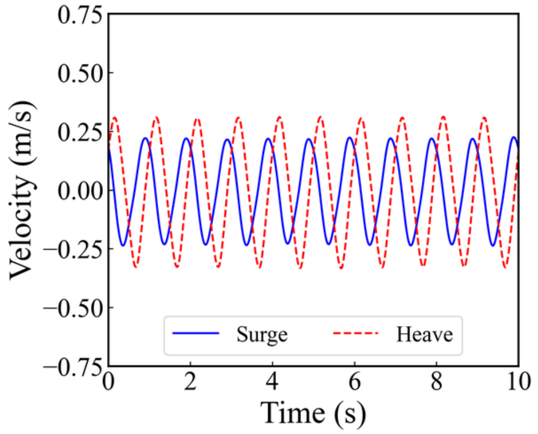

Rope Length

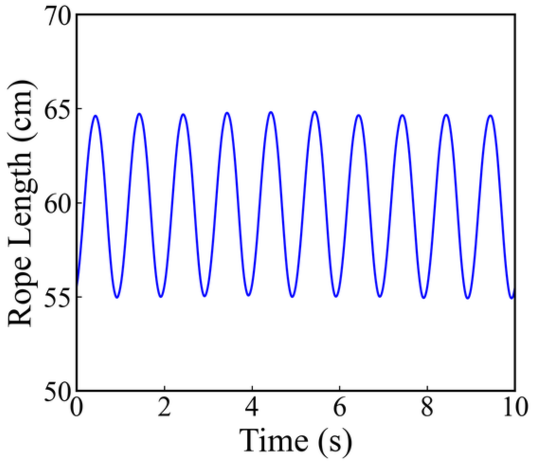

Rope Velocity

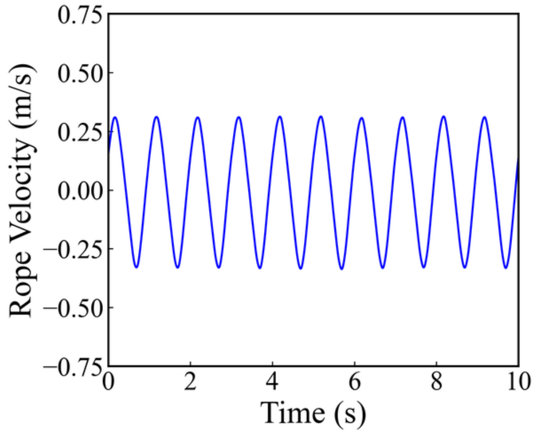

Regular wave: Period1.0s_Height14cm

Trajectory

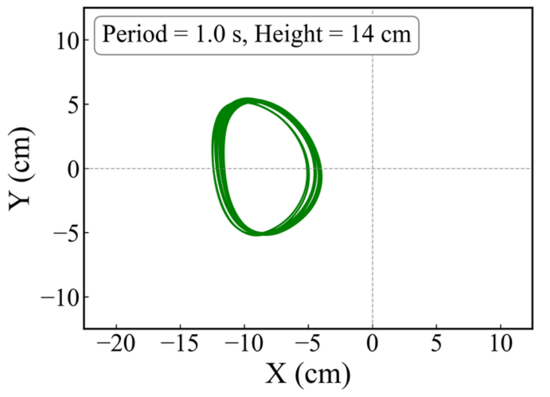

Displacement

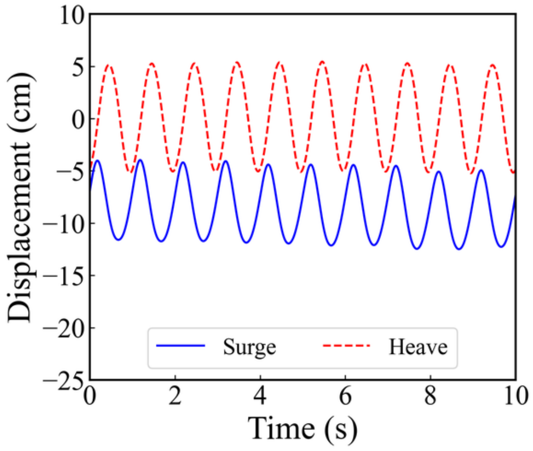

Velocity

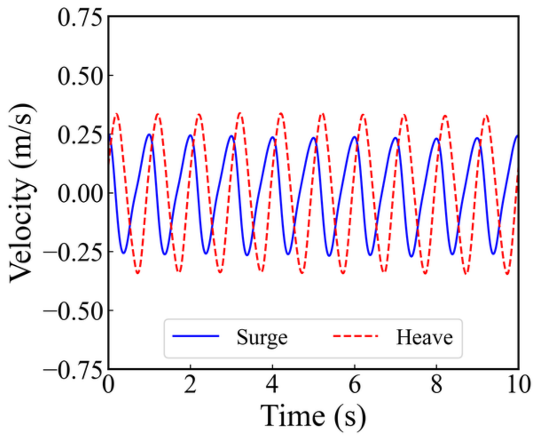

Rope Length

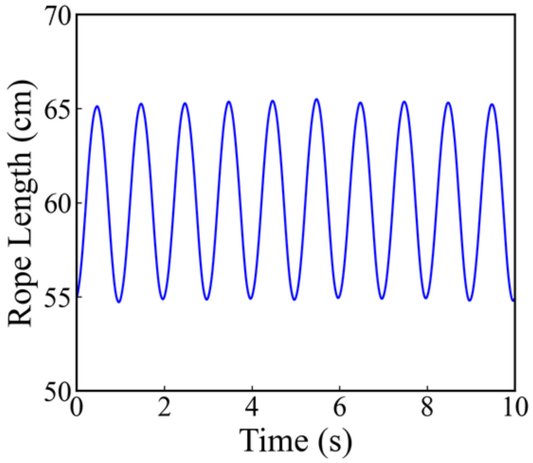

Rope Velocity

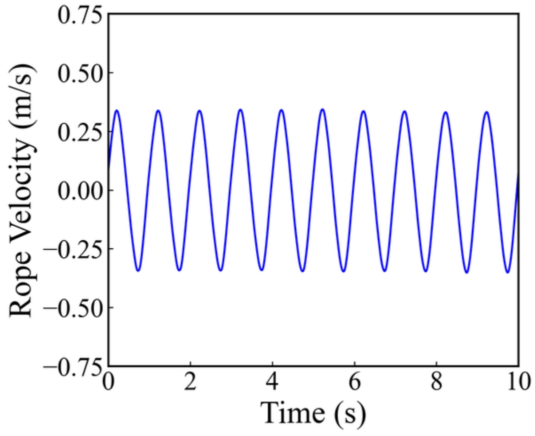

Regular wave: Period1.0s_Height15cm

Trajectory

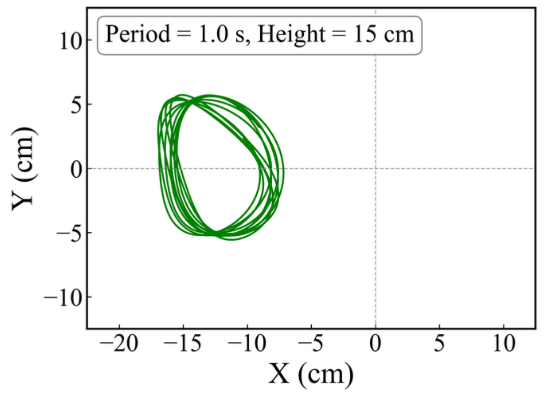

Displacement

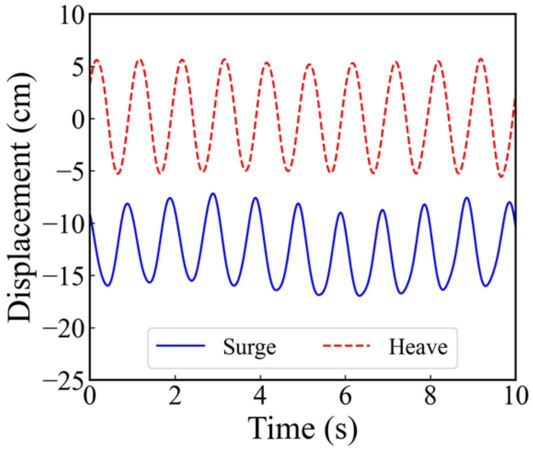

Velocity

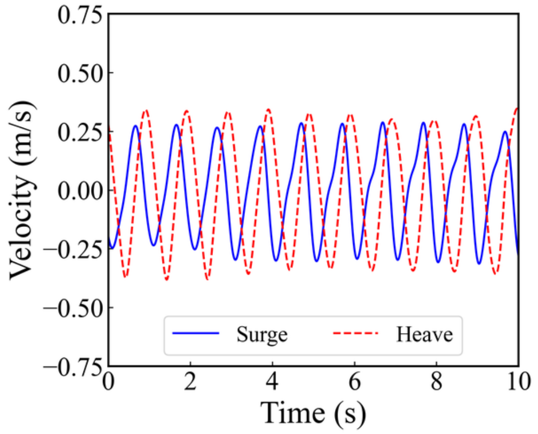

Rope Length

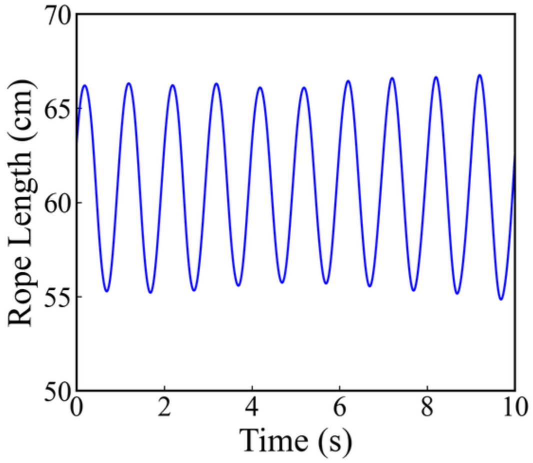

Rope Velocity

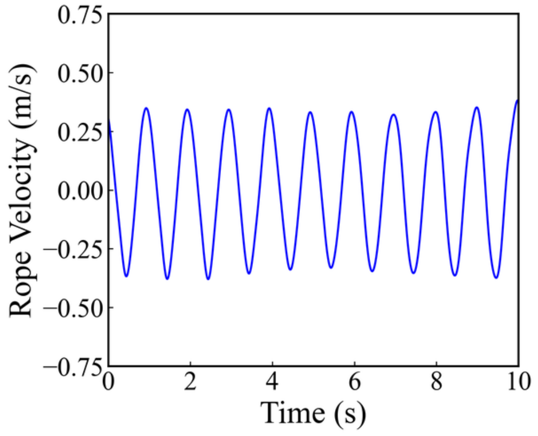

Regular wave: Period1.1s_Height5cm

Trajectory

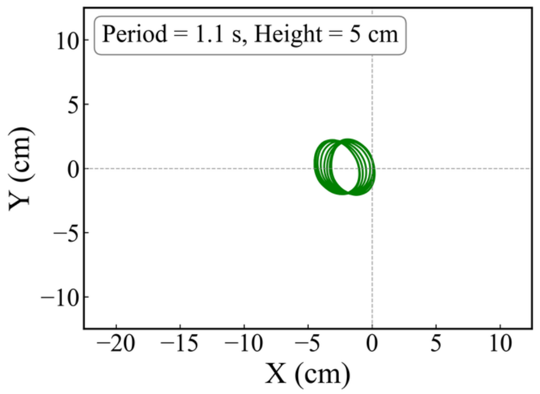

Displacement

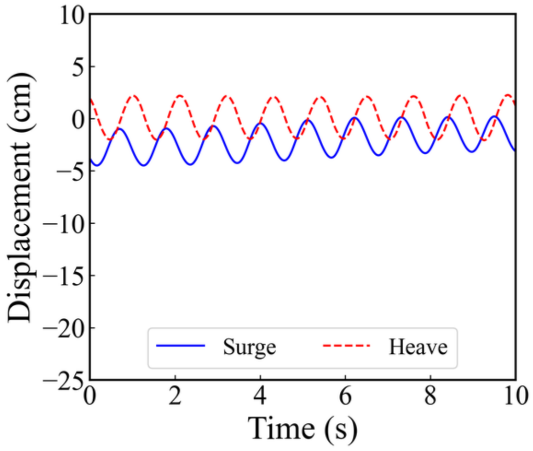

Velocity

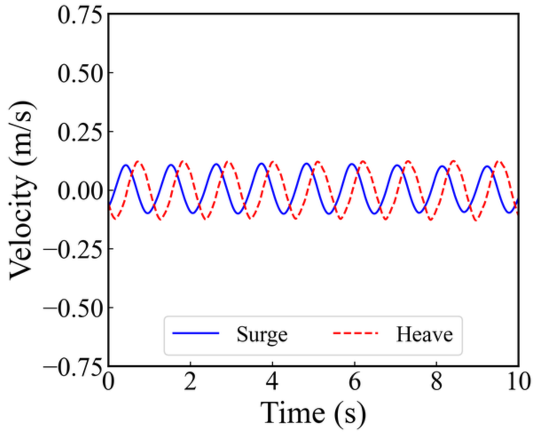

Rope Length

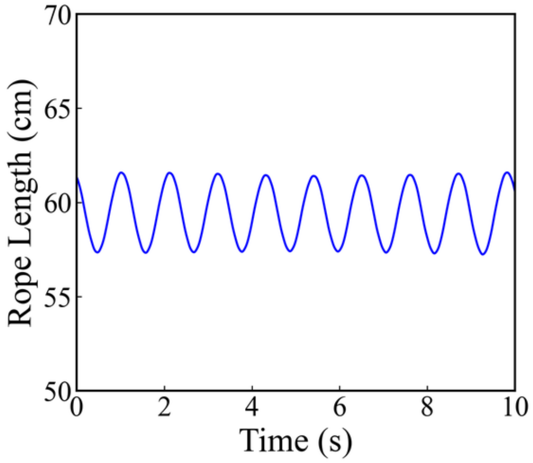

Rope Velocity

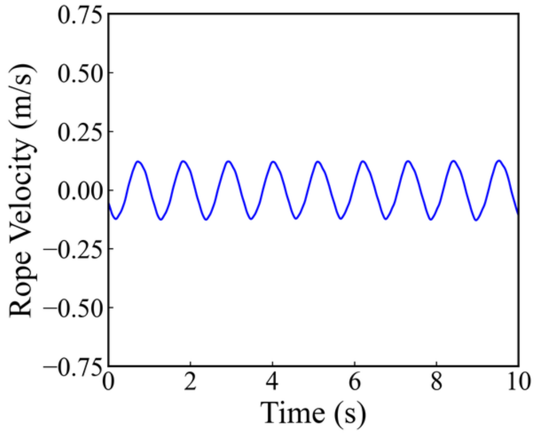

Regular wave: Period1.1s_Height6cm

Trajectory

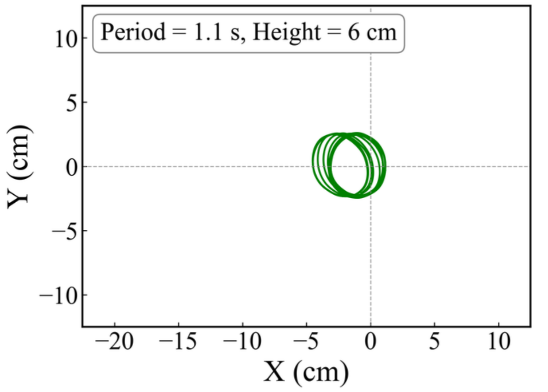

Displacement

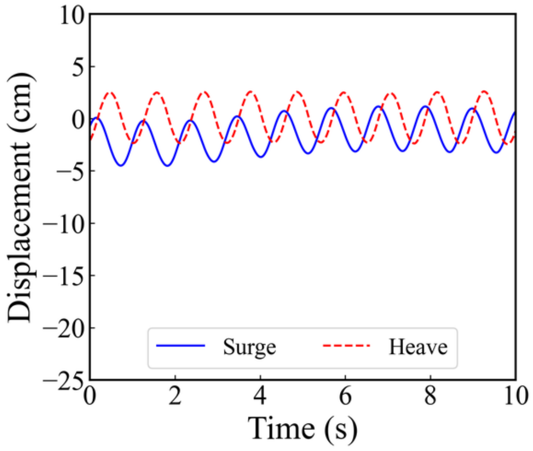

Velocity

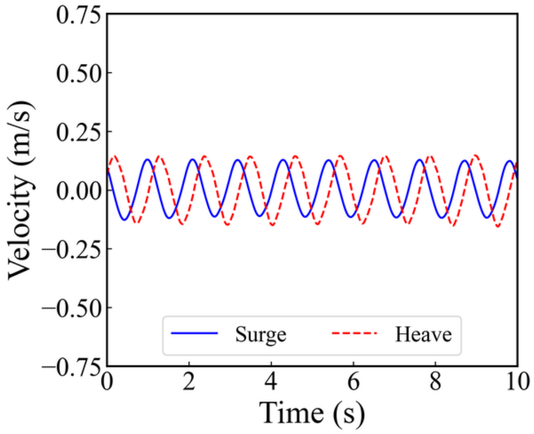

Rope Length

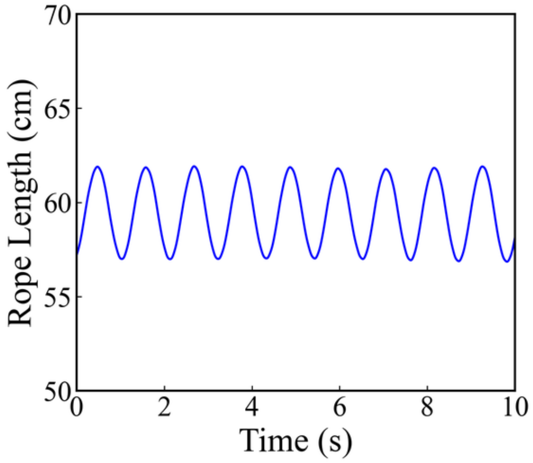

Rope Velocity

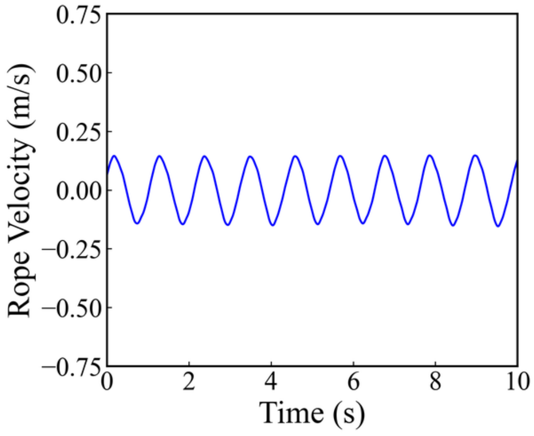

Regular wave: Period1.1s_Height7cm

Trajectory

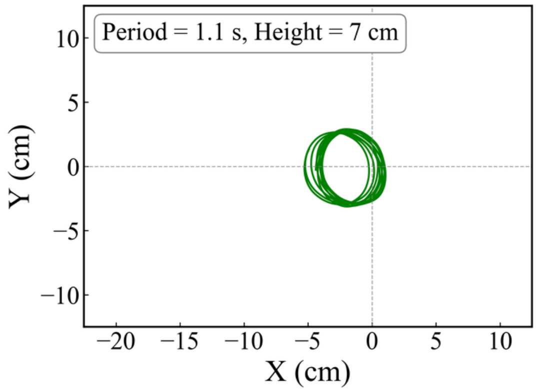

Displacement

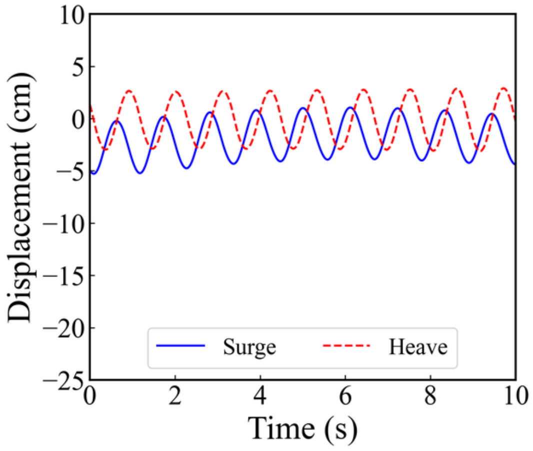

Velocity

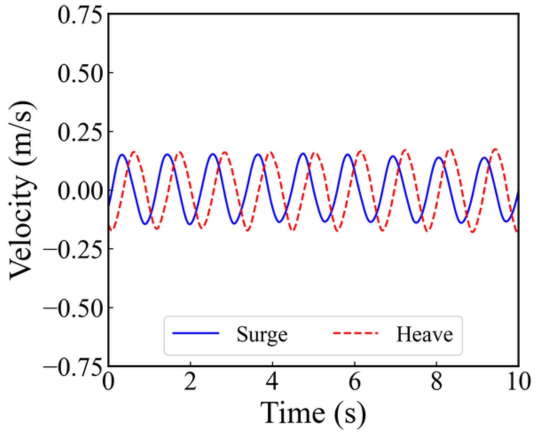

Rope Length

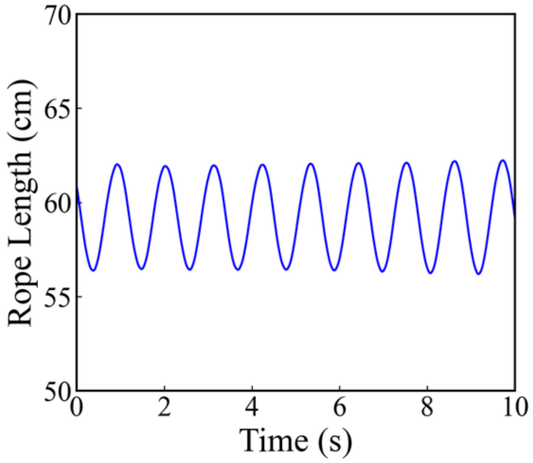

Rope Velocity

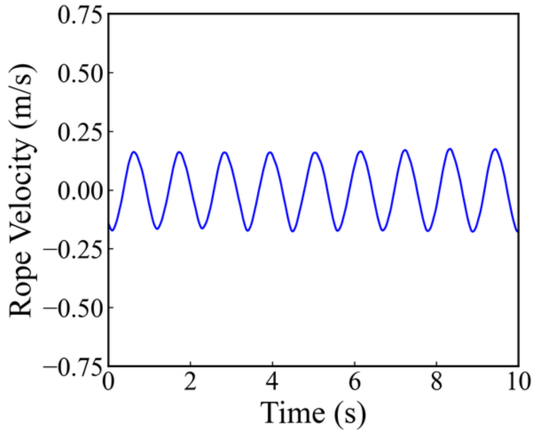

Regular wave: Period1.1s_Height8cm

Trajectory

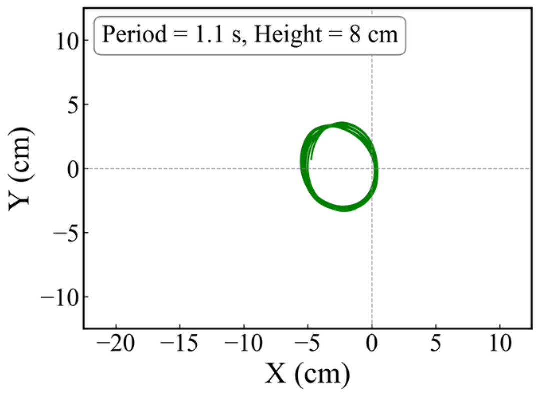

Displacement

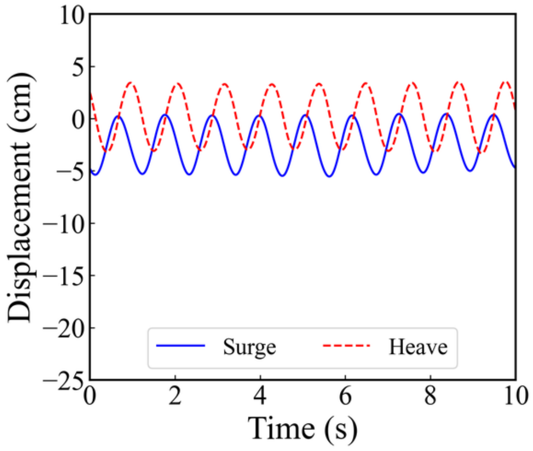

Velocity

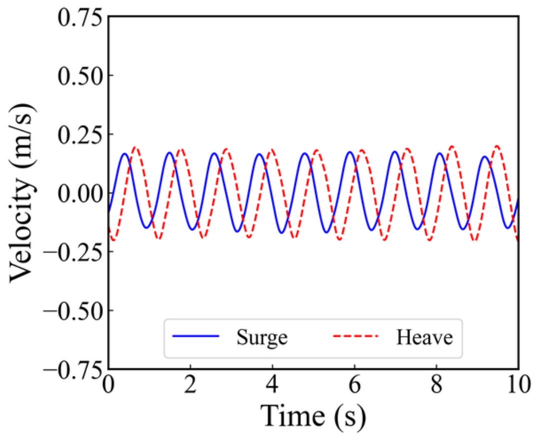

Rope Length

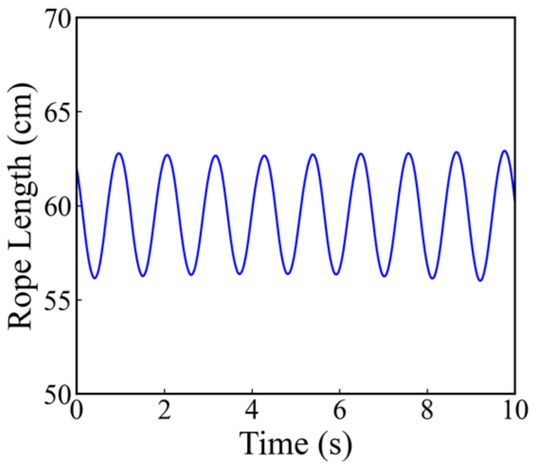

Rope Velocity

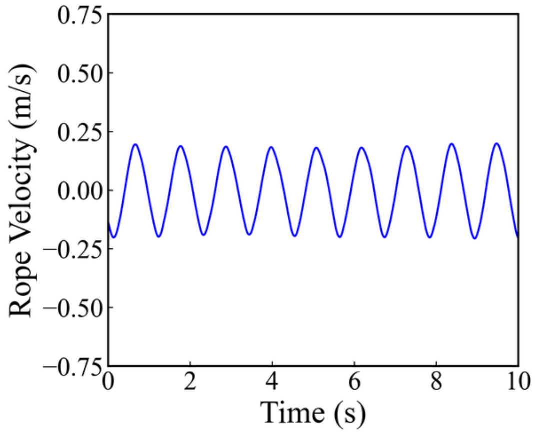

Regular wave: Period1.1s_Height9cm

Trajectory

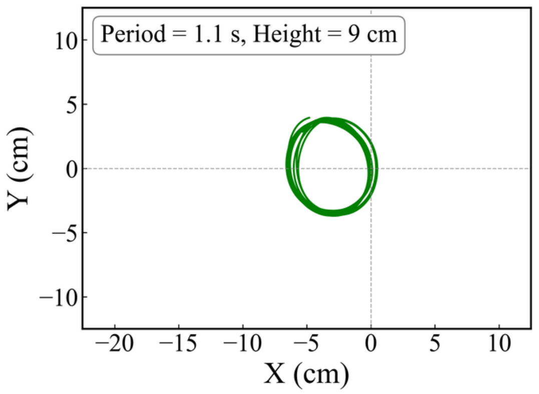

Displacement

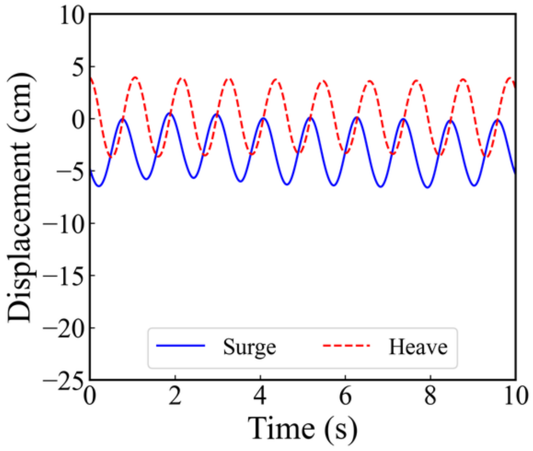

Velocity

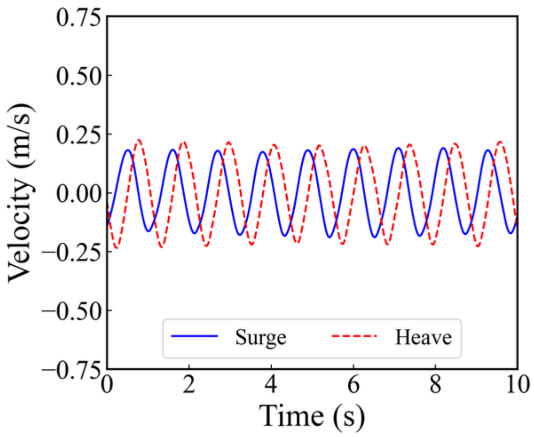

Rope Length

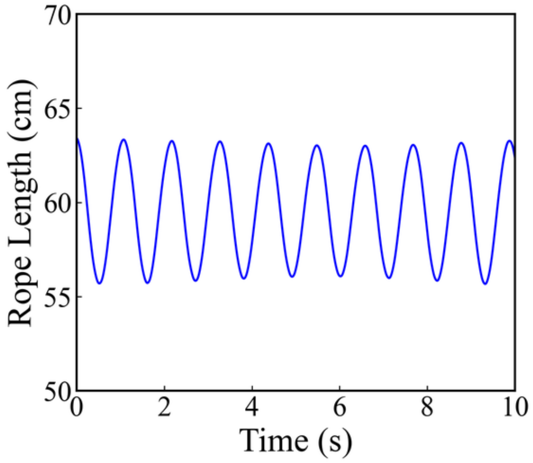

Rope Velocity

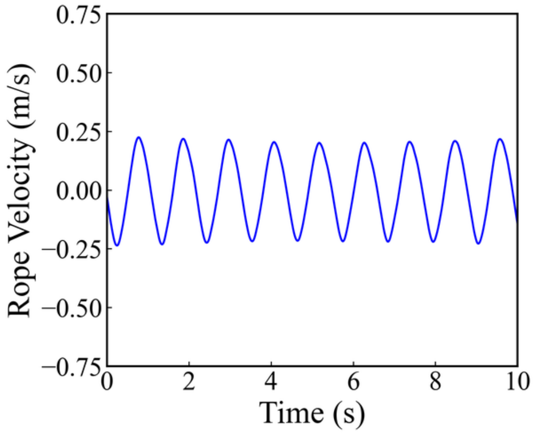

Regular wave: Period1.1s_Height10cm

Trajectory

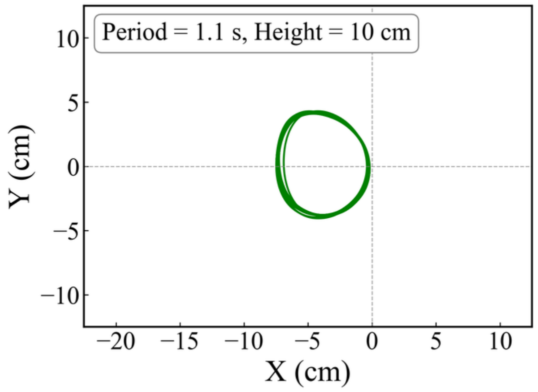

Displacement

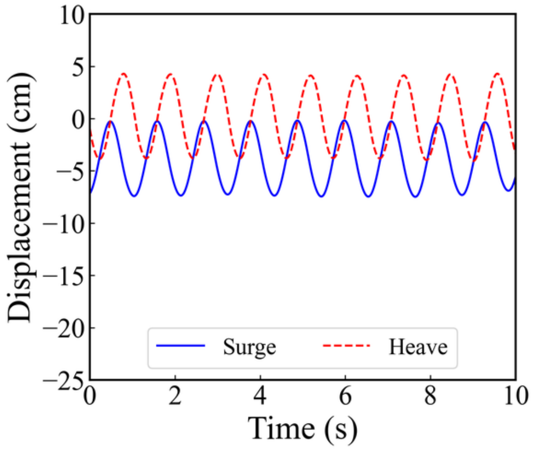

Velocity

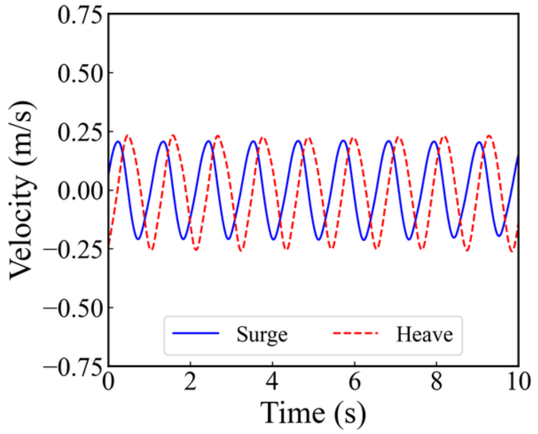

Rope Length

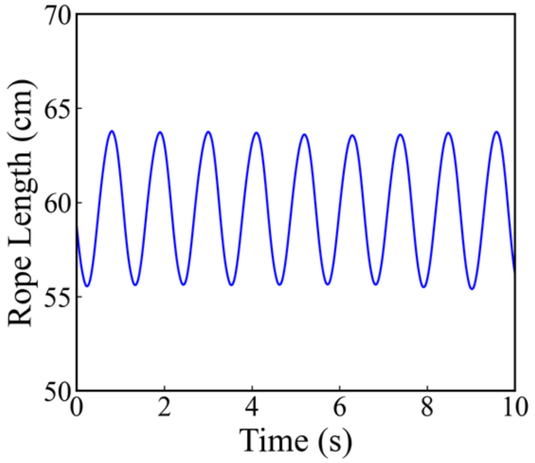

Rope Velocity

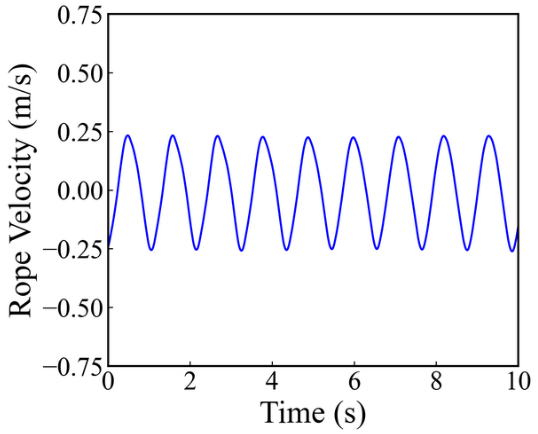

Regular wave: Period1.1s_Height11cm

Trajectory

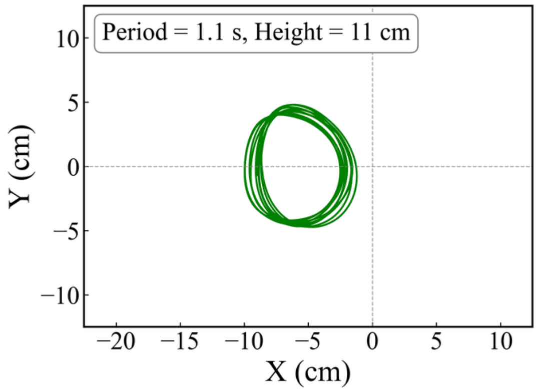

Displacement

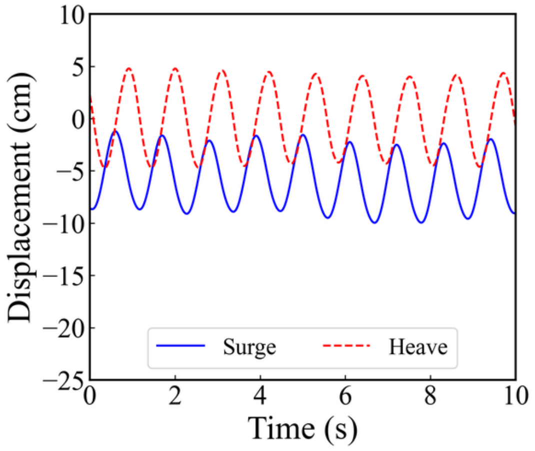

Velocity

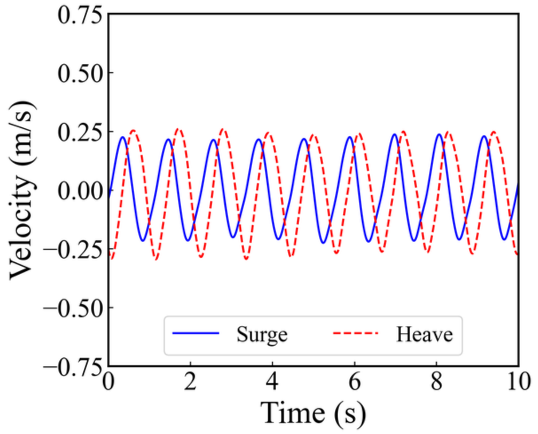

Rope Length

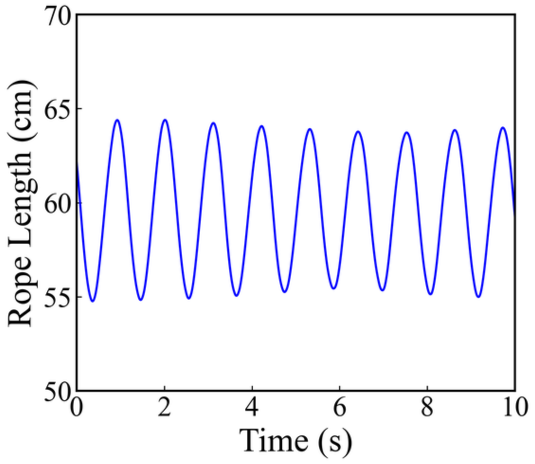

Rope Velocity

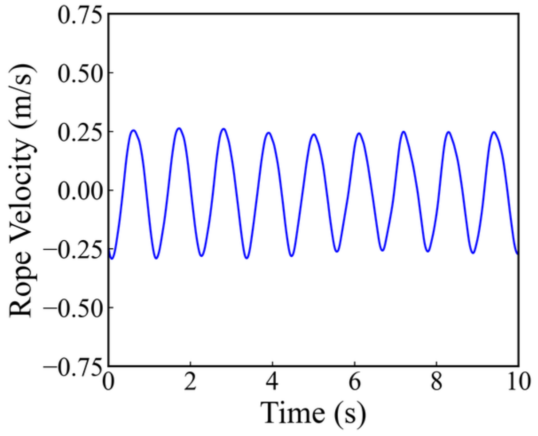

Regular wave: Period1.1s_Height12cm

Trajectory

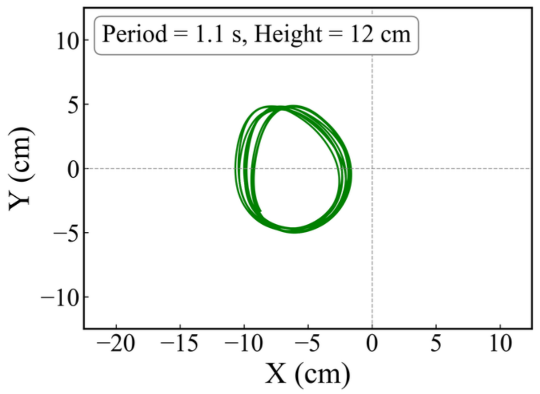

Displacement

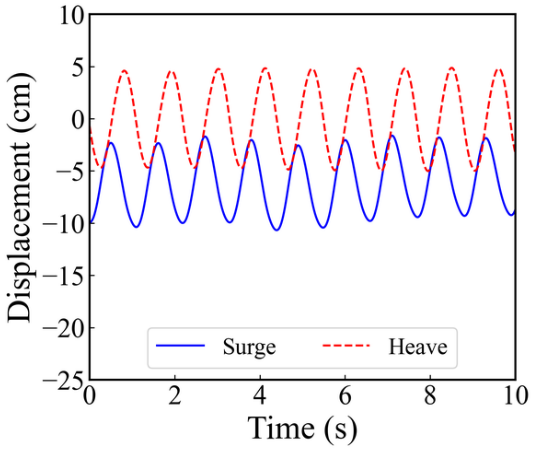

Velocity

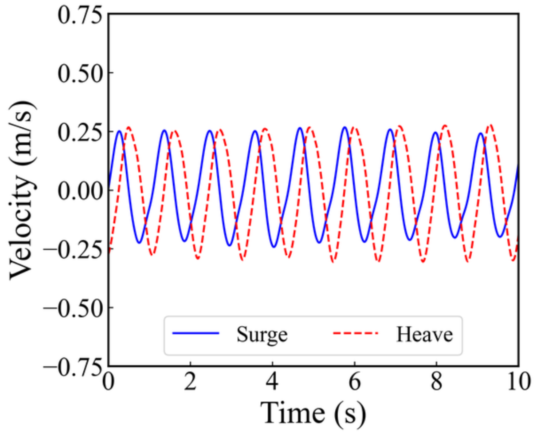

Rope Length

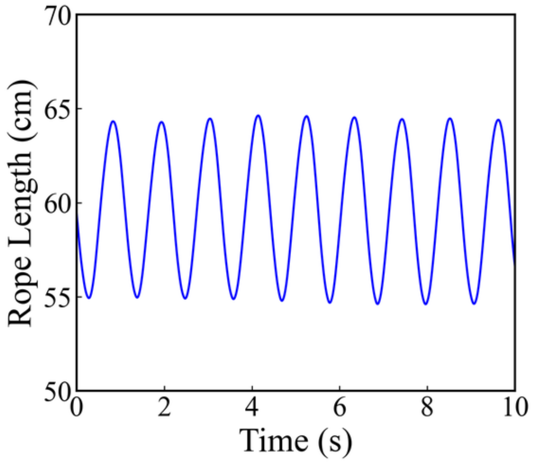

Rope Velocity

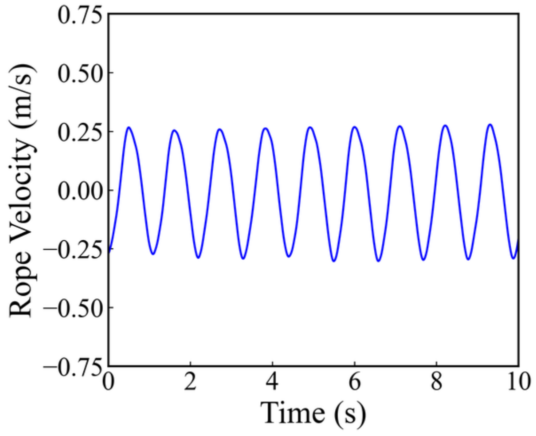

Regular wave: Period1.1s_Height13cm

Trajectory

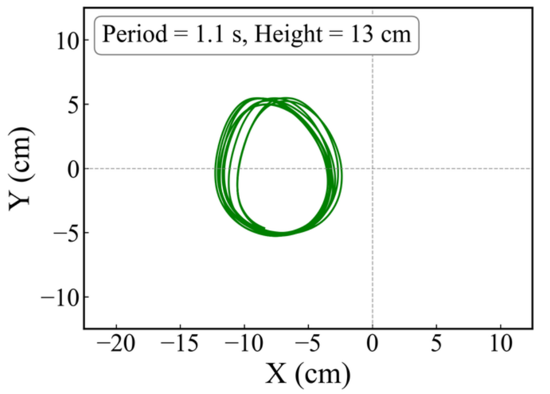

Displacement

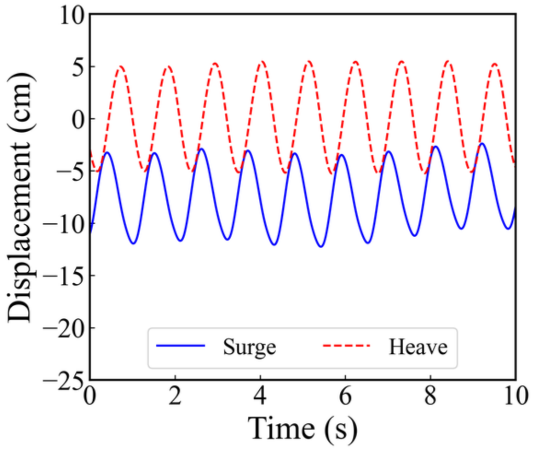

Velocity

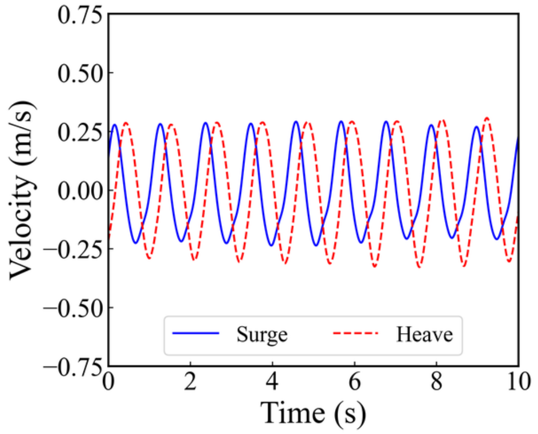

Rope Length

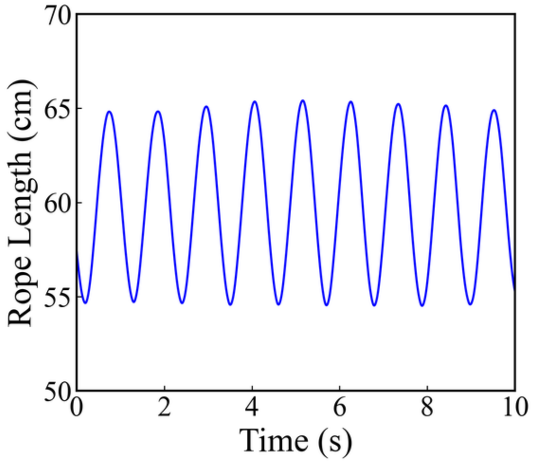

Rope Velocity

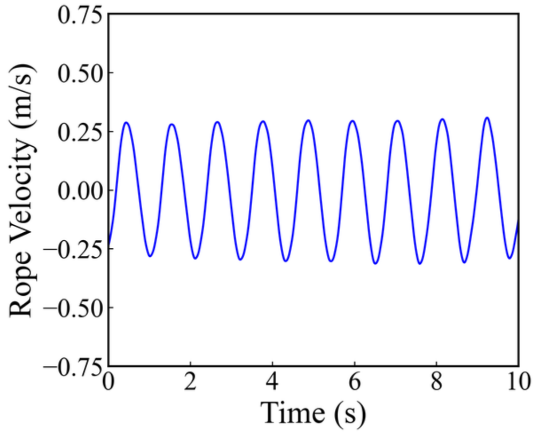

Regular wave: Period1.1s_Height14cm

Trajectory

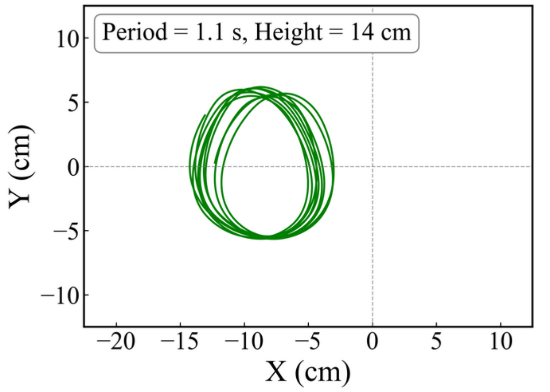

Displacement

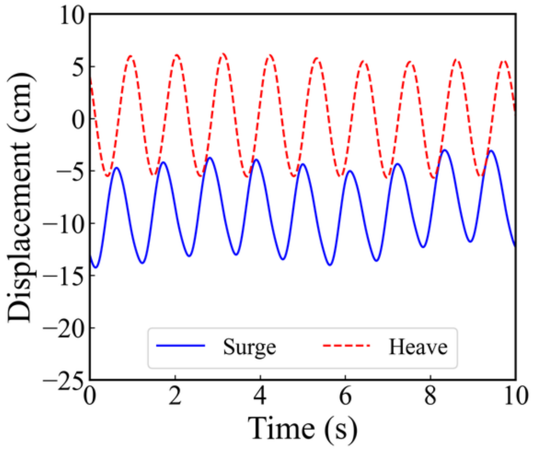

Velocity

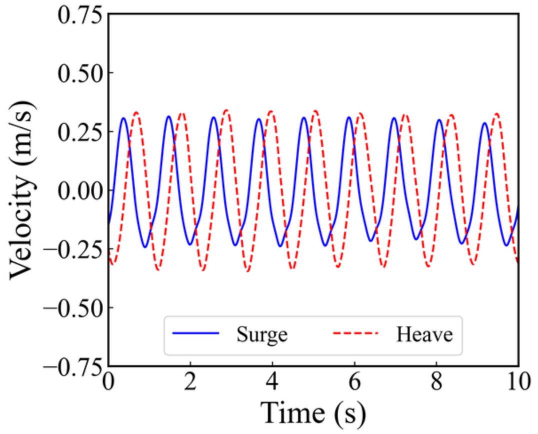

Rope Length

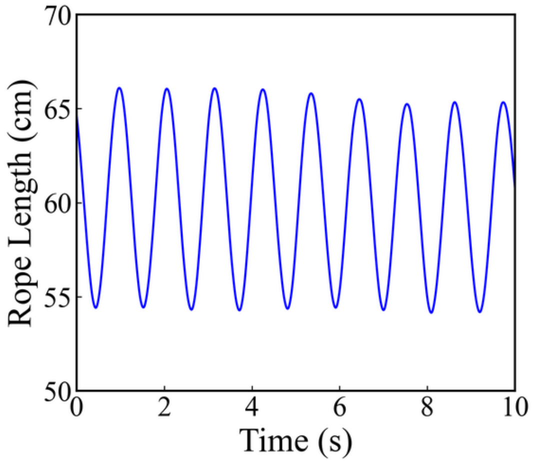

Rope Velocity

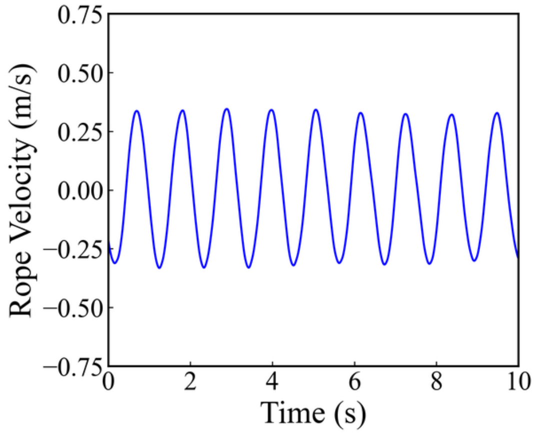

Regular wave: Period1.1s_Height15cm

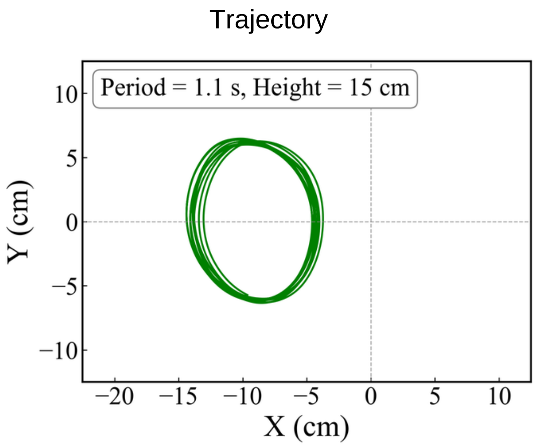

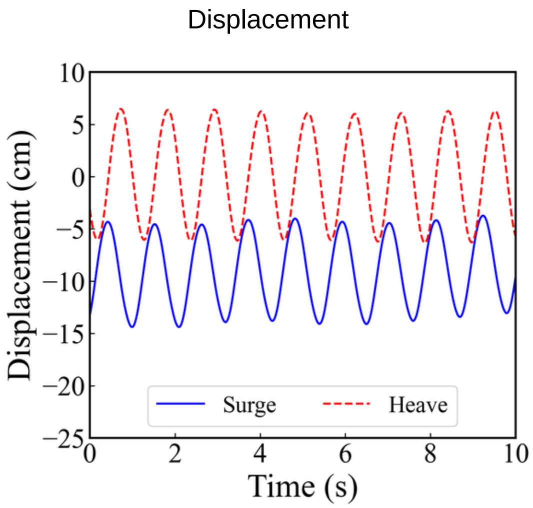

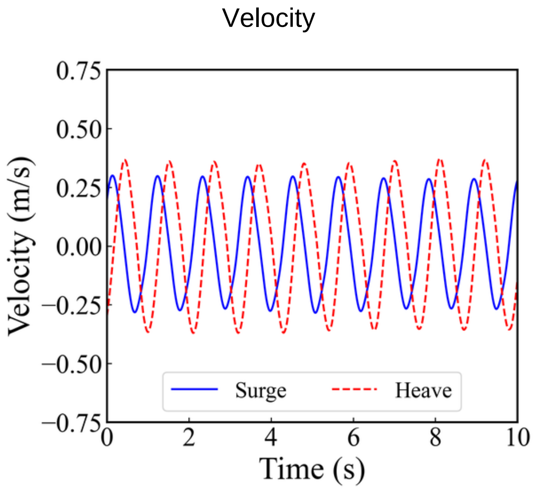

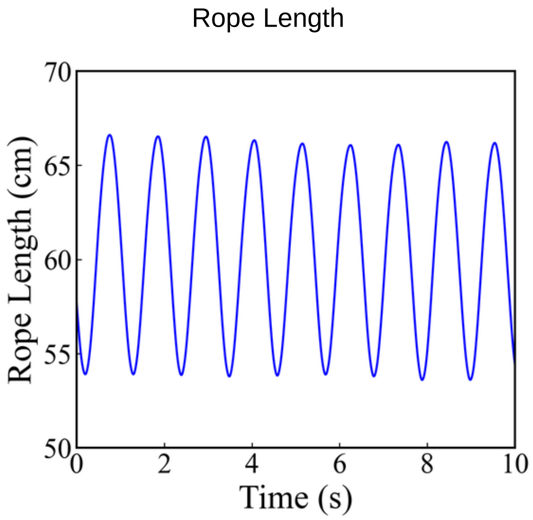

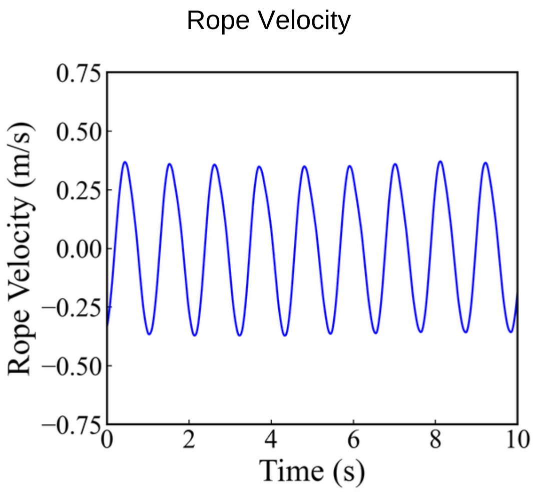

Regular wave: Period1.2s_Height5cm

Trajectory

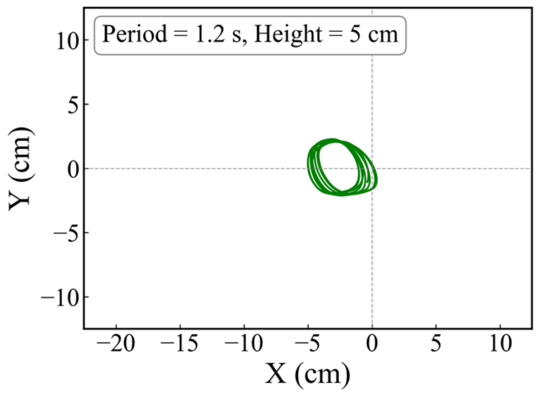

Displacement

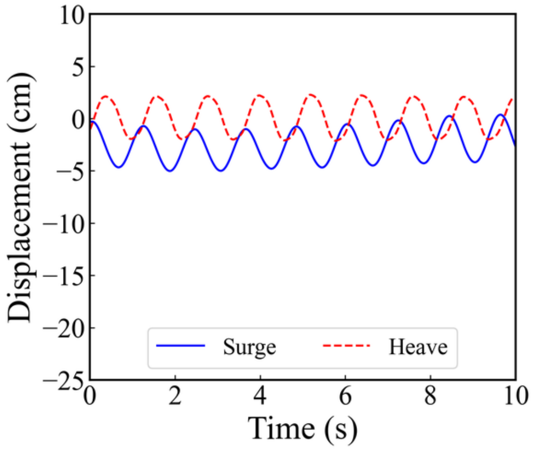

Velocity

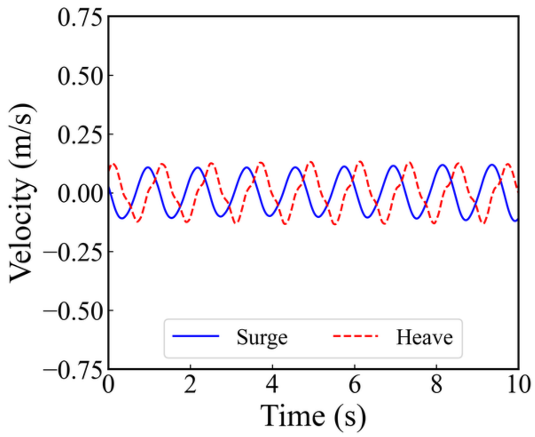

Rope Length

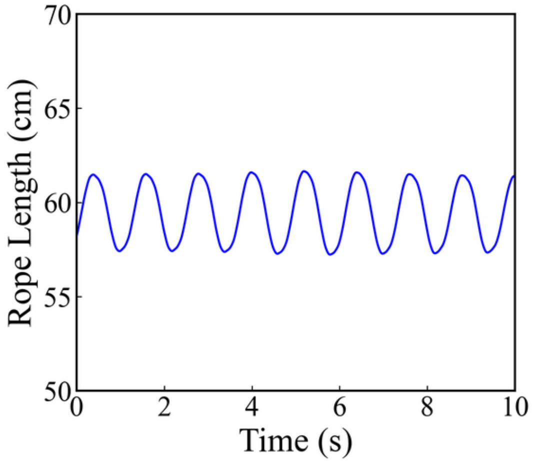

Rope Velocity

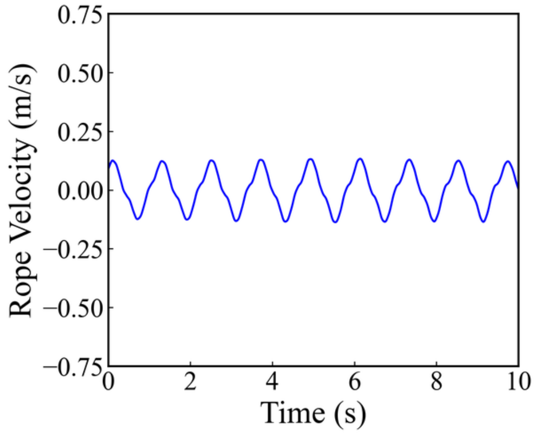

Regular wave: Period1.2s_Height6cm

Trajectory

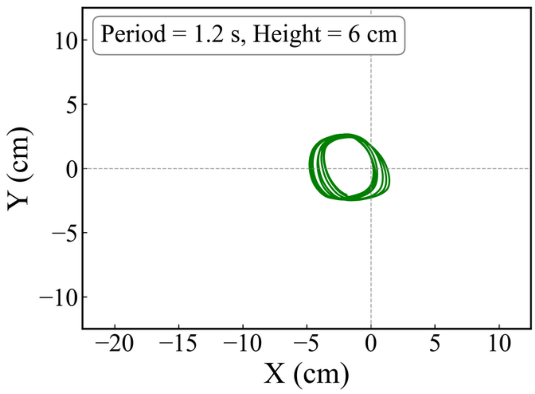

Displacement

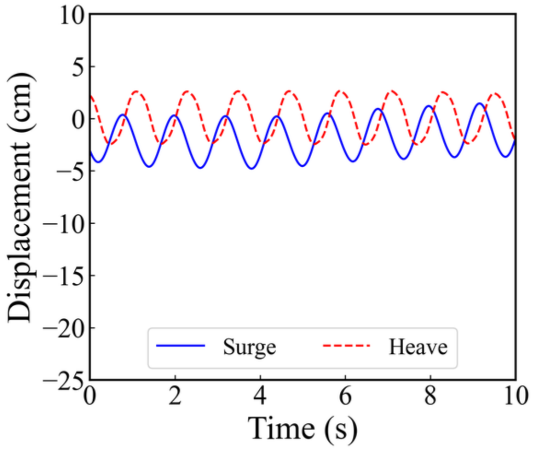

Velocity

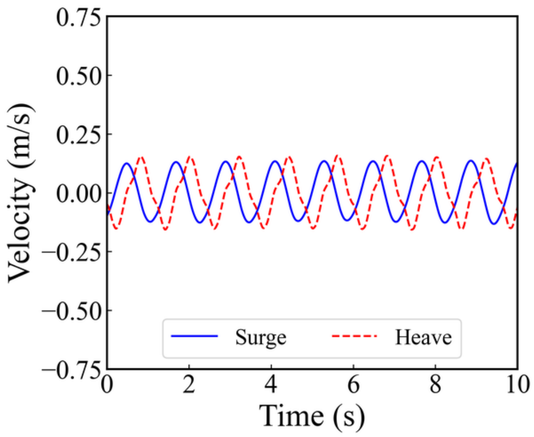

Rope Length

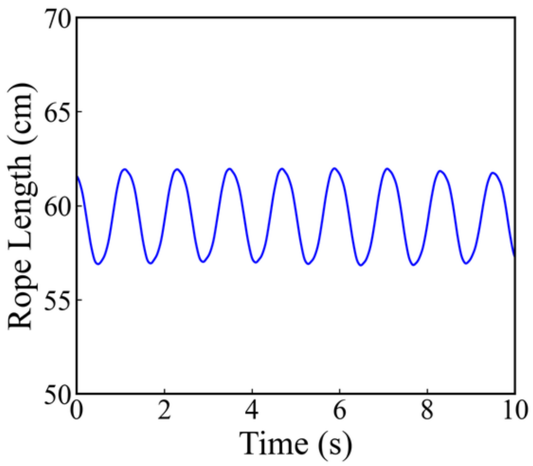

Rope Velocity

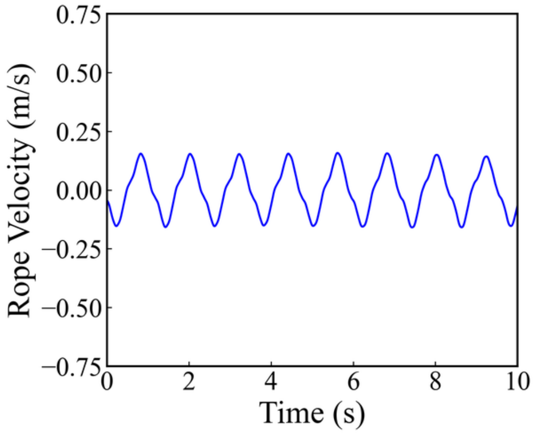

Regular wave: Period1.2s_Height7cm

Trajectory

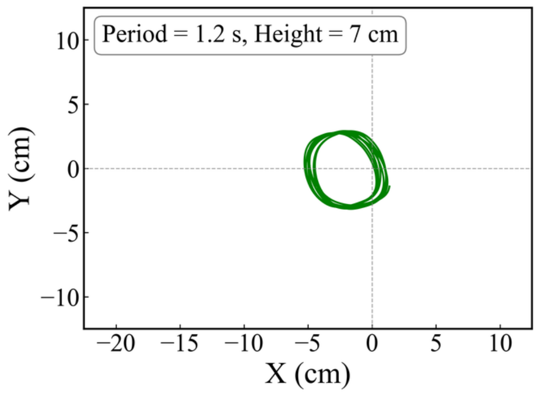

Displacement

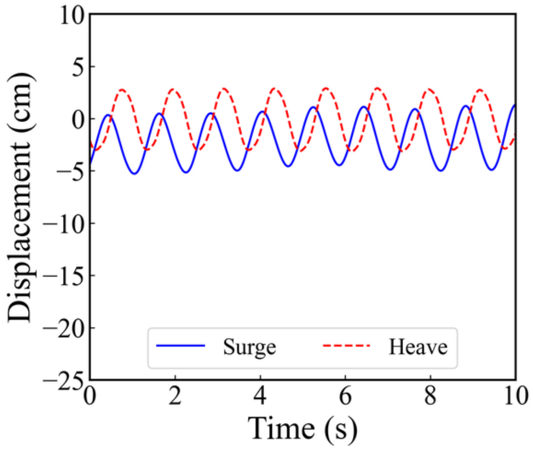

Velocity

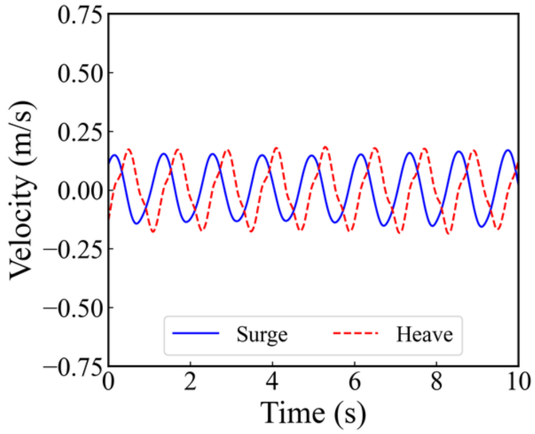

Rope Length

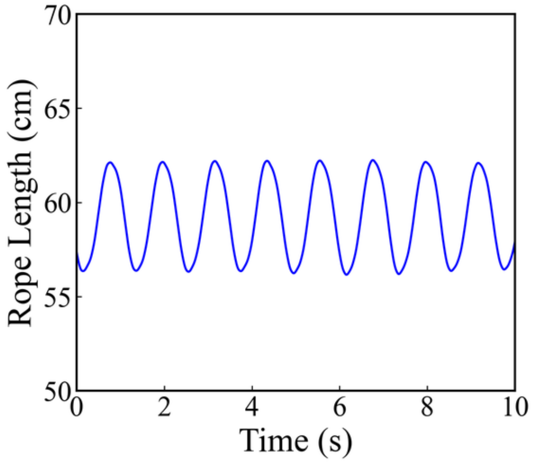

Rope Velocity

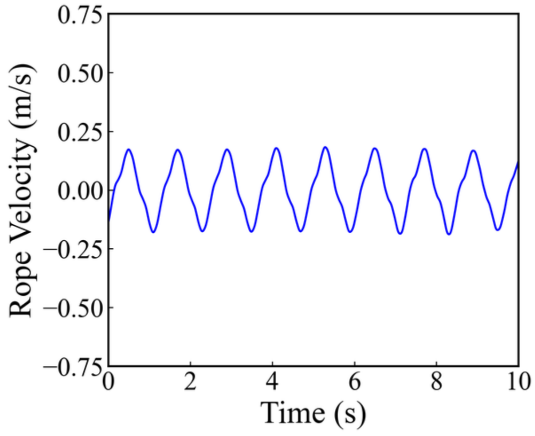

Regular wave: Period1.2s_Height8cm

Trajectory

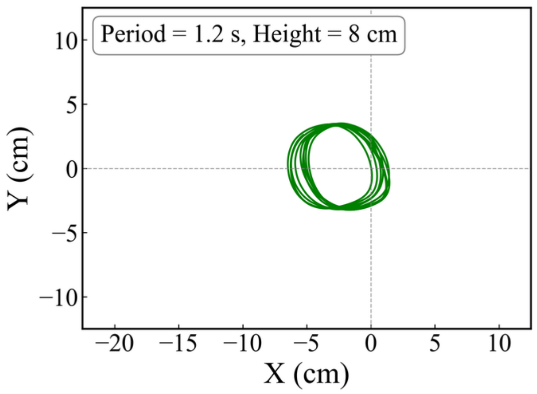

Displacement

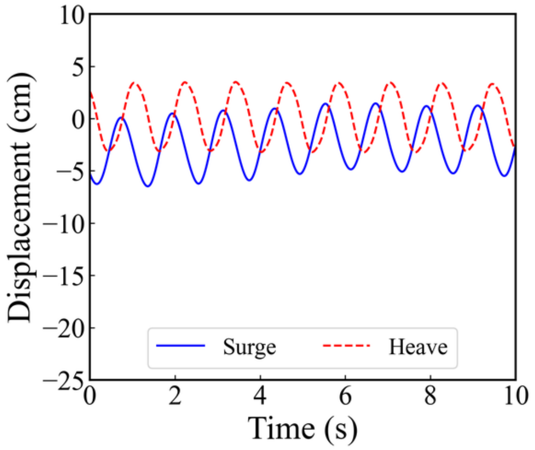

Velocity

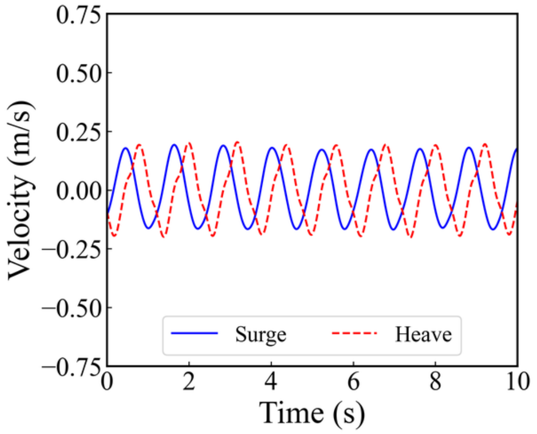

Rope Length

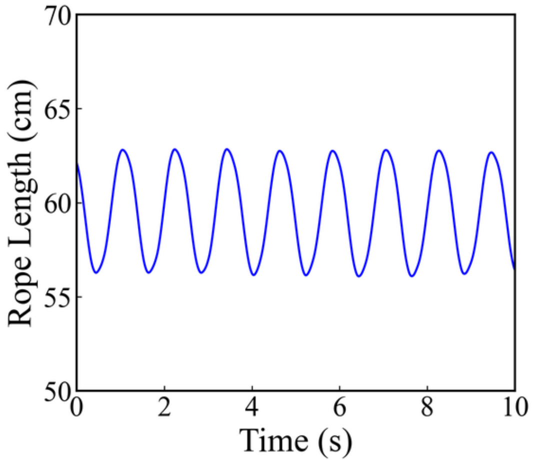

Rope Velocity

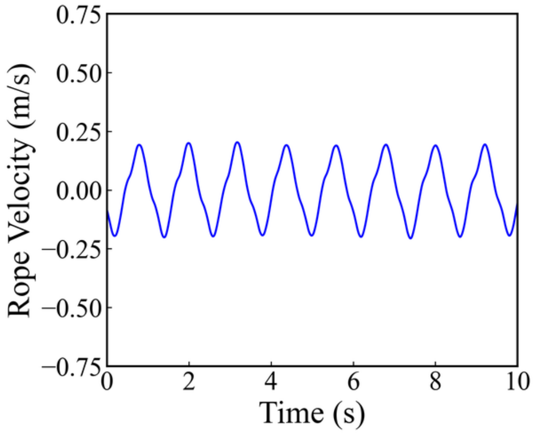

Regular wave: Period1.2s_Height9cm

Trajectory

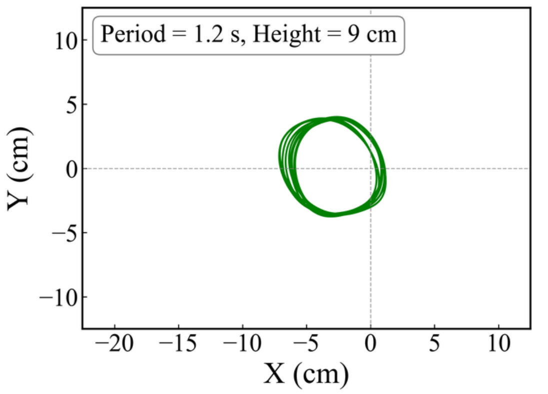

Displacement

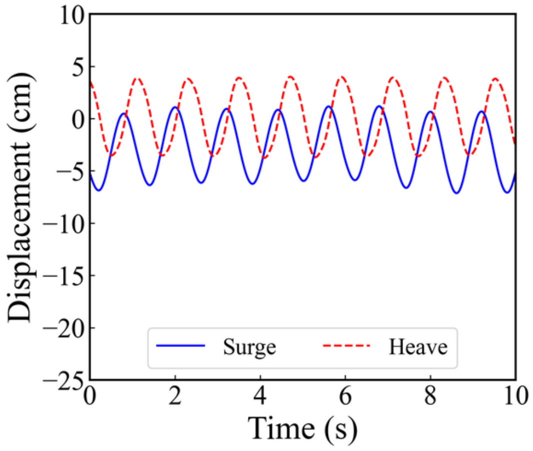

Velocity

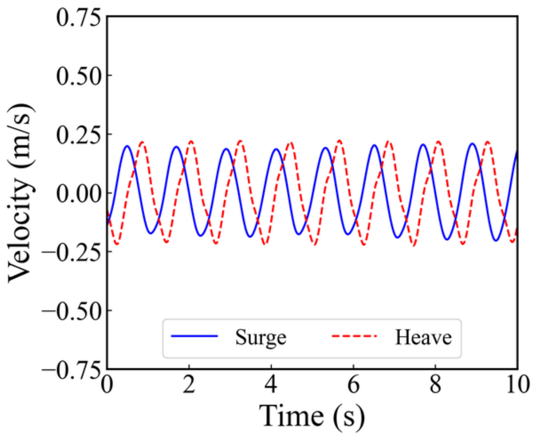

Rope Length

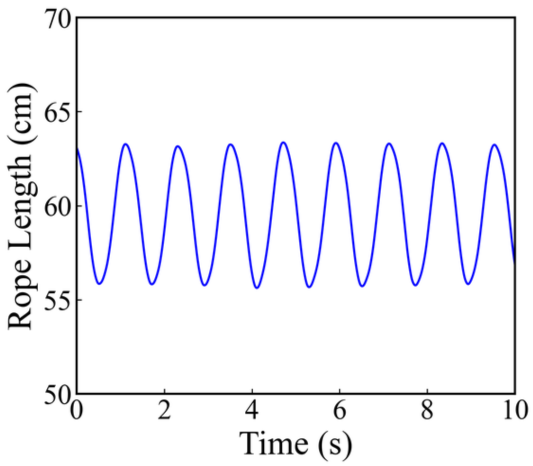

Rope Velocity

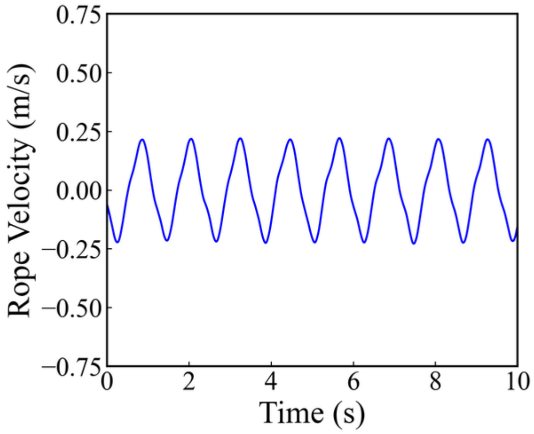

Regular wave: Period1.2s_Height10cm

Trajectory

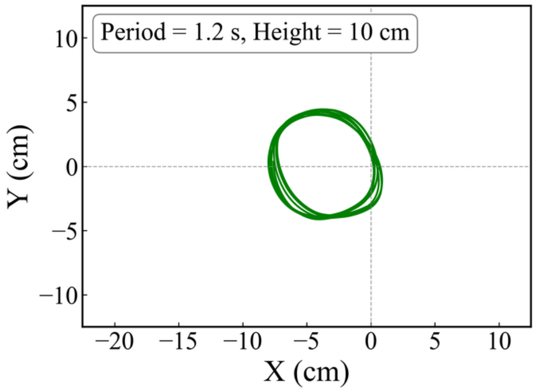

Displacement

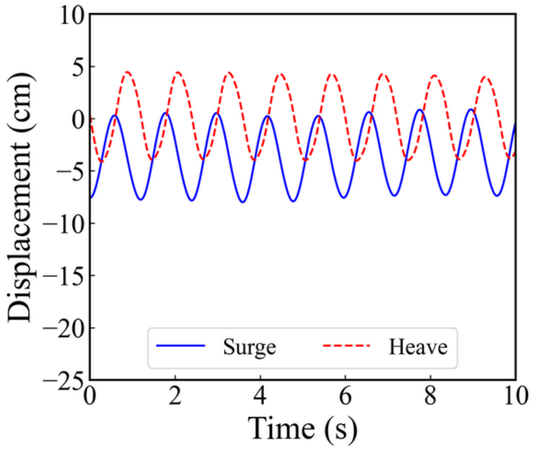

Velocity

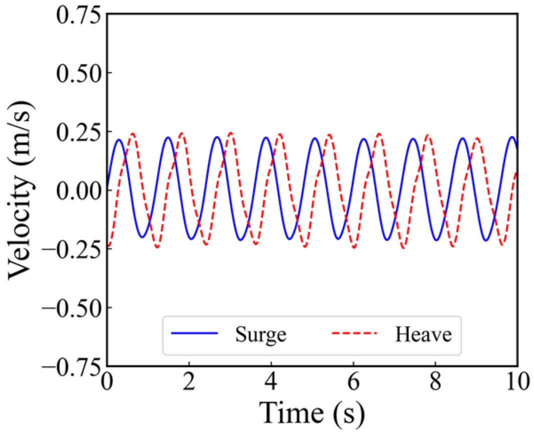

Rope Length

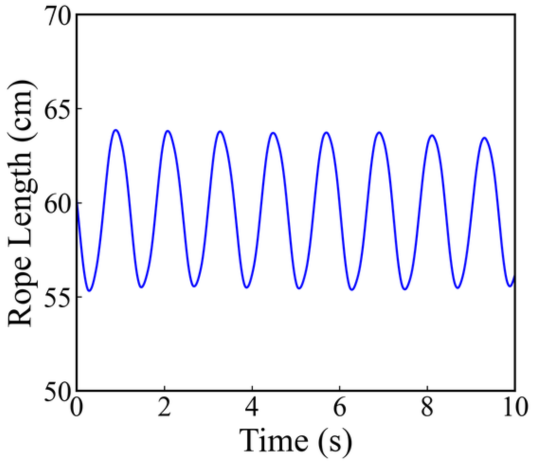

Rope Velocity

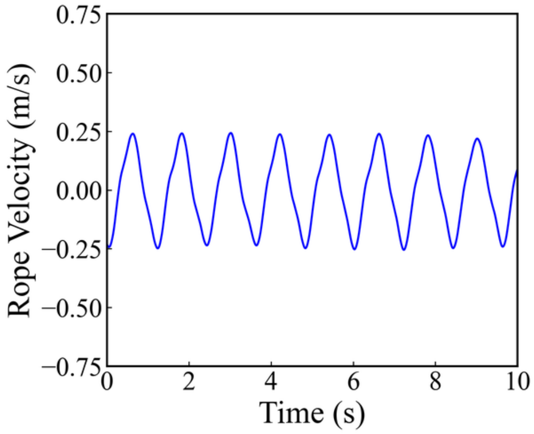

Regular wave: Period1.2s_Height11cm

Trajectory

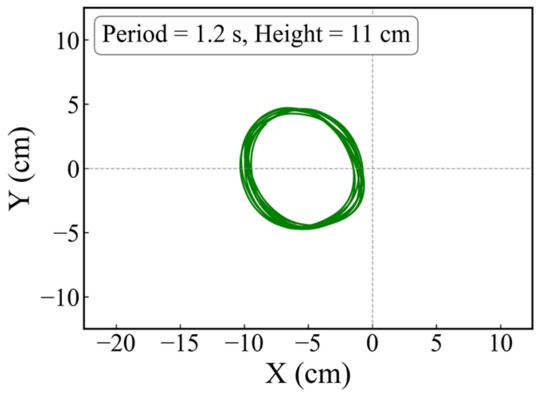

Displacement

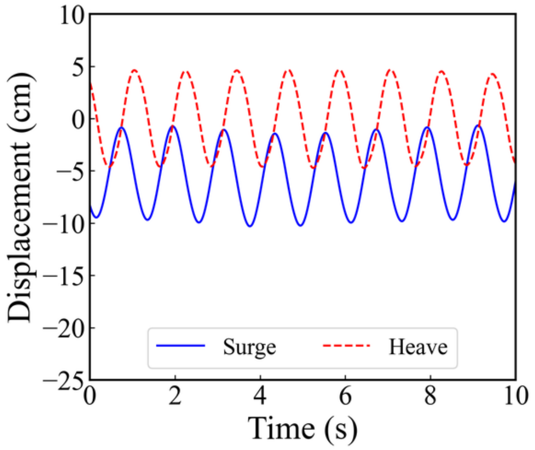

Velocity

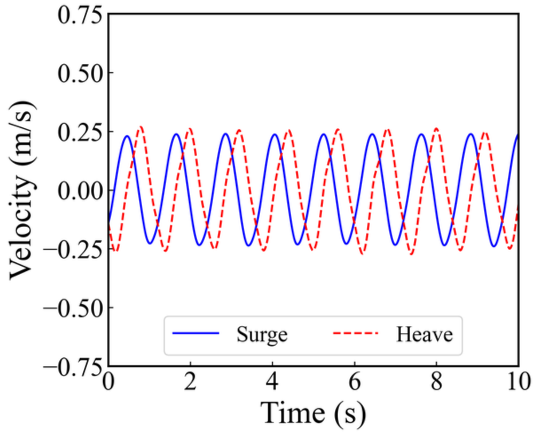

Rope Length

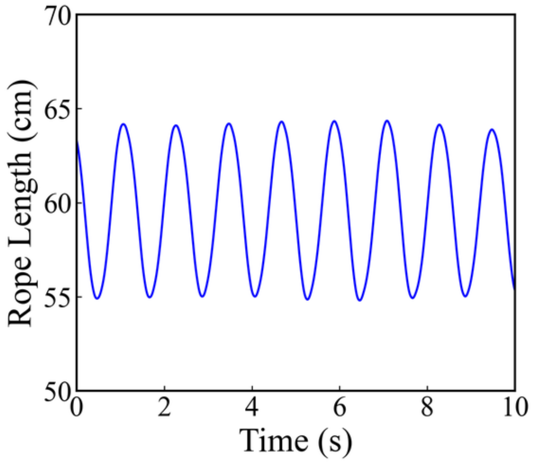

Rope Velocity

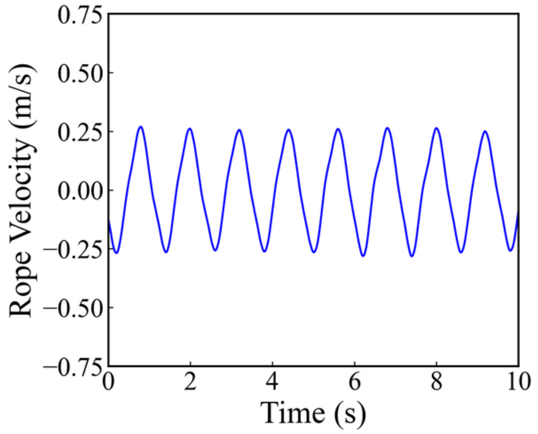

Regular wave: Period1.2s_Height12cm

Trajectory

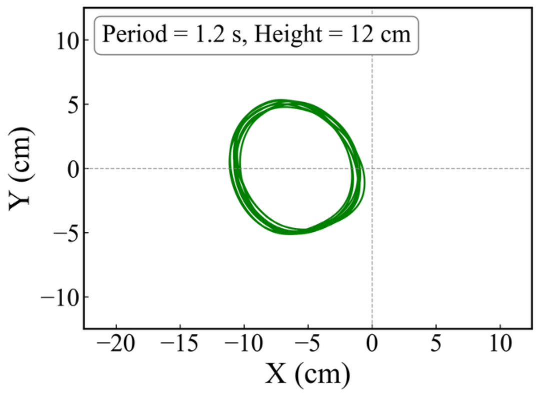

Displacement

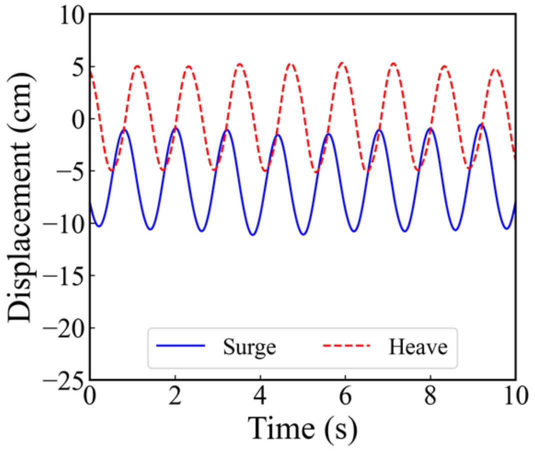

Velocity

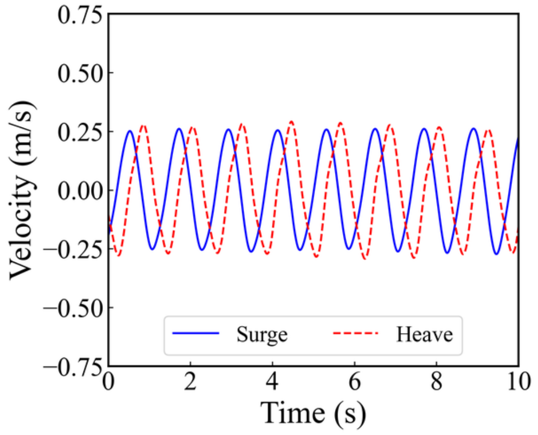

Rope Length

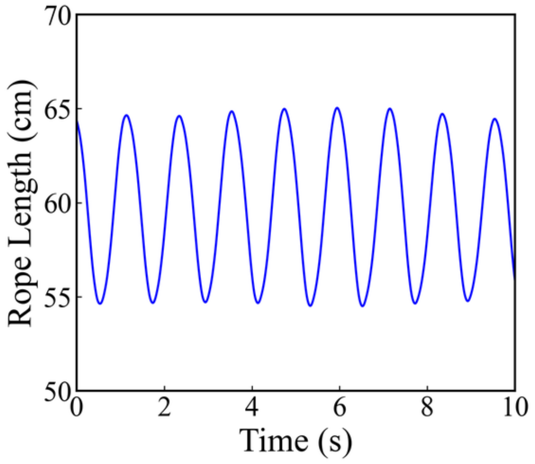

Rope Velocity

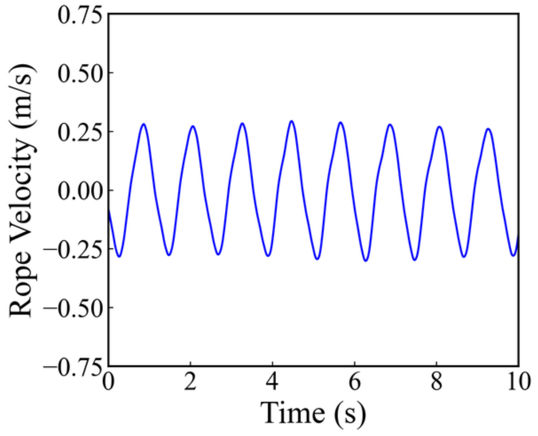

Regular wave: Period1.2s_Height13cm

Trajectory

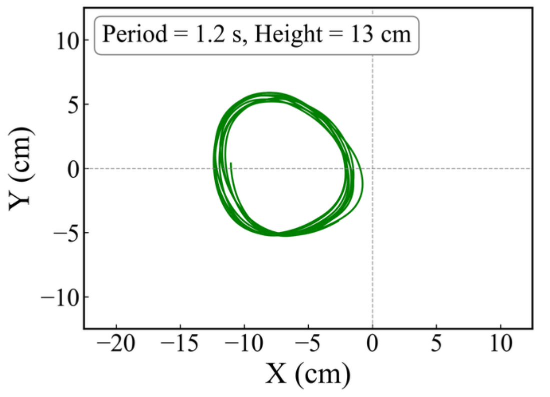

Displacement

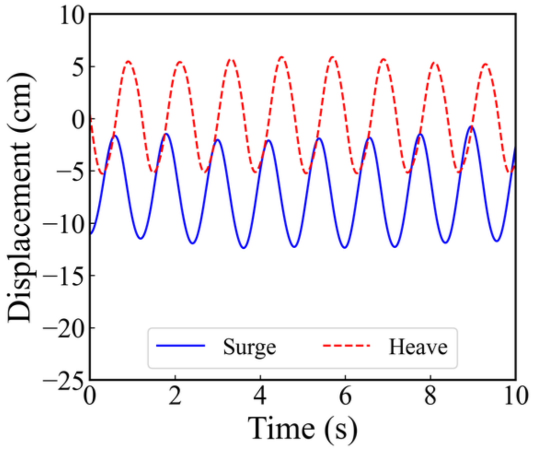

Velocity

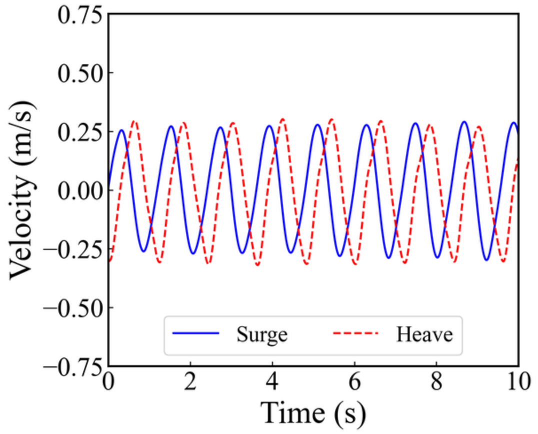

Rope Length

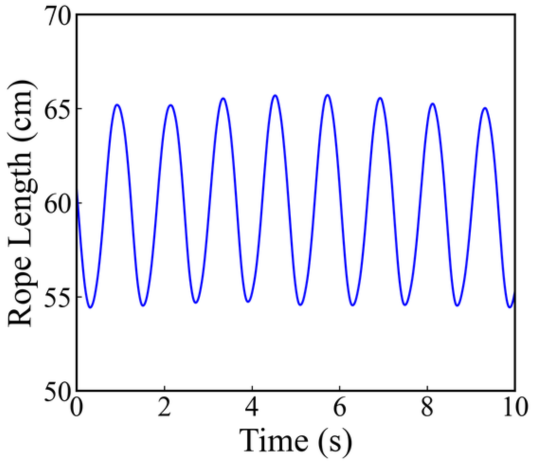

Rope Velocity

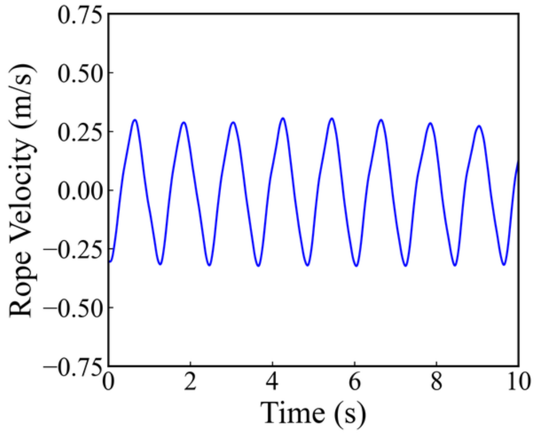

Regular wave: Period1.2s_Height14cm

Trajectory

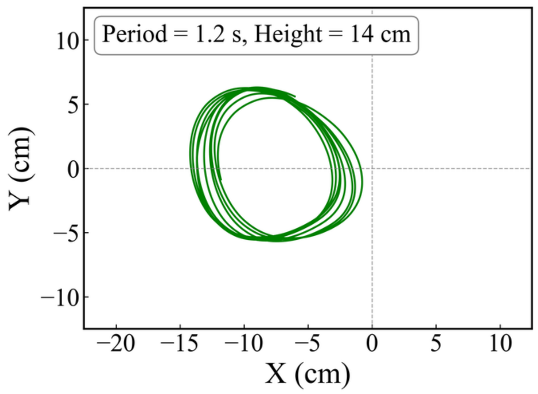

Displacement

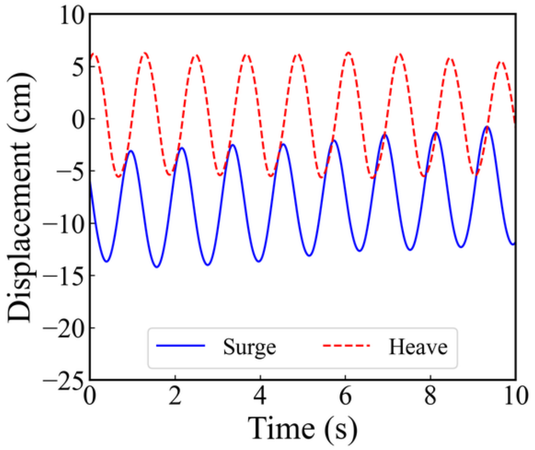

Velocity

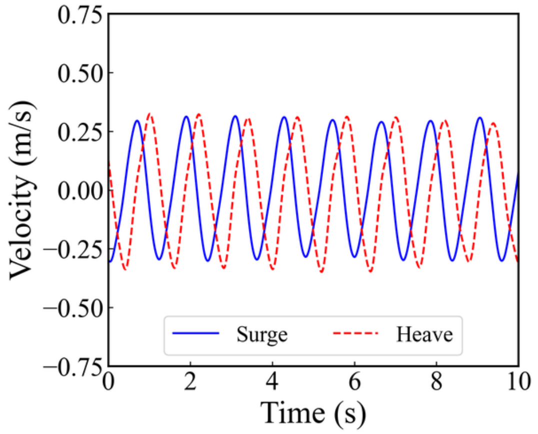

Rope Length

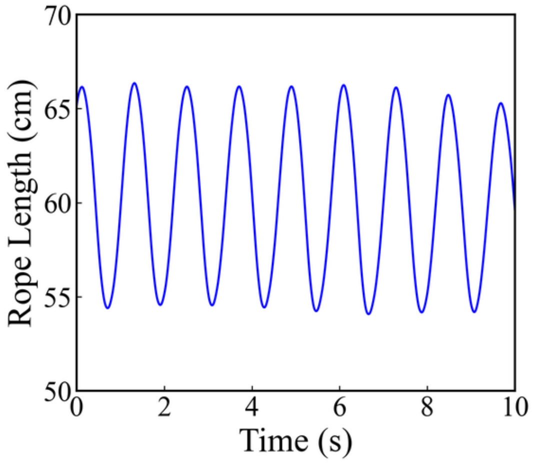

Rope Velocity

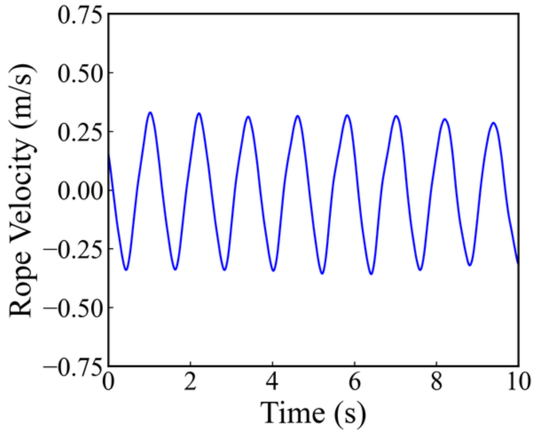

Regular wave: Period1.2s_Height15cm

Trajectory

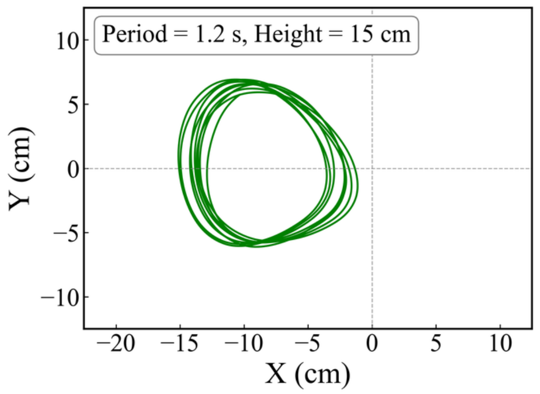

Displacement

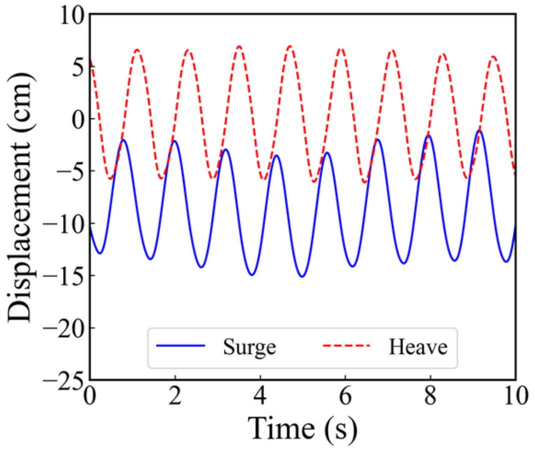

Velocity

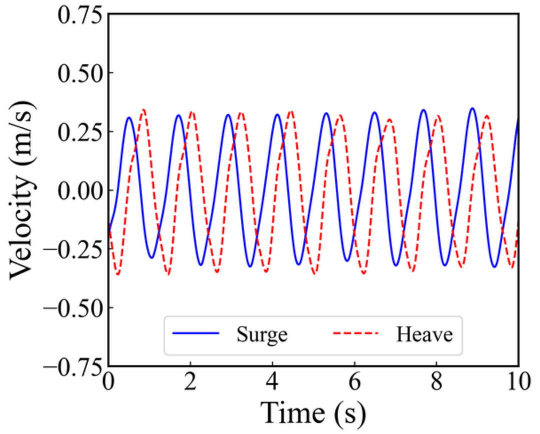

Rope Length

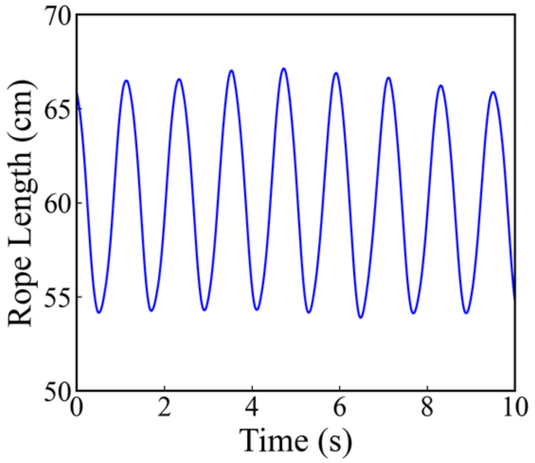

Rope Velocity

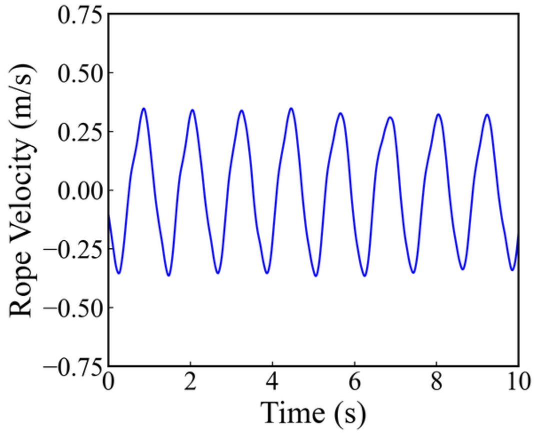

Regular wave: Period1.3s_Height5cm

Trajectory

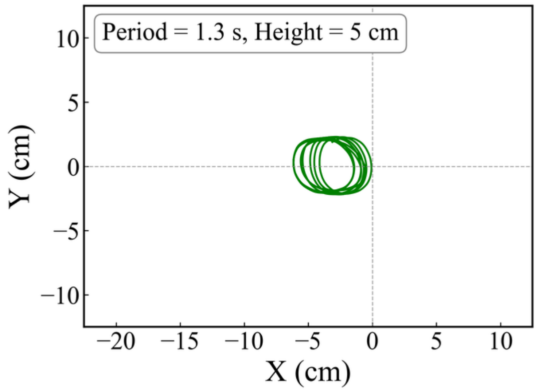

Displacement

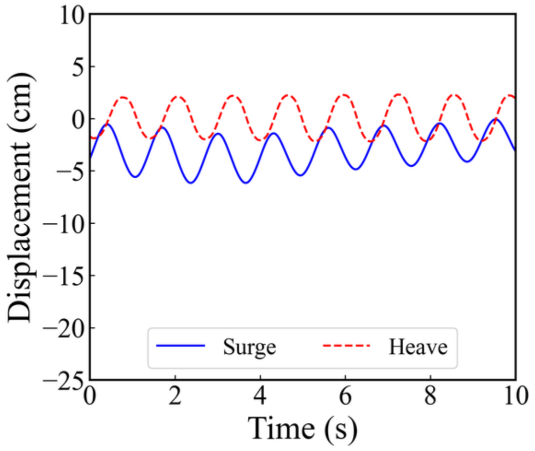

Velocity

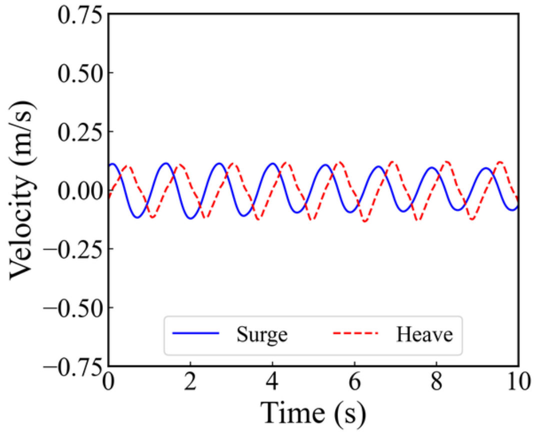

Rope Length

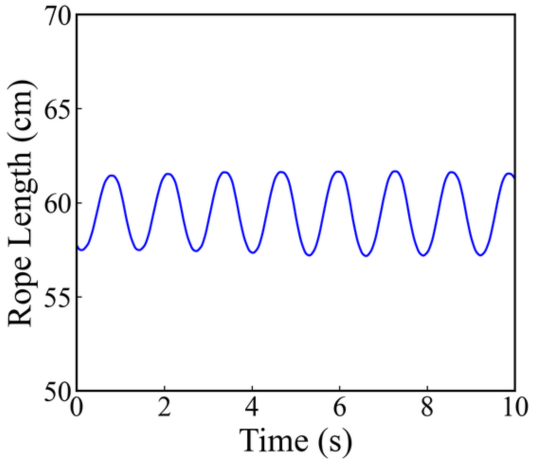

Rope Velocity

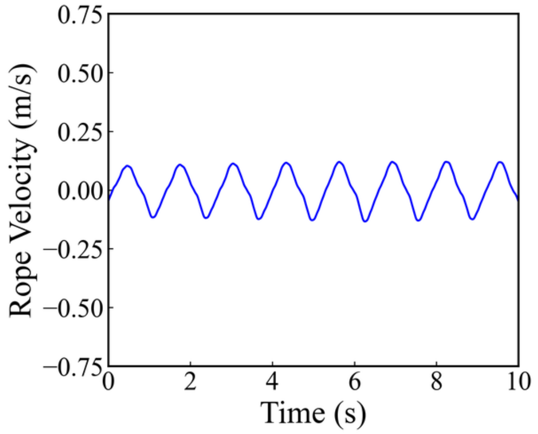

Regular wave: Period1.3s_Height6cm

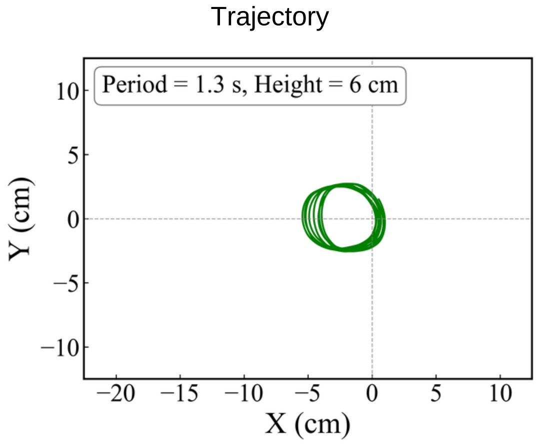

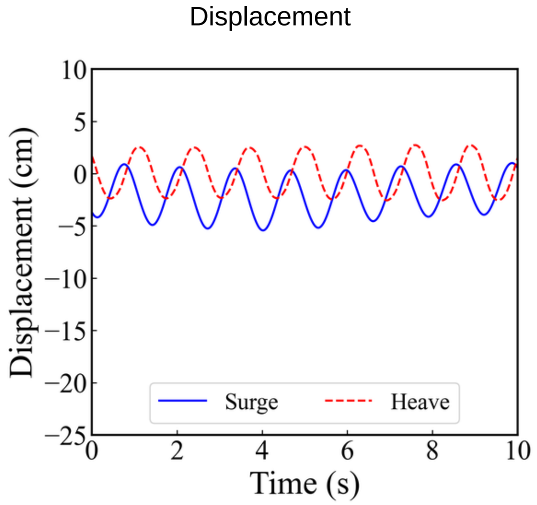

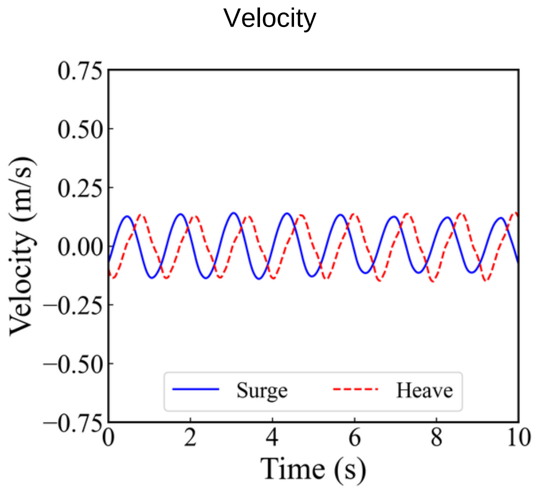

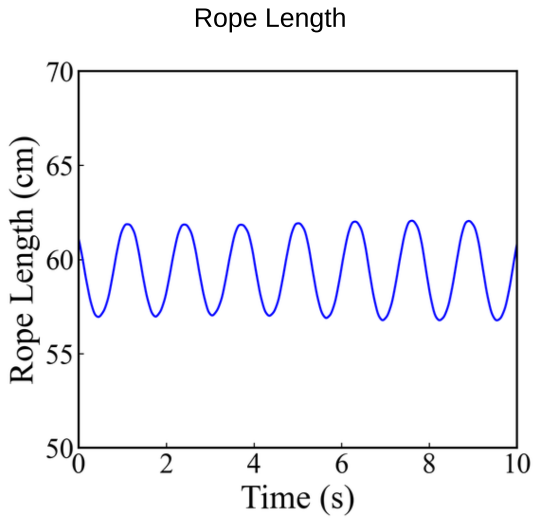

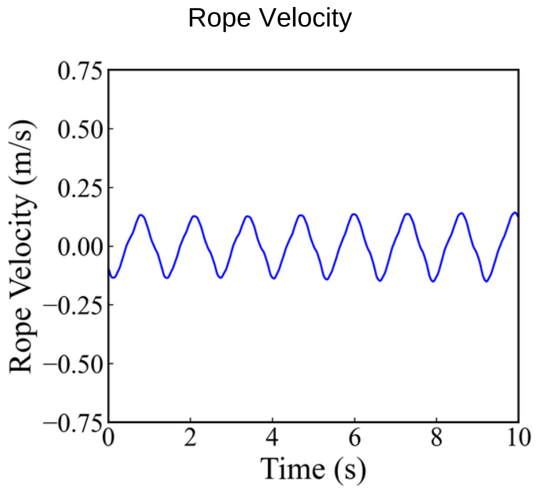

Regular wave: Period1.3s_Height7cm

Trajectory

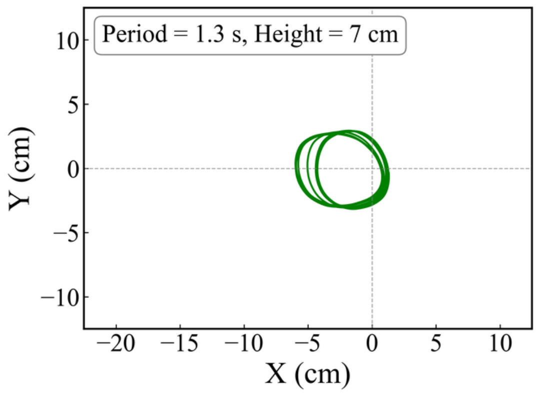

Displacement

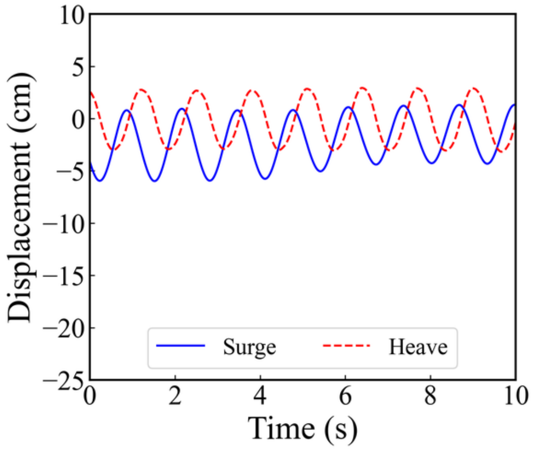

Velocity

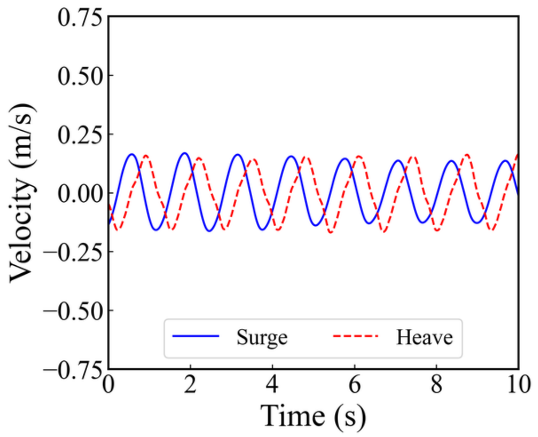

Rope Length

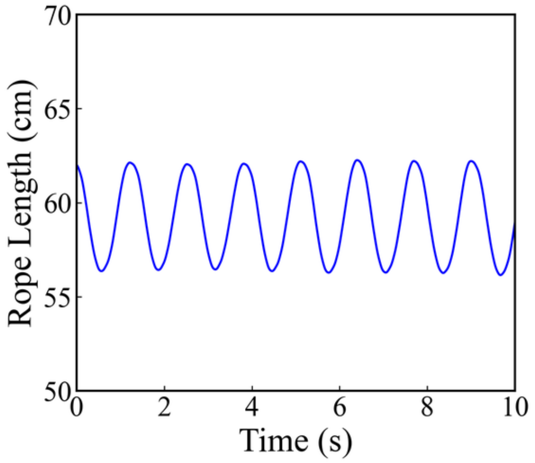

Rope Velocity

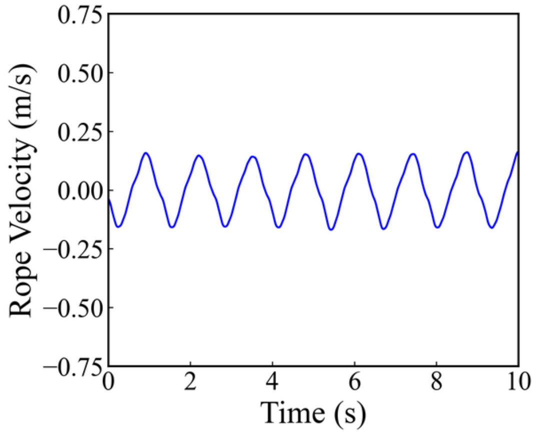

Regular wave: Period1.3s_Height8cm

Trajectory

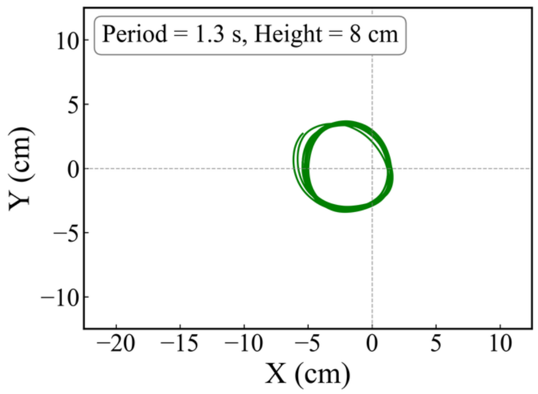

Displacement

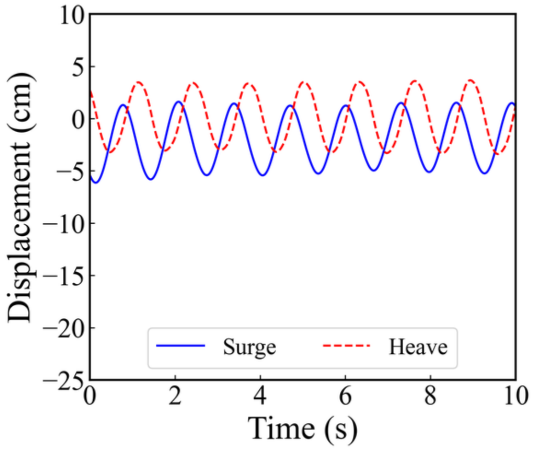

Velocity

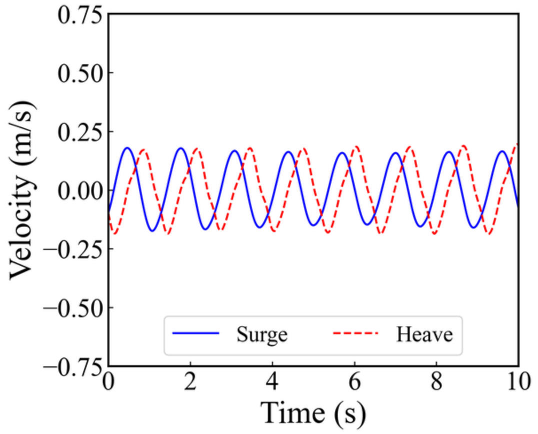

Rope Length

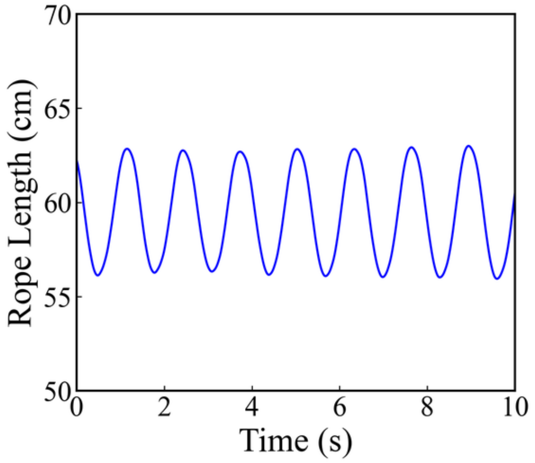

Rope Velocity

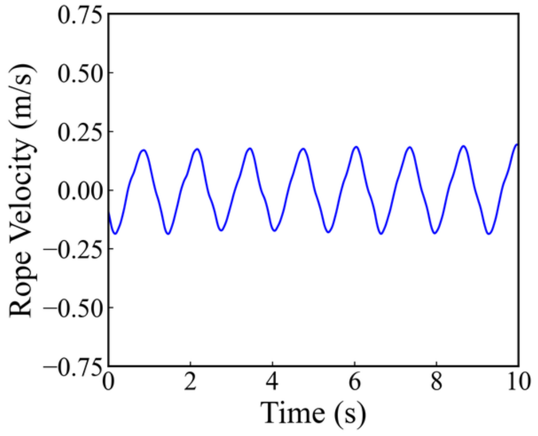

Regular wave: Period1.3s_Height9cm

Trajectory

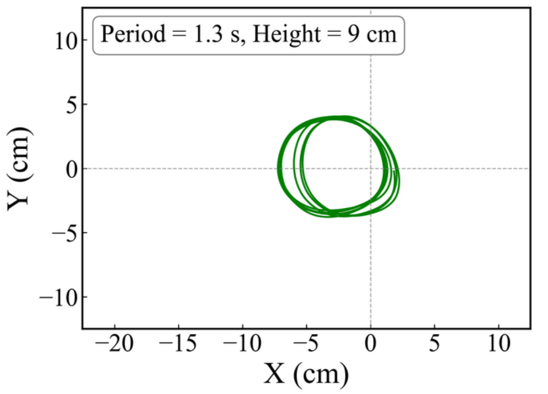

Displacement

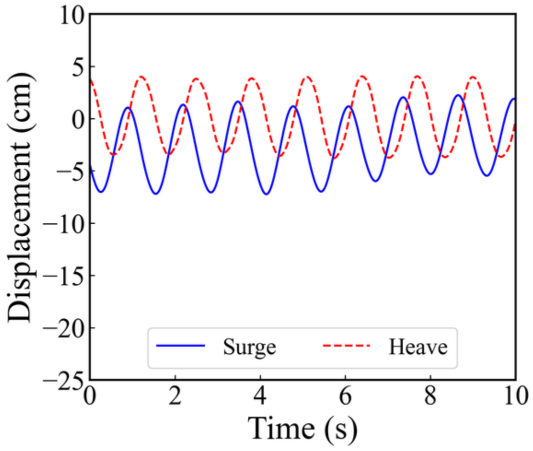

Velocity

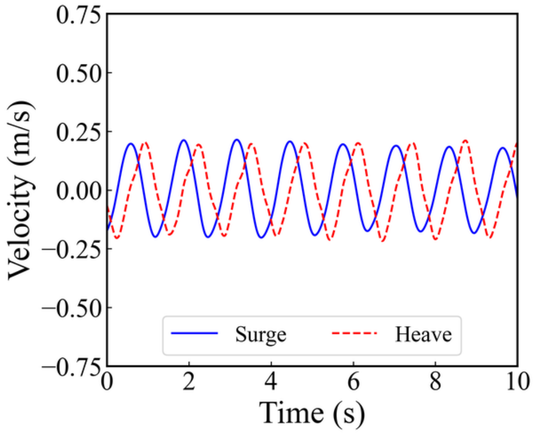

Rope Length

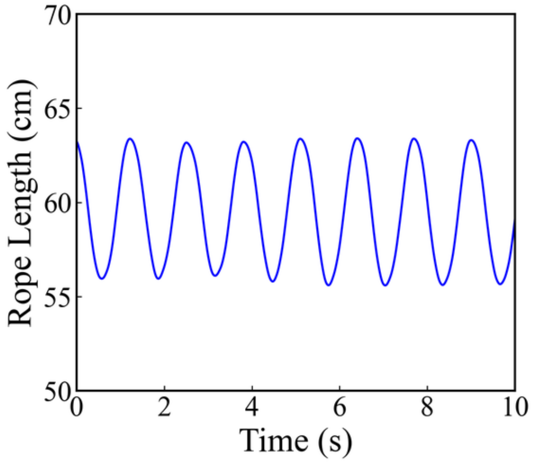

Rope Velocity

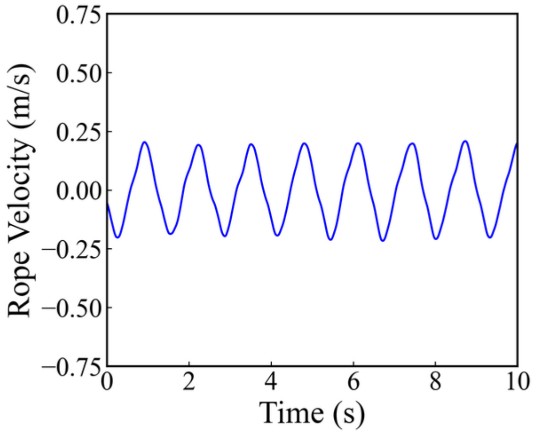

Regular wave: Period1.3s_Height10cm

Trajectory

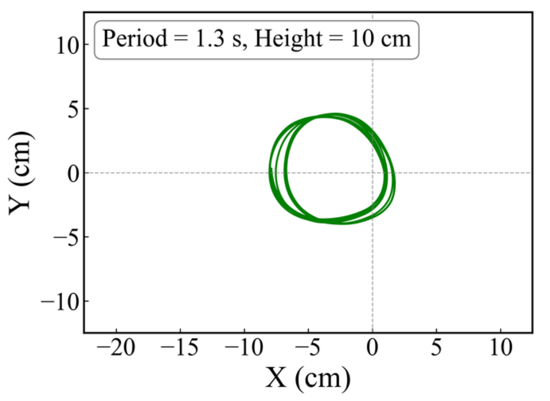

Displacement

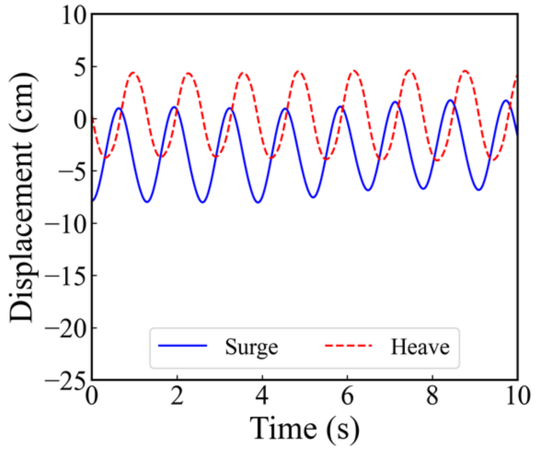

Velocity

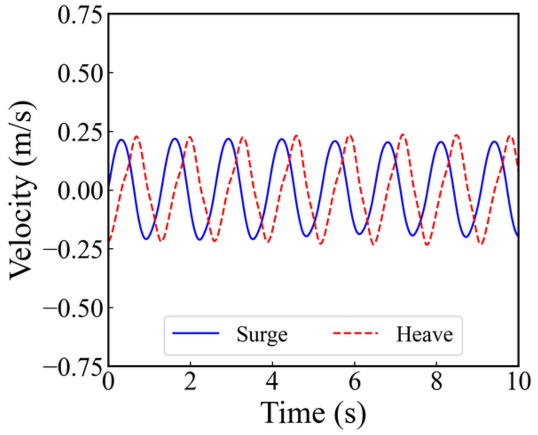

Rope Length

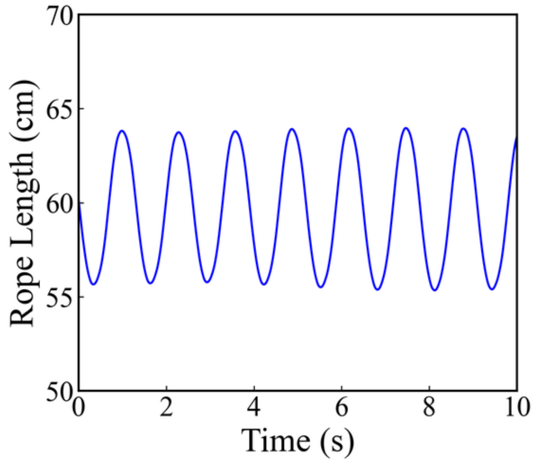

Rope Velocity

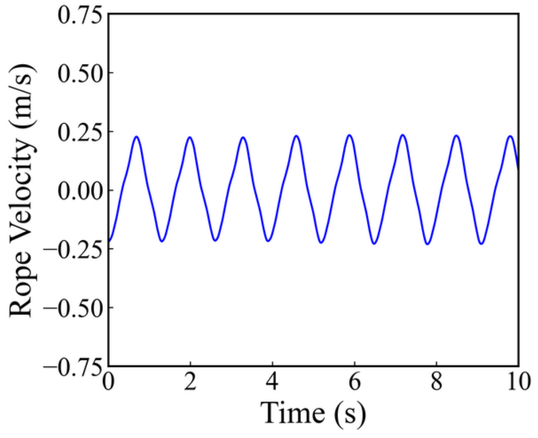

Regular wave: Period1.3s_Height11cm

Trajectory

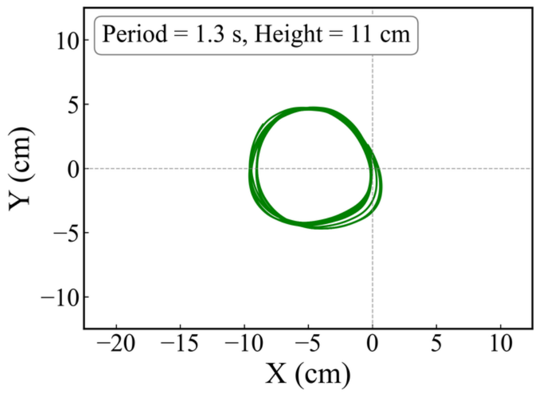

Displacement

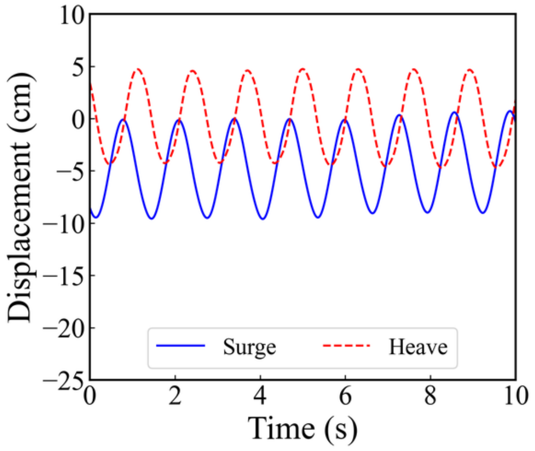

Velocity

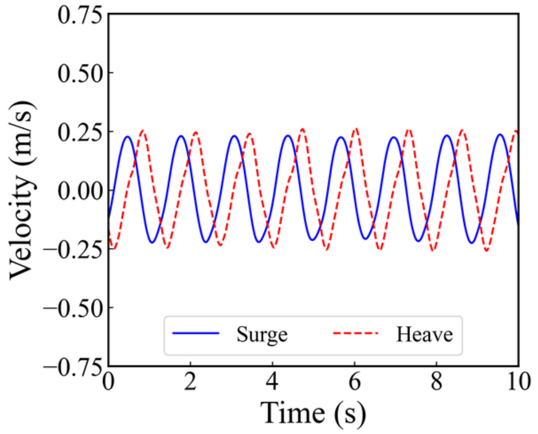

Rope Length

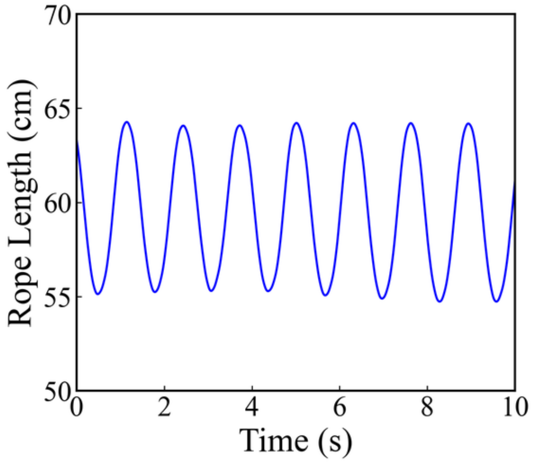

Rope Velocity

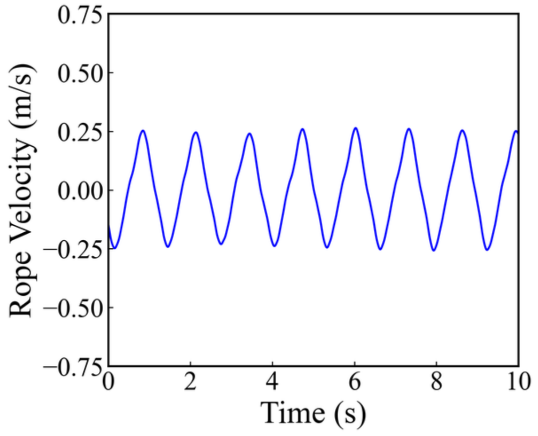

Regular wave: Period1.3s_Height12cm

Trajectory

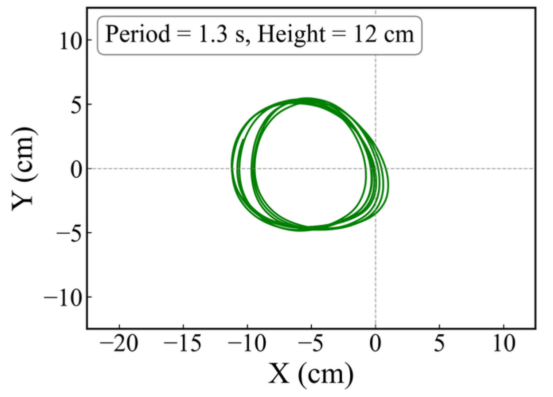

Displacement

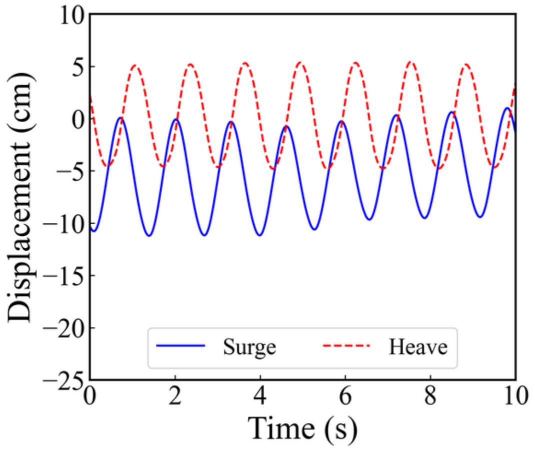

Velocity

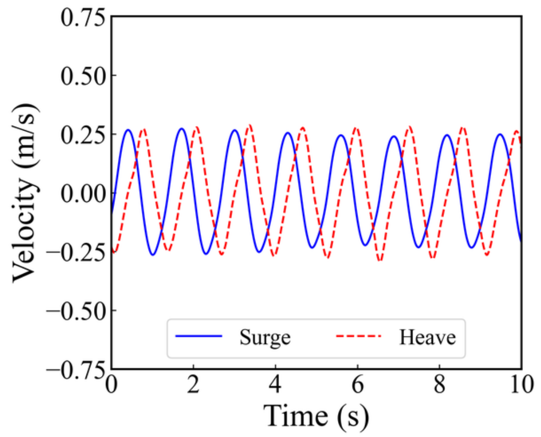

Rope Length

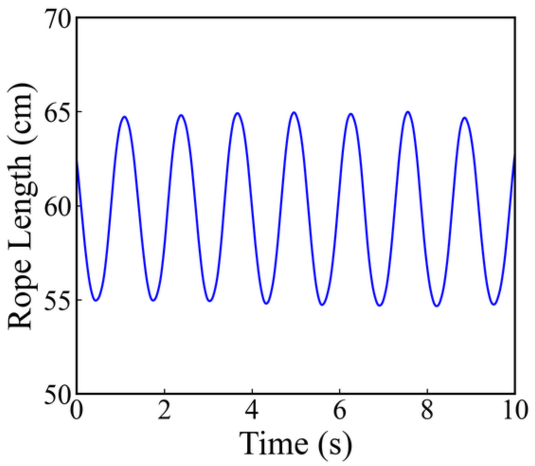

Rope Velocity

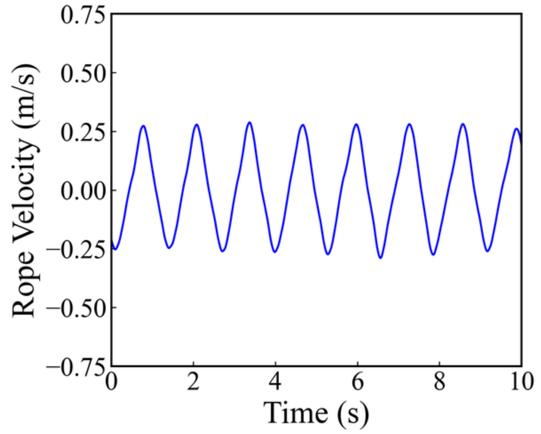

Regular wave: Period1.3s_Height13cm

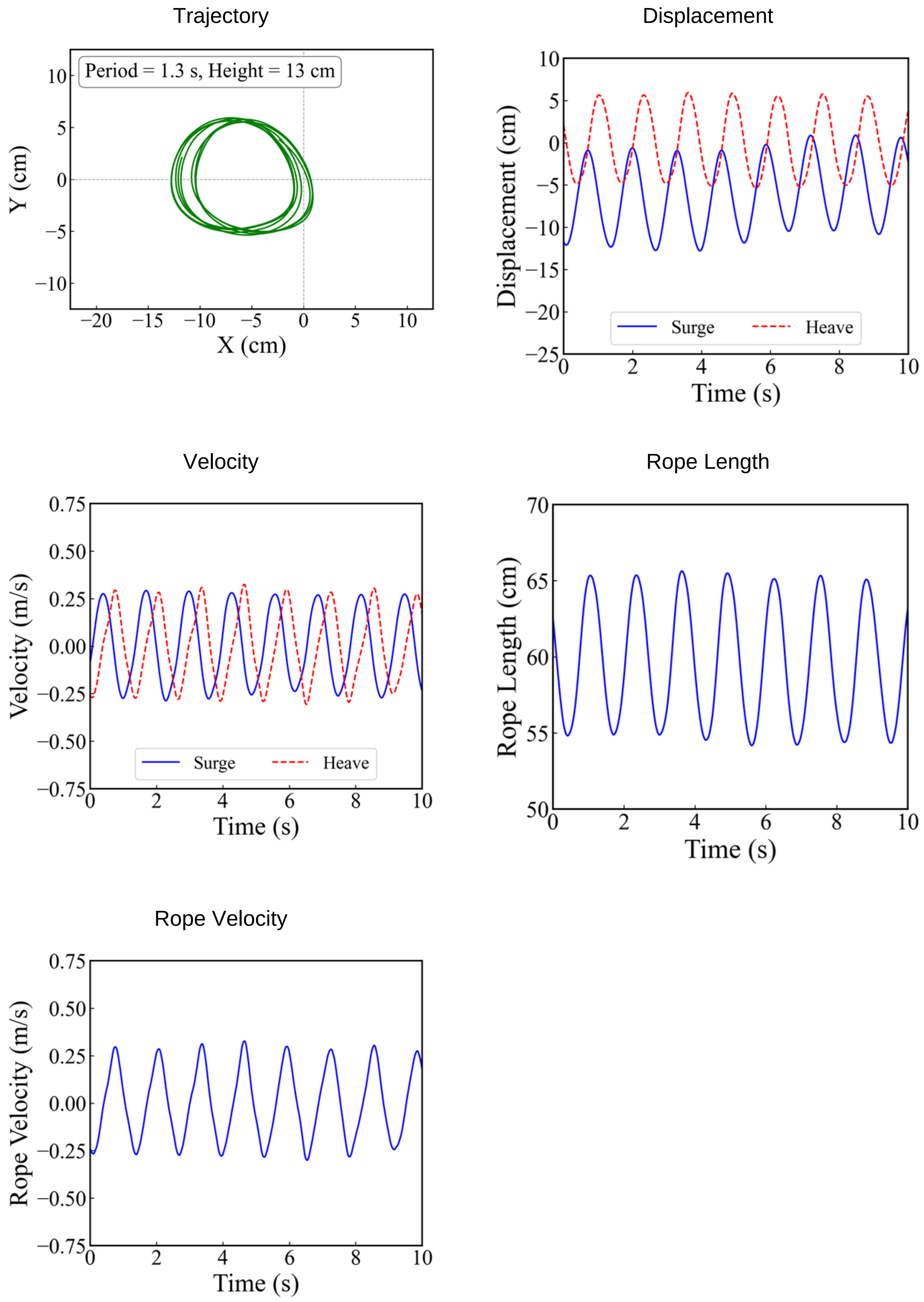

Regular wave: Period1.3s_Height14cm

Trajectory

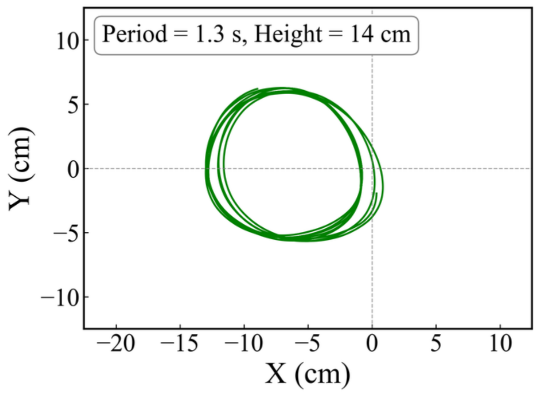

Displacement

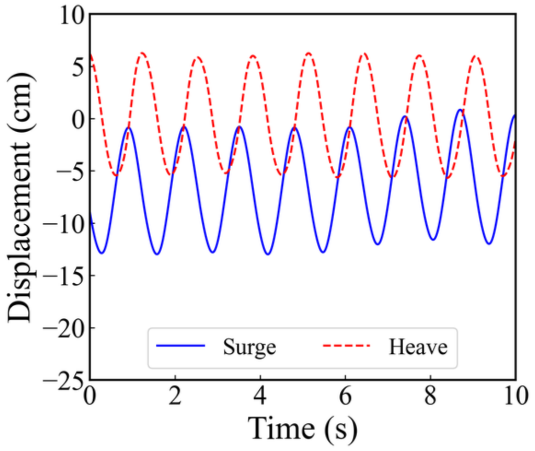

Velocity

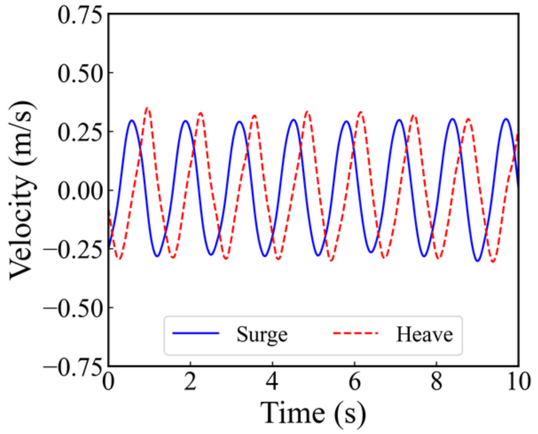

Rope Length

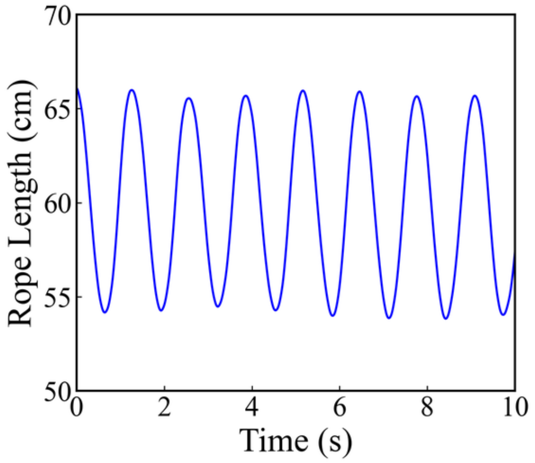

Rope Velocity

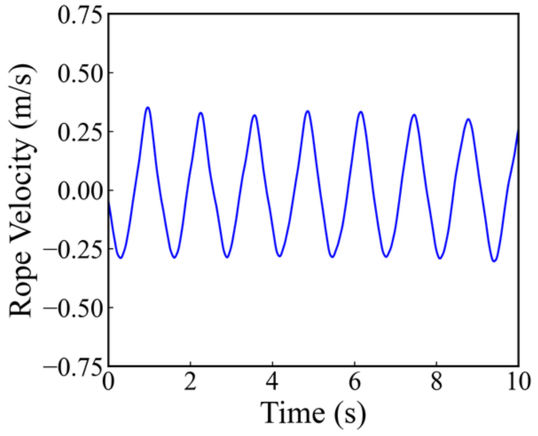

Regular wave: Period1.3s_Height15cm

Trajectory

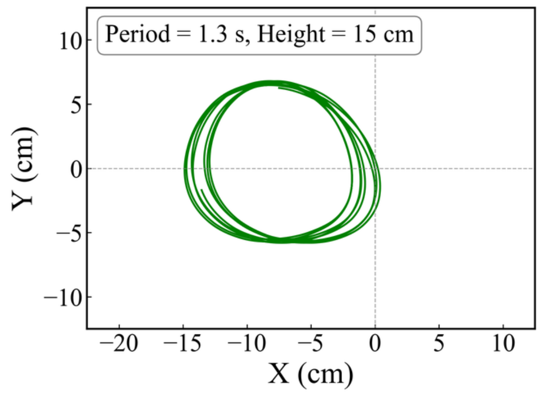

Displacement

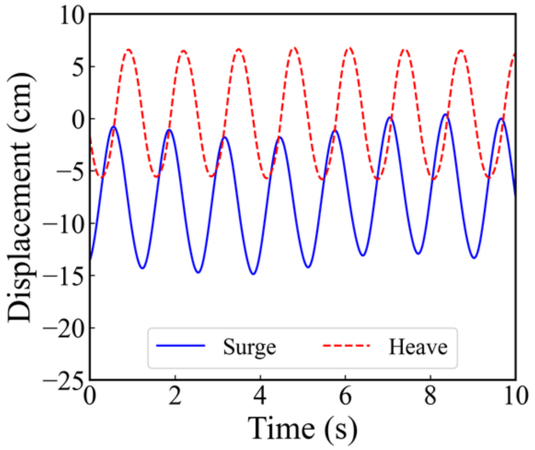

Velocity

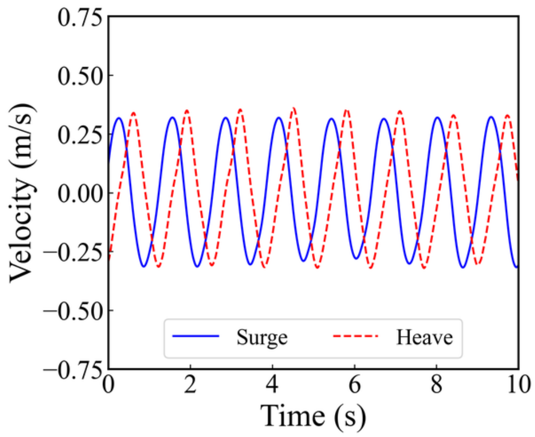

Rope Length

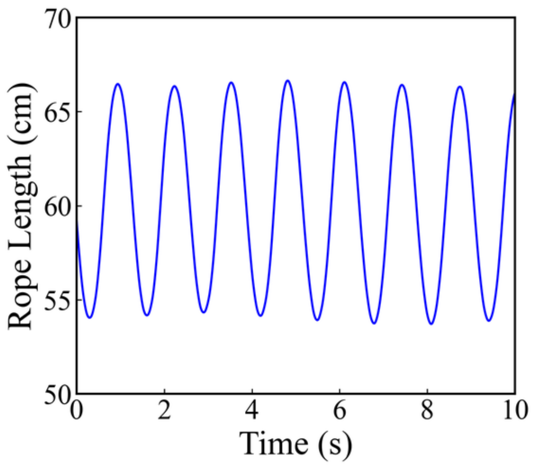

Rope Velocity

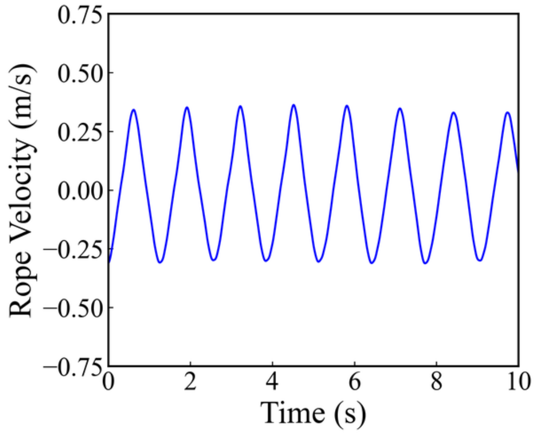

Regular wave: Period1.4s_Height5cm

Trajectory

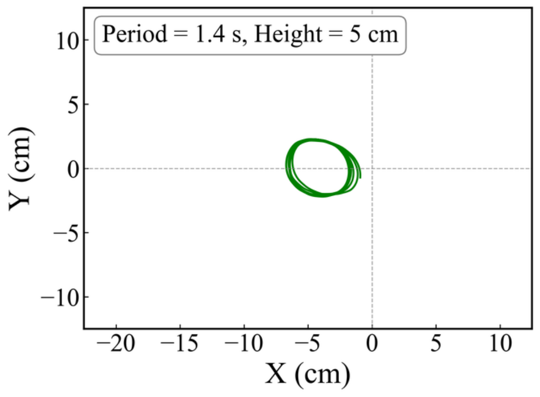

Displacement

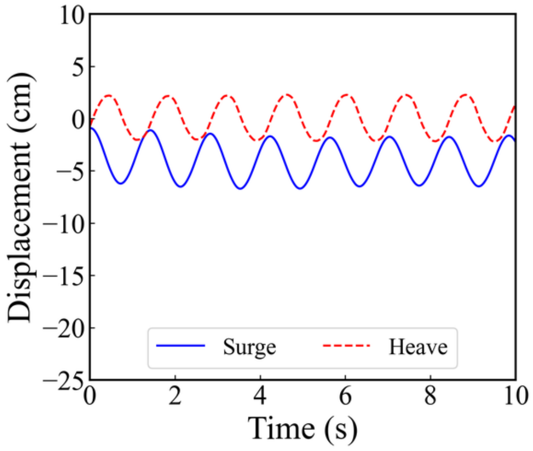

Velocity

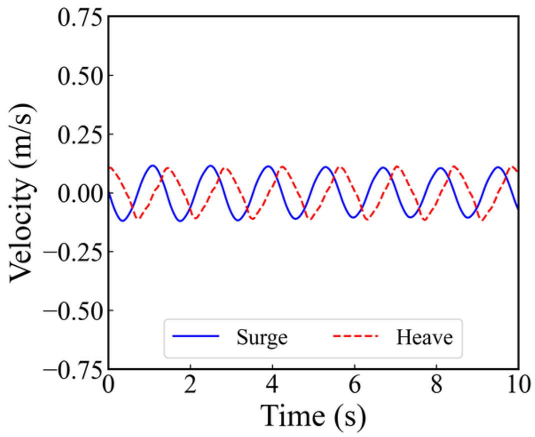

Rope Length

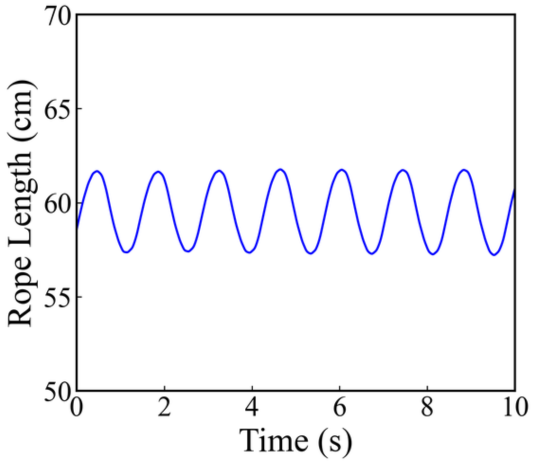

Rope Velocity

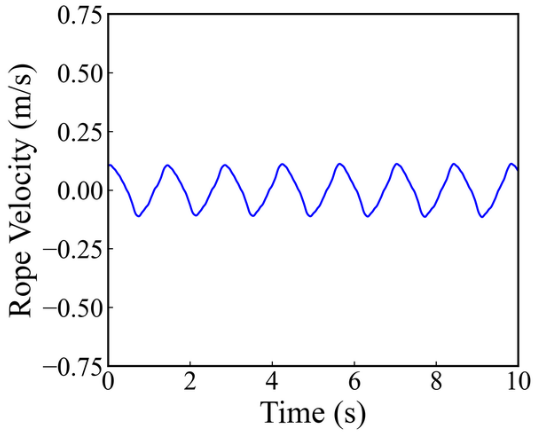

Regular wave: Period1.4s_Height6cm

Trajectory

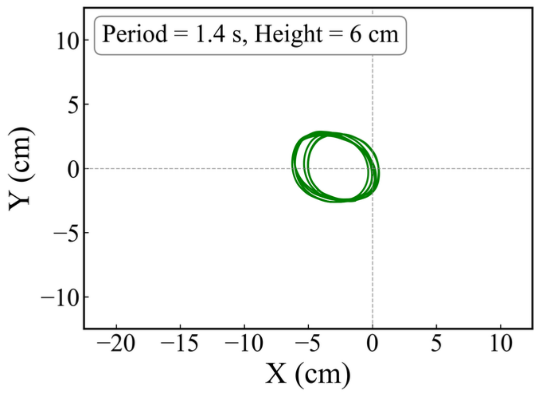

Displacement

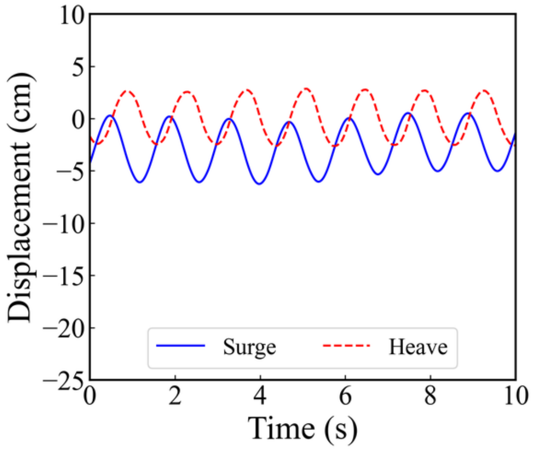

Velocity

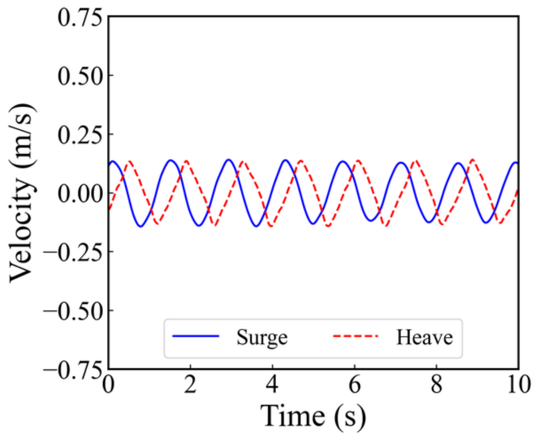

Rope Length

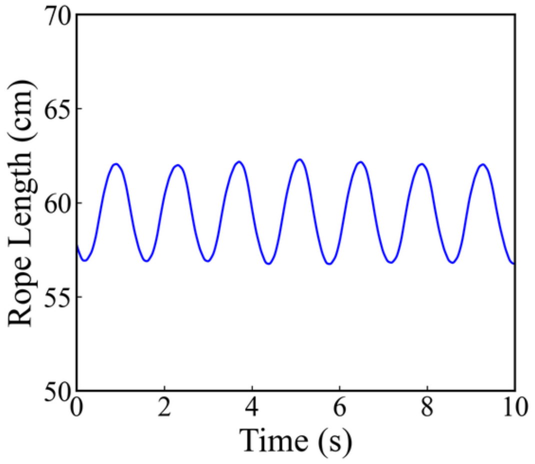

Rope Velocity

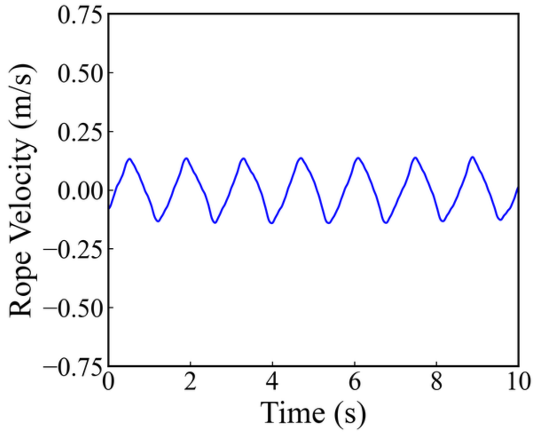

Regular wave: Period1.4s_Height7cm

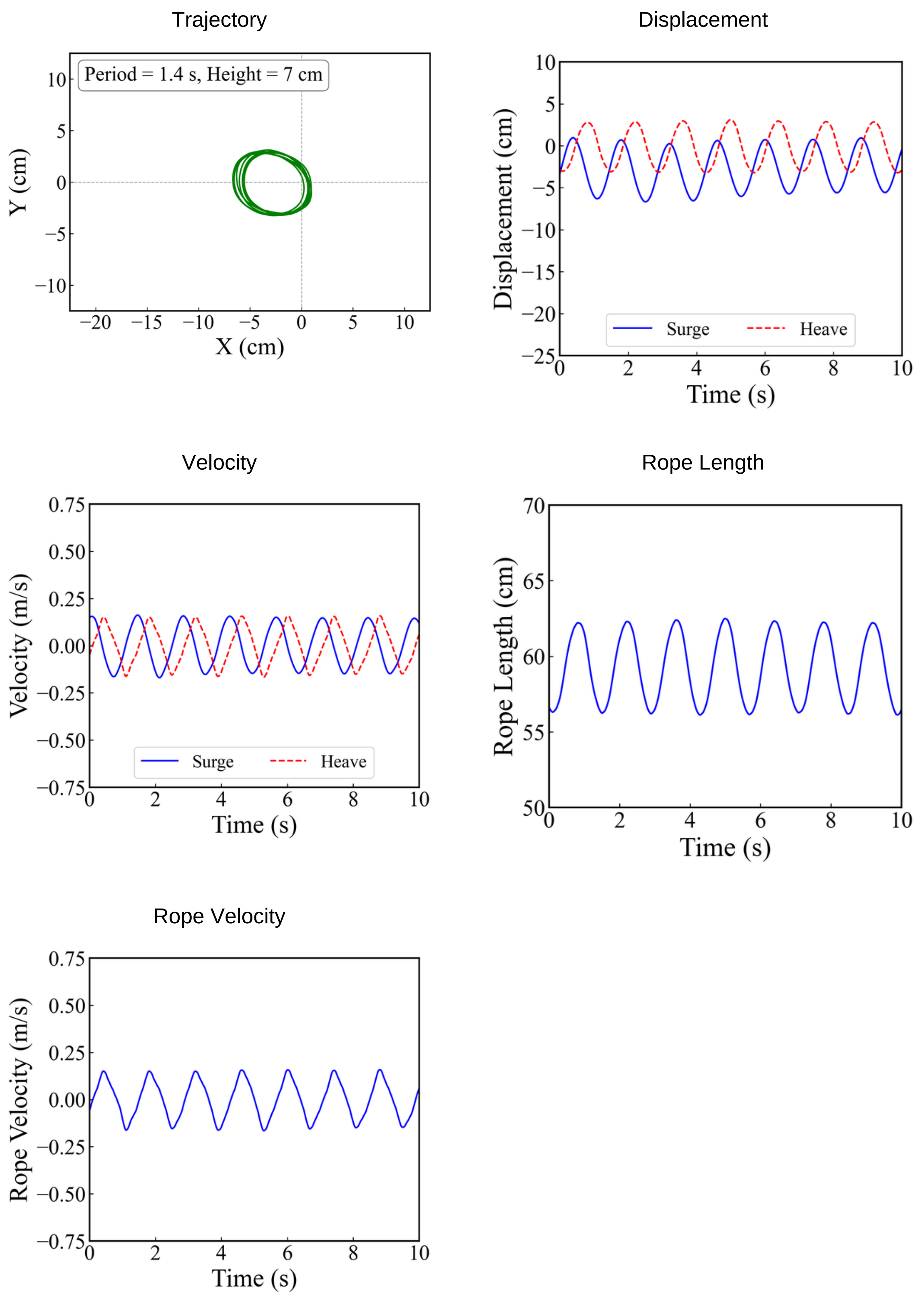

Regular wave: Period1.4s_Height8cm

Trajectory

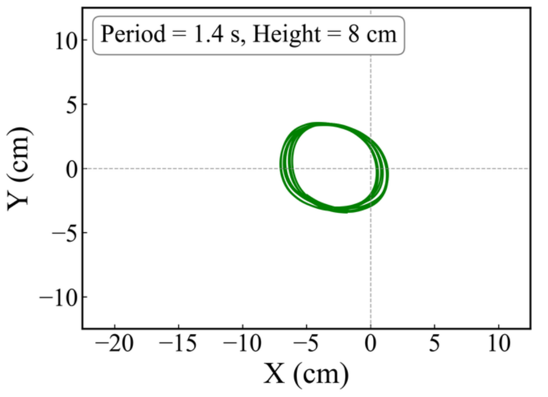

Displacement

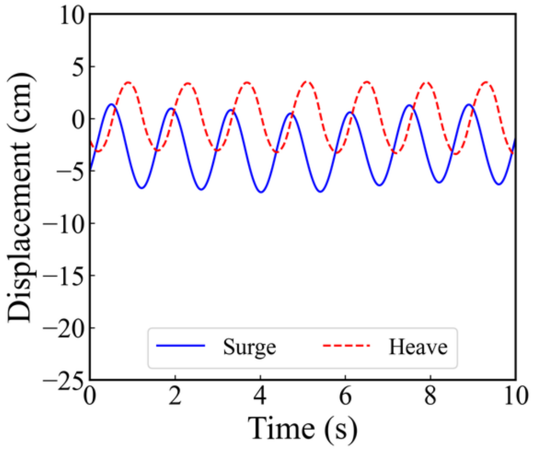

Velocity

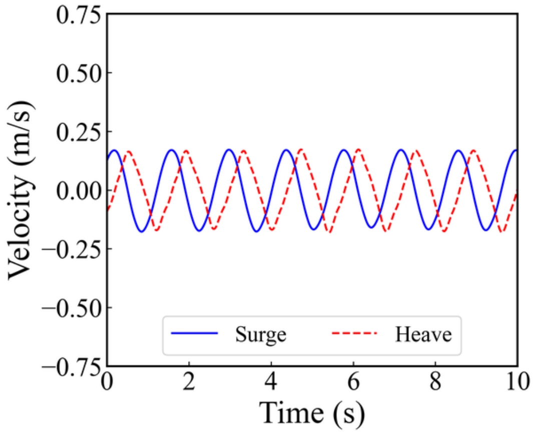

Rope Length

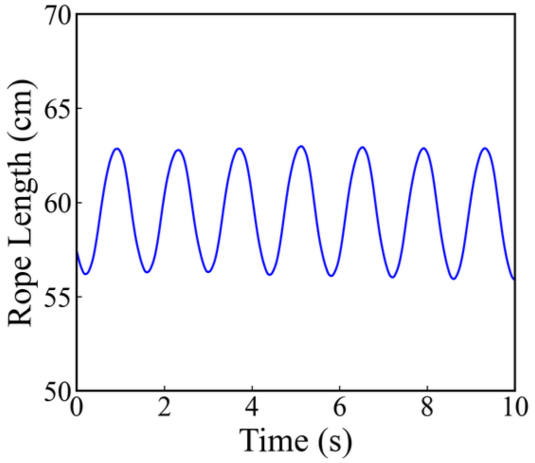

Rope Velocity

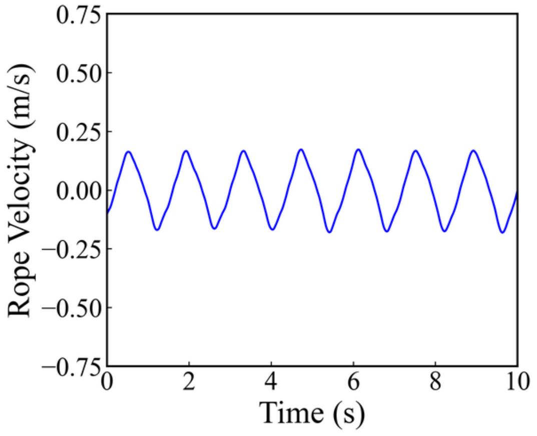

Regular wave: Period1.4s_Height9cm

Trajectory

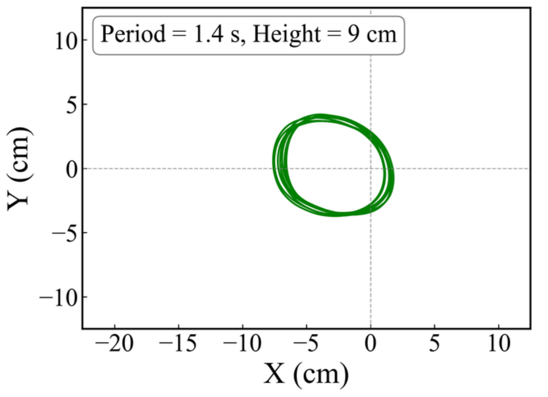

Displacement

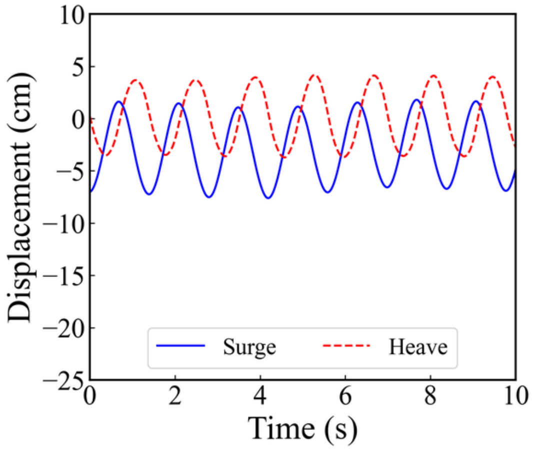

Velocity

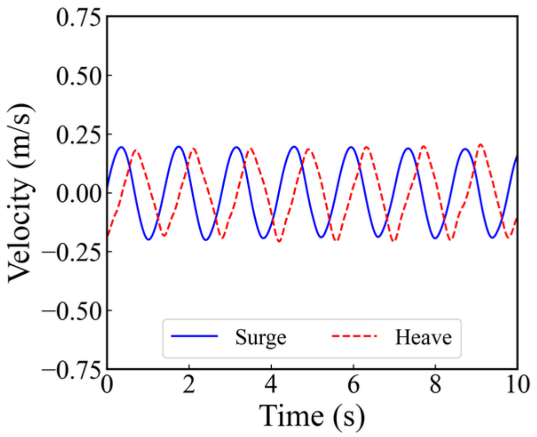

Rope Length

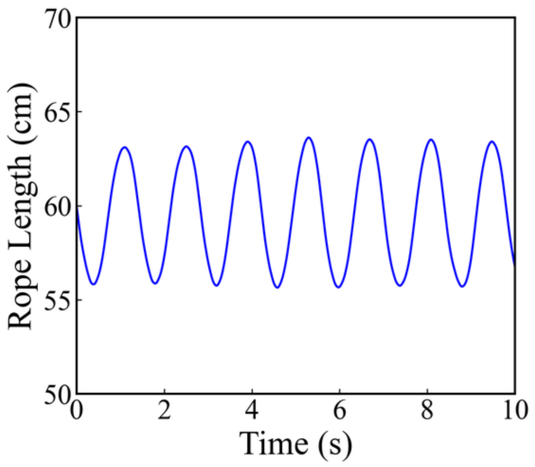

Rope Velocity

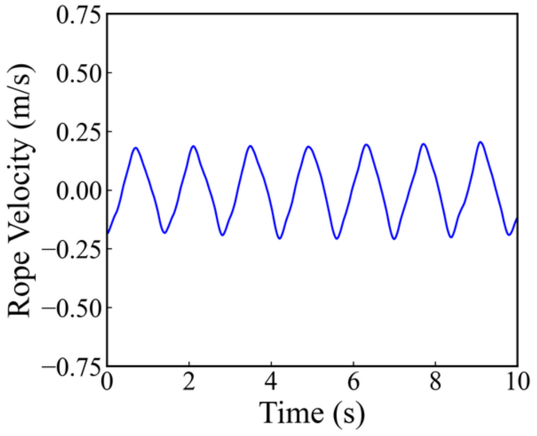

Regular wave: Period1.4s_Height10cm

Trajectory

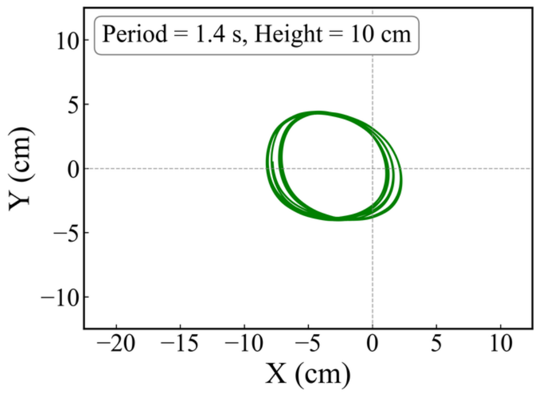

Displacement

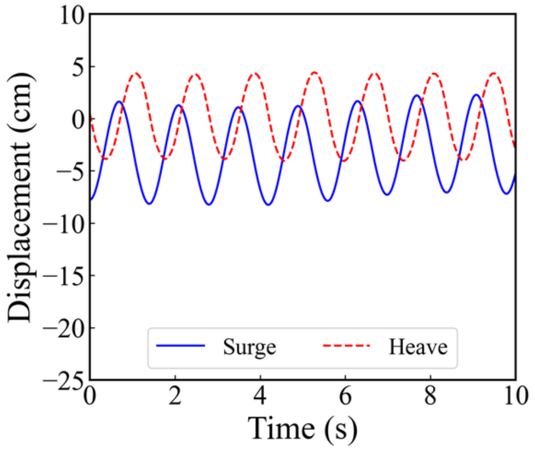

Velocity

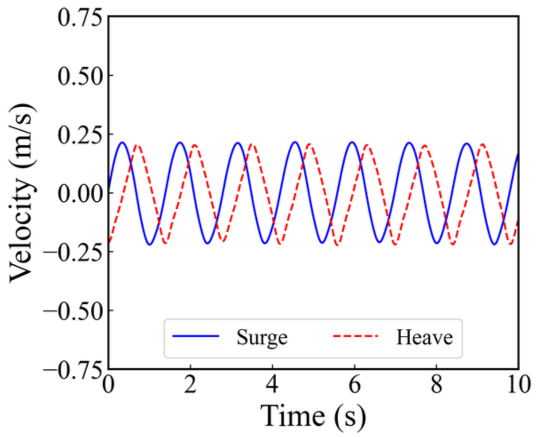

Rope Length

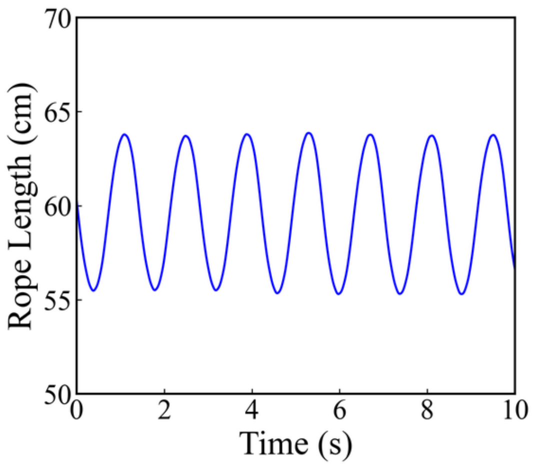

Rope Velocity

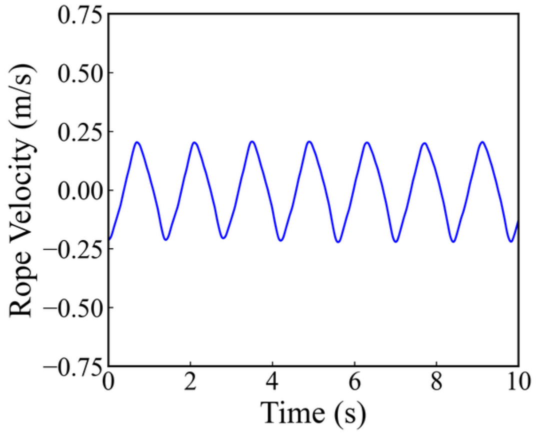

Regular wave: Period1.4s_Height11cm

Trajectory

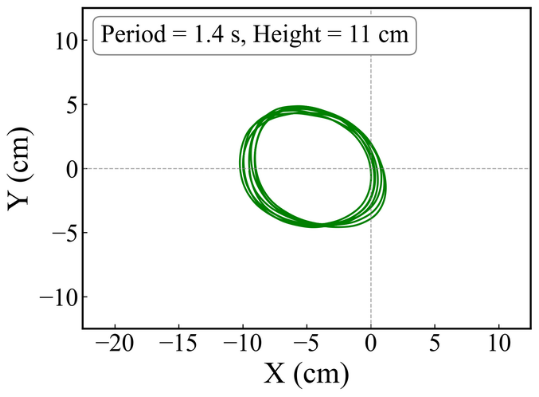

Displacement

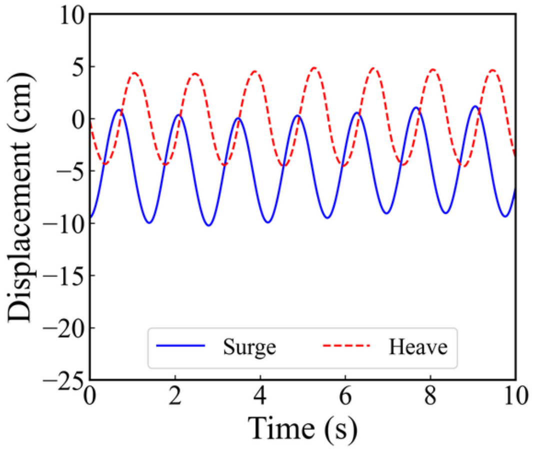

Velocity

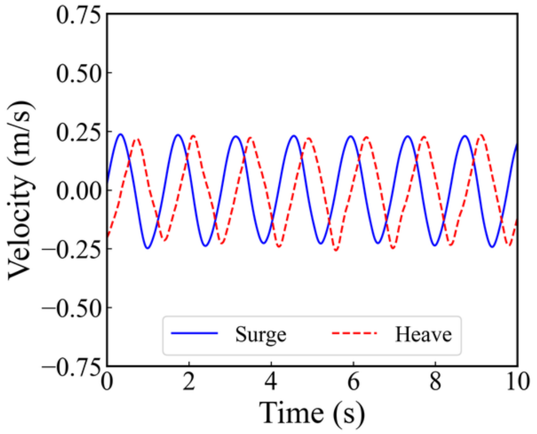

Rope Length

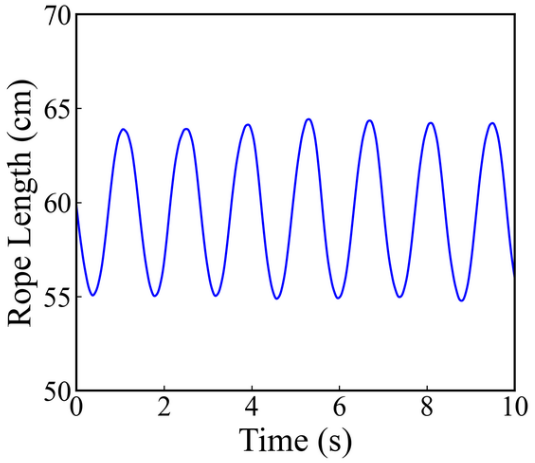

Rope Velocity

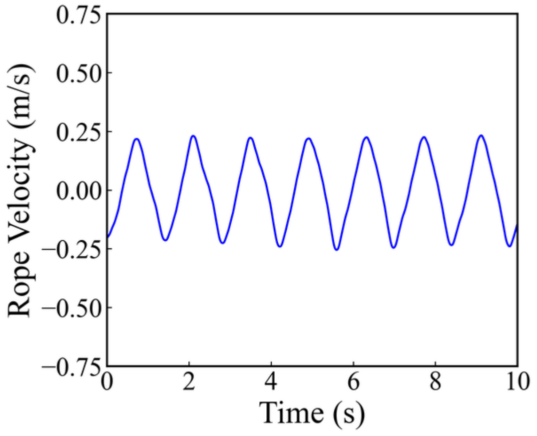

Regular wave: Period1.4s_Height12cm

Trajectory

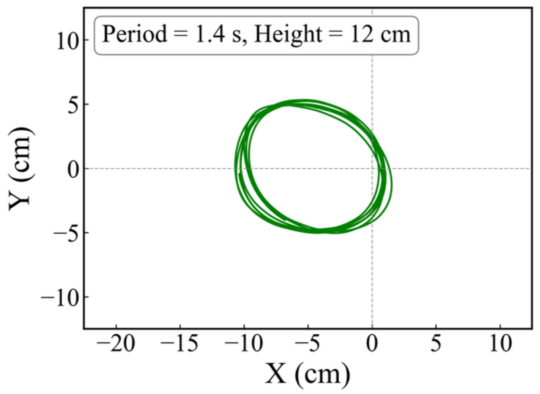

Displacement

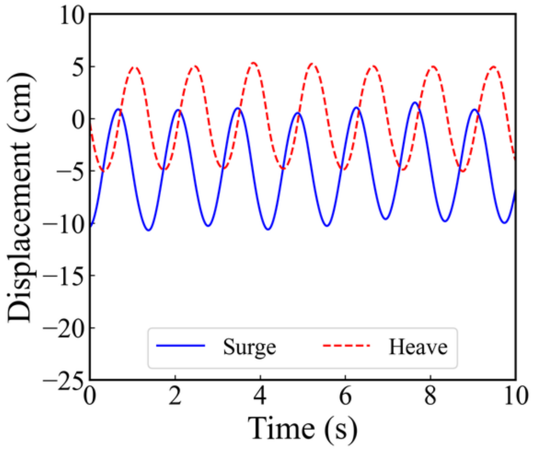

Velocity

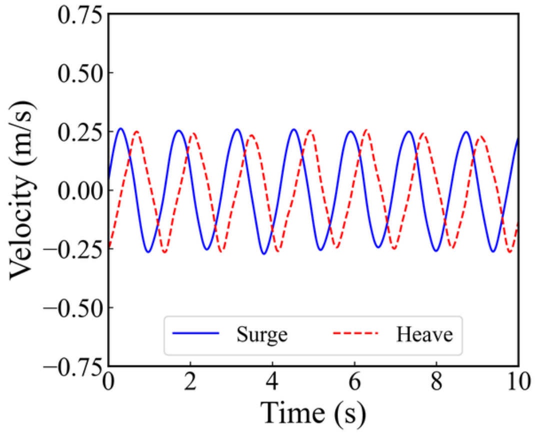

Rope Length

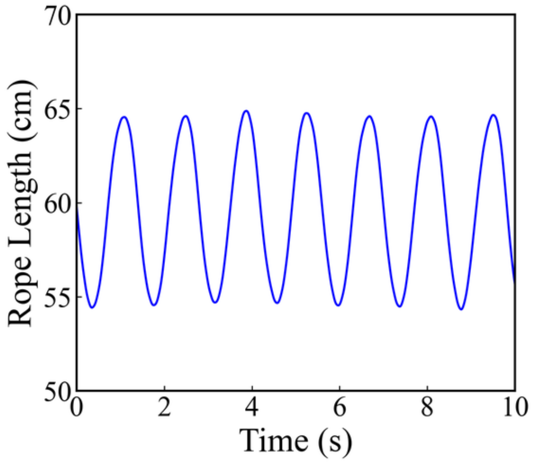

Rope Velocity

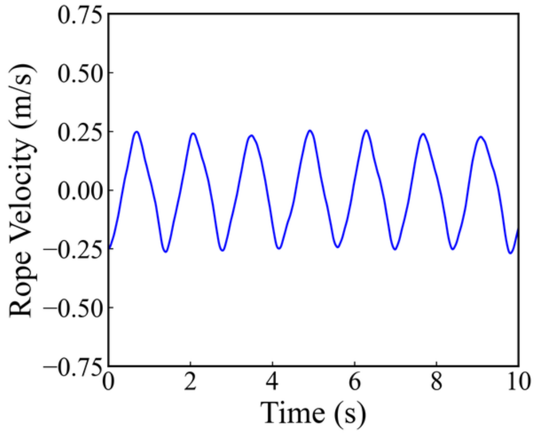

Regular wave: Period1.4s_Height13cm

Trajectory

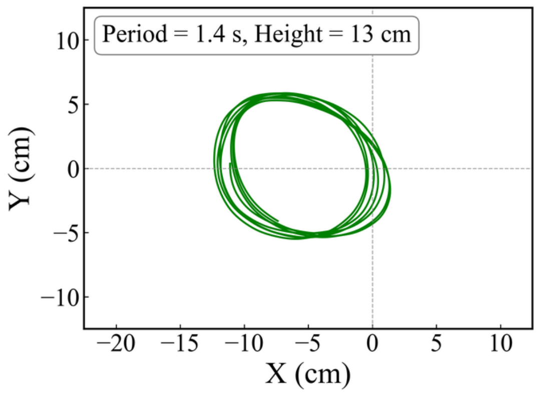

Displacement

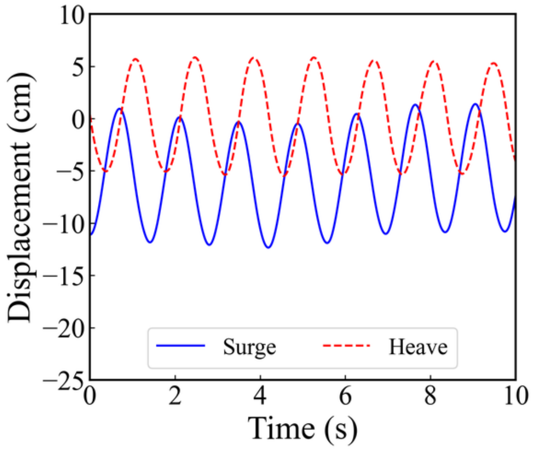

Velocity

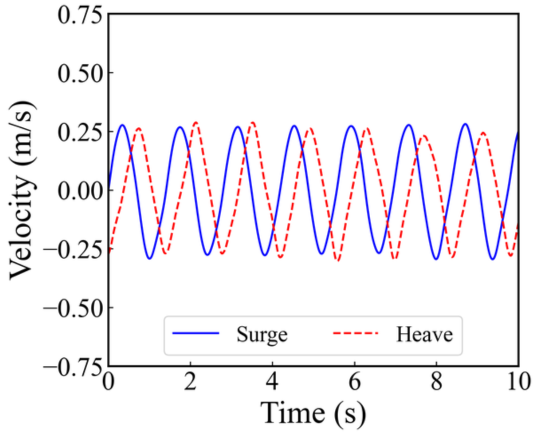

Rope Length

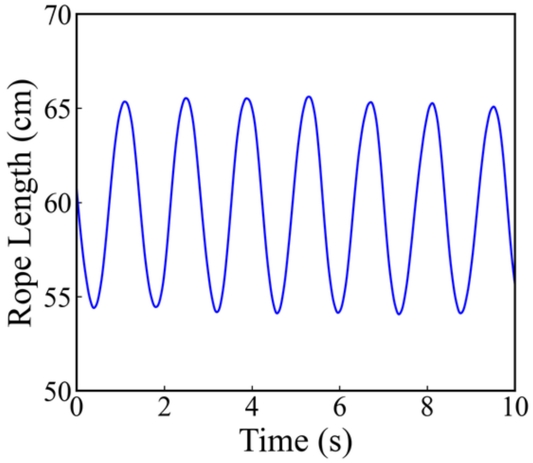

Rope Velocity

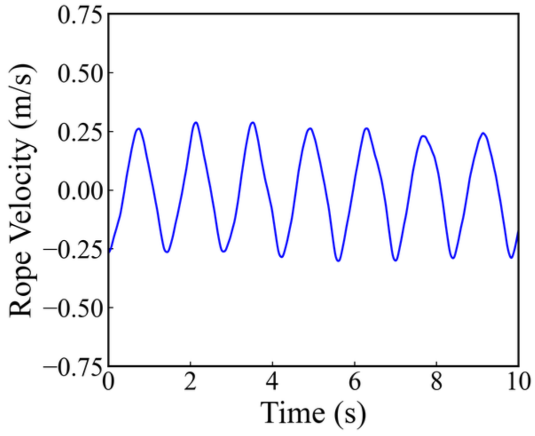

Regular wave: Period1.4s_Height14cm

Trajectory

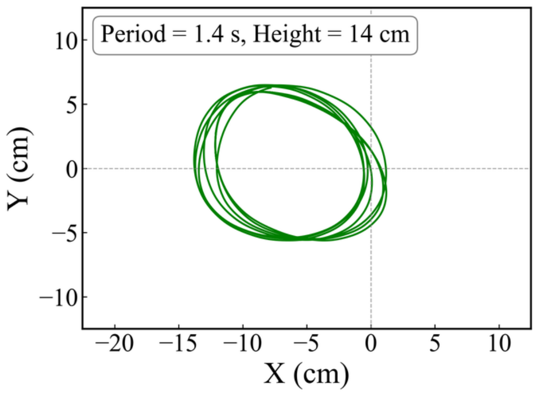

Displacement

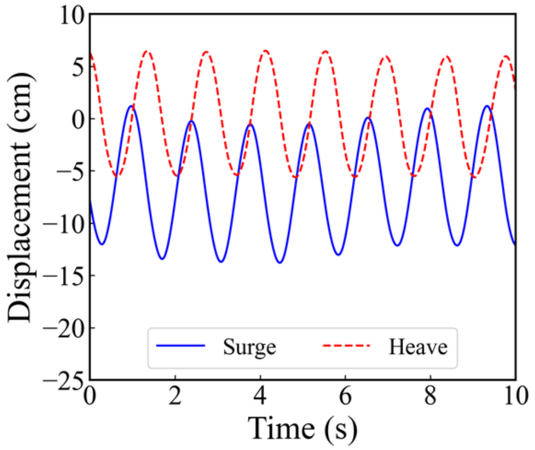

Velocity

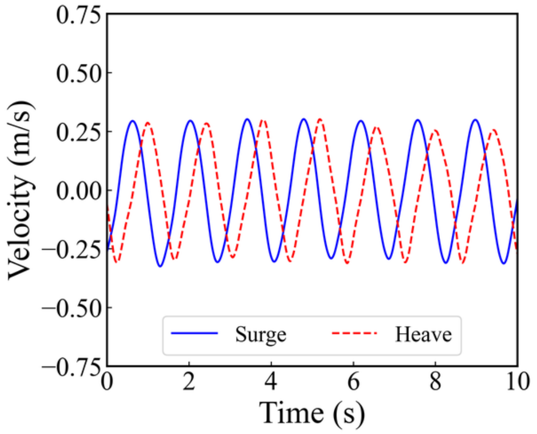

Rope Length

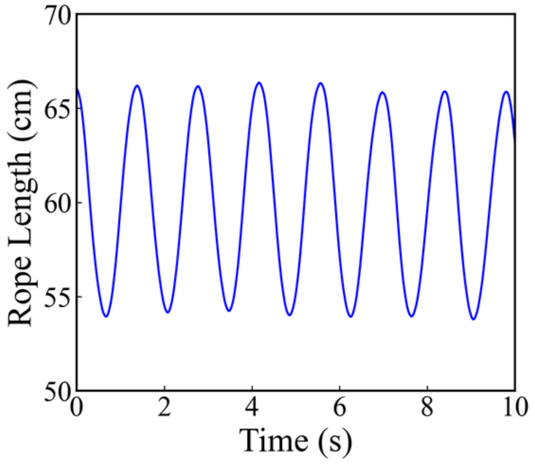

Rope Velocity

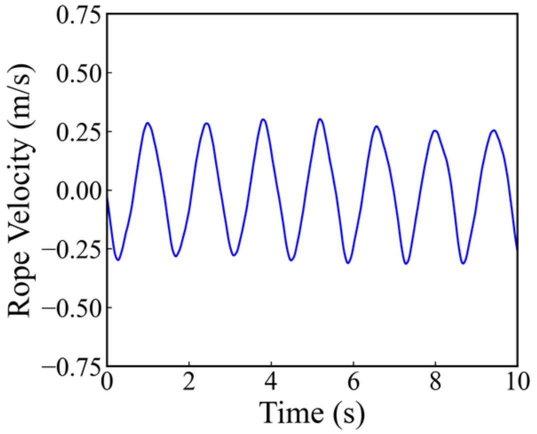

Regular wave: Period1.4s_Height15cm

Trajectory

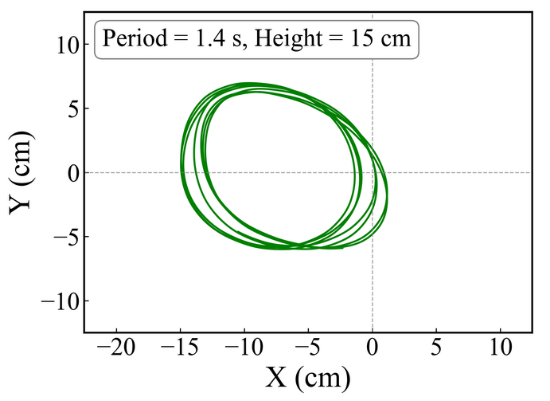

Displacement

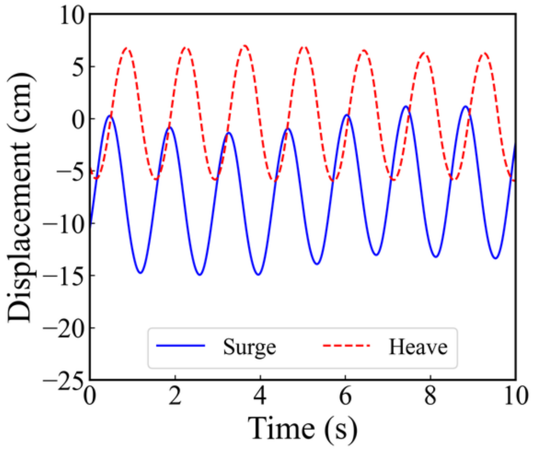

Velocity

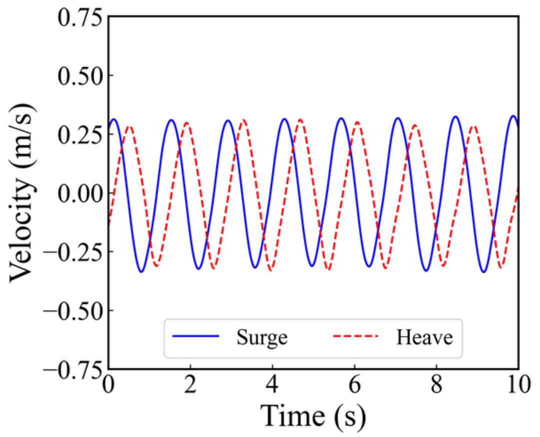

Rope Length

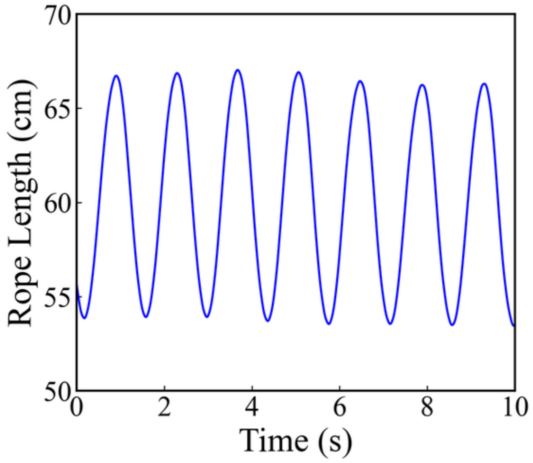

Rope Velocity

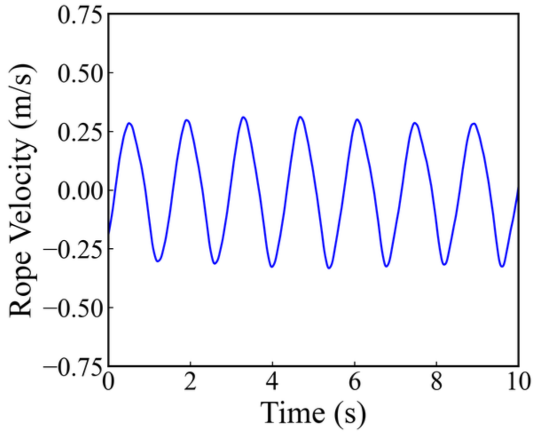

Regular wave: Period1.5s_Height5cm

Trajectory

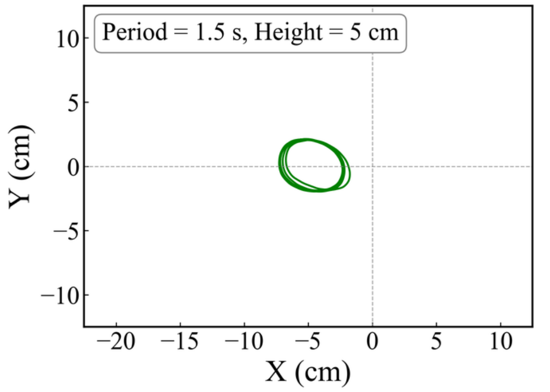

Displacement

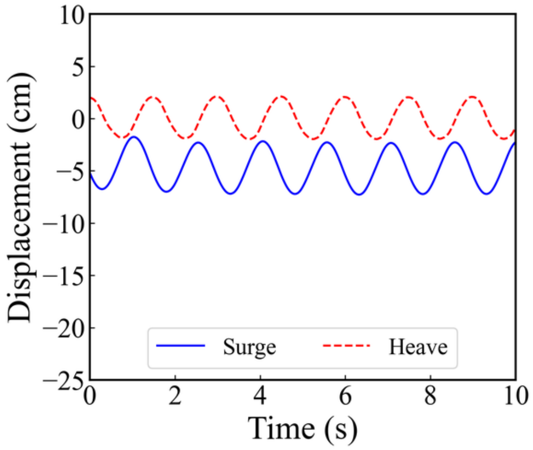

Velocity

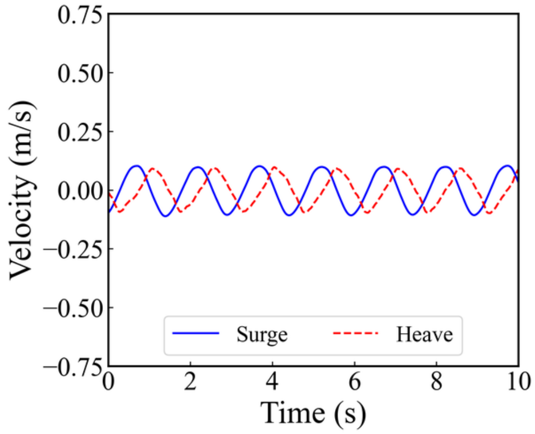

Rope Length

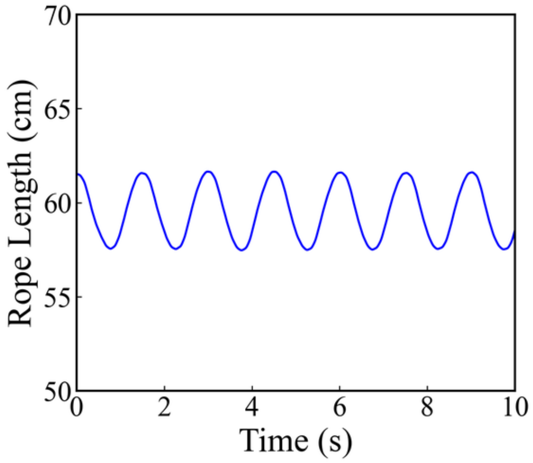

Rope Velocity

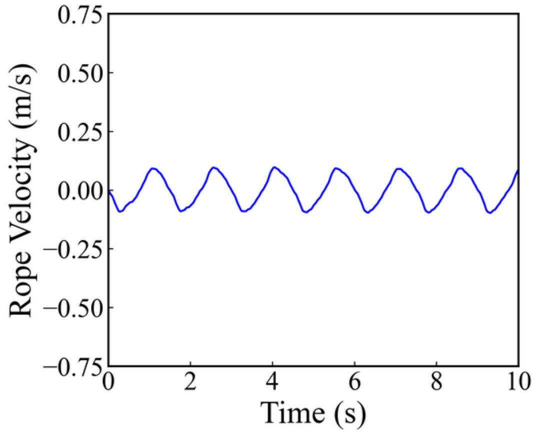

Regular wave: Period1.5s_Height6cm

Trajectory

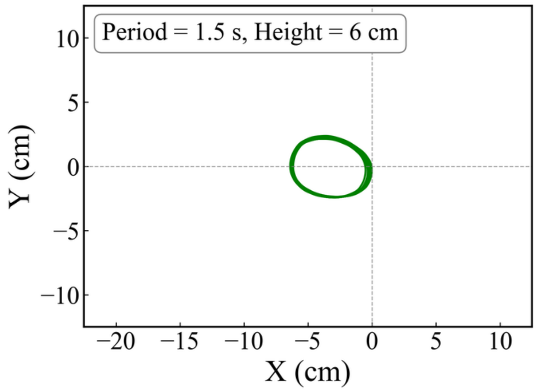

Displacement

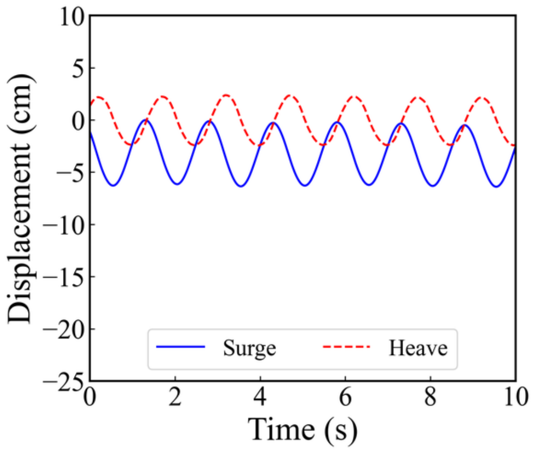

Velocity

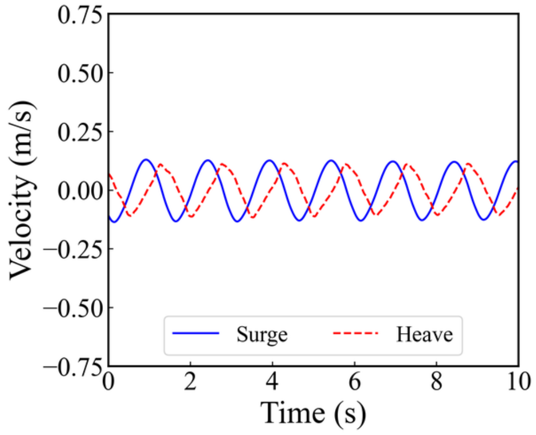

Rope Length

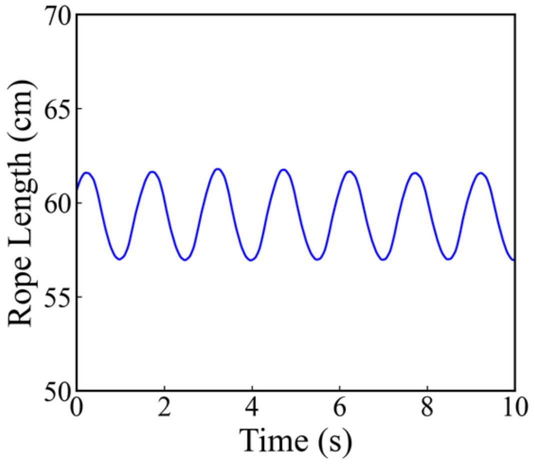

Rope Velocity

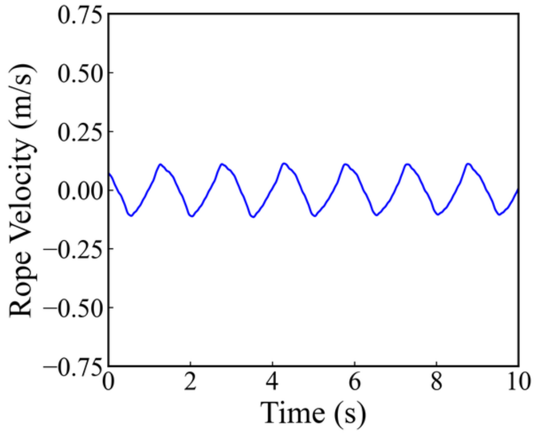

Regular wave: Period1.5s_Height7cm

Trajectory

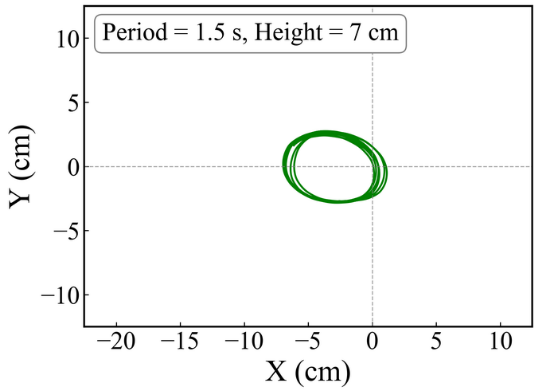

Displacement

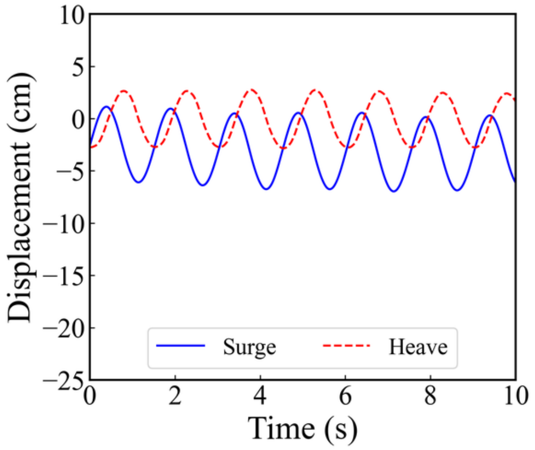

Velocity

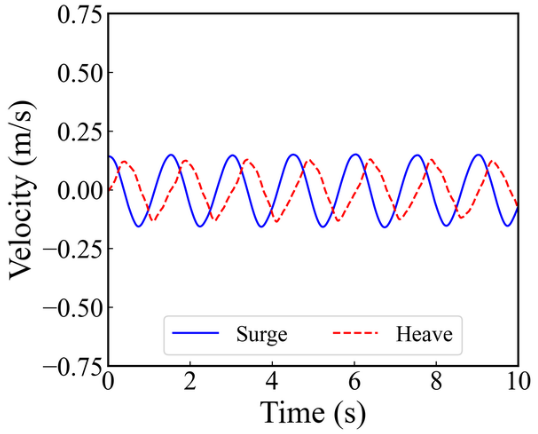

Rope Length

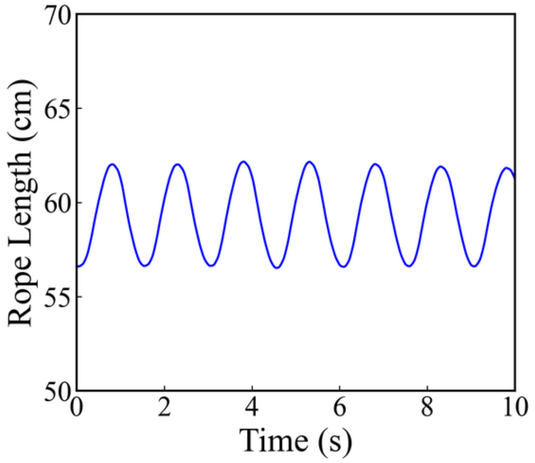

Rope Velocity

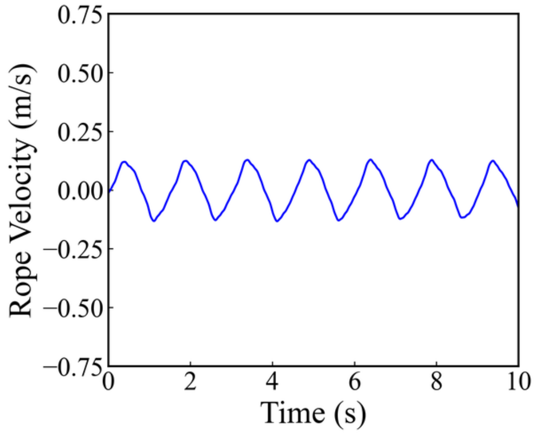

Regular wave: Period1.5s_Height8cm

Trajectory

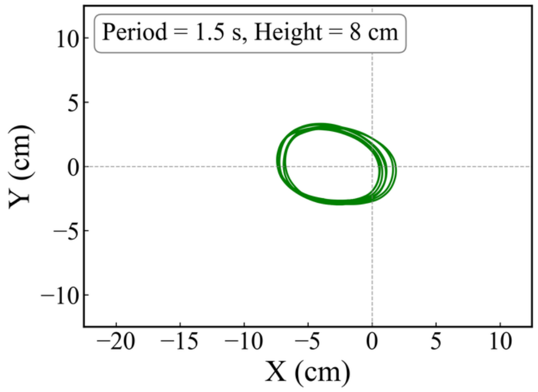

Displacement

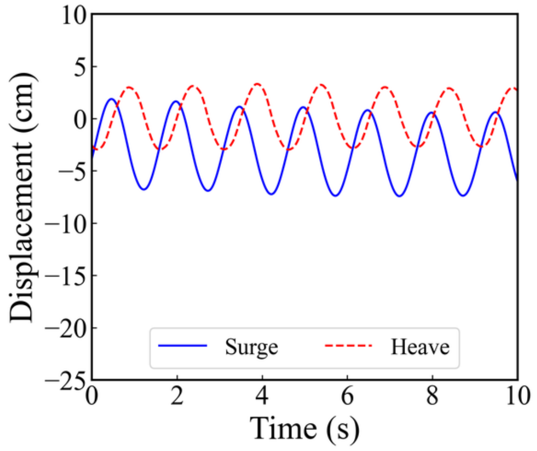

Velocity

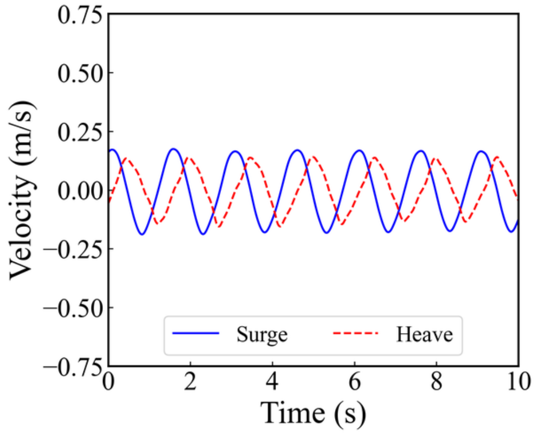

Rope Length

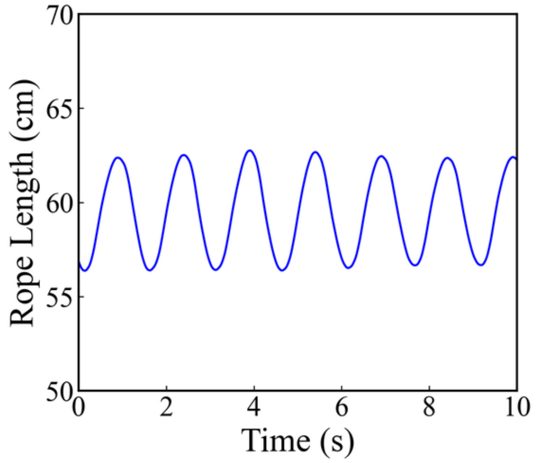

Rope Velocity

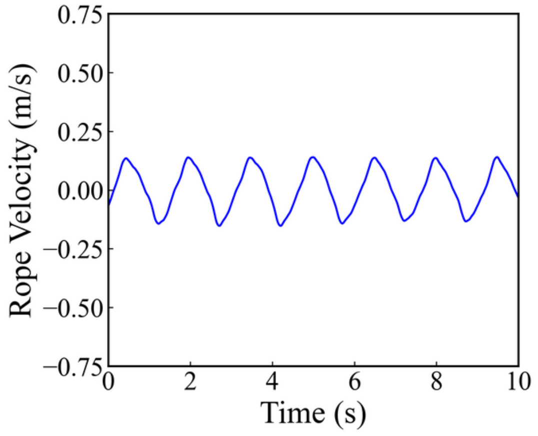

Regular wave: Period1.5s_Height9cm

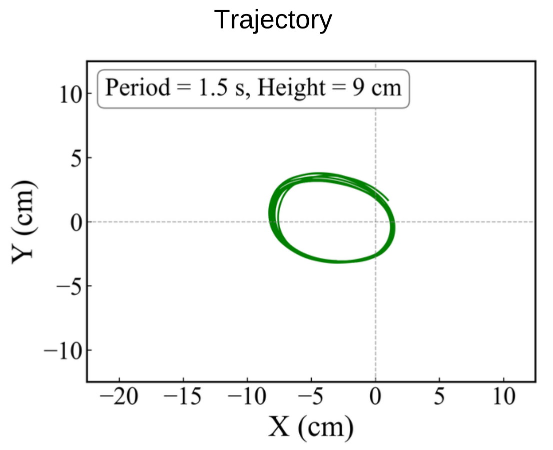

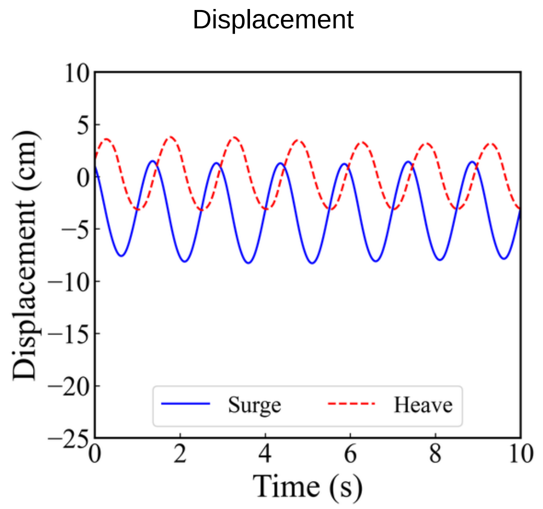

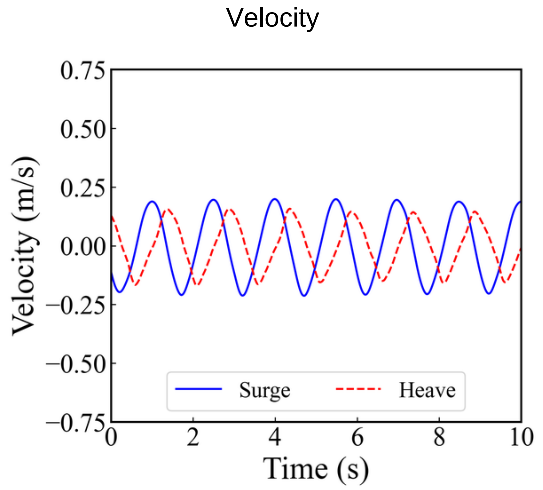

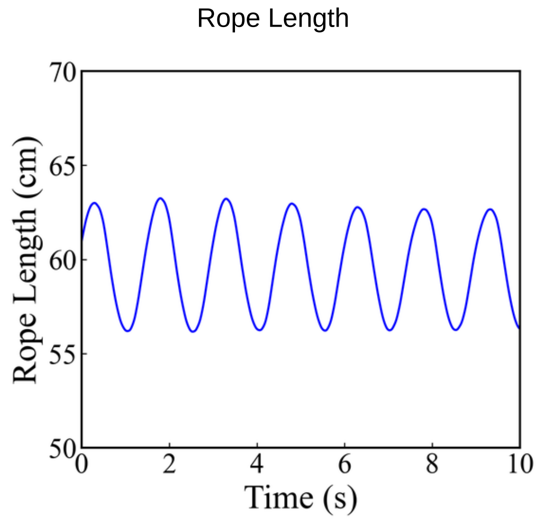

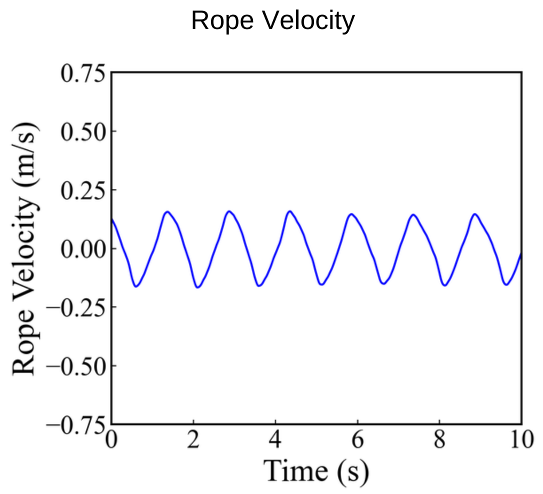

Regular wave: Period1.5s_Height10cm

Trajectory

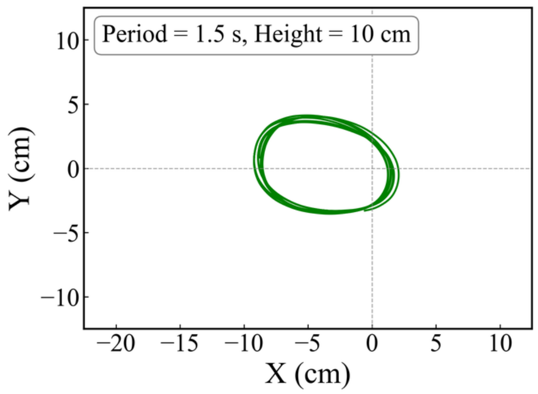

Displacement

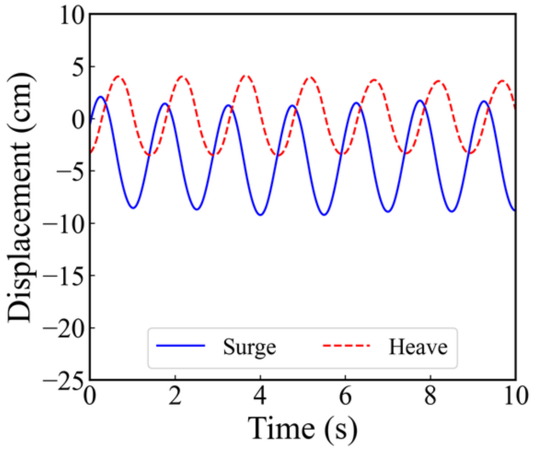

Velocity

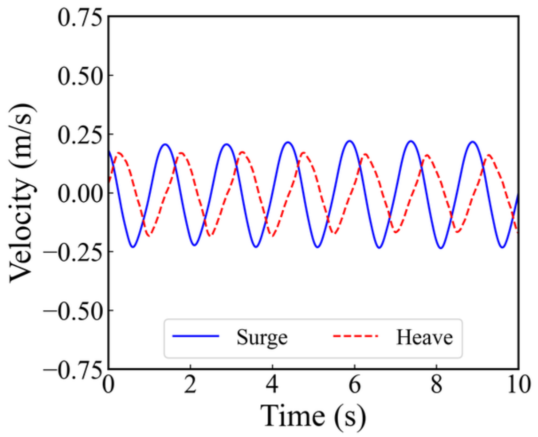

Rope Length

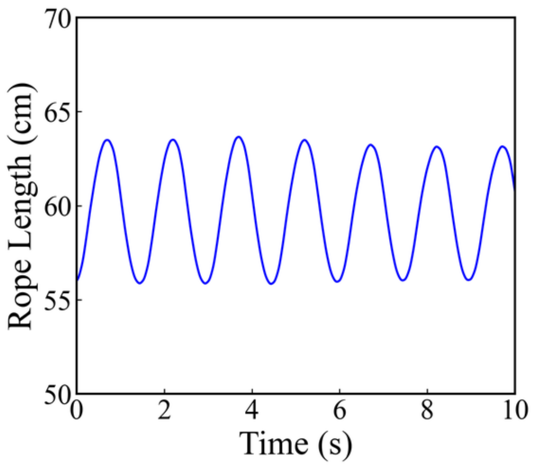

Rope Velocity

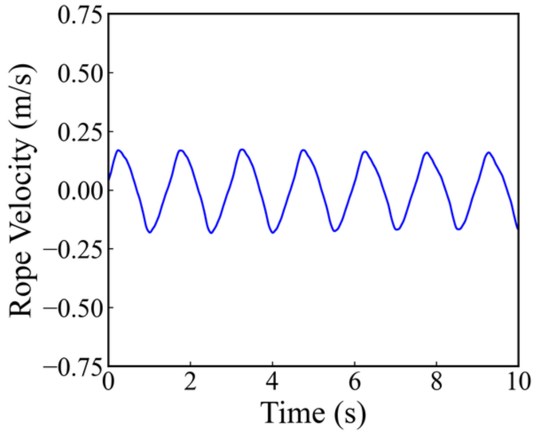

Regular wave: Period1.5s_Height11cm

Trajectory

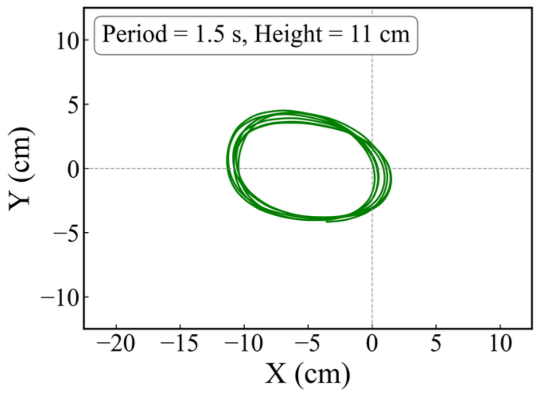

Displacement

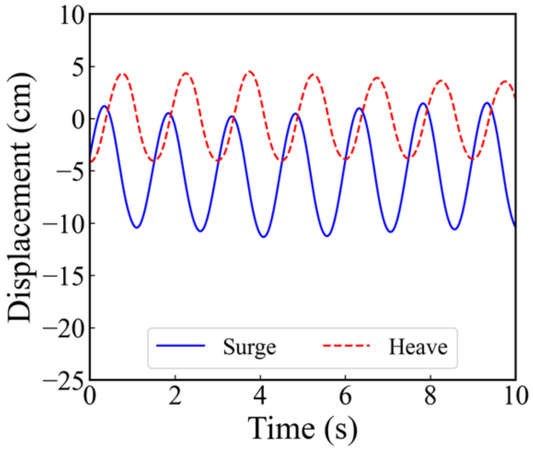

Velocity

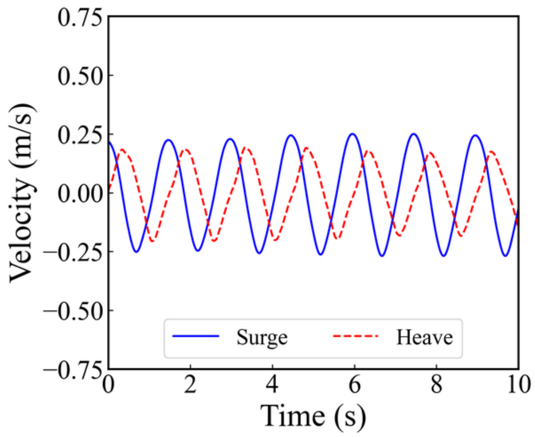

Rope Length

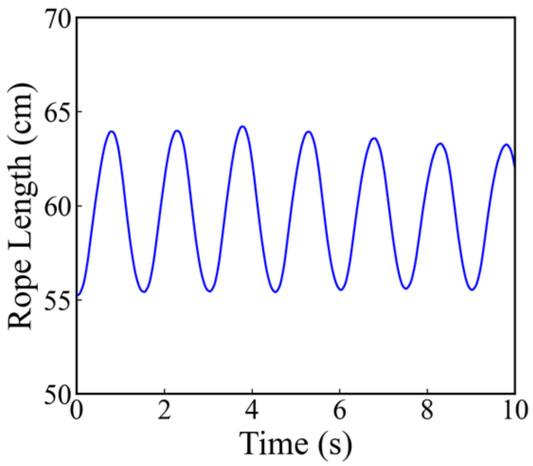

Rope Velocity

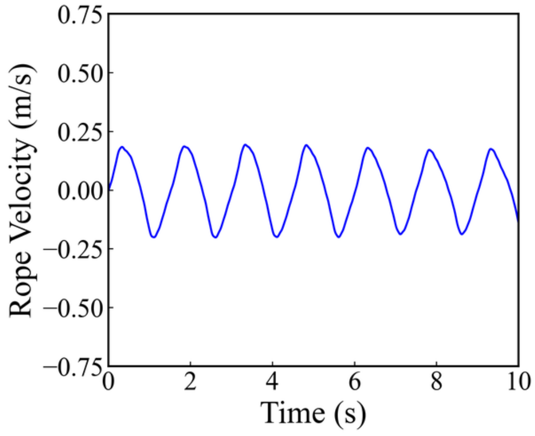

Regular wave: Period1.5s_Height12cm

Trajectory

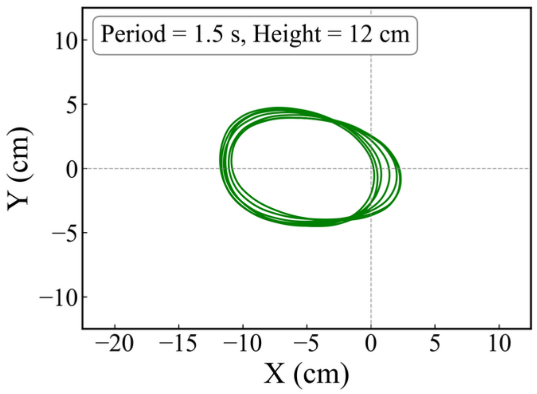

Displacement

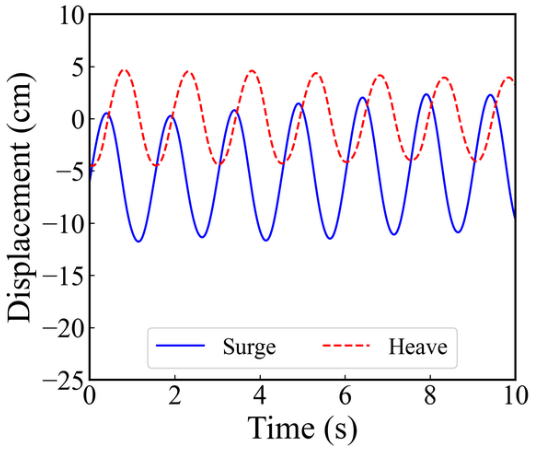

Velocity

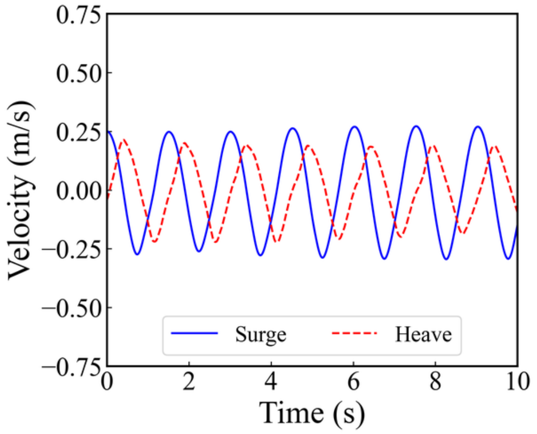

Rope Length

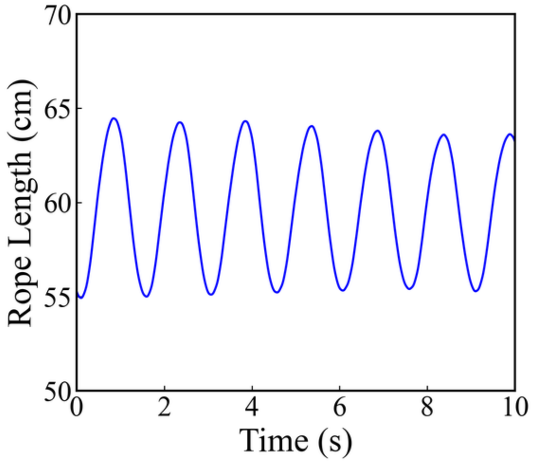

Rope Velocity

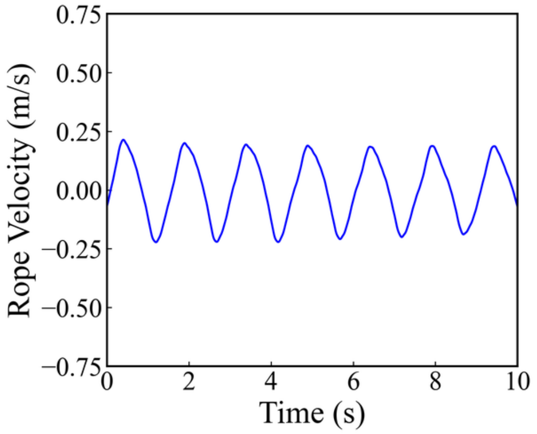

Regular wave: Period1.5s_Height13cm

Trajectory

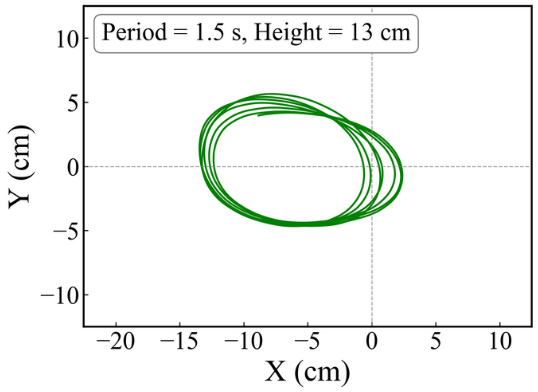

Displacement

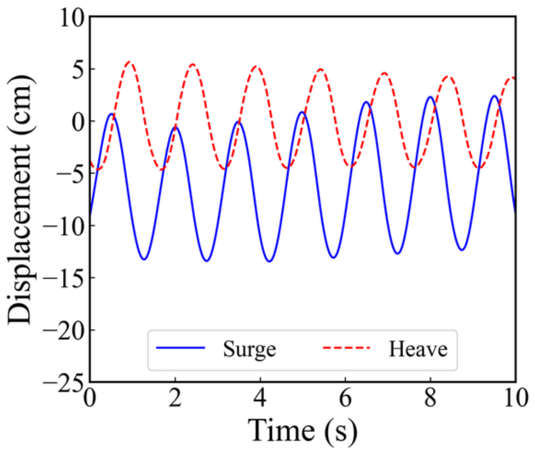

Velocity

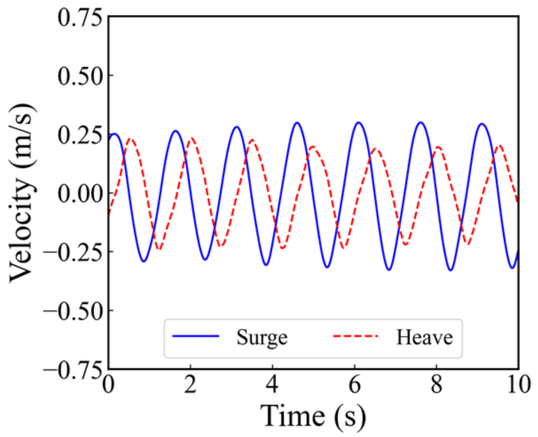

Rope Length

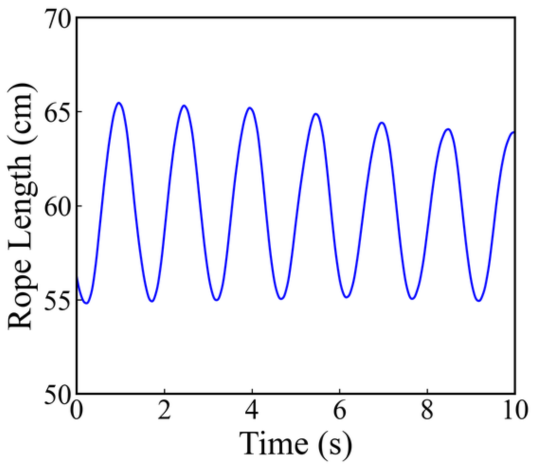

Rope Velocity

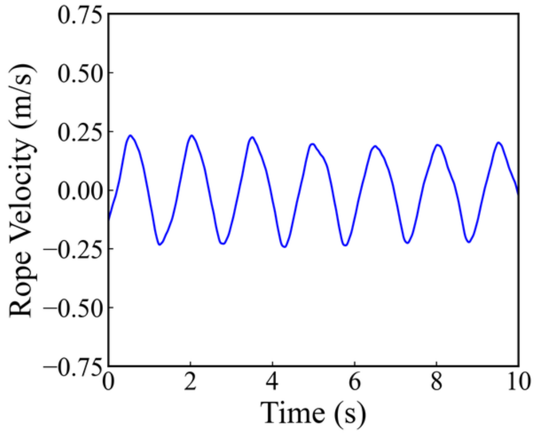

Regular wave: Period1.5s_Height14cm

Trajectory

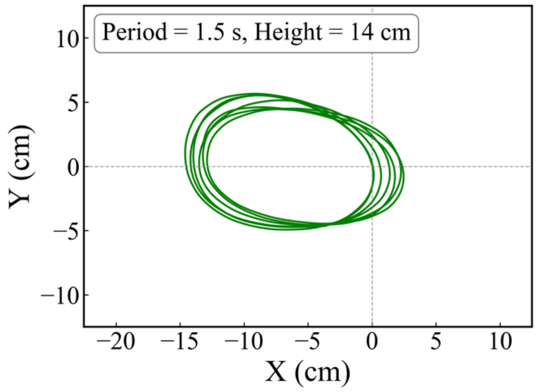

Displacement

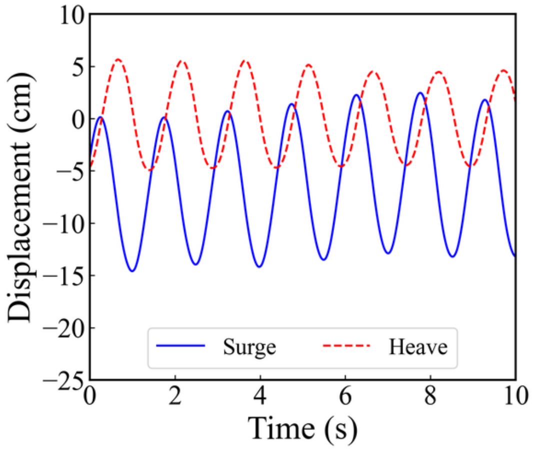

Velocity

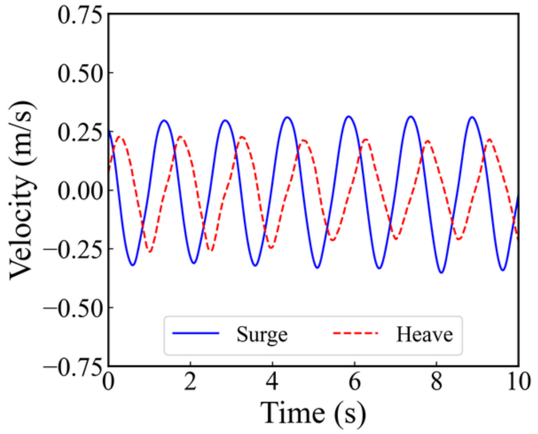

Rope Length

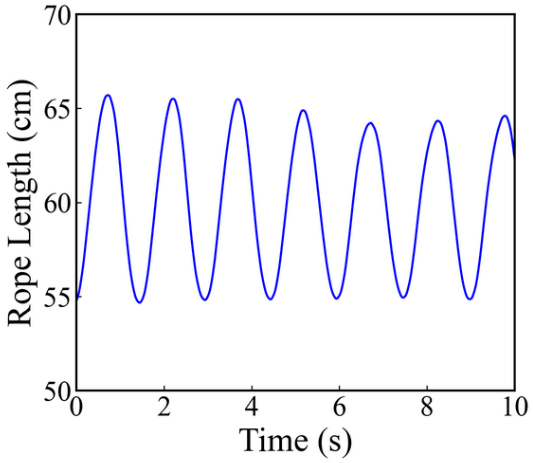

Rope Velocity

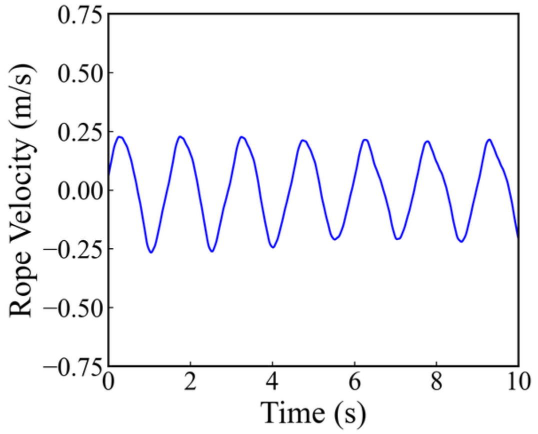

Regular wave: Period1.5s_Height15cm

Trajectory

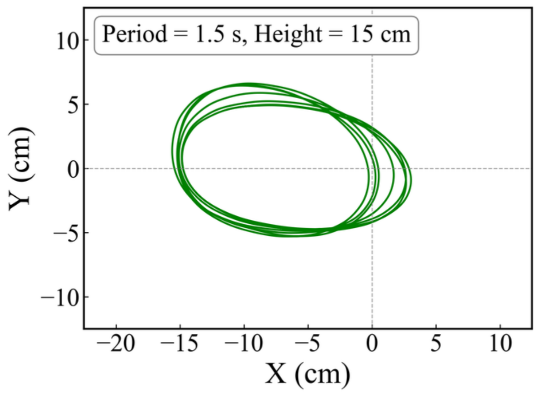

Displacement

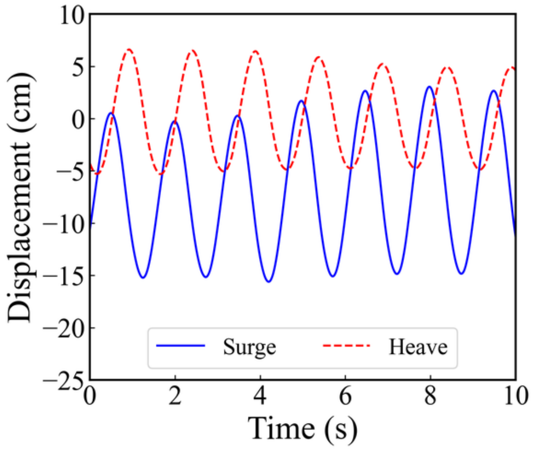

Velocity

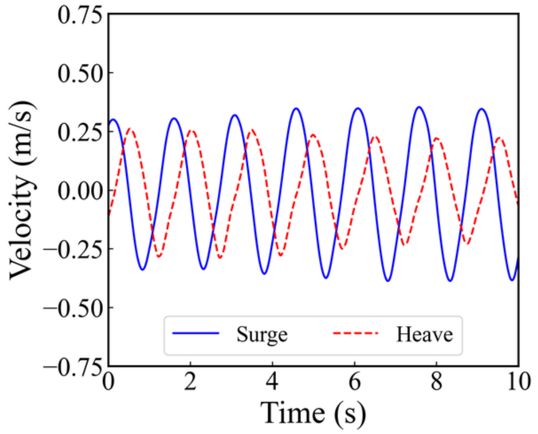

Rope Length

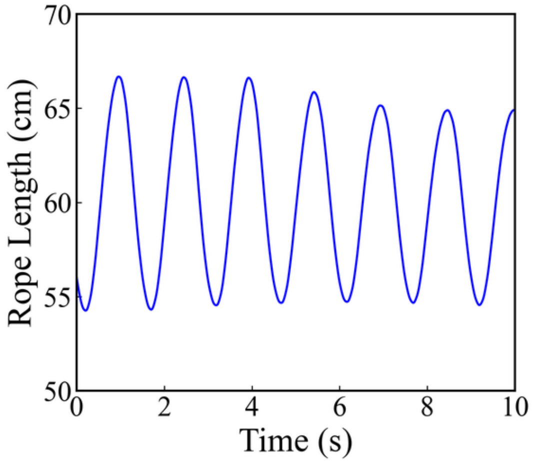

Rope Velocity

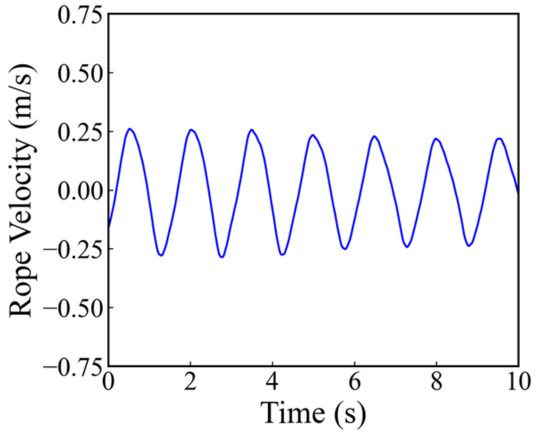

Regular wave: Period1.6s_Height5cm

Trajectory

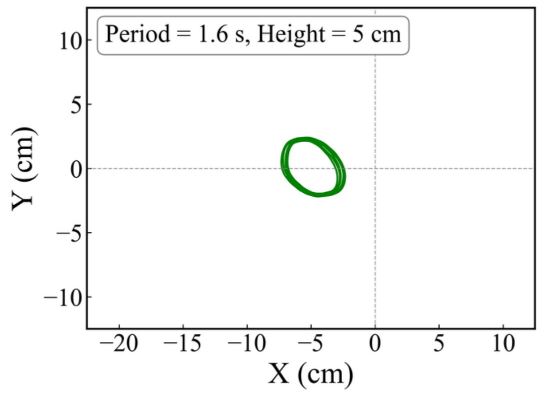

Displacement

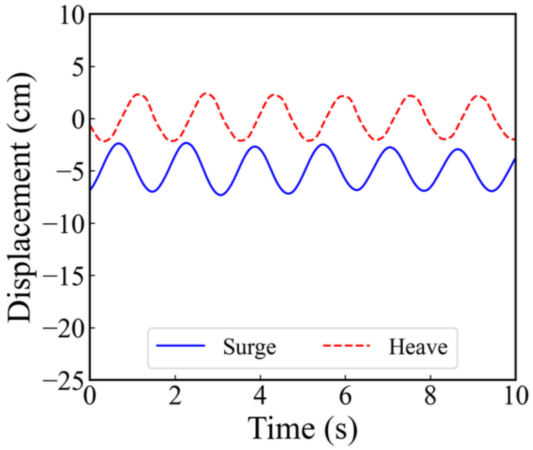

Velocity

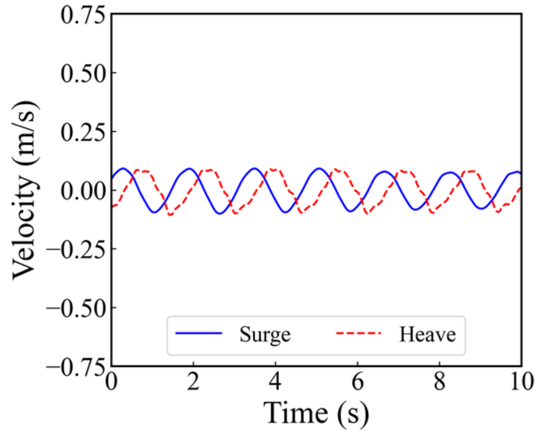

Rope Length

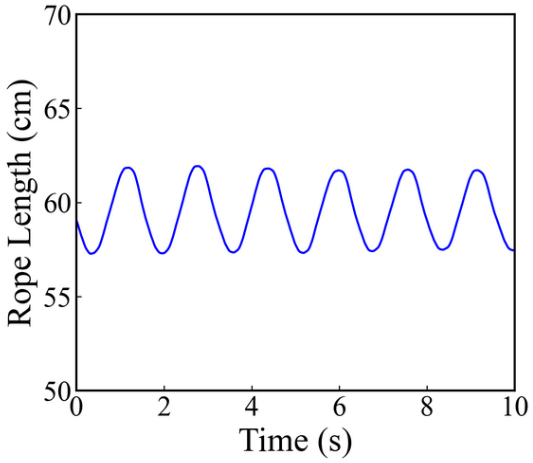

Rope Velocity

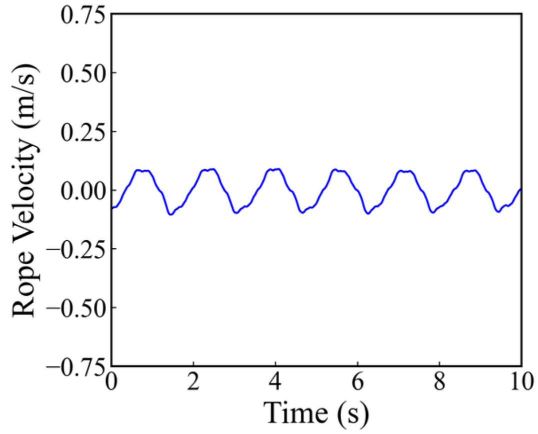

Regular wave: Period1.6s_Height6cm

Trajectory

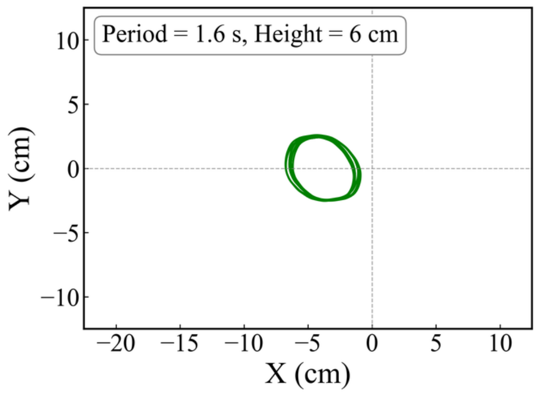

Displacement

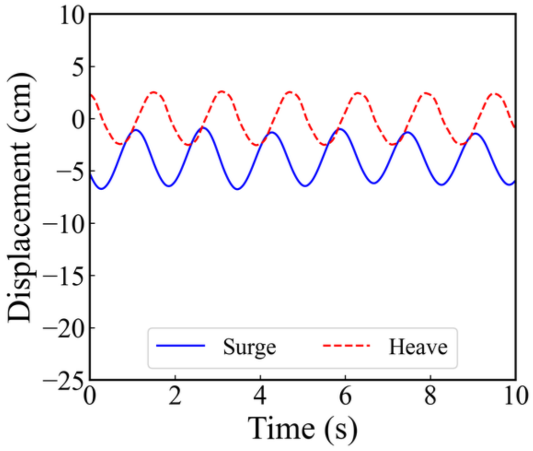

Velocity

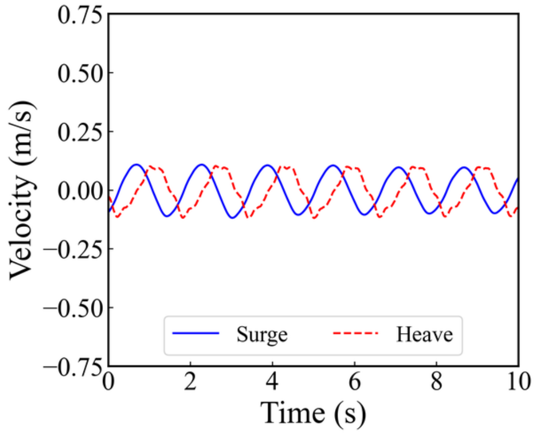

Rope Length

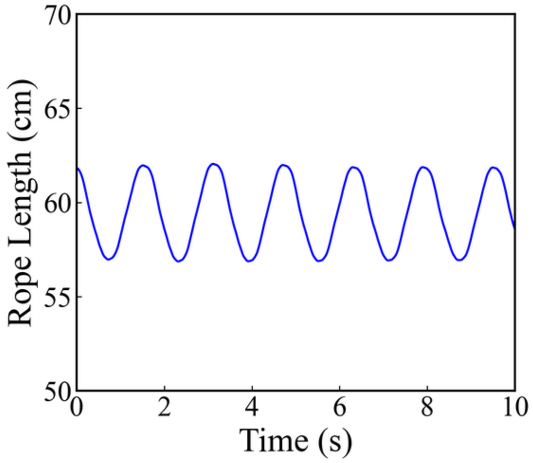

Rope Velocity

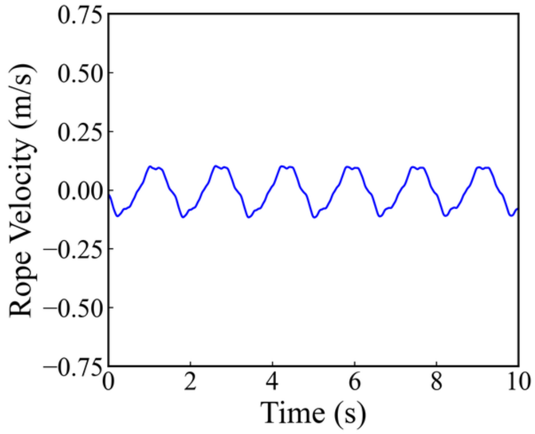

Regular wave: Period1.6s_Height7cm

Trajectory

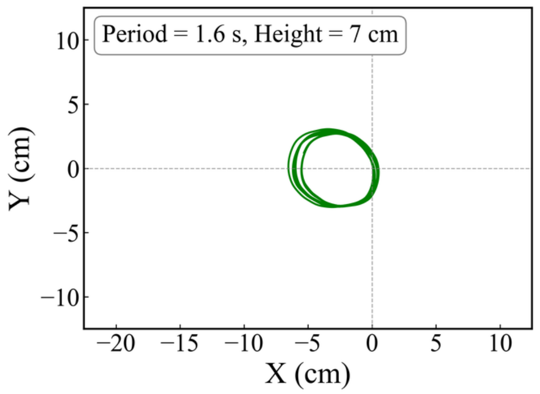

Displacement

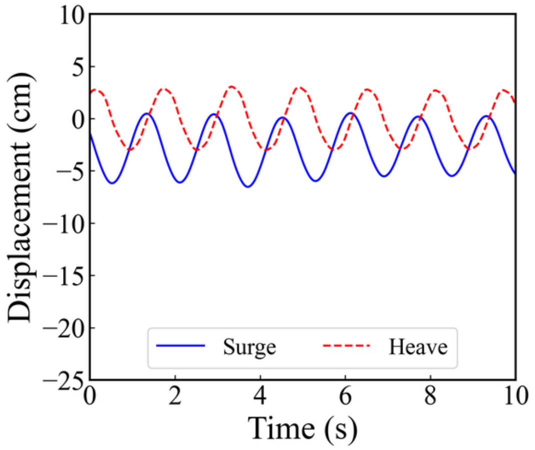

Velocity

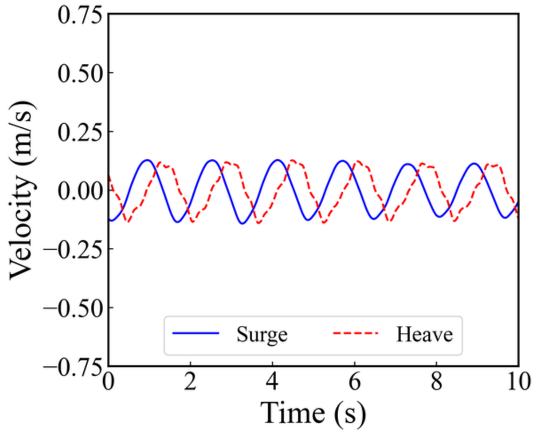

Rope Length

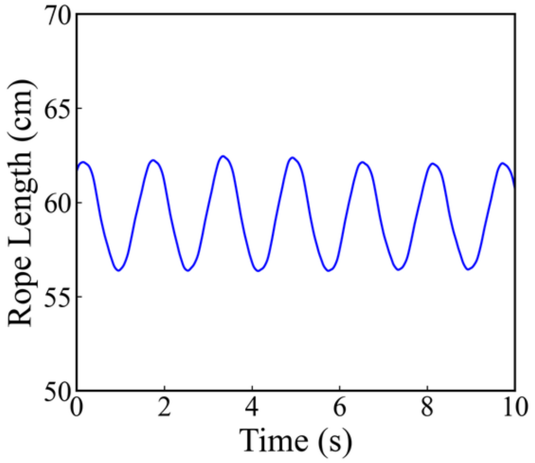

Rope Velocity

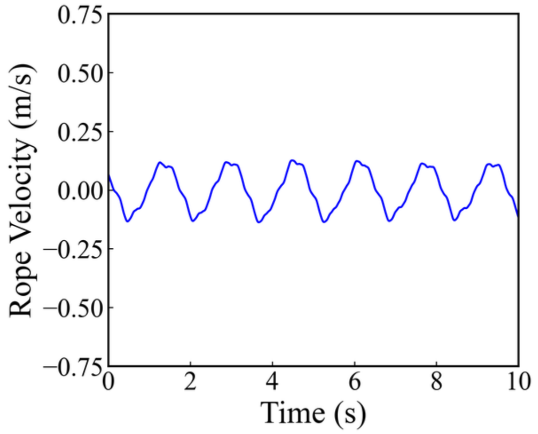

Regular wave: Period1.6s_Height8cm

Trajectory

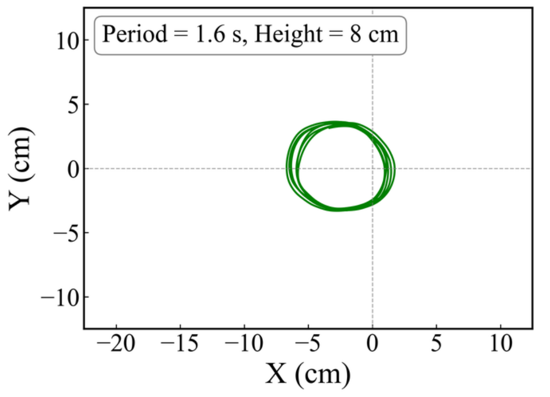

Displacement

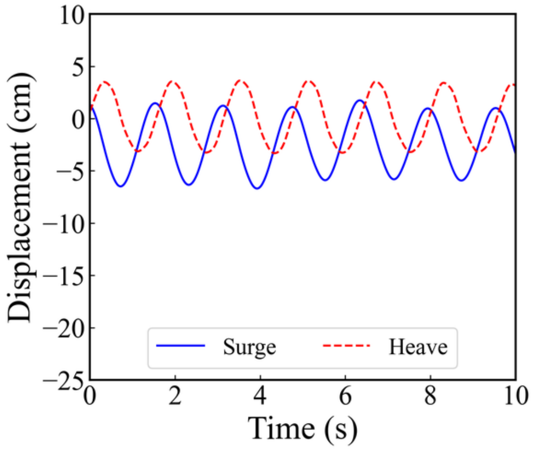

Velocity

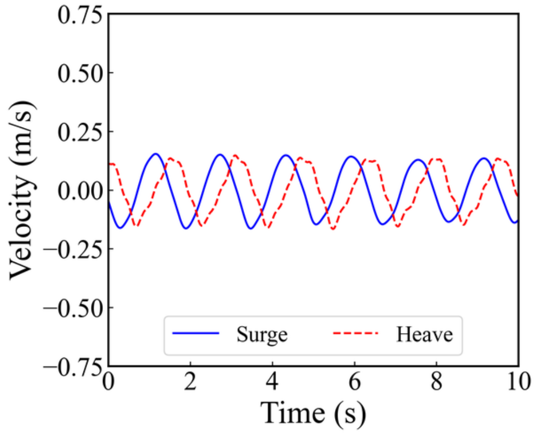

Rope Length

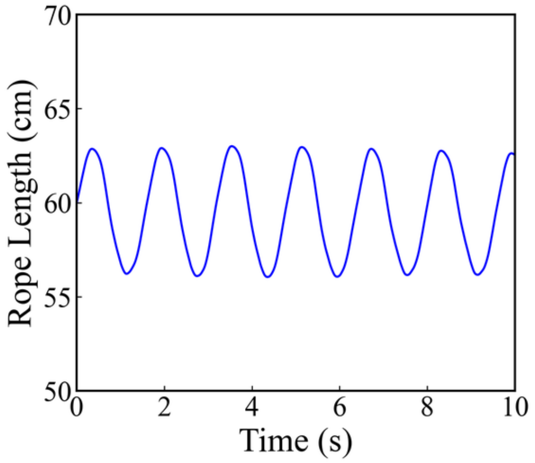

Rope Velocity

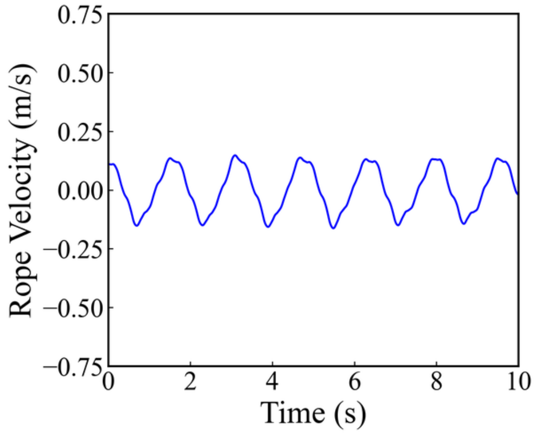

Regular wave: Period1.6s_Height9cm

Trajectory

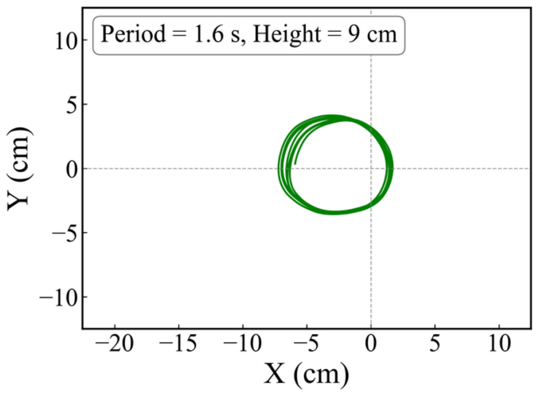

Displacement

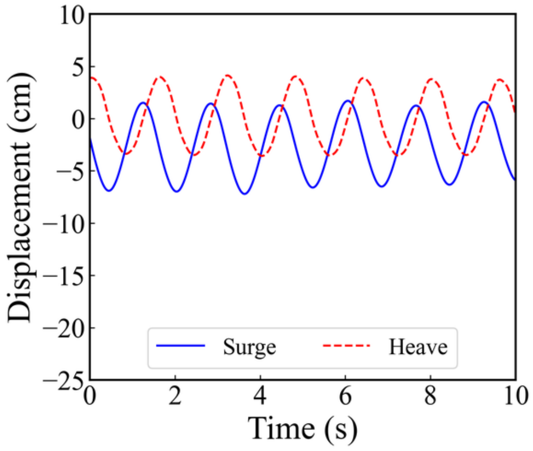

Velocity

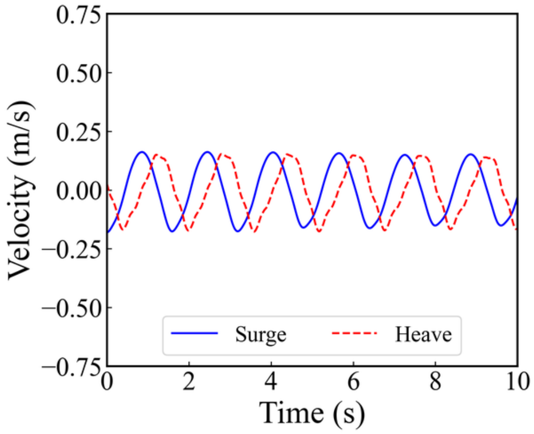

Rope Length

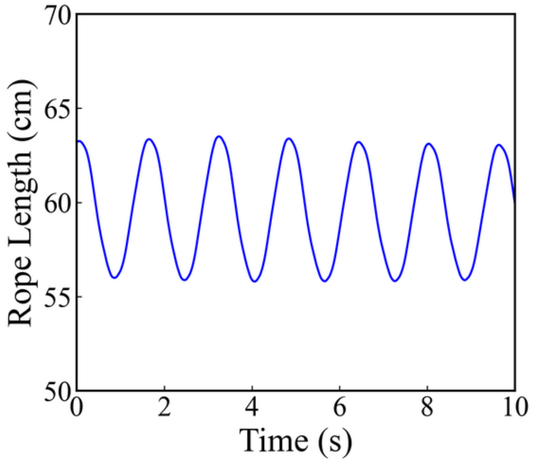

Rope Velocity

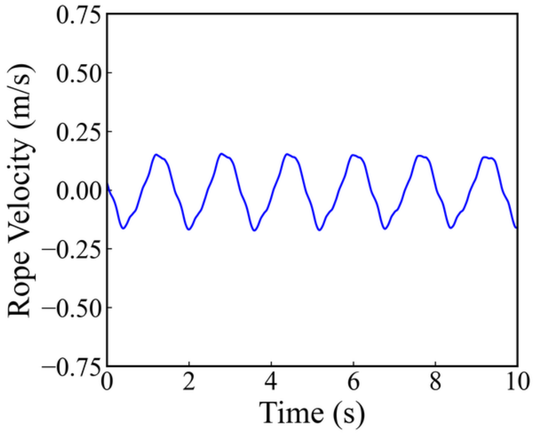

Regular wave: Period1.6s_Height10cm

Trajectory

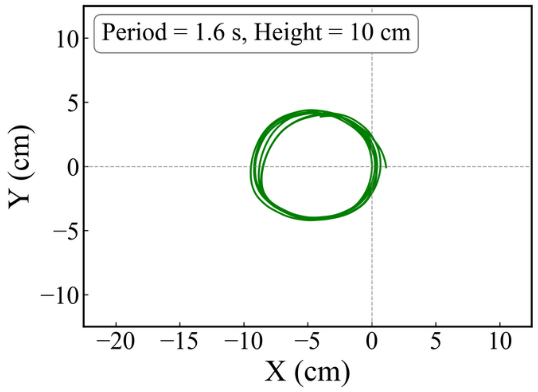

Displacement

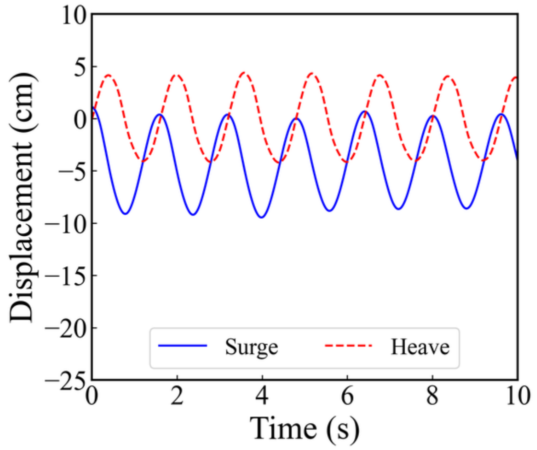

Velocity

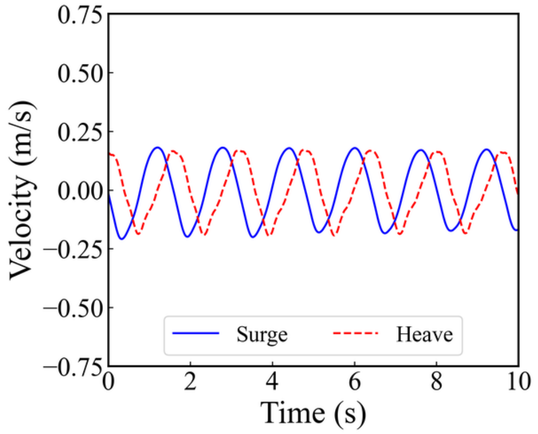

Rope Length

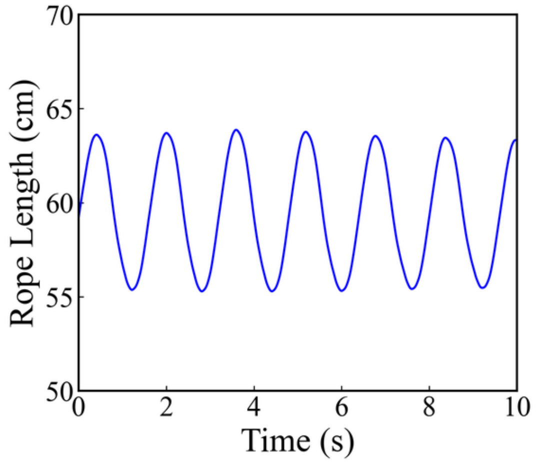

Rope Velocity

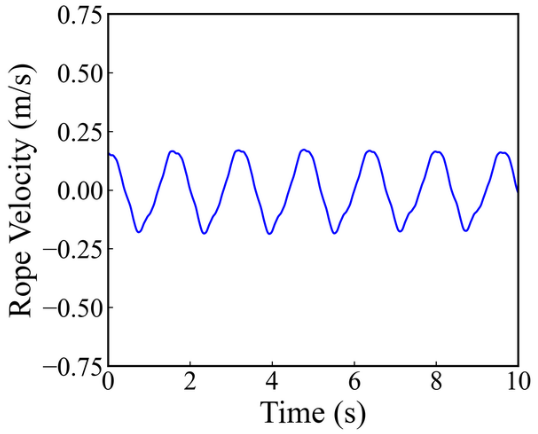

Regular wave: Period1.6s_Height11cm

Trajectory

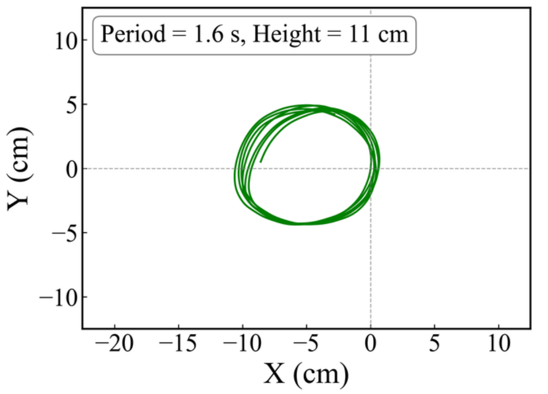

Displacement

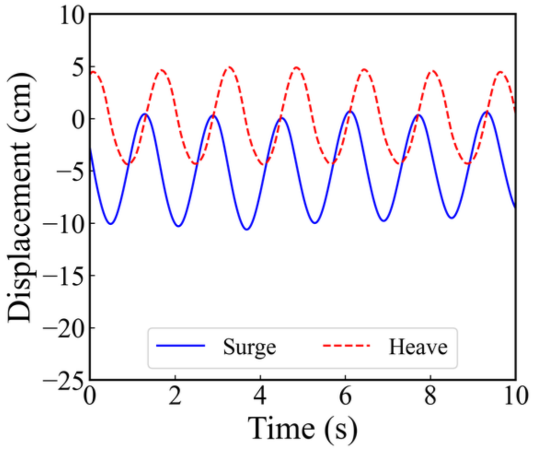

Velocity

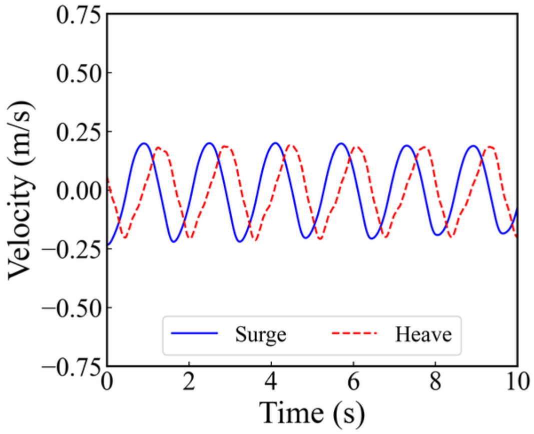

Rope Length

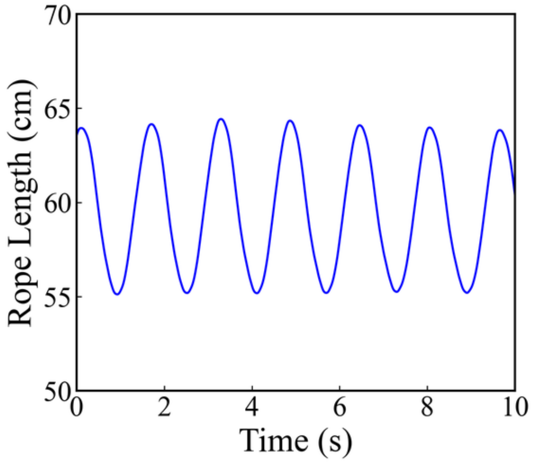

Rope Velocity

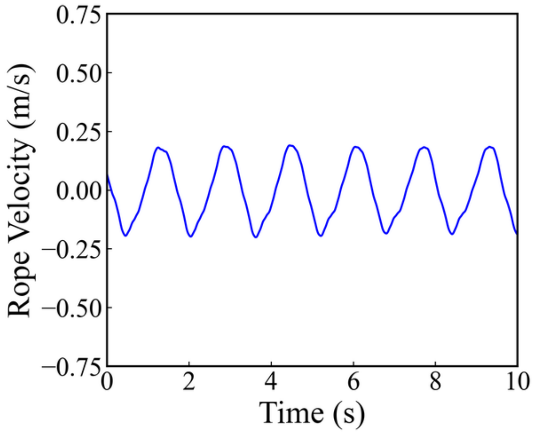

Regular wave: Period1.6s_Height12cm

Trajectory

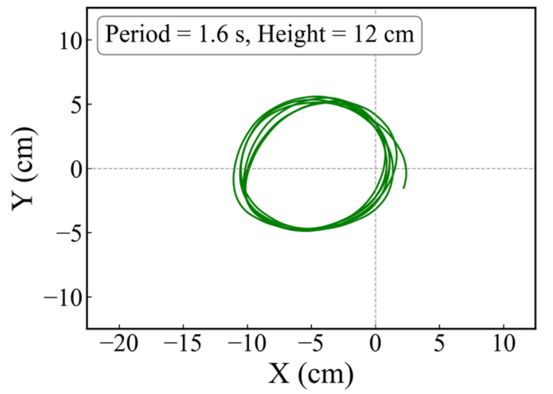

Displacement

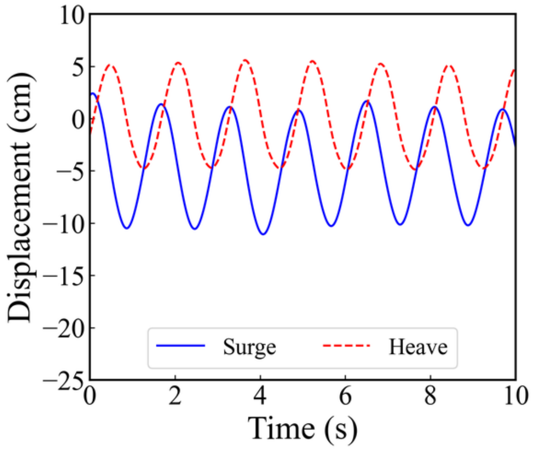

Velocity

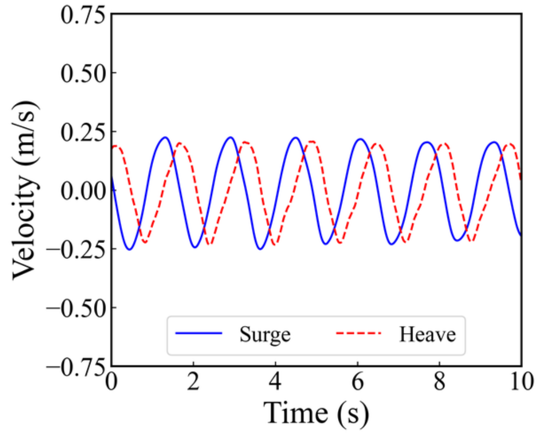

Rope Length

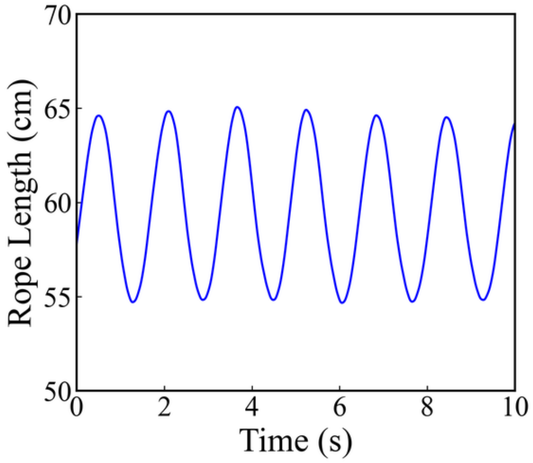

Rope Velocity

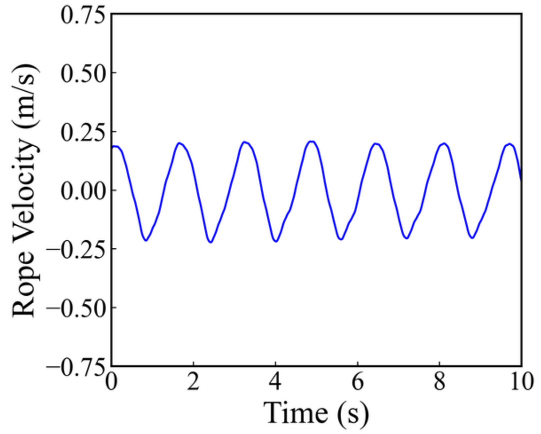

Regular wave: Period1.6s_Height13cm

Trajectory

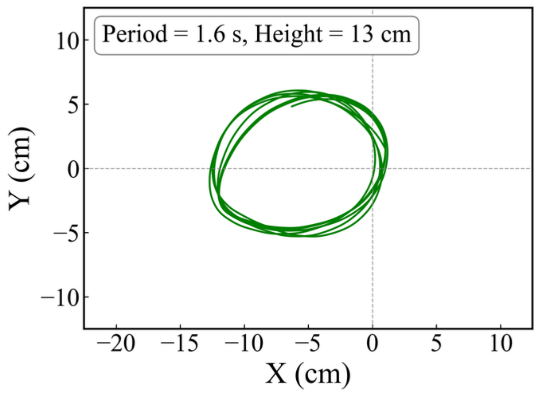

Displacement

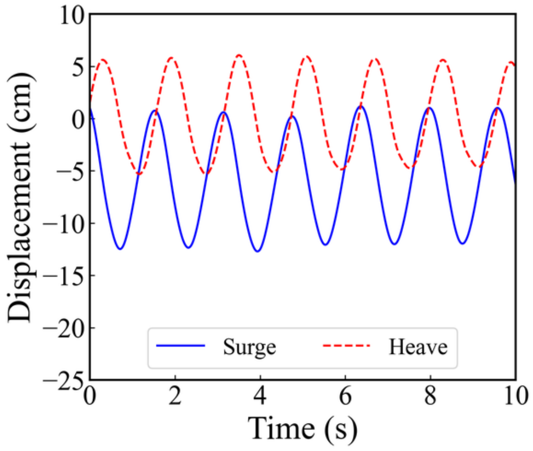

Velocity

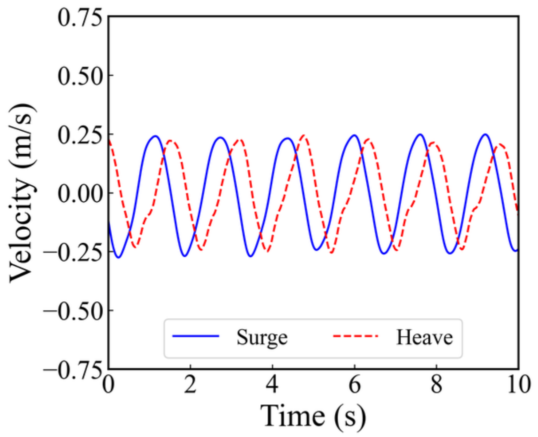

Rope Length

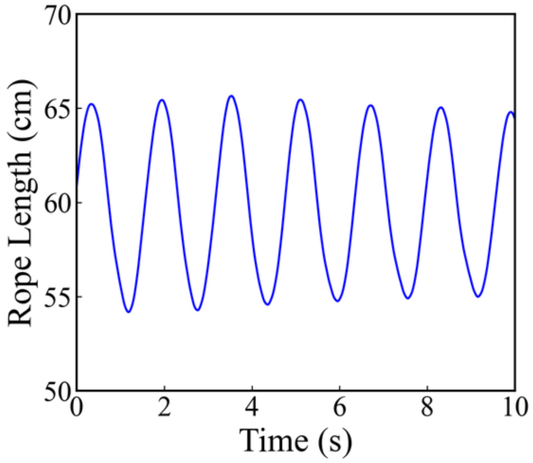

Rope Velocity

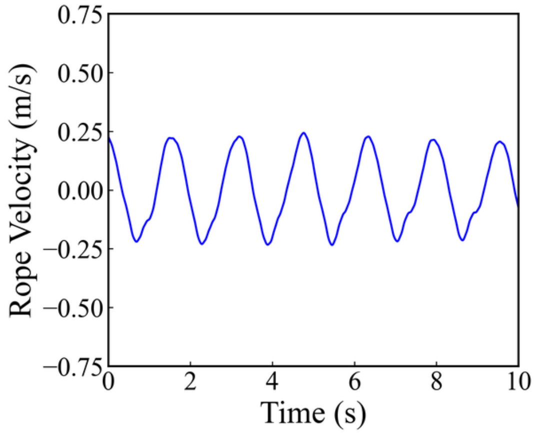

Regular wave: Period1.6s_Height14cm

Trajectory

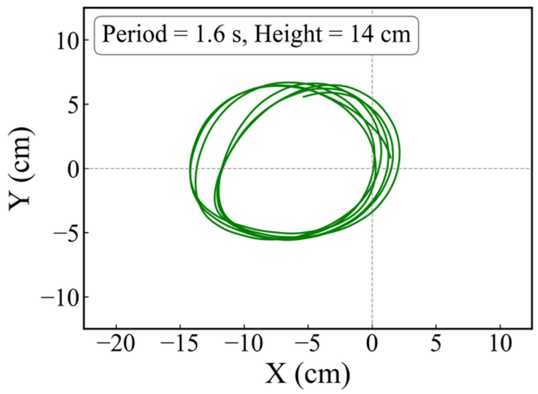

Displacement

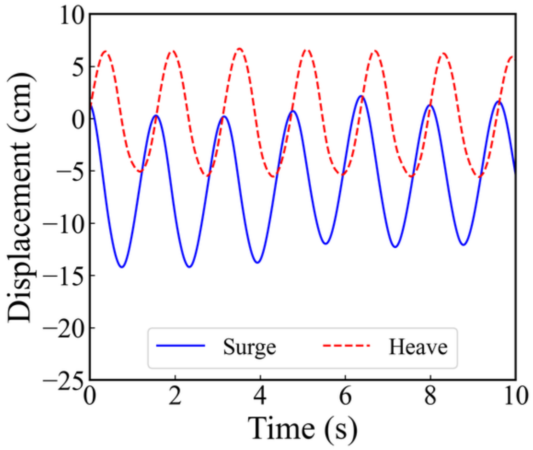

Velocity

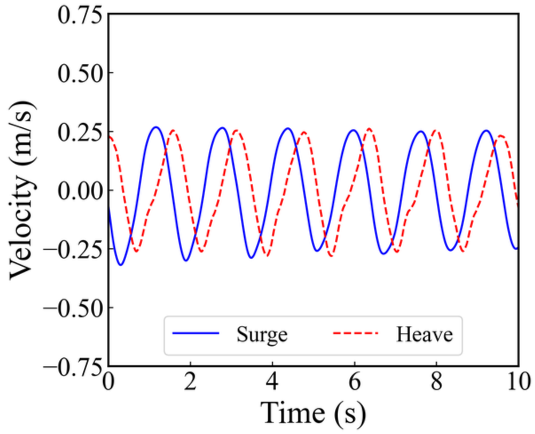

Rope Length

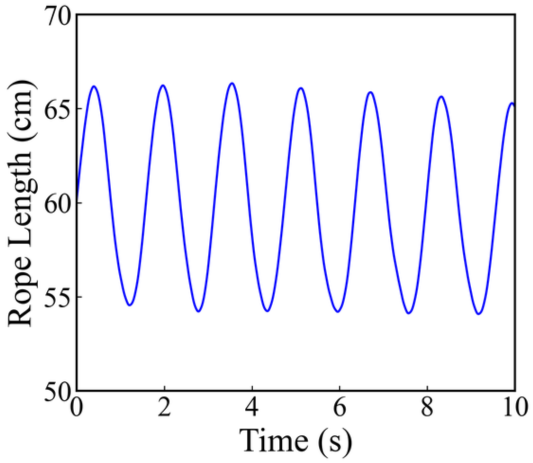

Rope Velocity

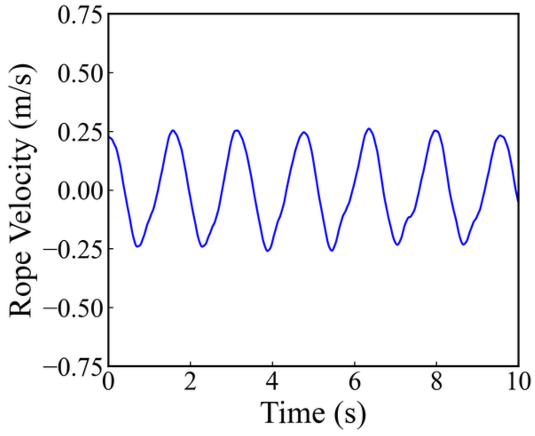

Regular wave: Period1.6s_Height15cm

Trajectory

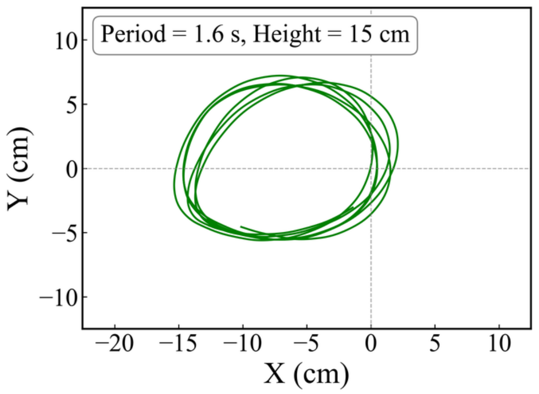

Displacement

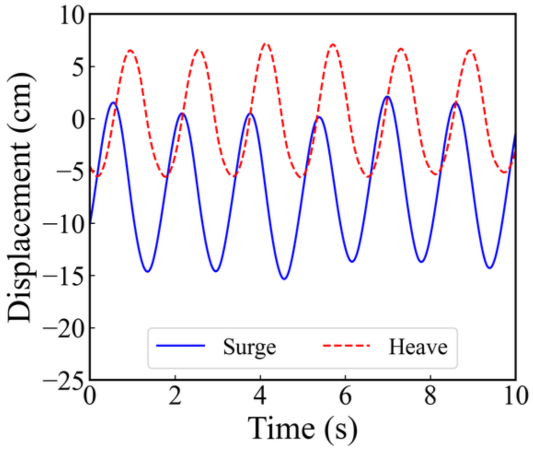

Velocity

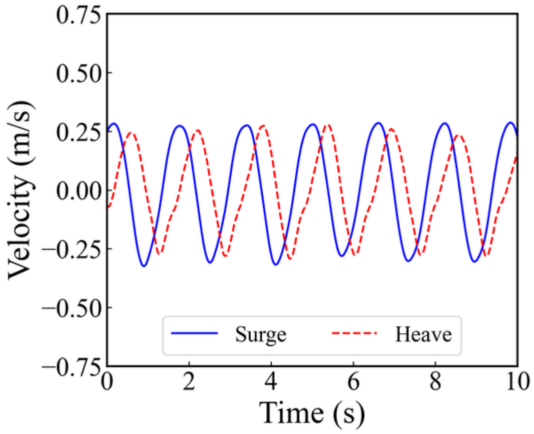

Rope Length

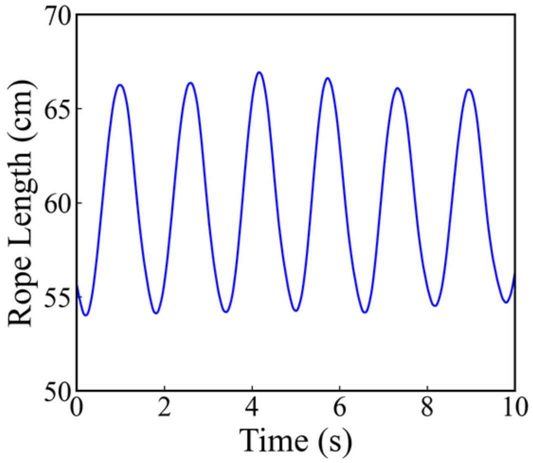

Rope Velocity

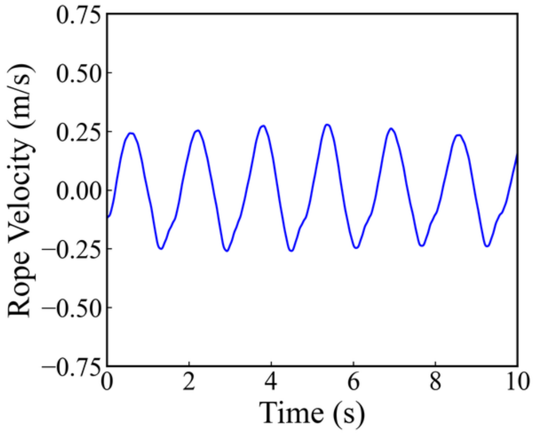

Regular wave: Period1.7s_Height5cm

Trajectory

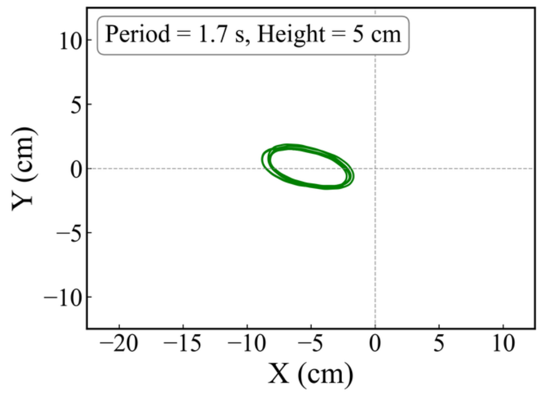

Displacement

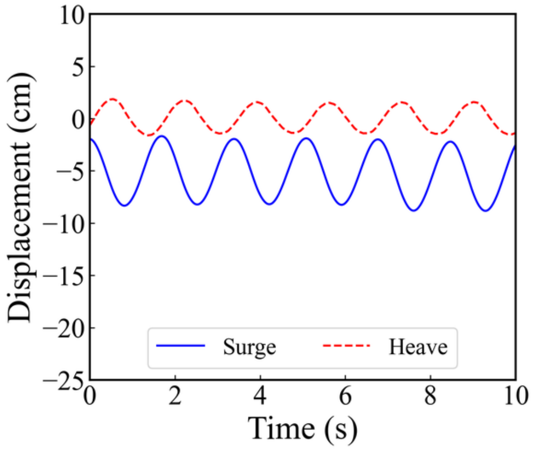

Velocity

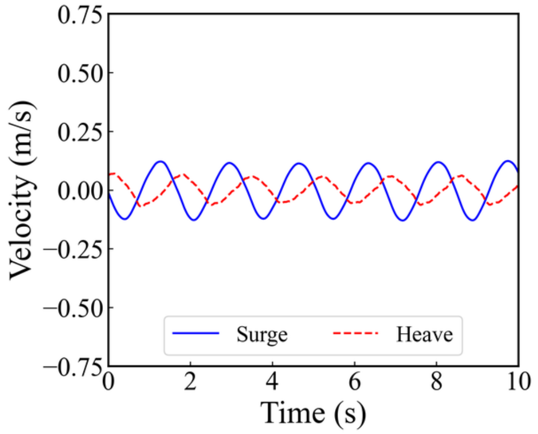

Rope Length

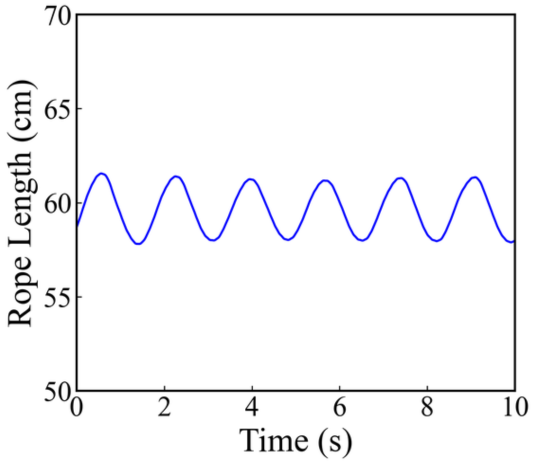

Rope Velocity

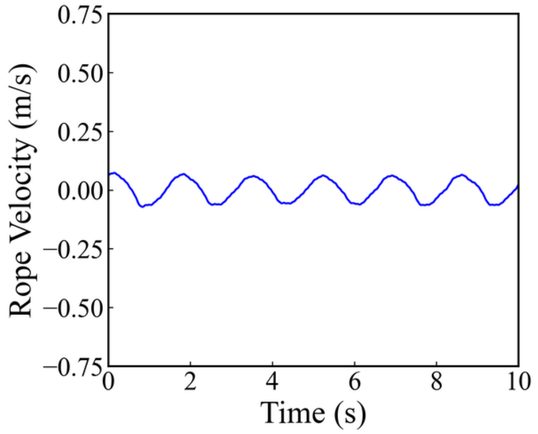

Regular wave: Period1.7s_Height6cm

Trajectory

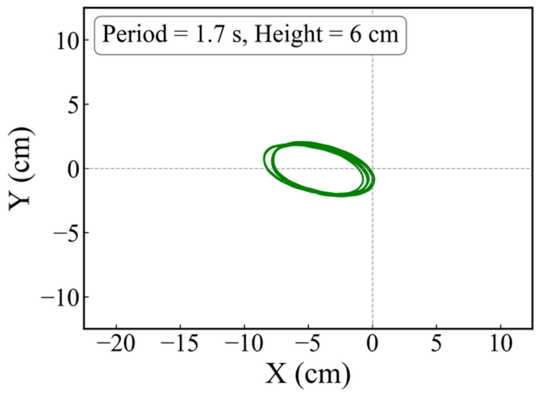

Displacement

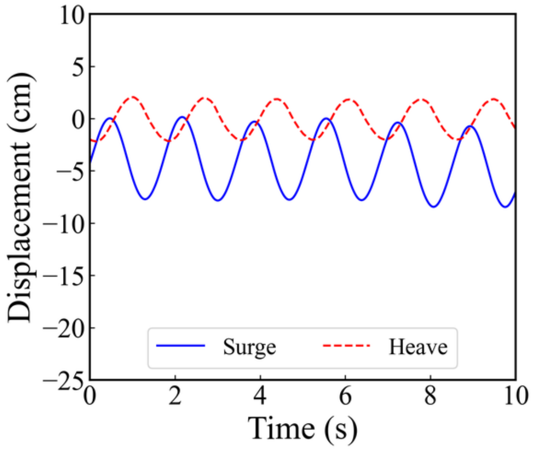

Velocity

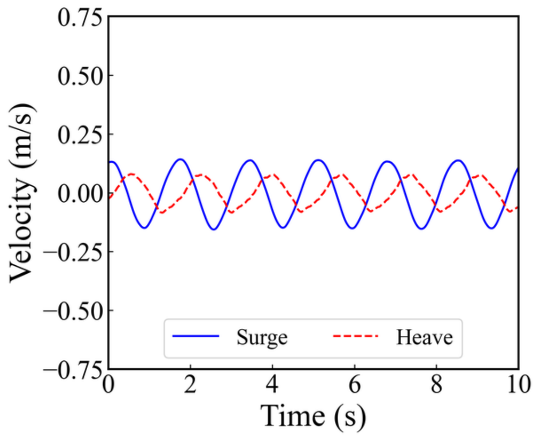

Rope Length

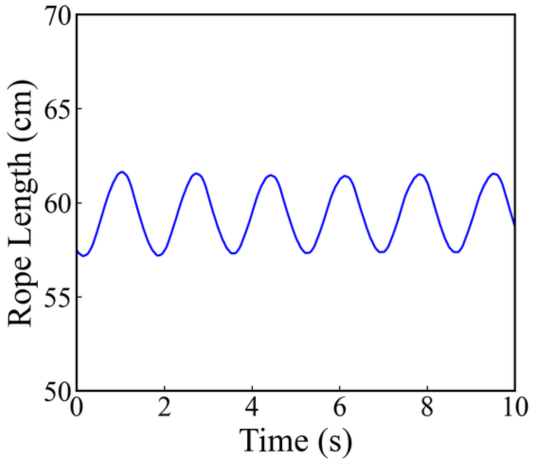

Rope Velocity

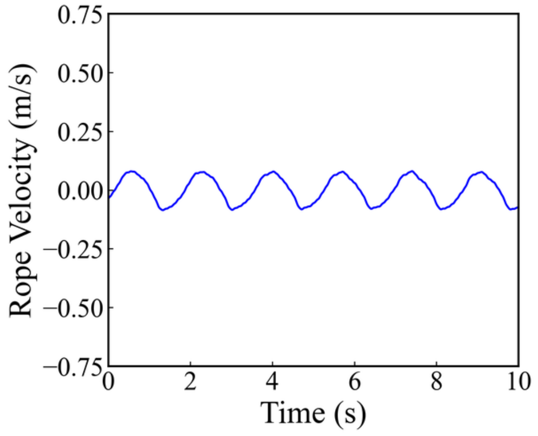

Regular wave: Period1.7s_Height7cm

Trajectory

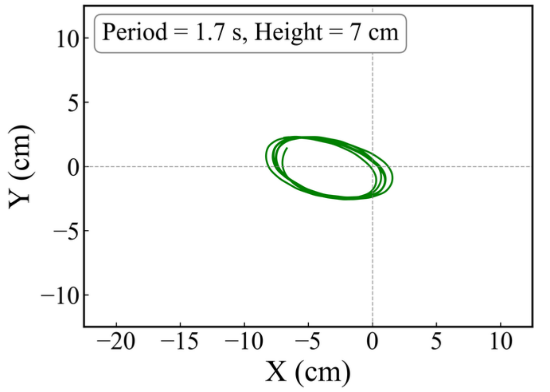

Displacement

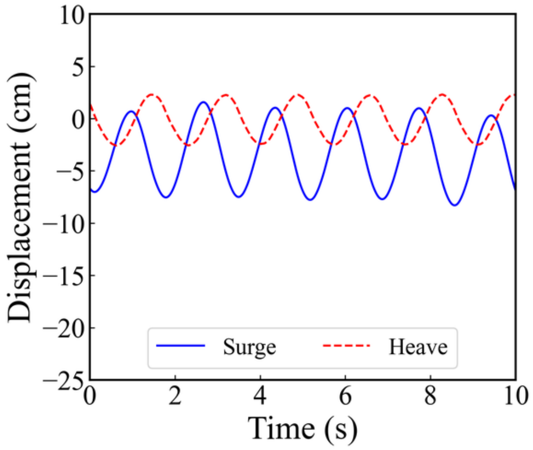

Velocity

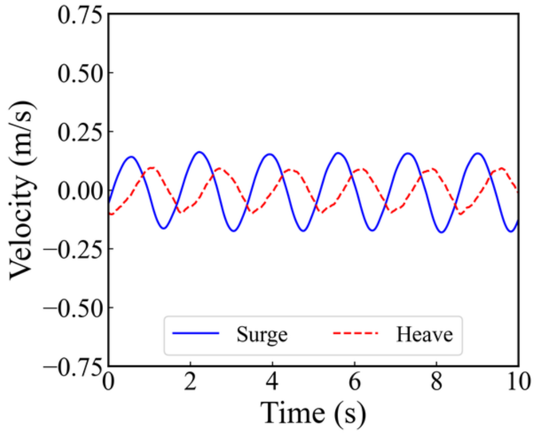

Rope Length

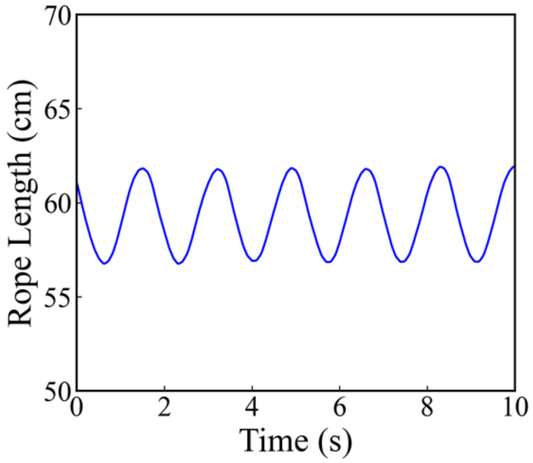

Rope Velocity

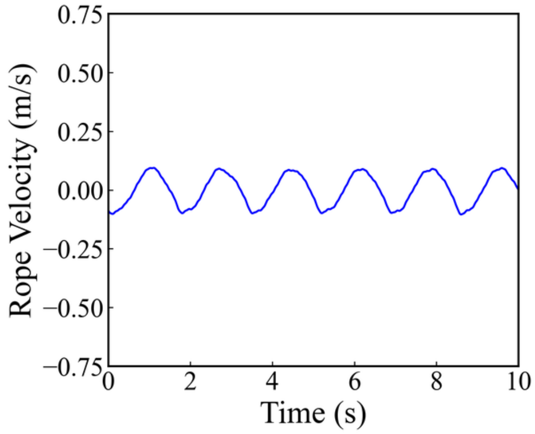

Regular wave: Period1.7s_Height8cm

Trajectory

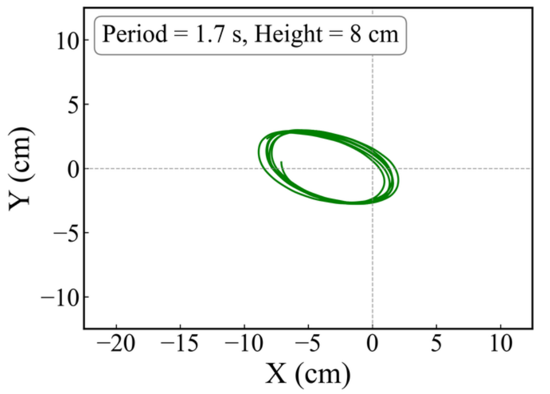

Displacement

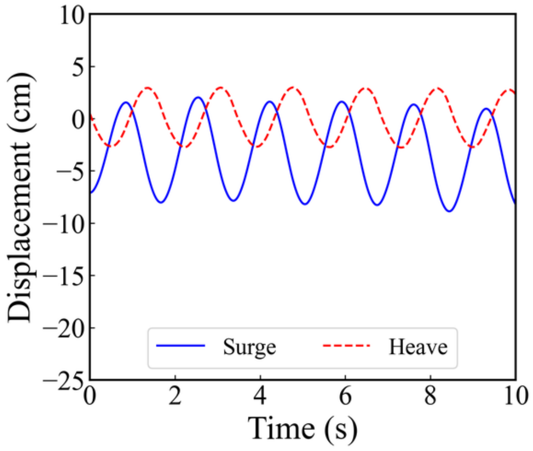

Velocity

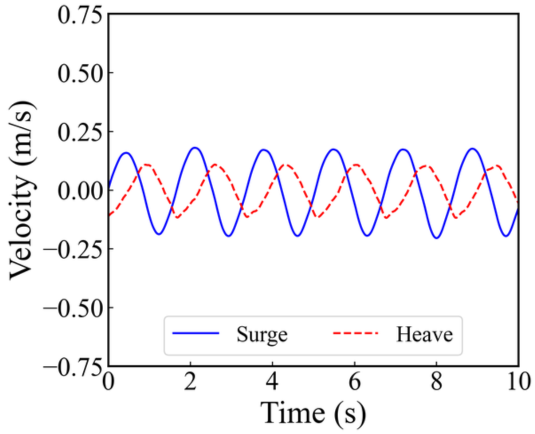

Rope Length

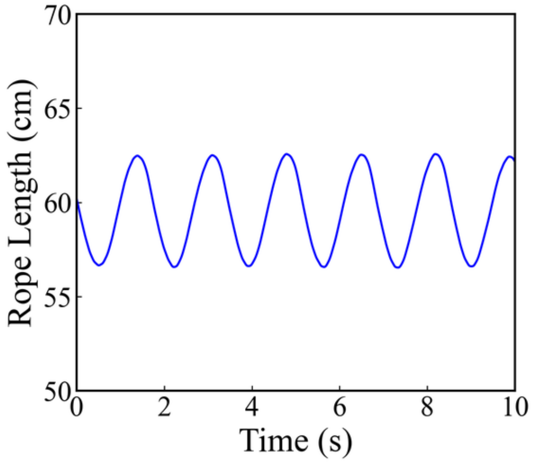

Rope Velocity

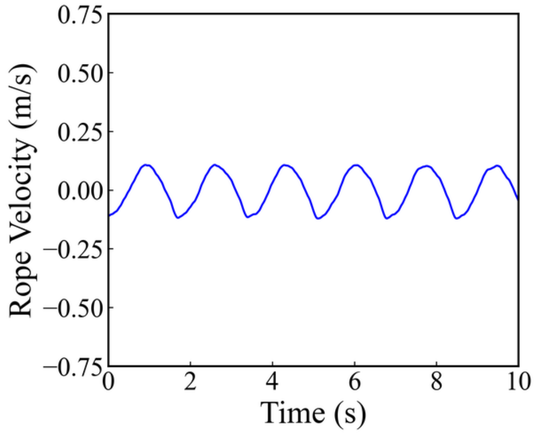

Regular wave: Period1.7s_Height9cm

Trajectory

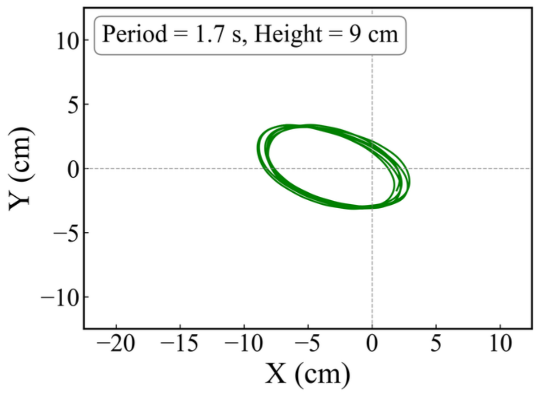

Displacement

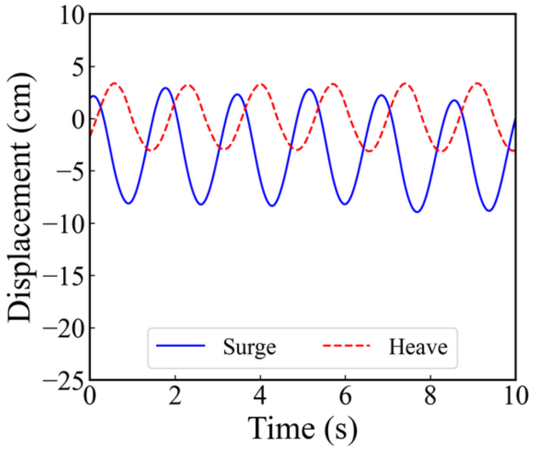

Velocity

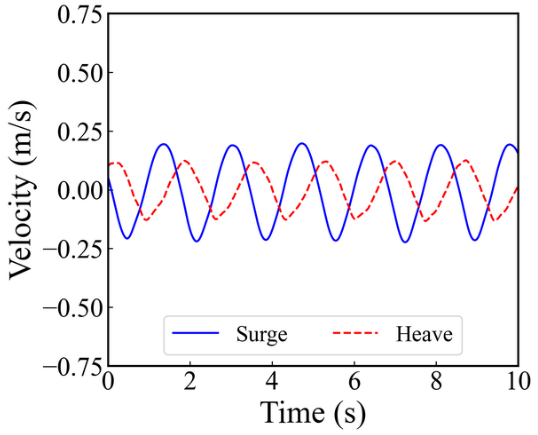

Rope Length

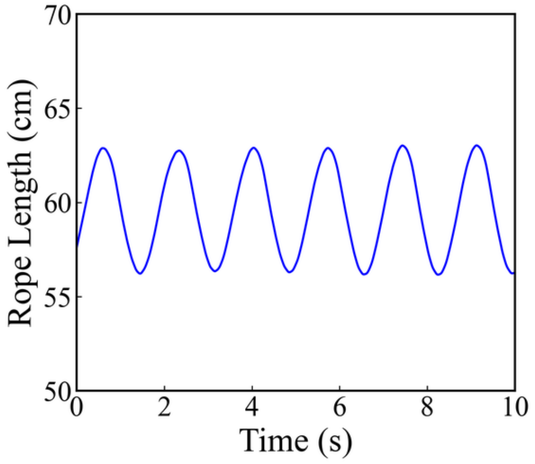

Rope Velocity

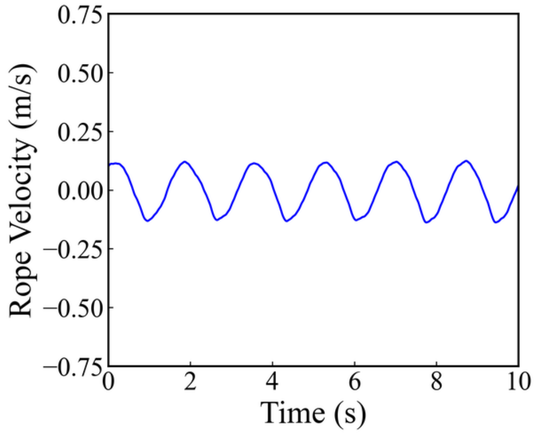

Regular wave: Period1.7s_Height10cm

Trajectory

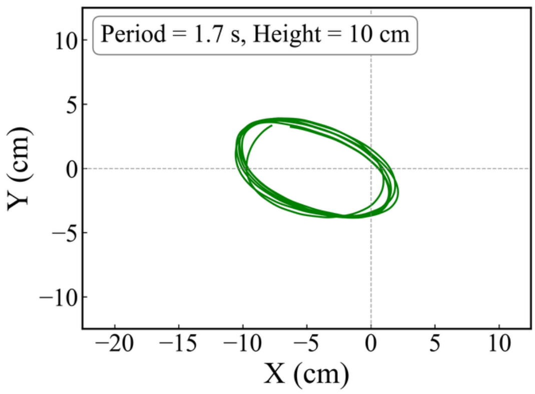

Displacement

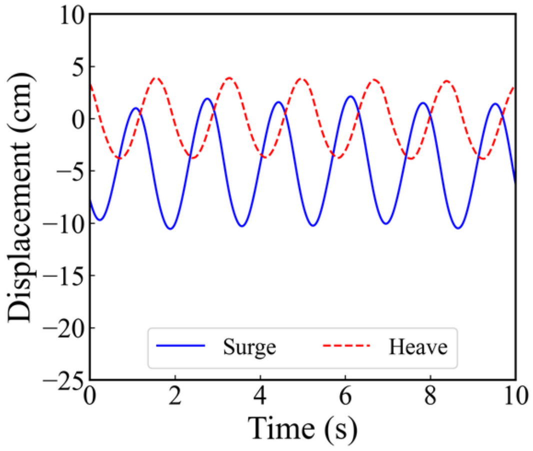

Velocity

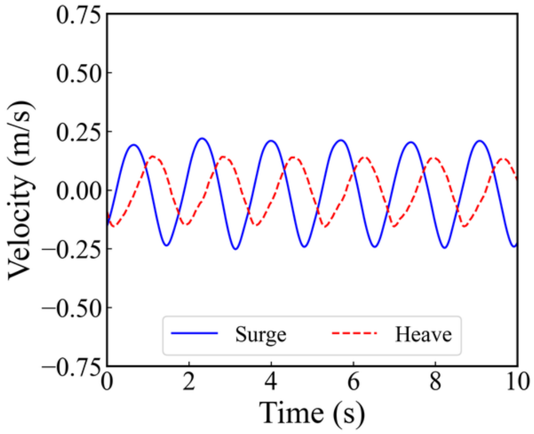

Rope Length

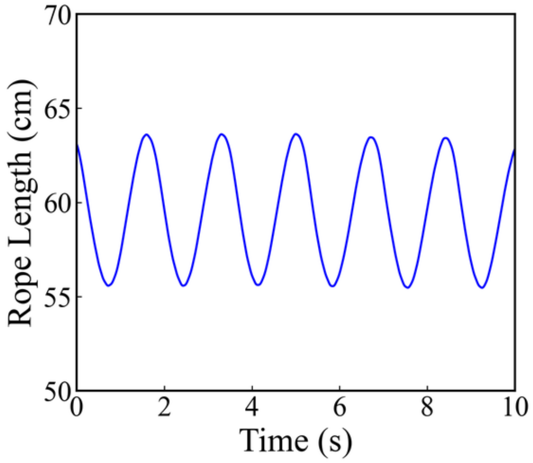

Rope Velocity

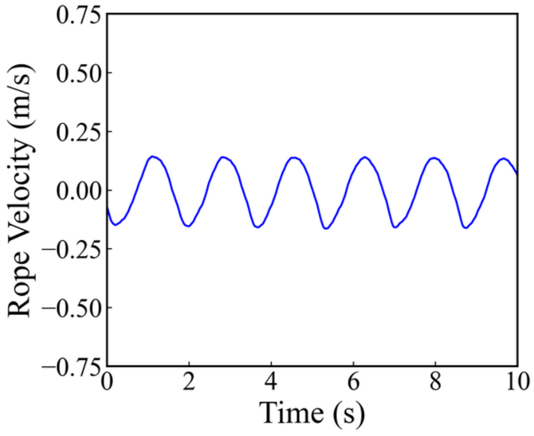

Regular wave: Period1.7s_Height11cm

Trajectory

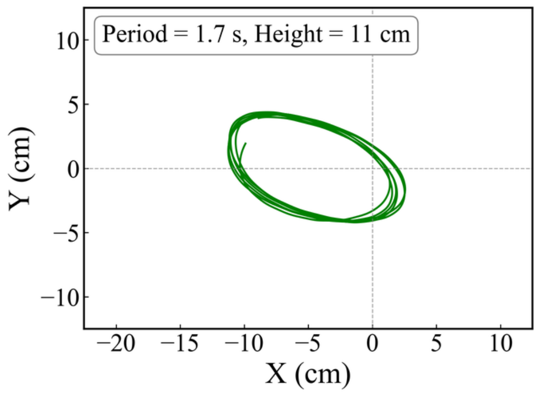

Displacement

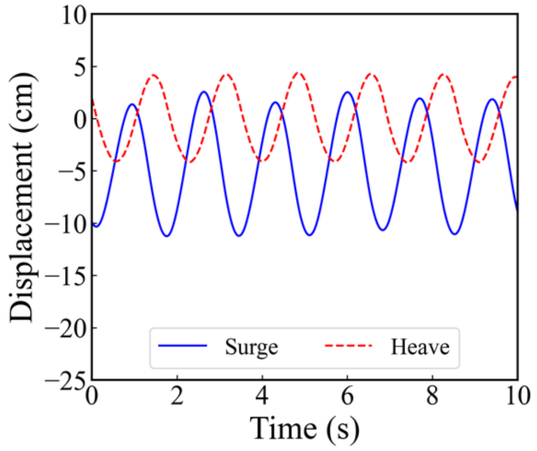

Velocity

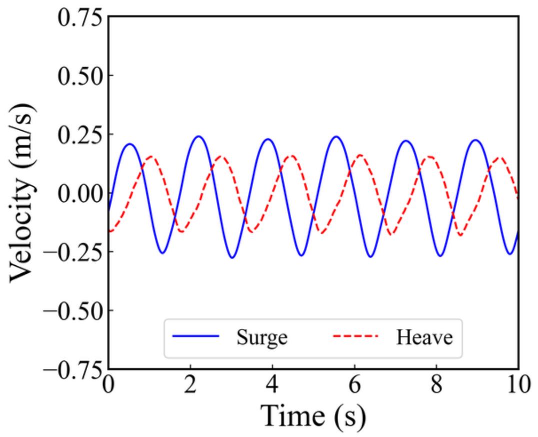

Rope Length

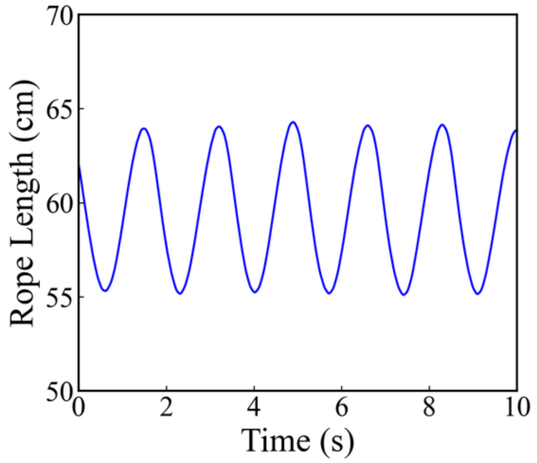

Rope Velocity

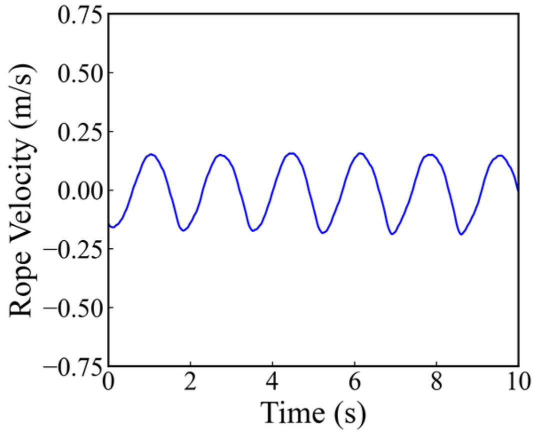

Regular wave: Period1.7s_Height12cm

Trajectory

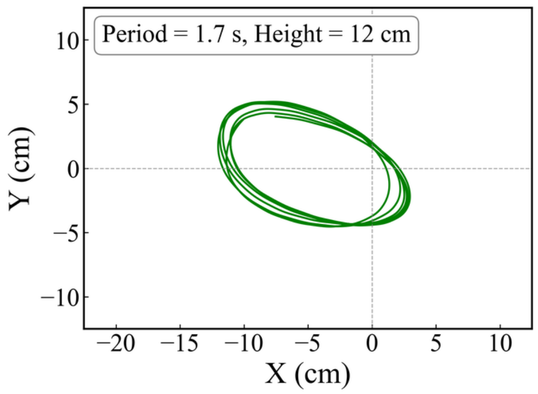

Displacement

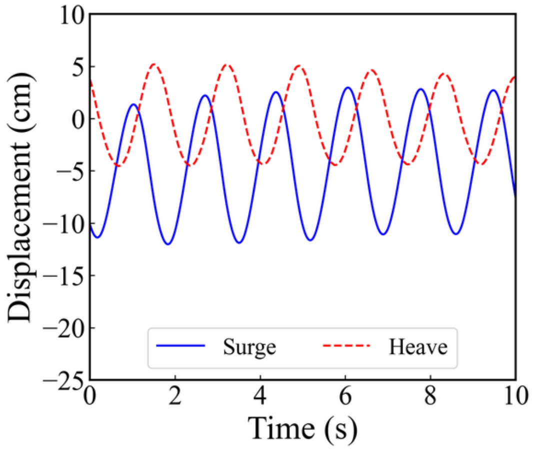

Velocity

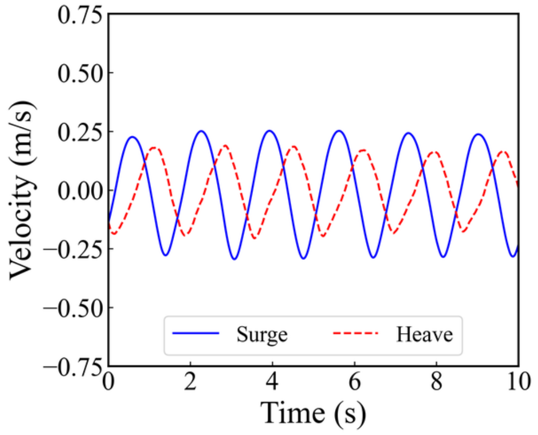

Rope Length

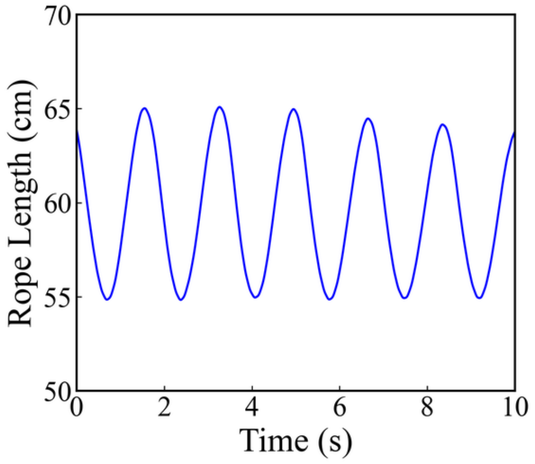

Rope Velocity

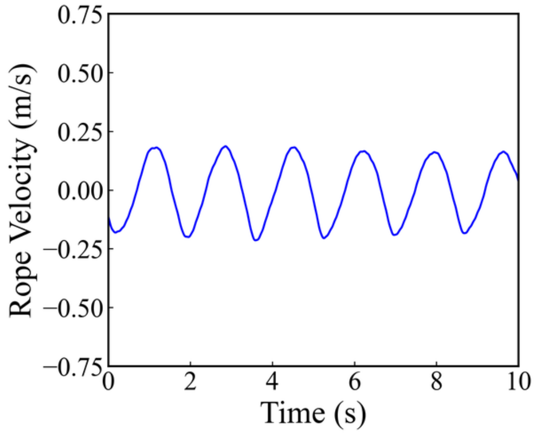

Regular wave: Period1.7s_Height13cm

Trajectory

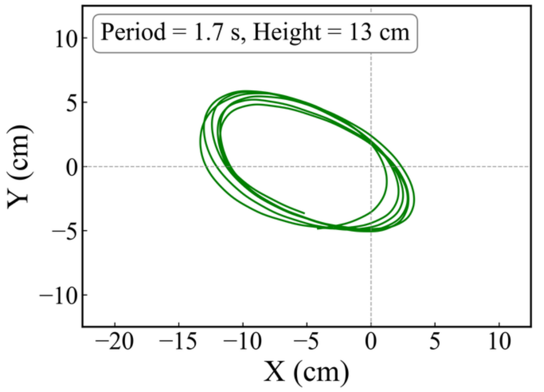

Displacement

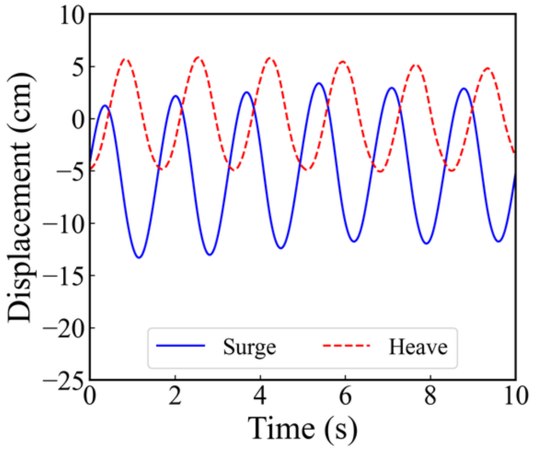

Velocity

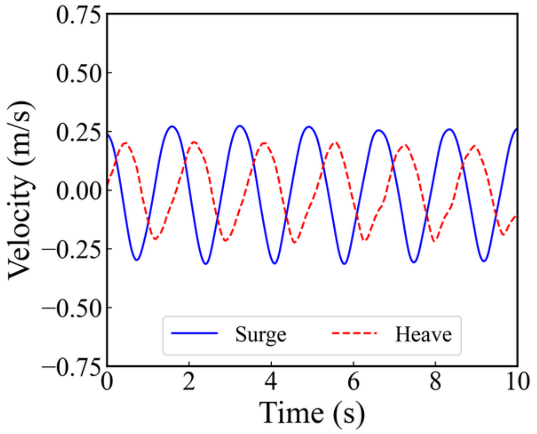

Rope Length

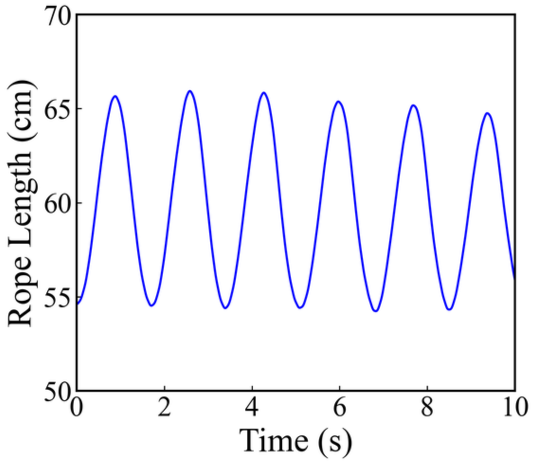

Rope Velocity

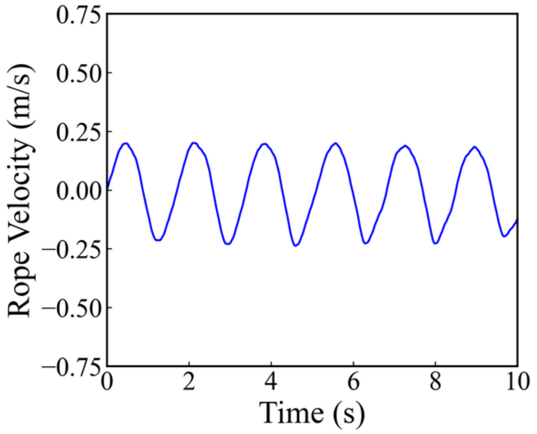

Regular wave: Period1.7s_Height14cm

Trajectory

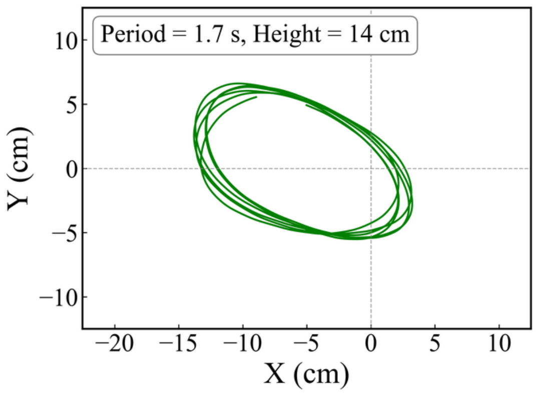

Displacement

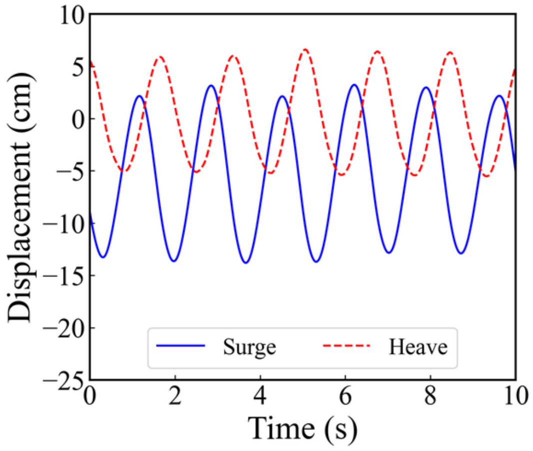

Velocity

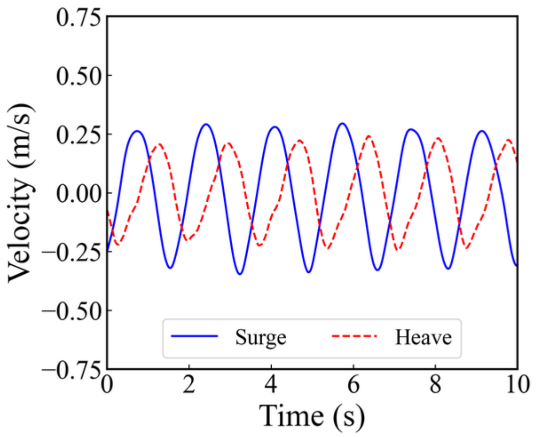

Rope Length

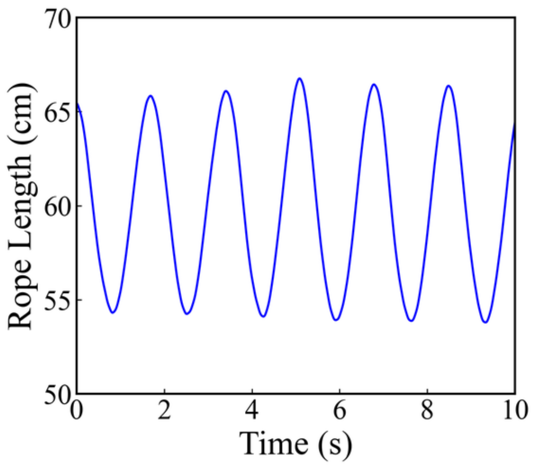

Rope Velocity

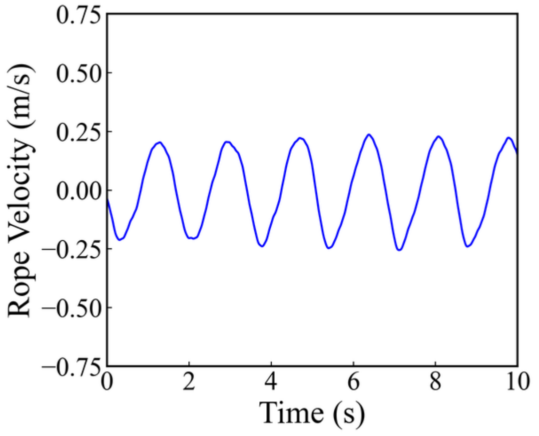

Regular wave: Period1.7s_Height15cm

Trajectory

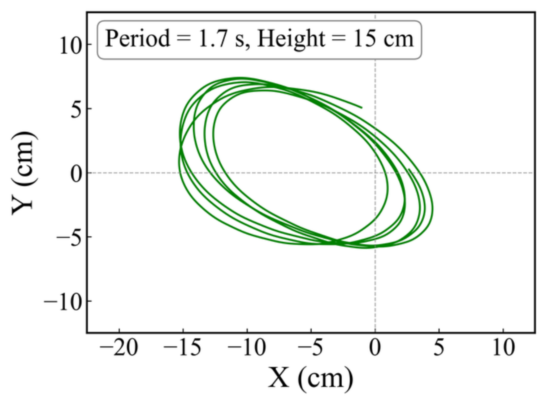

Displacement

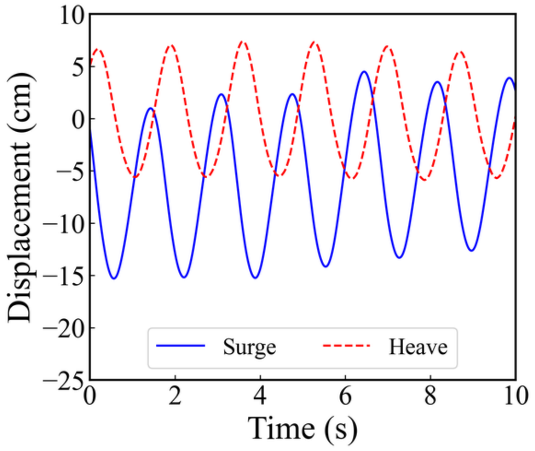

Velocity

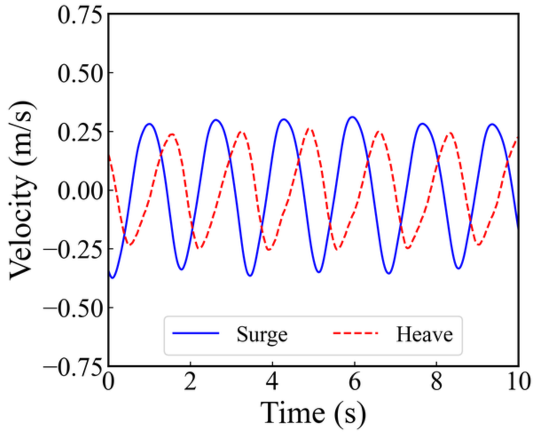

Rope Length

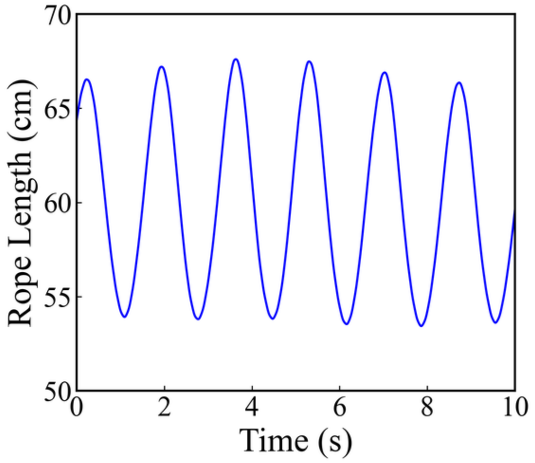

Rope Velocity

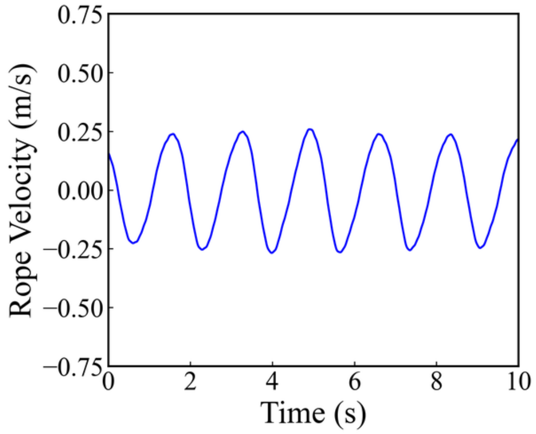

Regular wave: Period1.8s_Height5cm

Trajectory

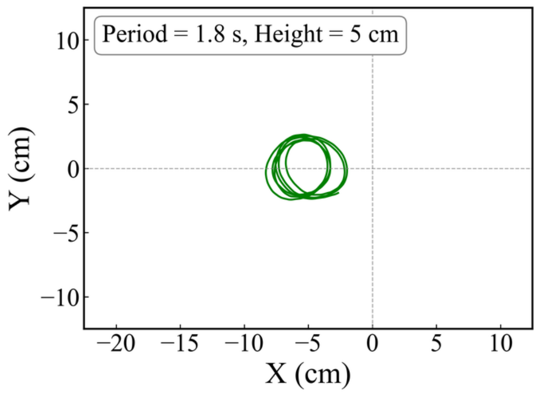

Displacement

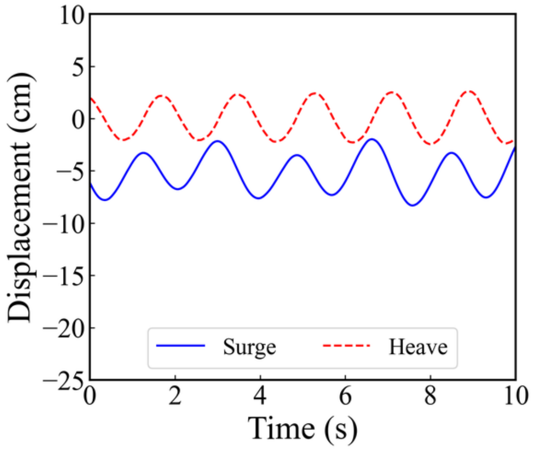

Velocity

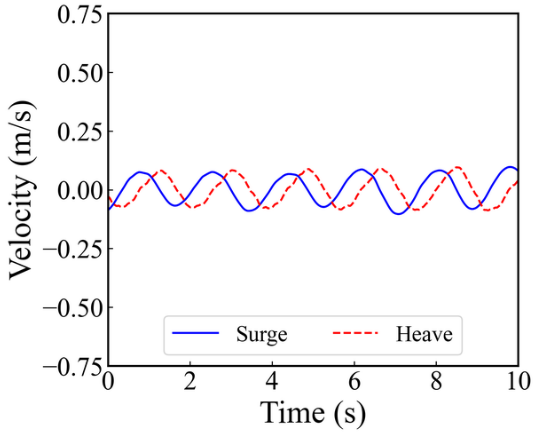

Rope Length

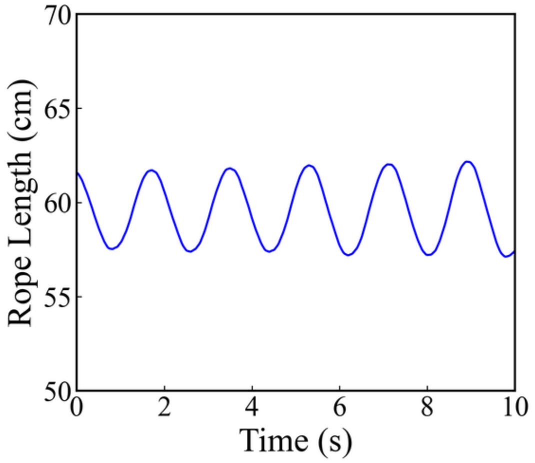

Rope Velocity

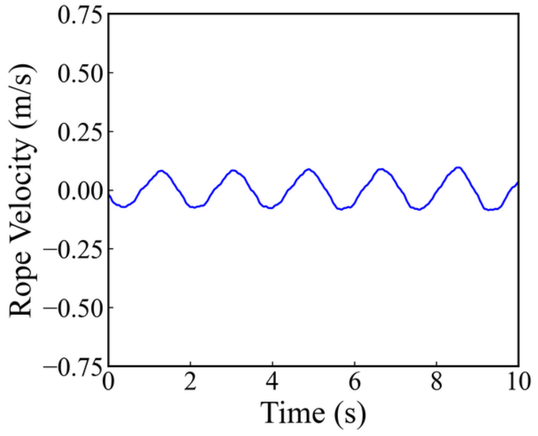

Regular wave: Period1.8s_Height6cm

Trajectory

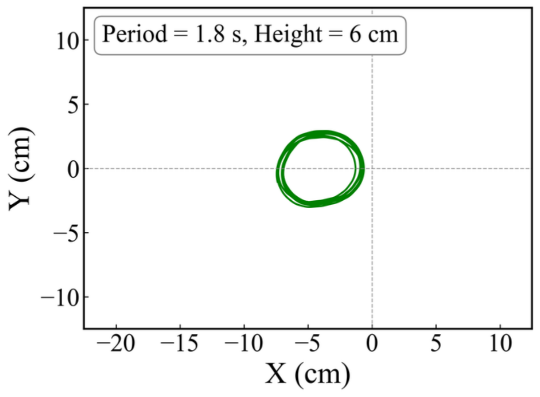

Displacement

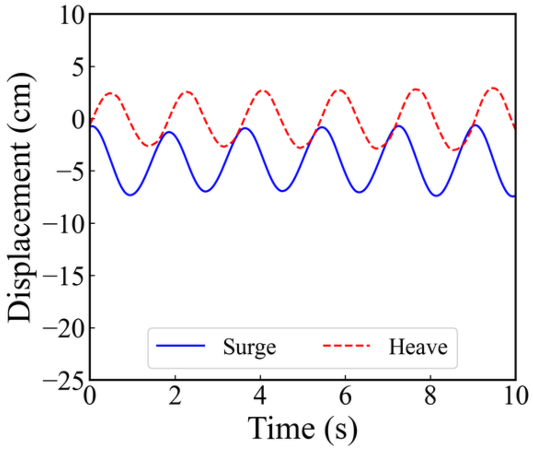

Velocity

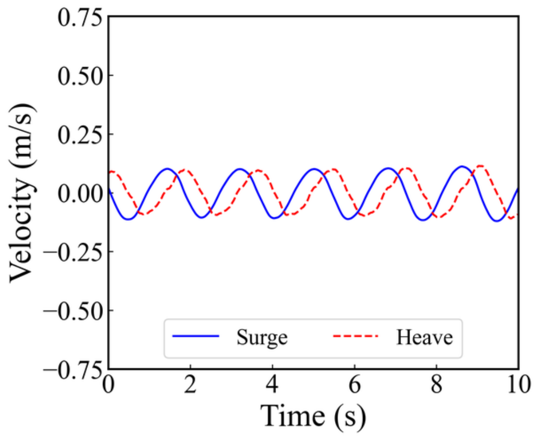

Rope Length

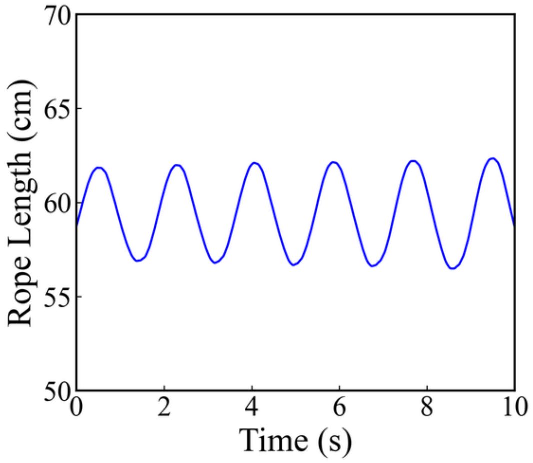

Rope Velocity

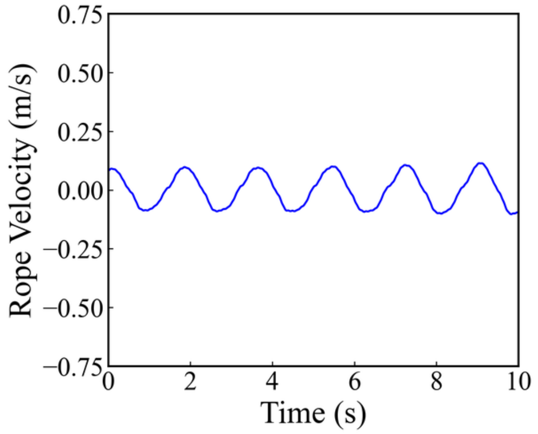

Regular wave: Period1.8s_Height7cm

Trajectory

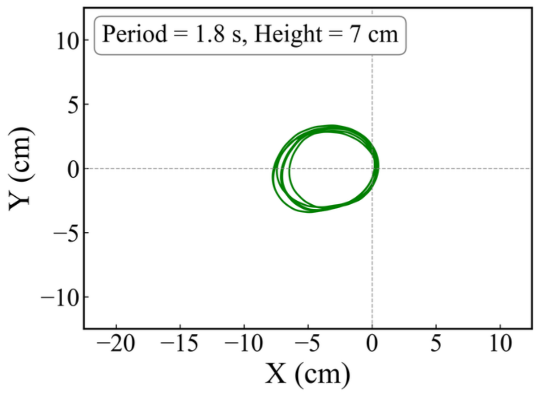

Displacement

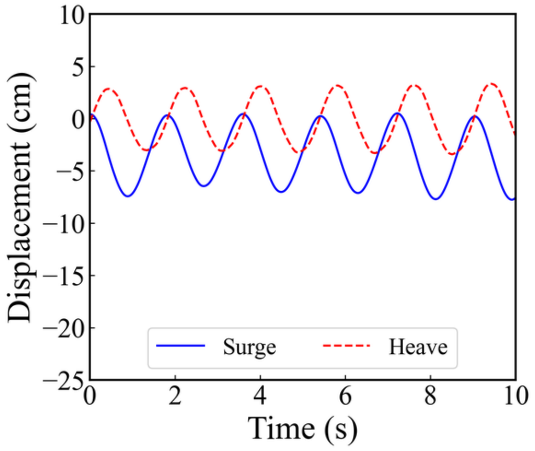

Velocity

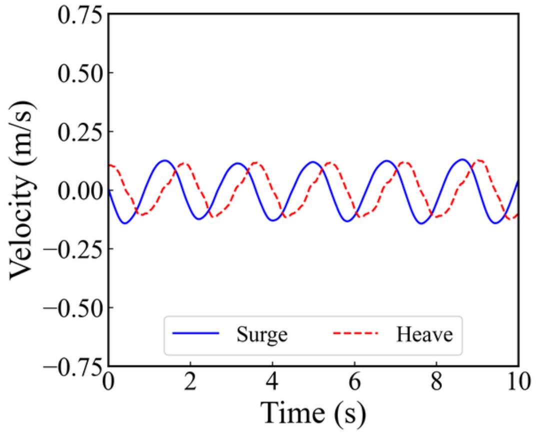

Rope Length

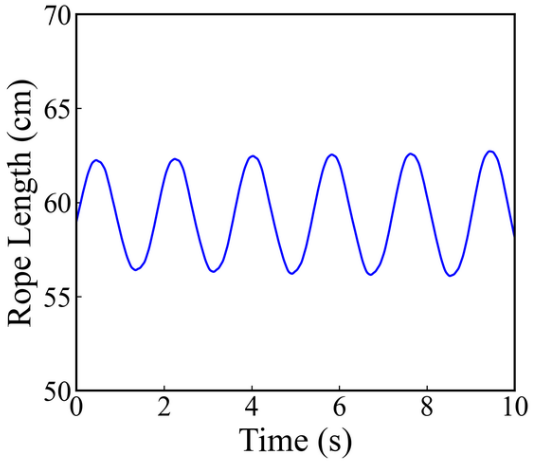

Rope Velocity

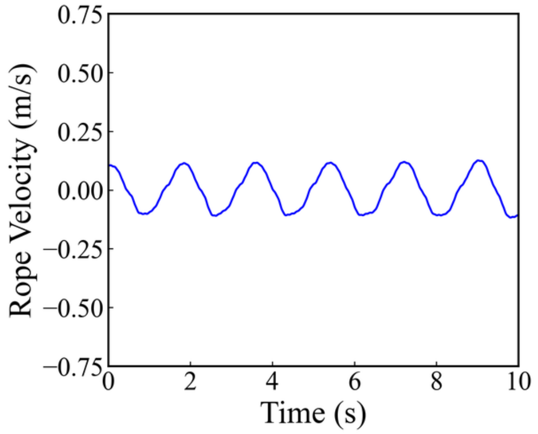

Regular wave: Period1.8s_Height8cm

Trajectory

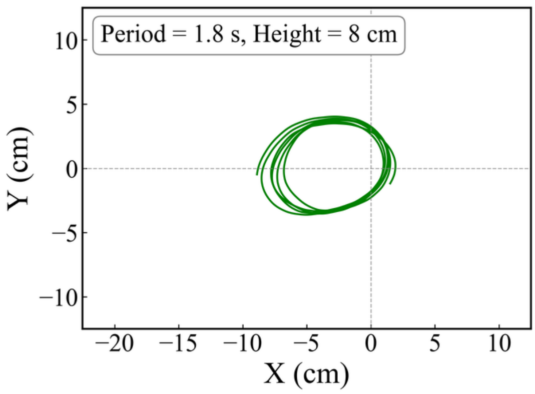

Displacement

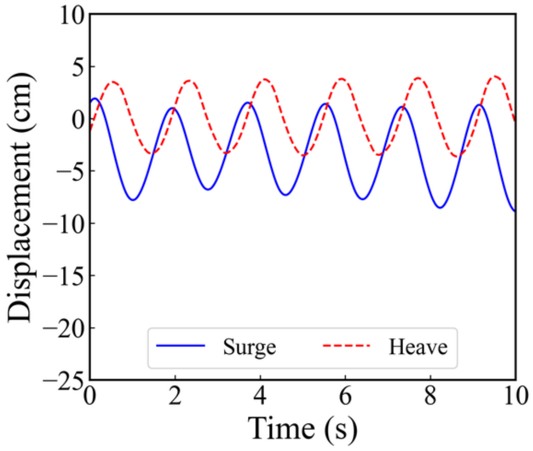

Velocity

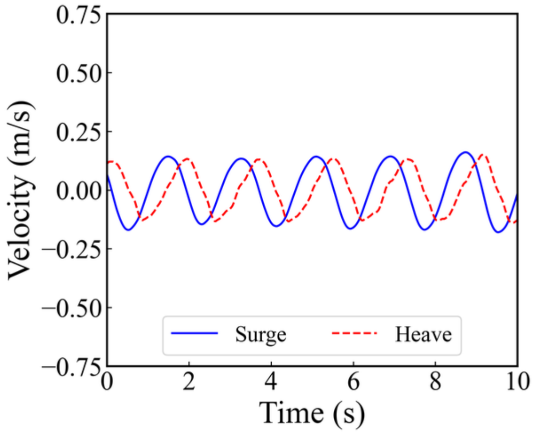

Rope Length

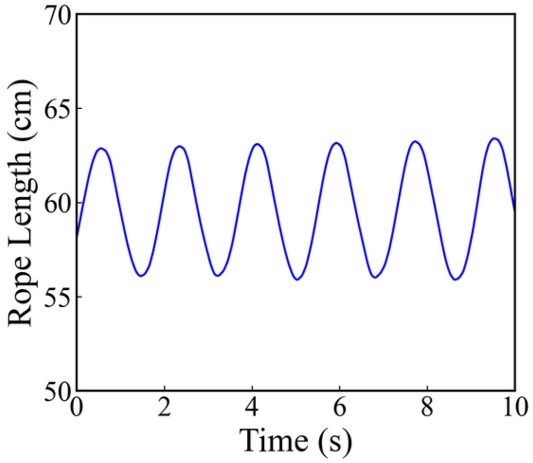

Rope Velocity

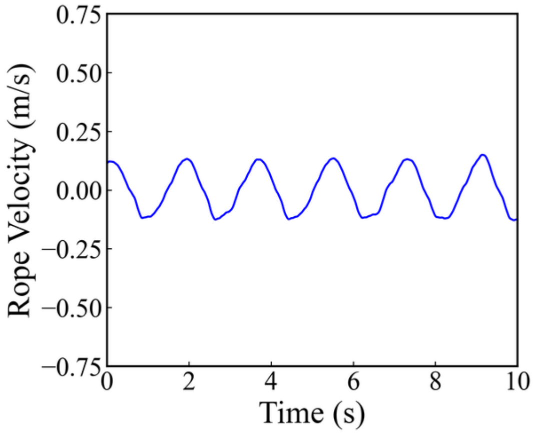

Regular wave: Period1.8s_Height9cm

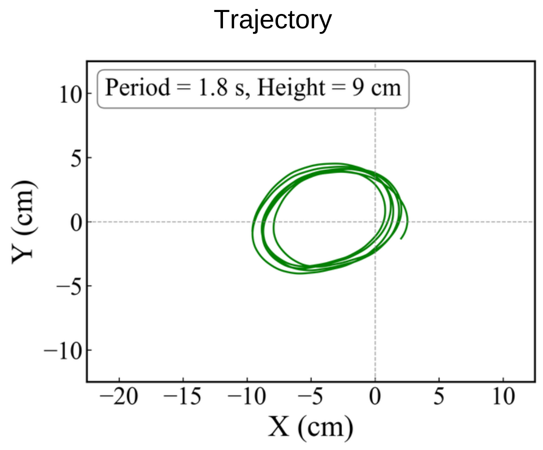

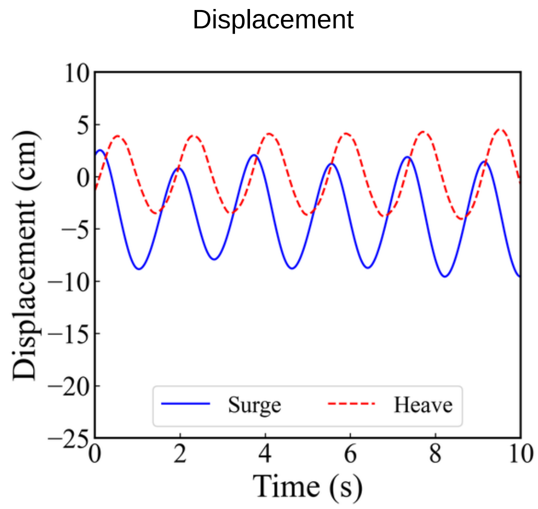

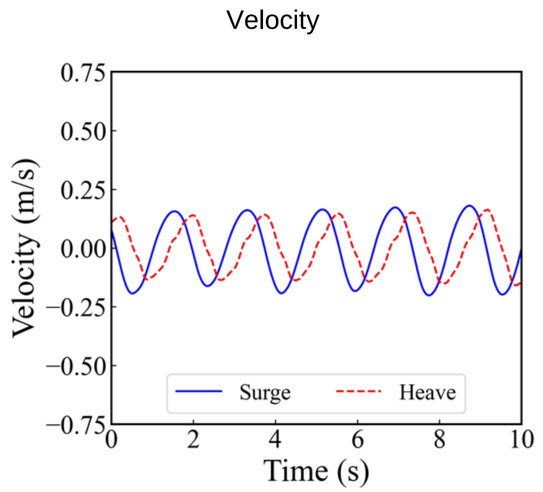

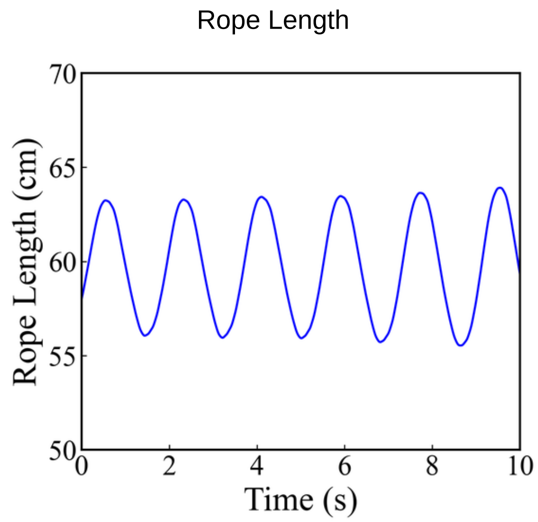

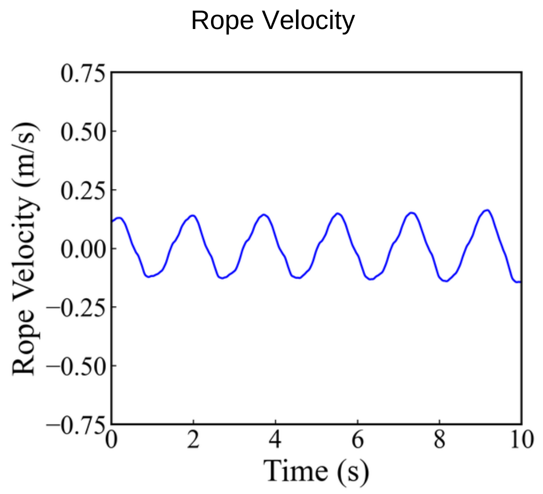

Regular wave: Period1.8s_Height10cm

Trajectory

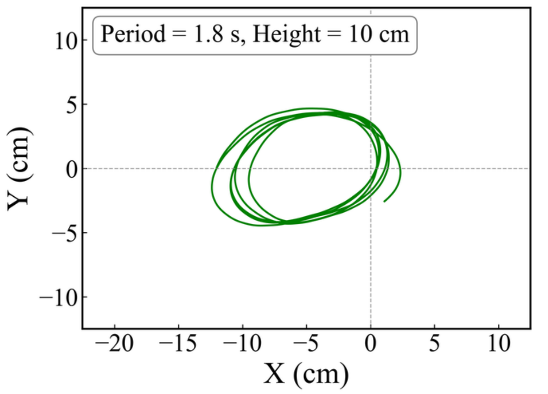

Displacement

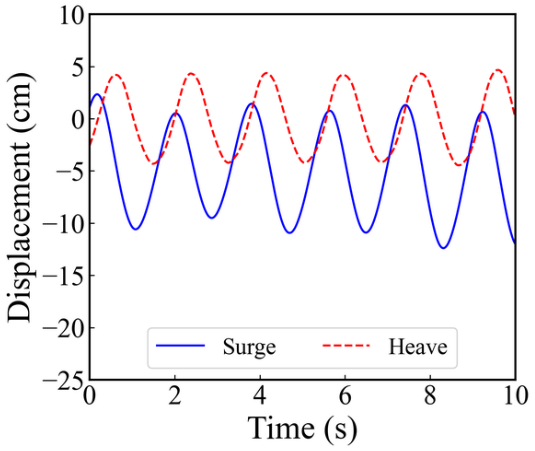

Velocity

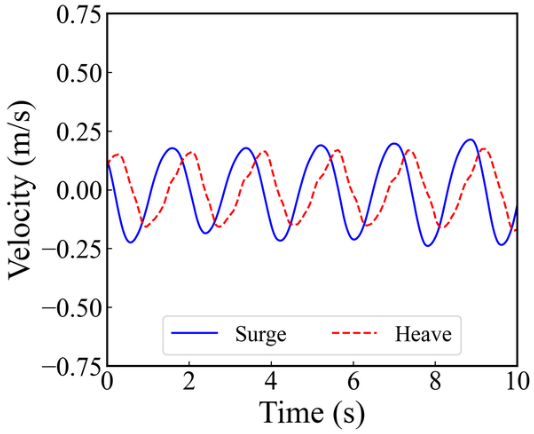

Rope Length

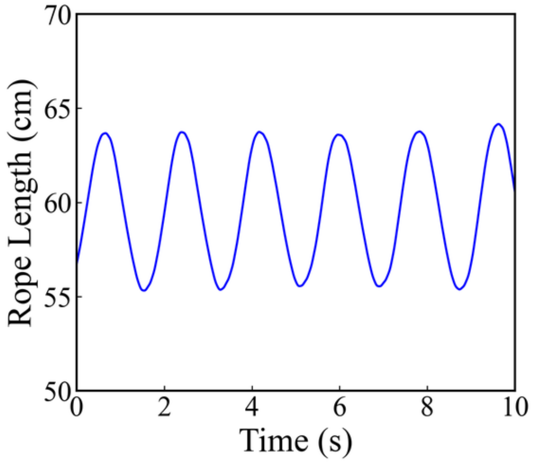

Rope Velocity

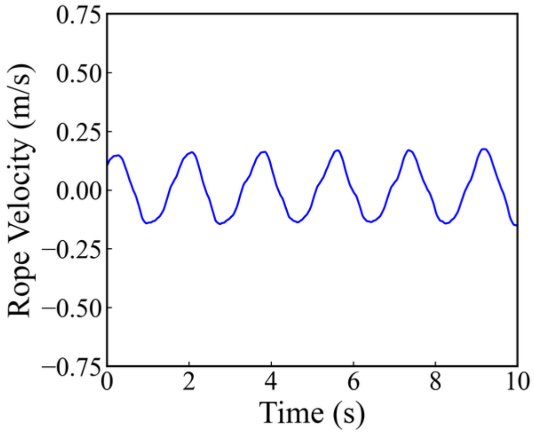

Regular wave: Period1.8s_Height11cm

Trajectory

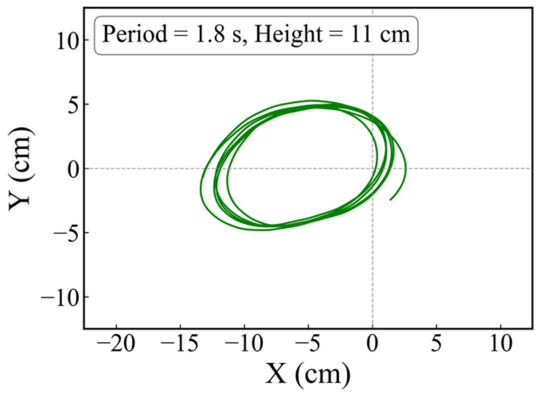

Displacement

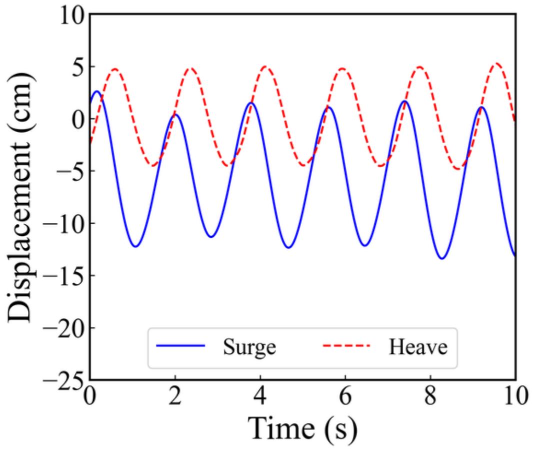

Velocity

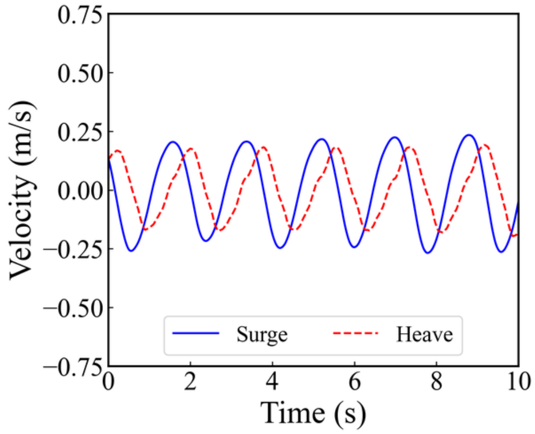

Rope Length

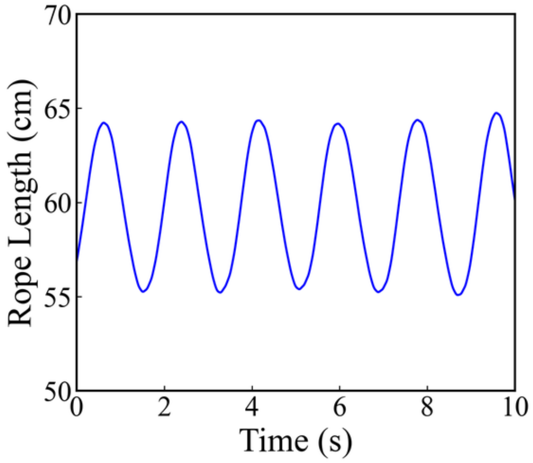

Rope Velocity

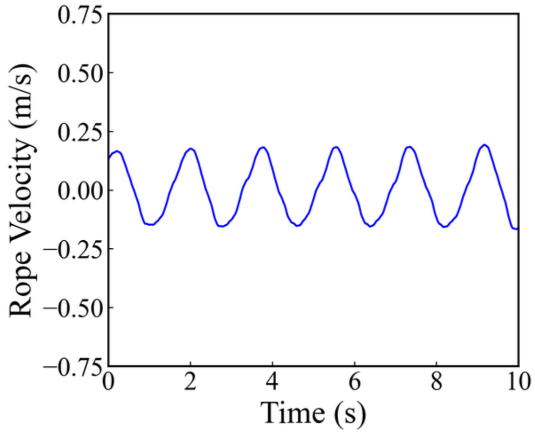

Regular wave: Period1.8s_Height12cm

Trajectory

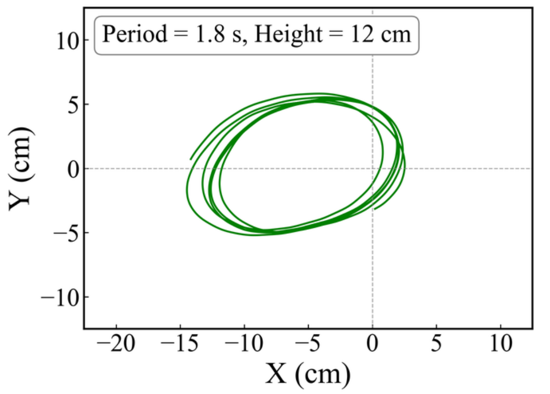

Displacement

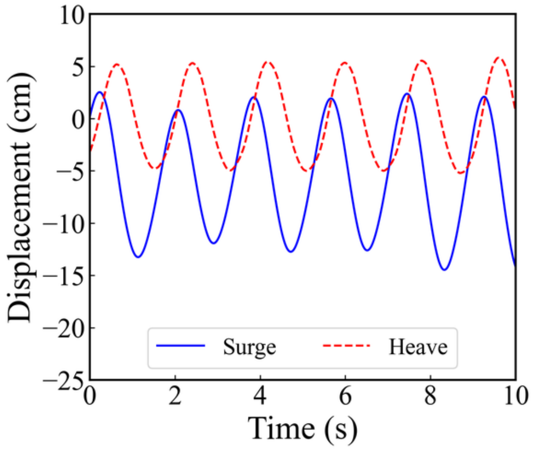

Velocity

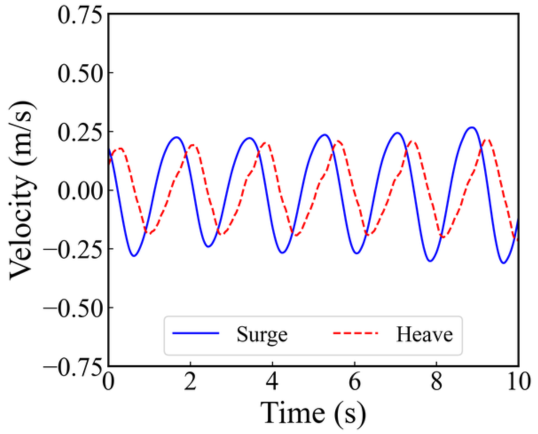

Rope Length

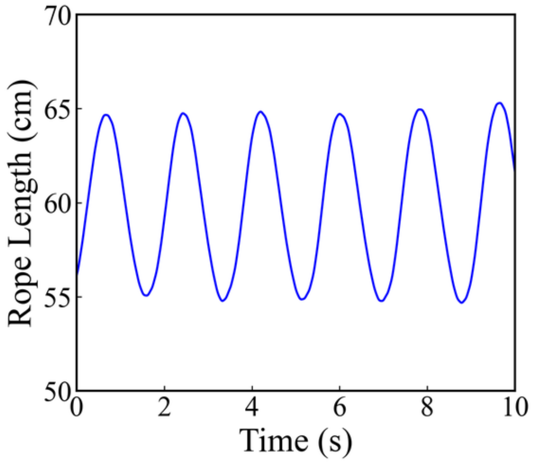

Rope Velocity

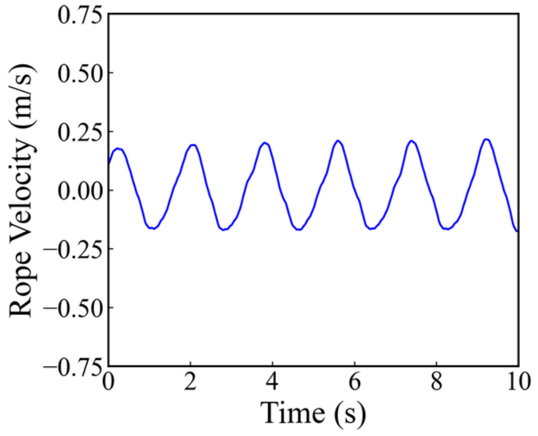

Regular wave: Period1.8s_Height13cm

Trajectory

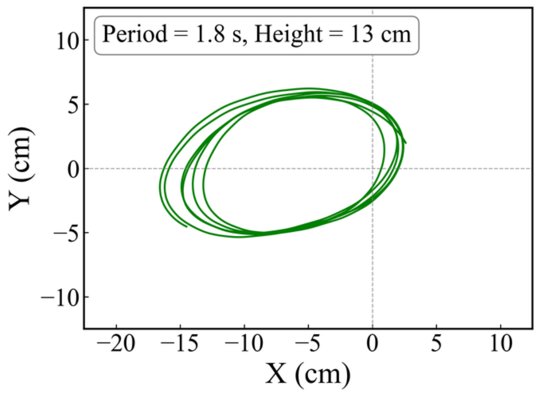

Displacement

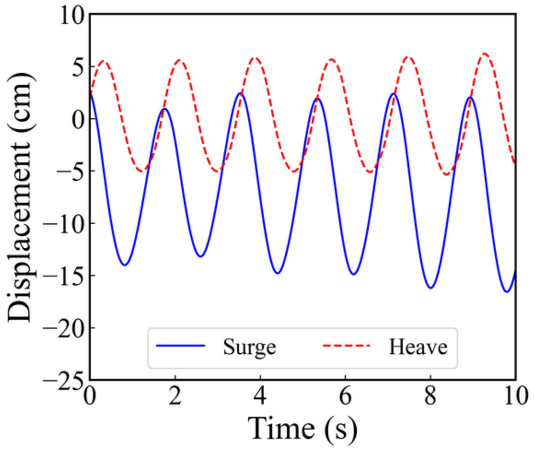

Velocity

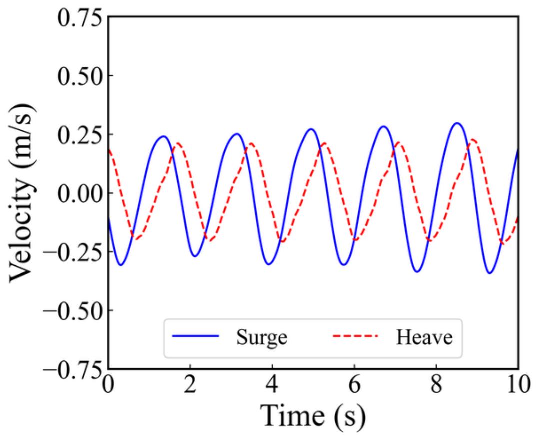

Rope Length

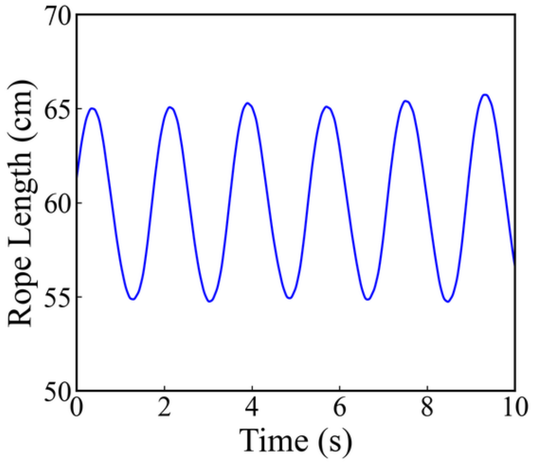

Rope Velocity

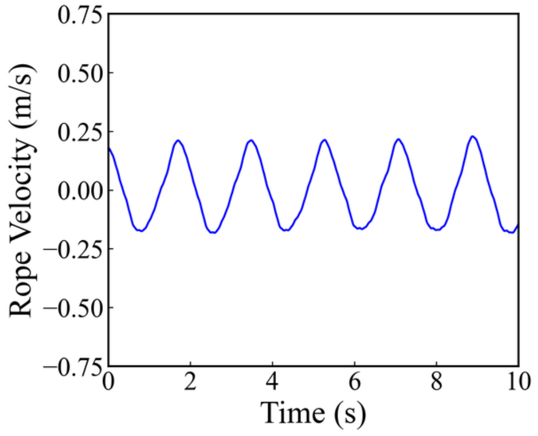

Regular wave: Period1.8s_Height14cm

Trajectory

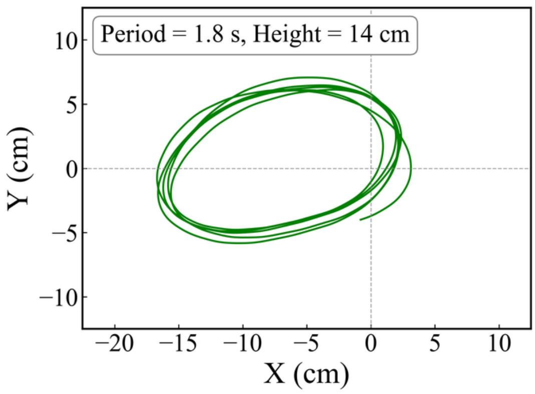

Displacement

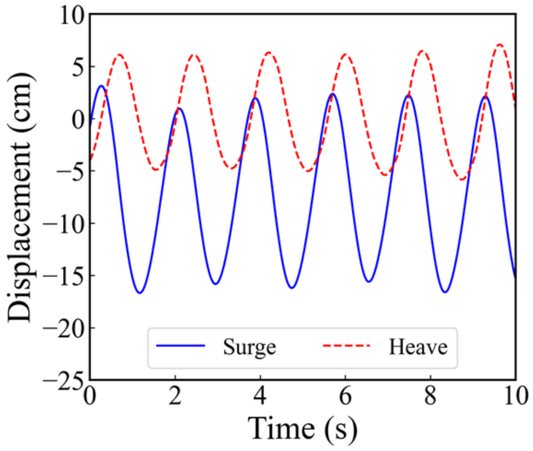

Velocity

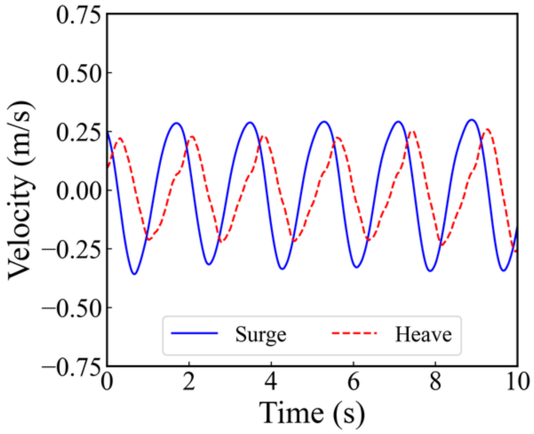

Rope Length

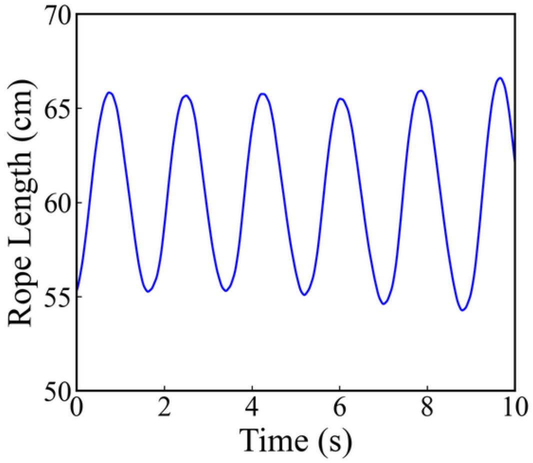

Rope Velocity

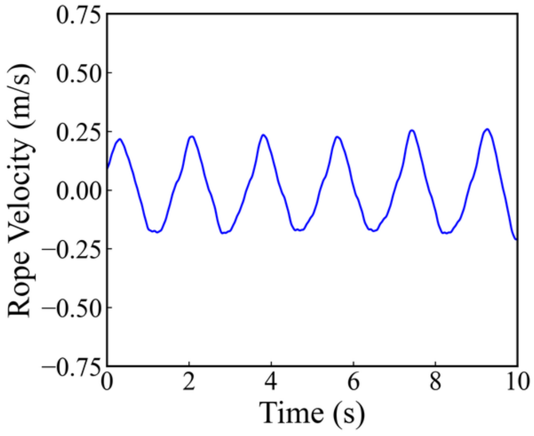

Regular wave: Period1.8s_Height15cm

Trajectory

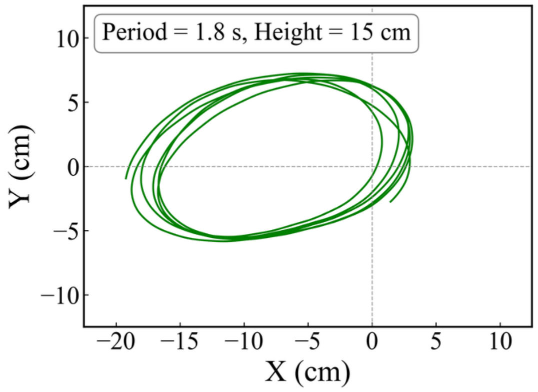

Displacement

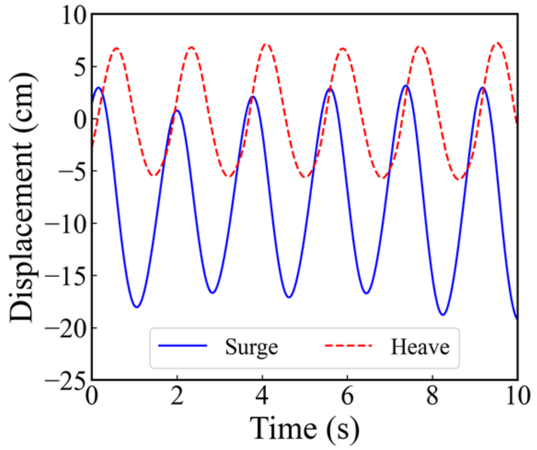

Velocity

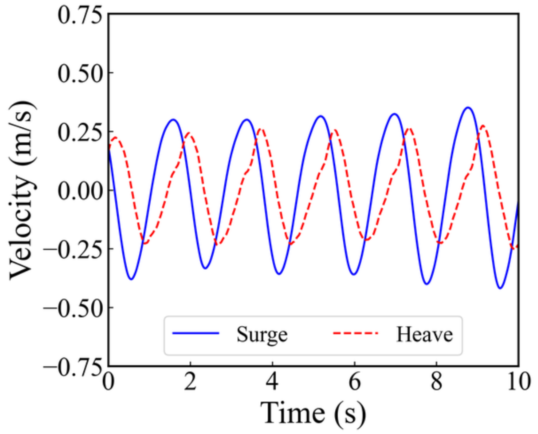

Rope Length

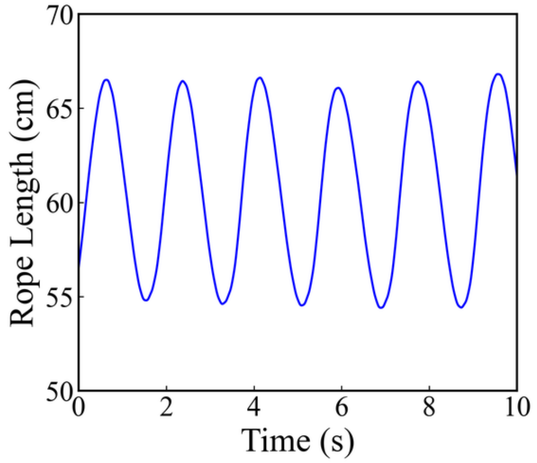

Rope Velocity

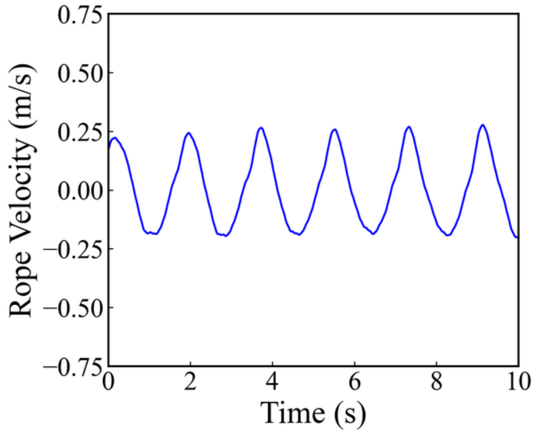

Regular wave: Period1.9s_Height5cm

Trajectory

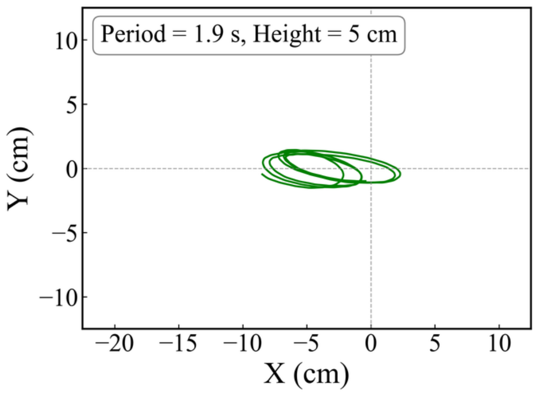

Displacement

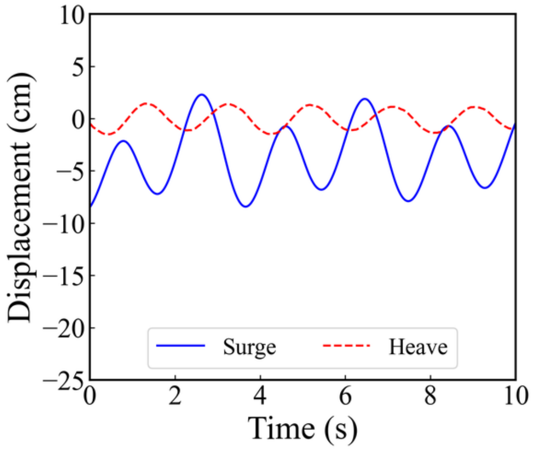

Velocity

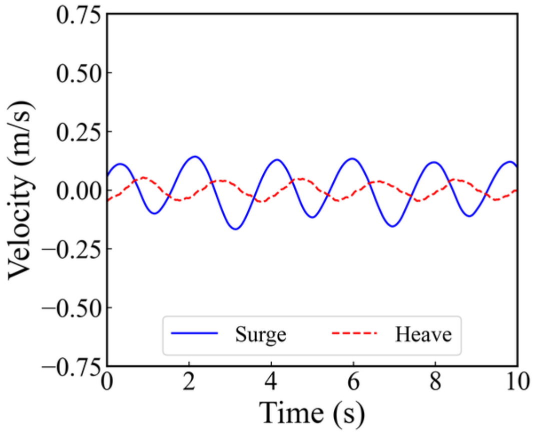

Rope Length

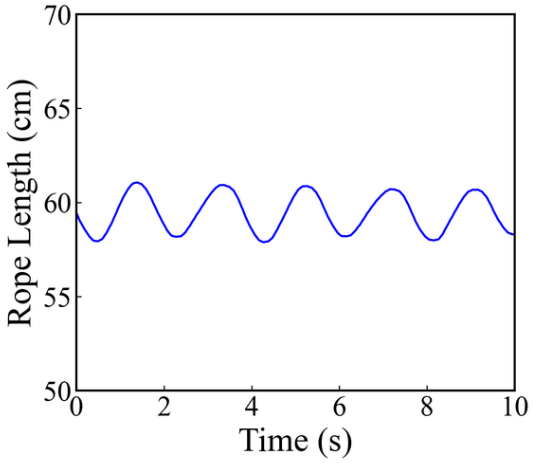

Rope Velocity

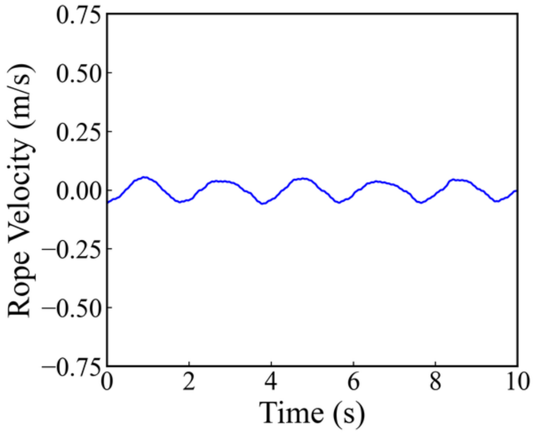

Regular wave: Period1.9s_Height6cm

Trajectory

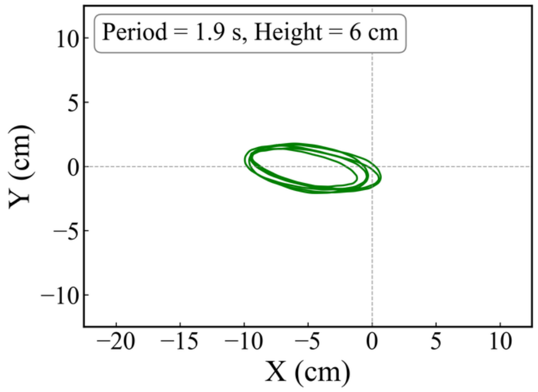

Displacement

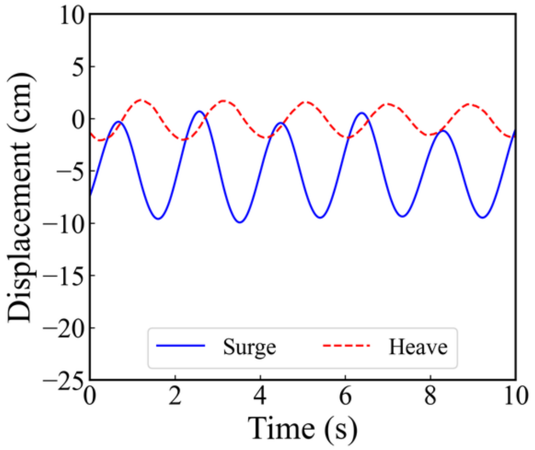

Velocity

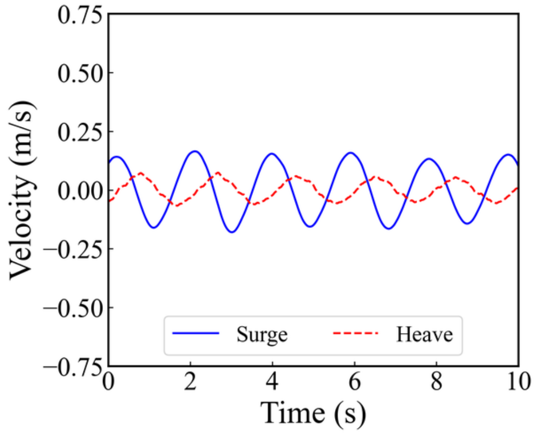

Rope Length

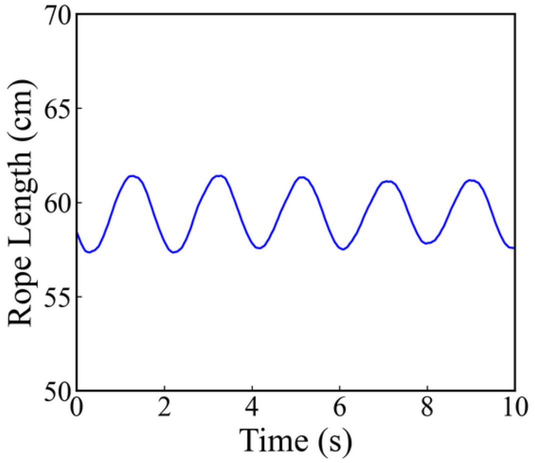

Rope Velocity

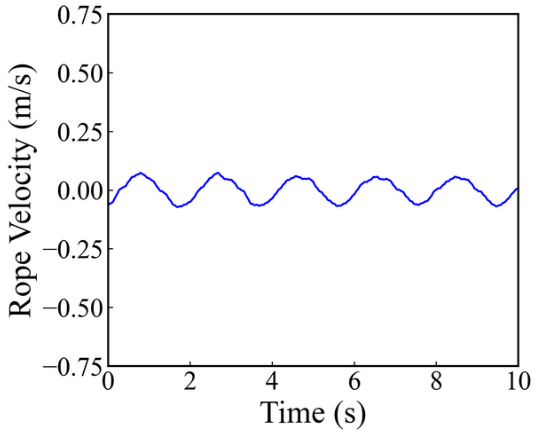

Regular wave: Period1.9s_Height7cm

Trajectory

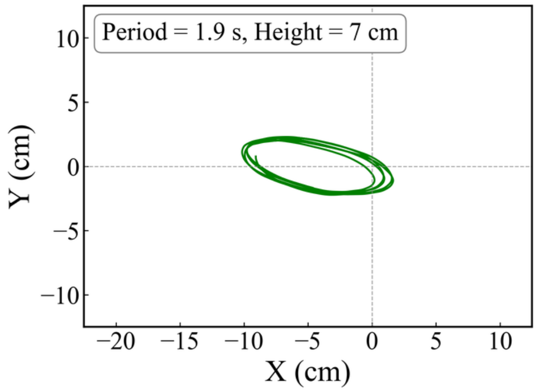

Displacement

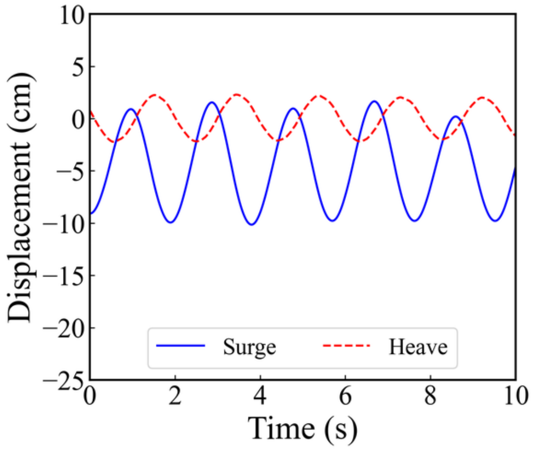

Velocity

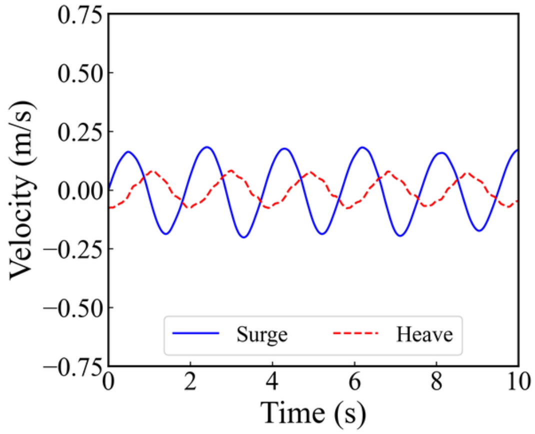

Rope Length

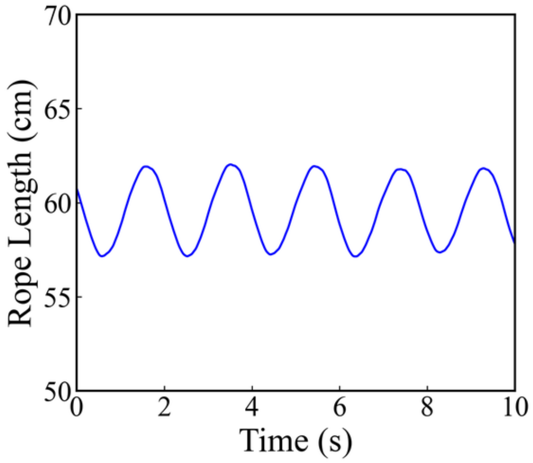

Rope Velocity

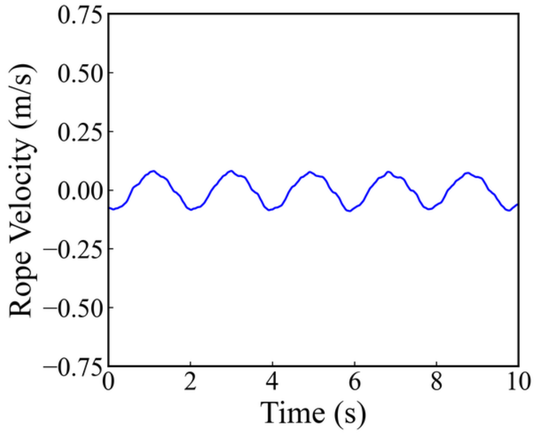

Regular wave: Period1.9s_Height8cm

Trajectory

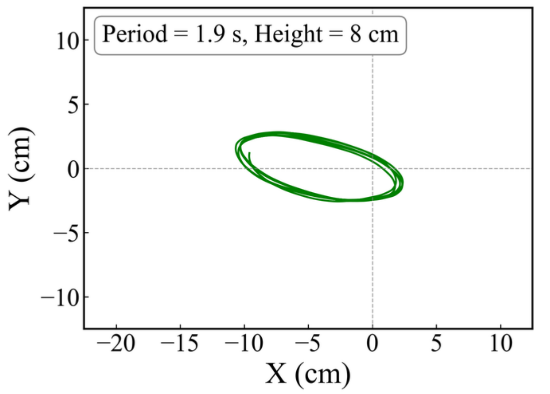

Displacement

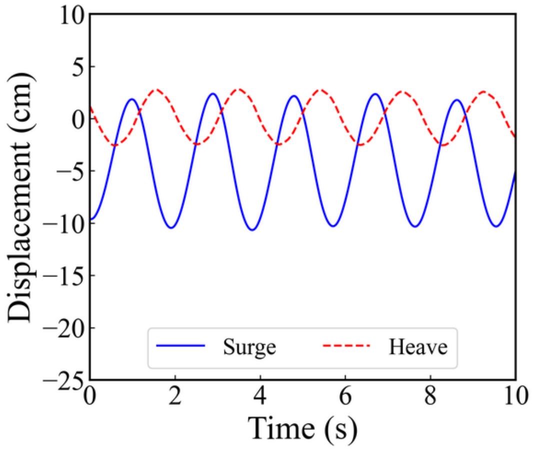

Velocity

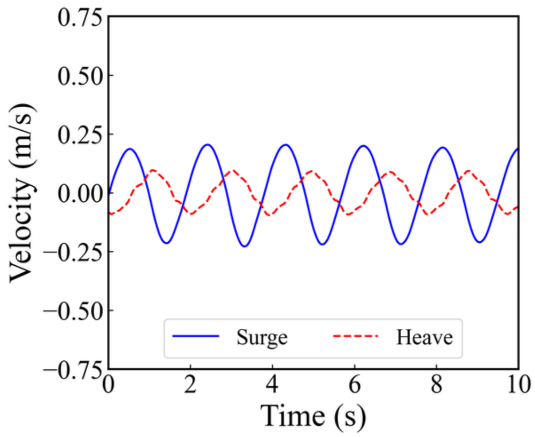

Rope Length

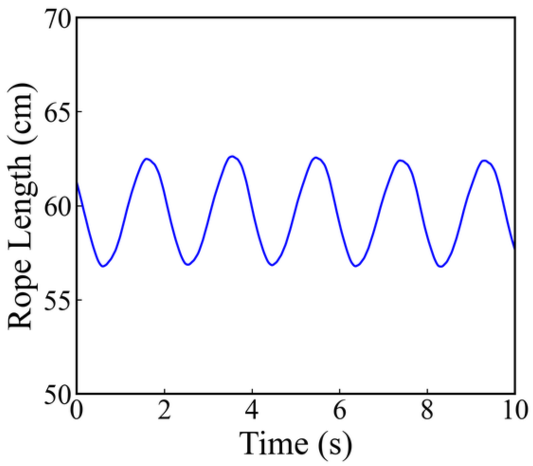

Rope Velocity

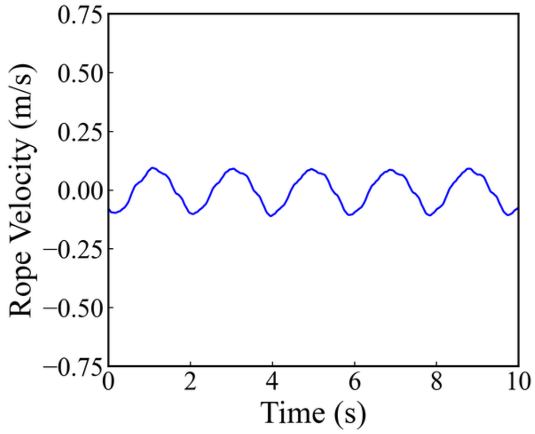

Regular wave: Period1.9s_Height9cm

Trajectory

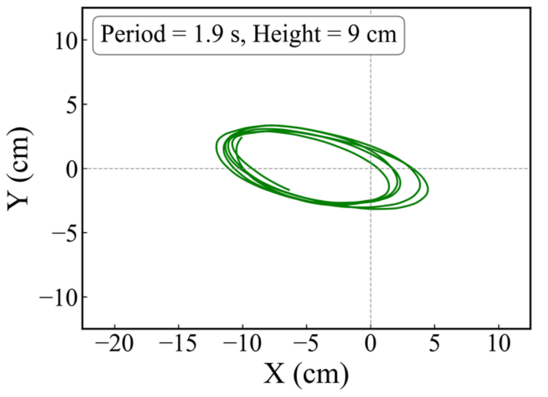

Displacement

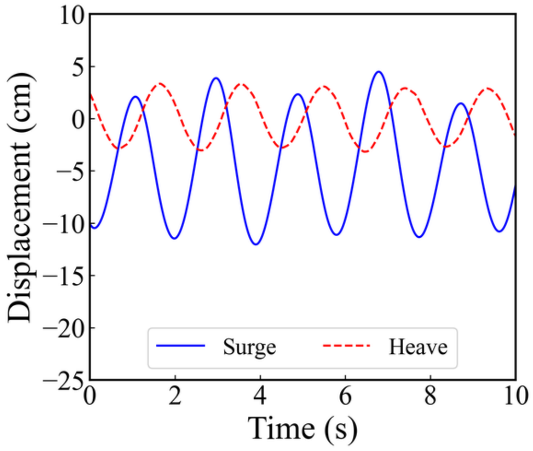

Velocity

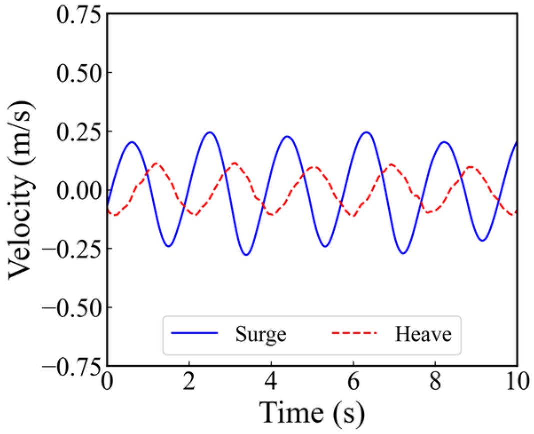

Rope Length

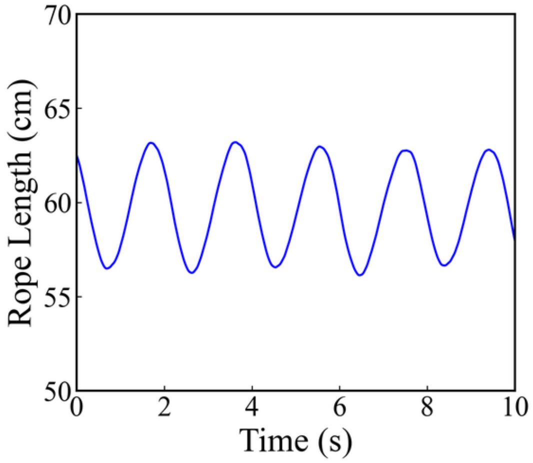

Rope Velocity

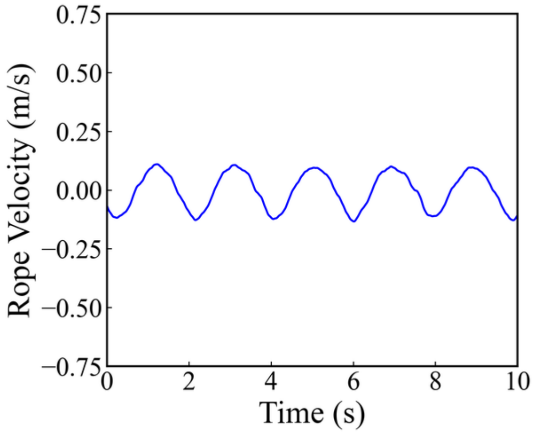

Regular wave: Period1.9s_Height10cm

Trajectory

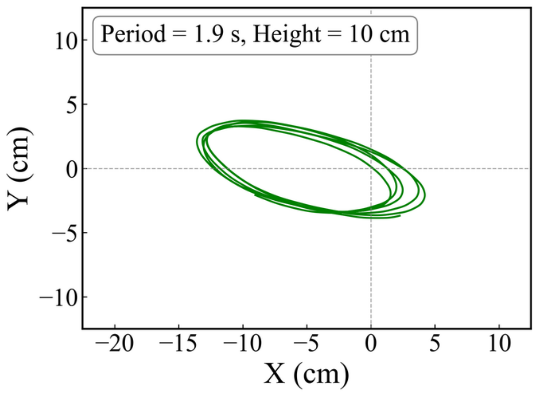

Displacement

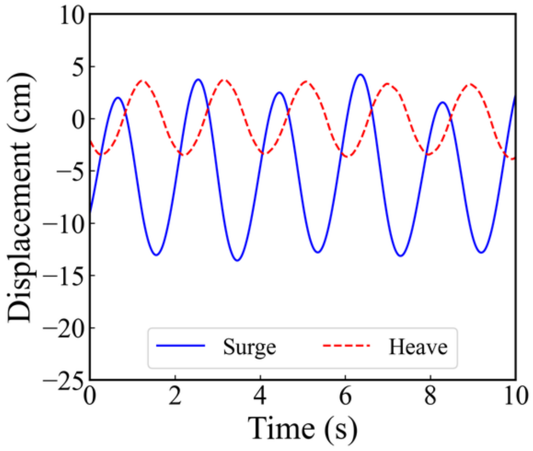

Velocity

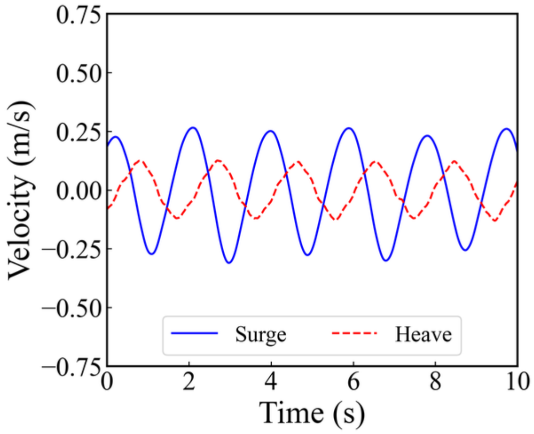

Rope Length

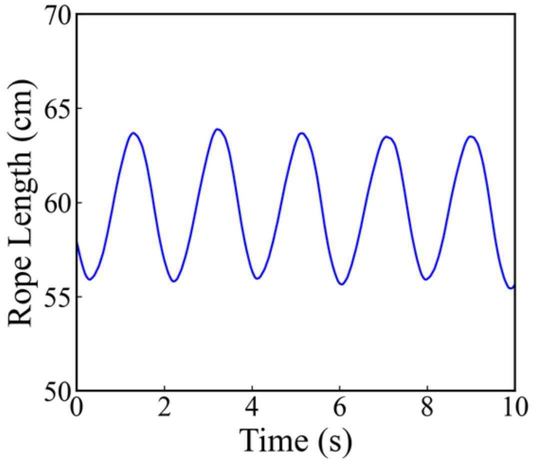

Rope Velocity

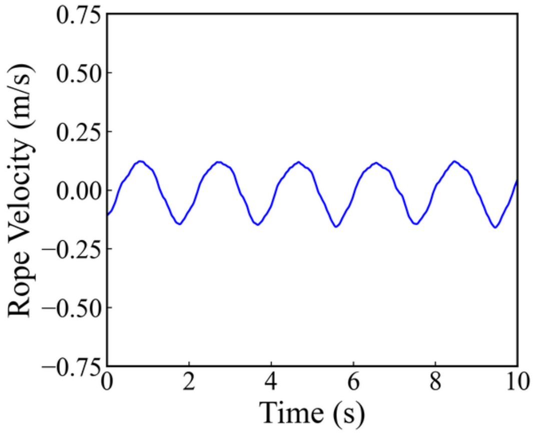

Regular wave: Period1.9s_Height11cm

Trajectory

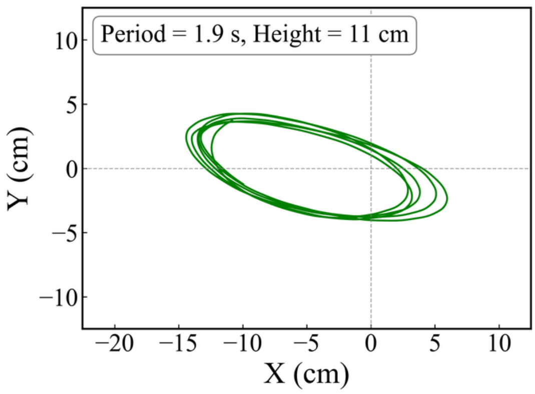

Displacement

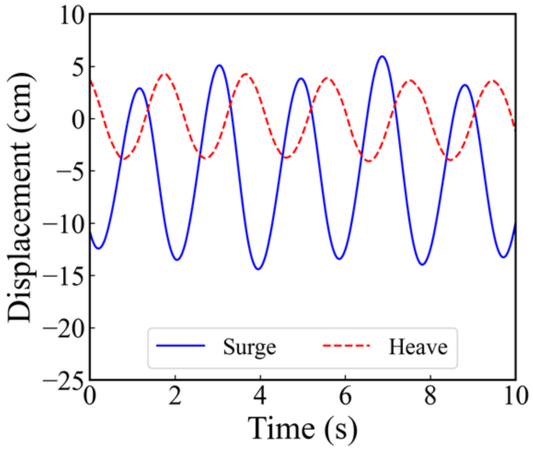

Velocity

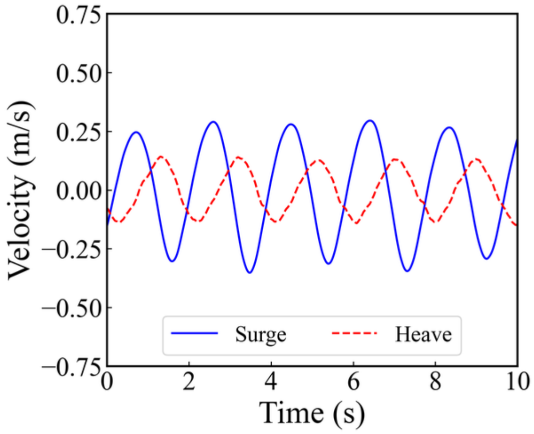

Rope Length

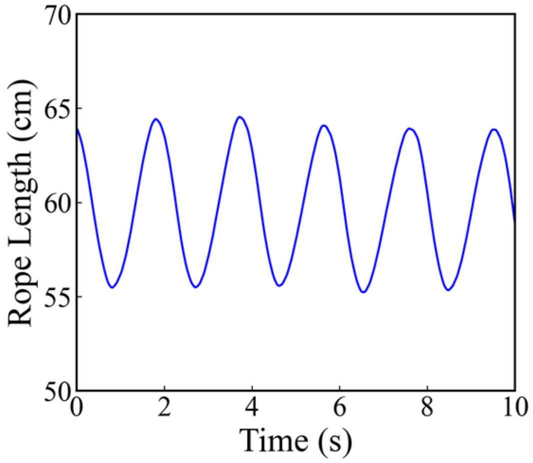

Rope Velocity

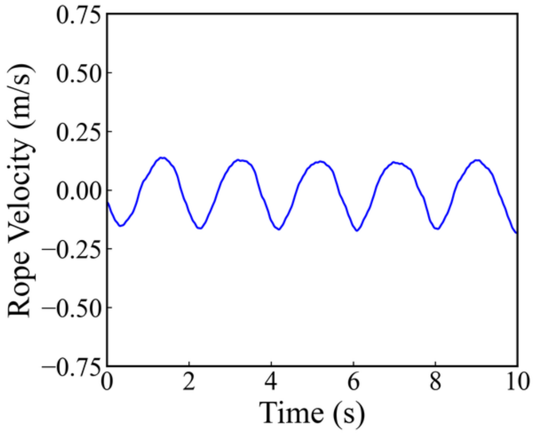

Regular wave: Period1.9s_Height12cm

Trajectory

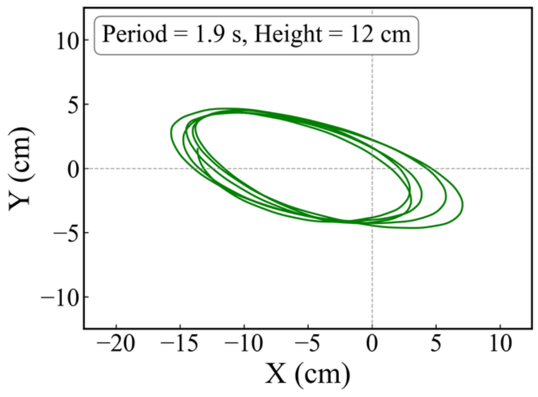

Displacement

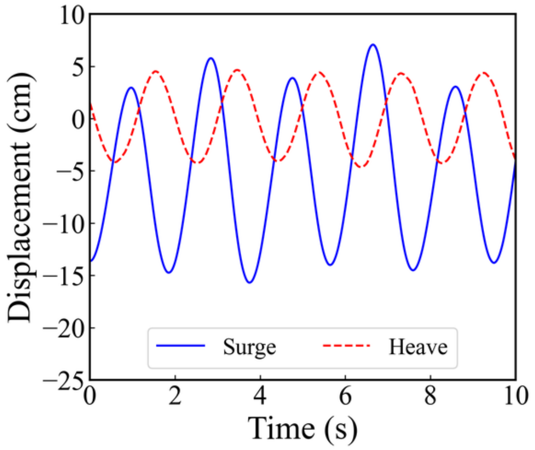

Velocity

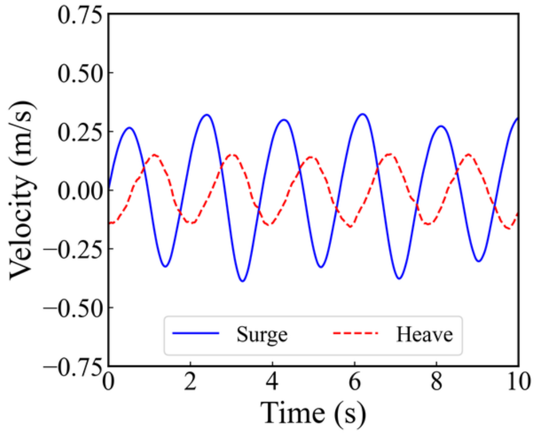

Rope Length

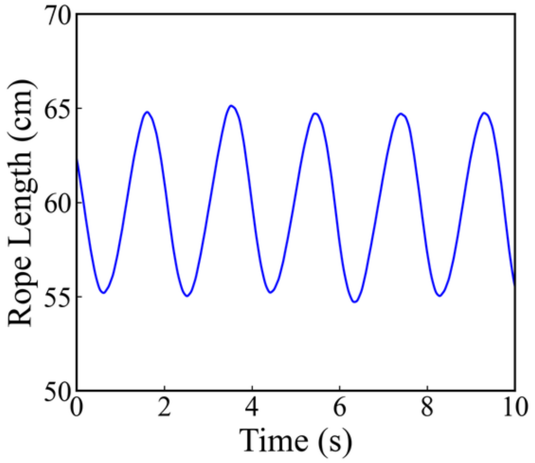

Rope Velocity

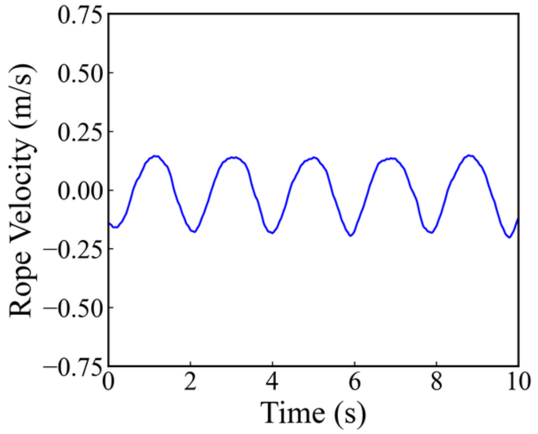

Regular wave: Period1.9s_Height13cm

Trajectory

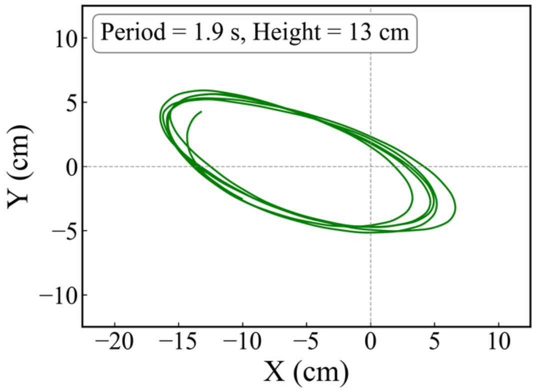

Displacement

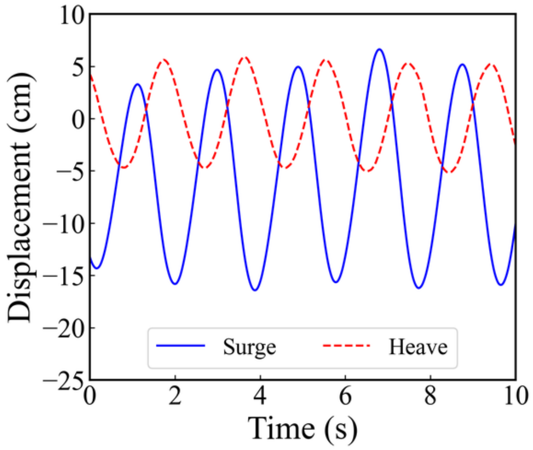

Velocity

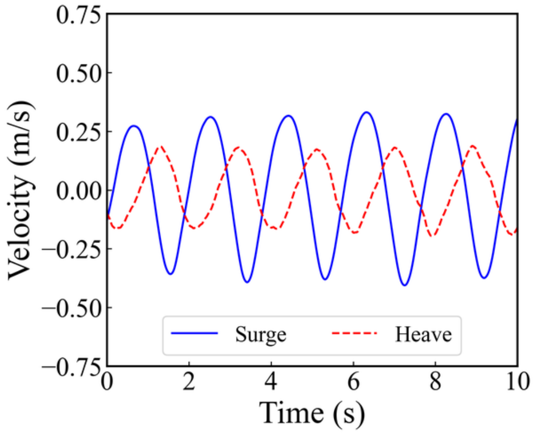

Rope Length

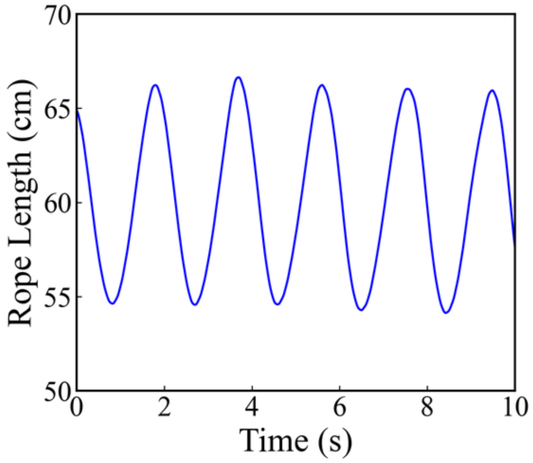

Rope Velocity

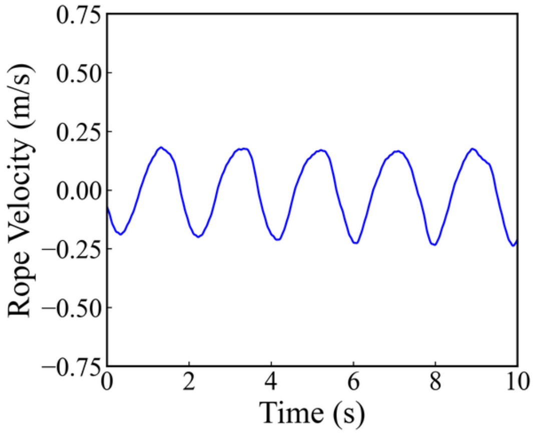

Regular wave: Period1.9s_Height14cm

Trajectory

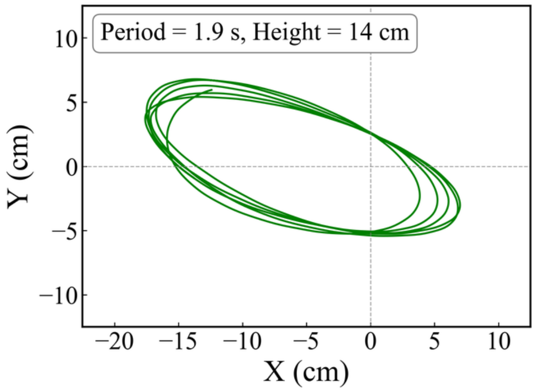

Displacement

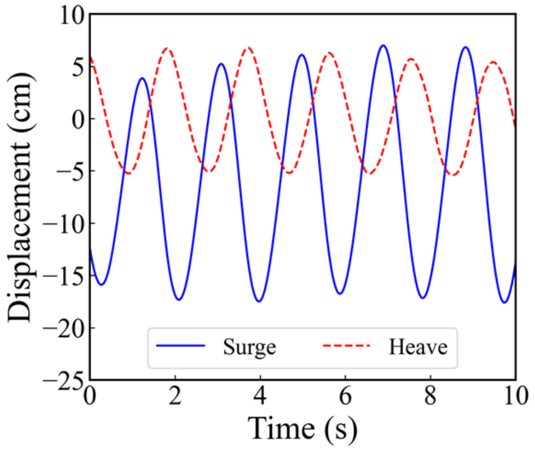

Velocity

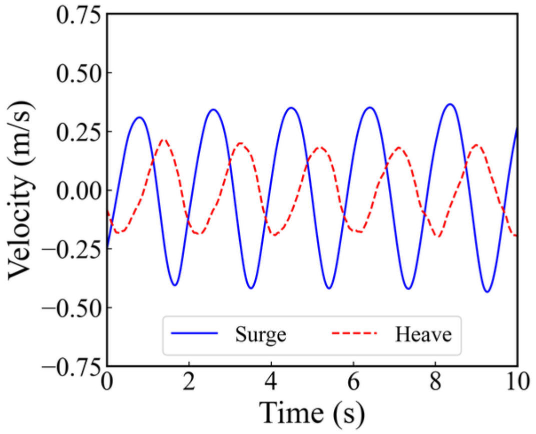

Rope Length

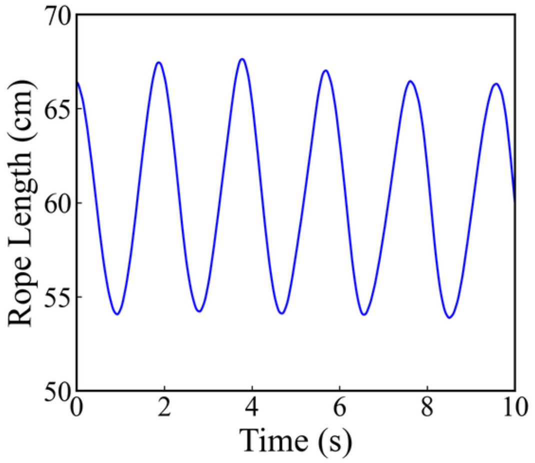

Rope Velocity

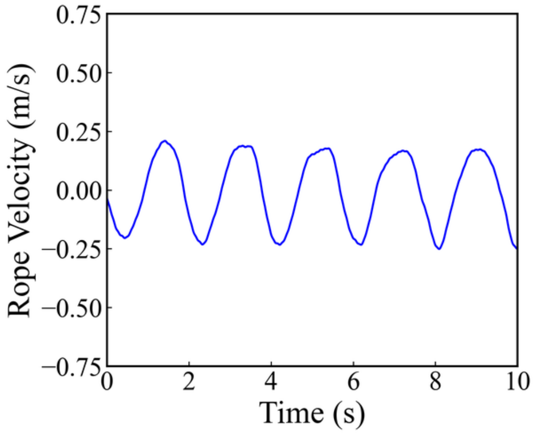

Regular wave: Period1.9s_Height15cm

Trajectory

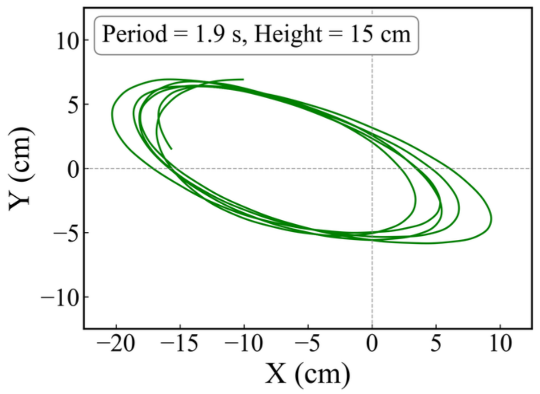

Displacement

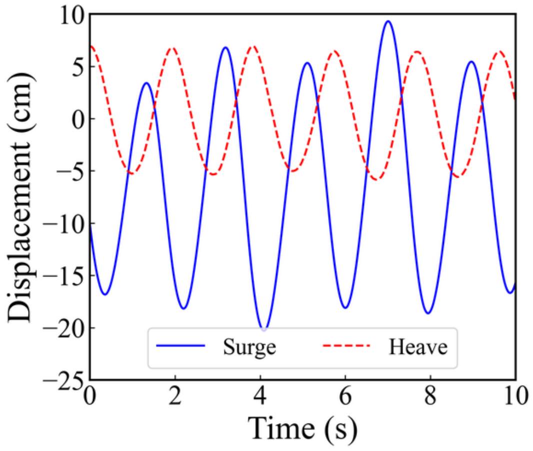

Velocity

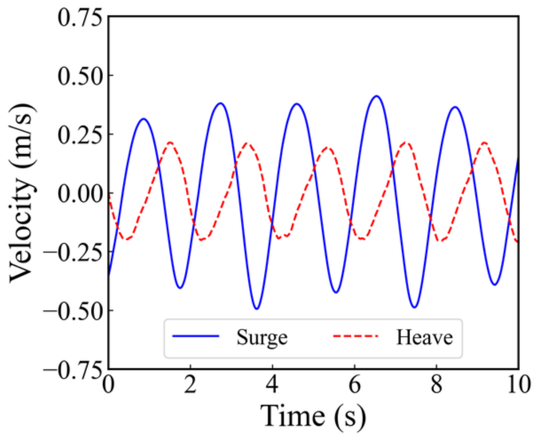

Rope Length

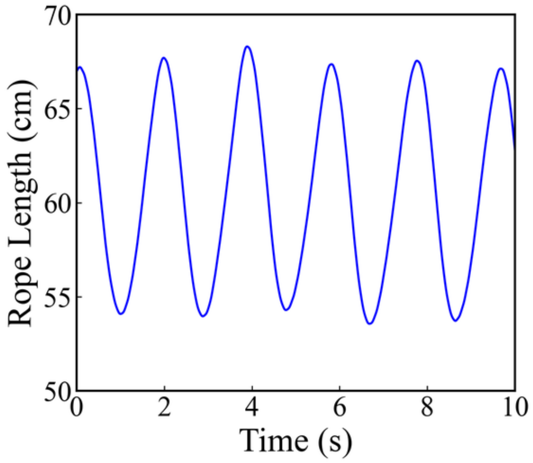

Rope Velocity

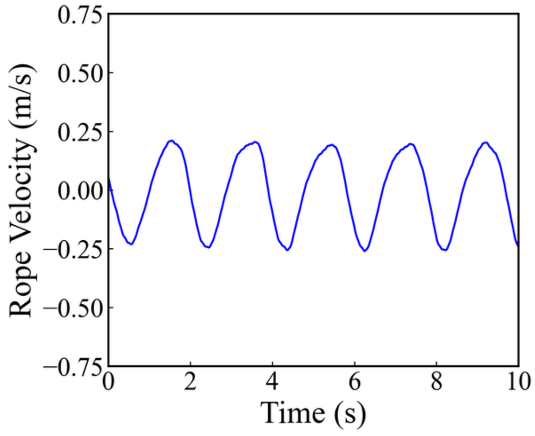

Regular wave: Period2.0s_Height5cm

Trajectory

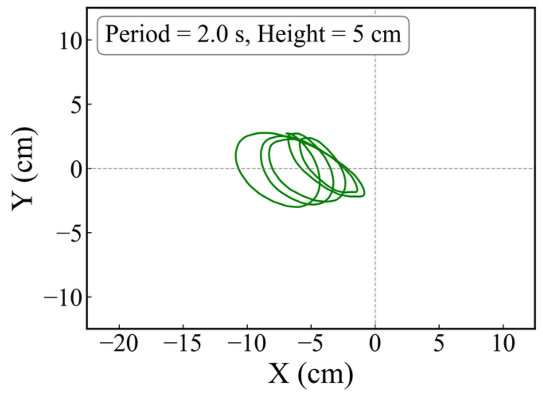

Displacement

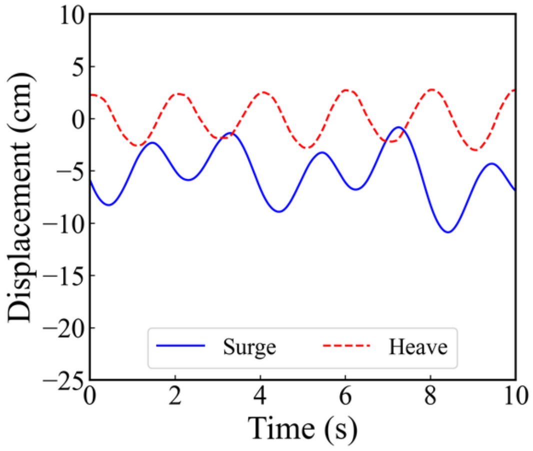

Velocity

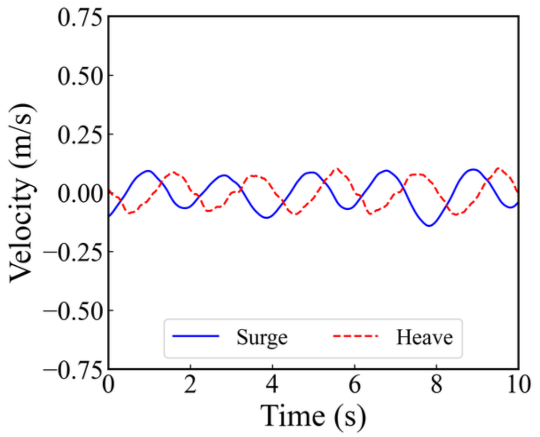

Rope Length

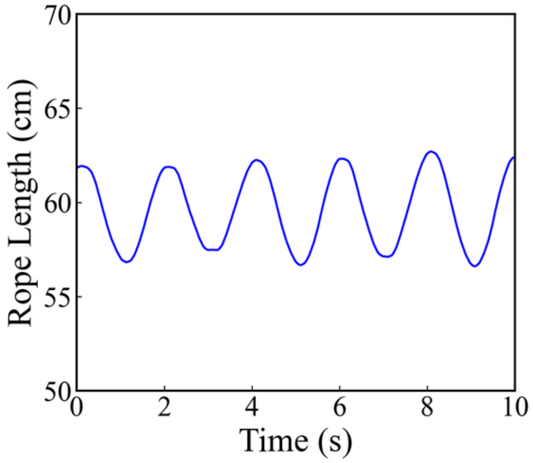

Rope Velocity

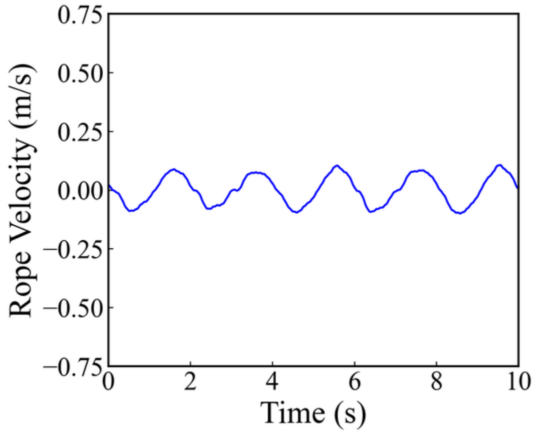

Regular wave: Period2.0s_Height6cm

Trajectory

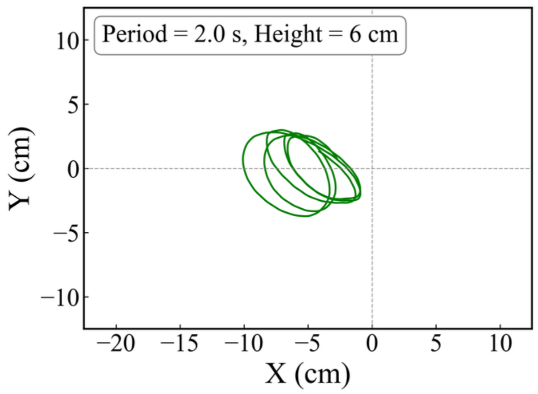

Displacement

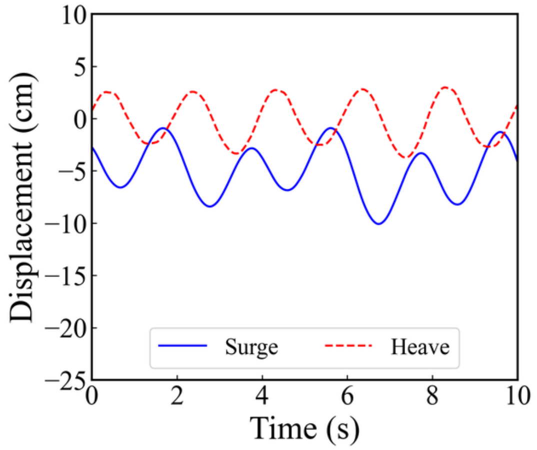

Velocity

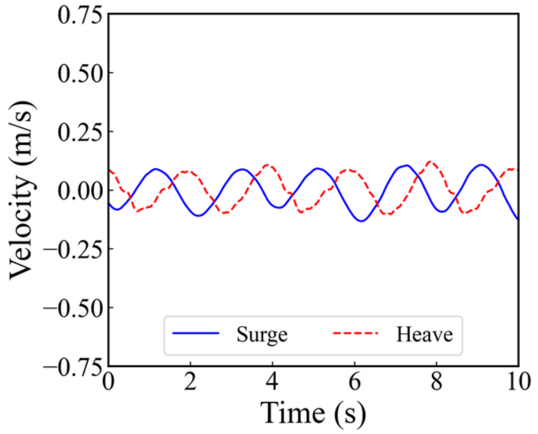

Rope Length

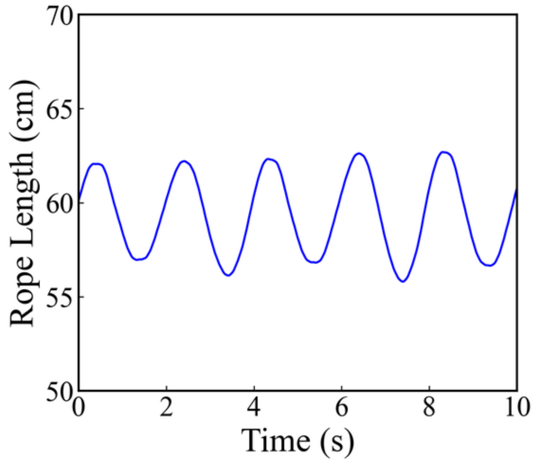

Rope Velocity

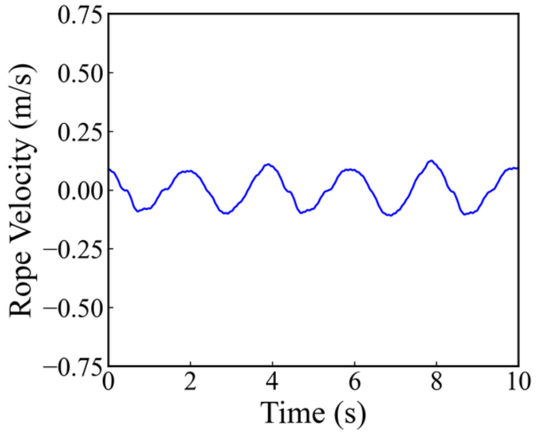

Regular wave: Period2.0s_Height7cm

Trajectory

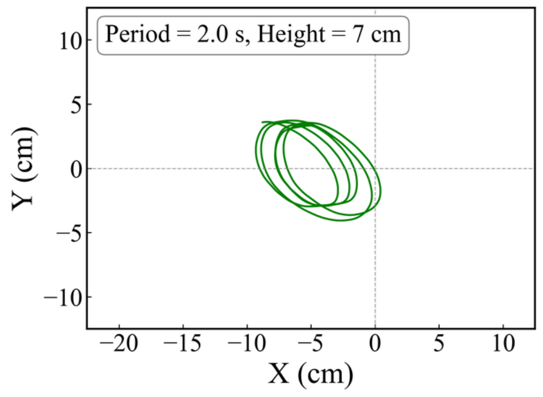

Displacement

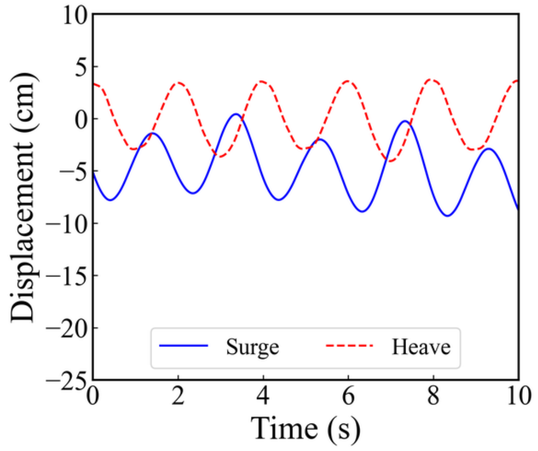

Velocity

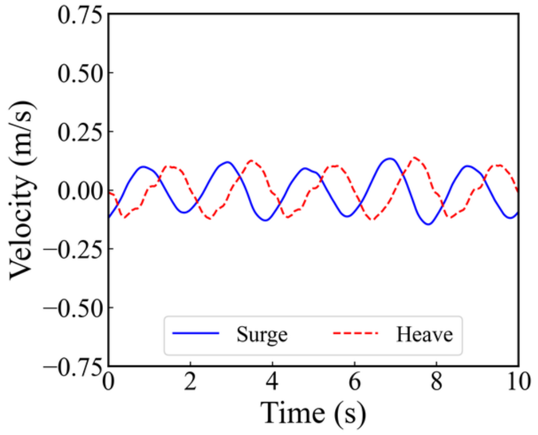

Rope Length

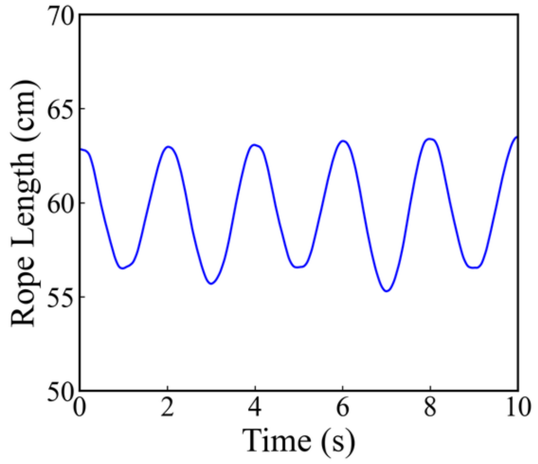

Rope Velocity

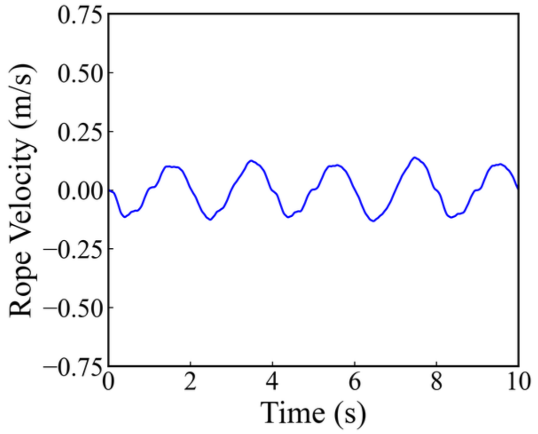

Regular wave: Period2.0s_Height8cm

Trajectory

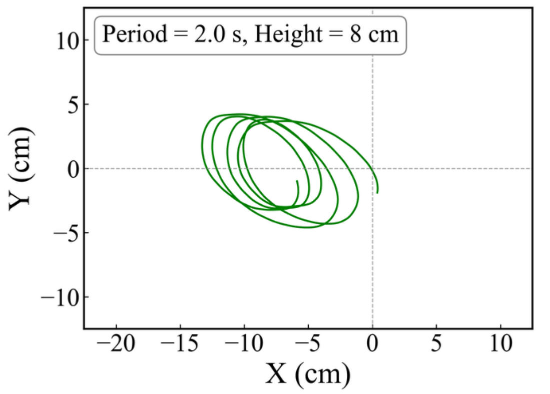

Displacement

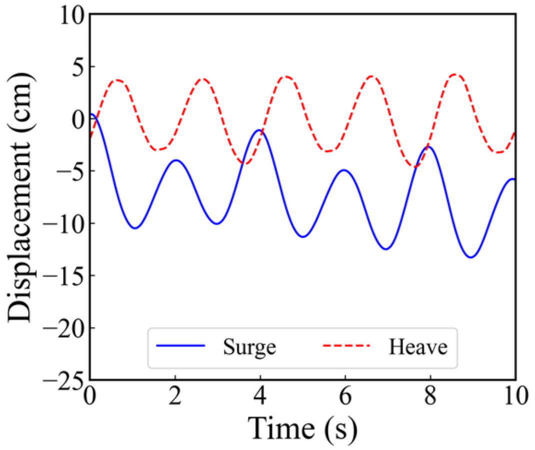

Velocity

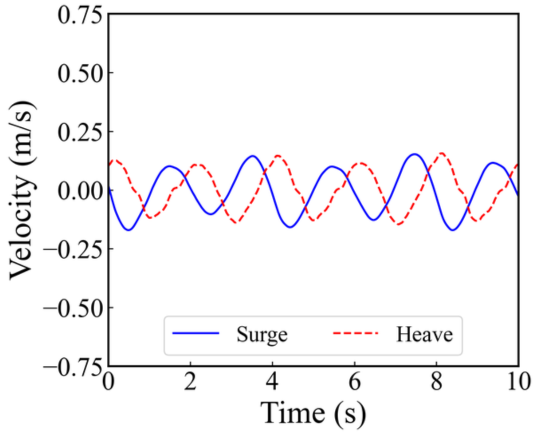

Rope Length

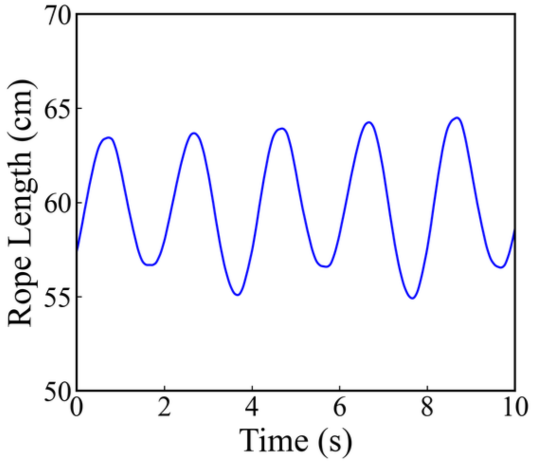

Rope Velocity

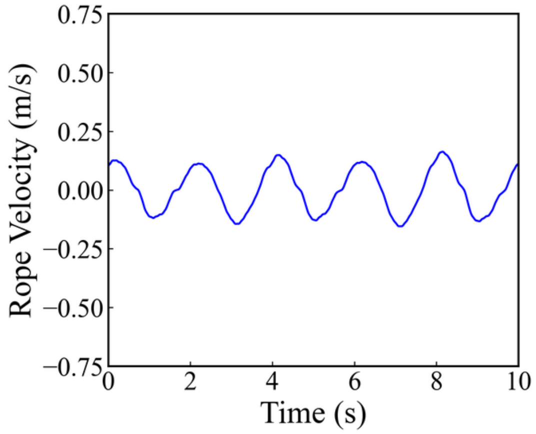

Regular wave: Period2.0s_Height9cm

Trajectory

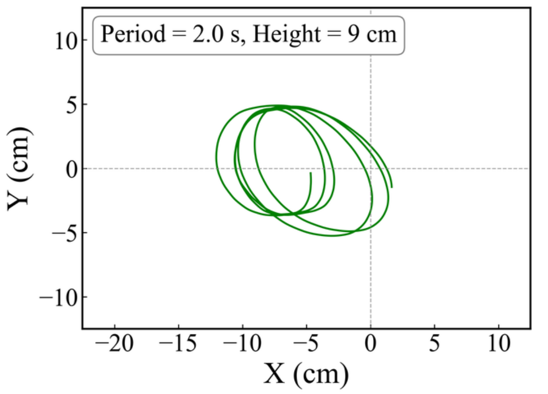

Displacement

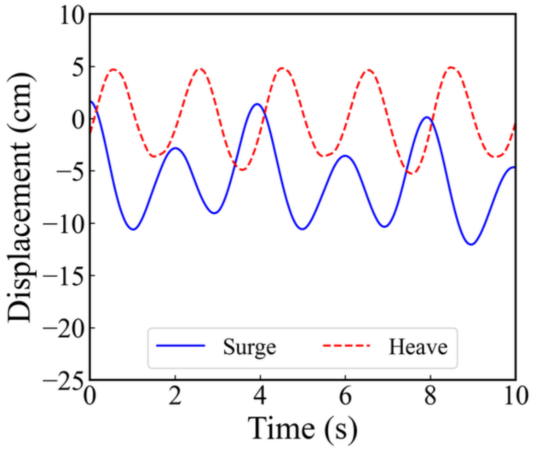

Velocity

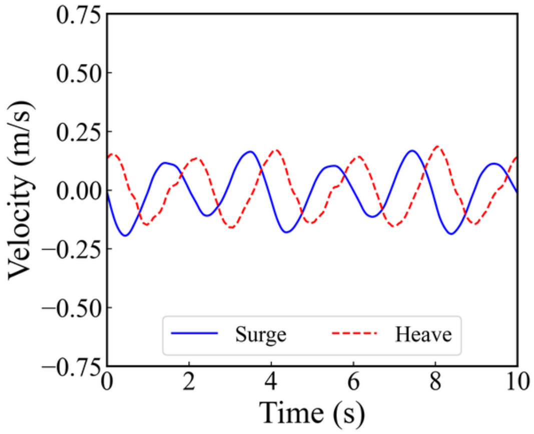

Rope Length

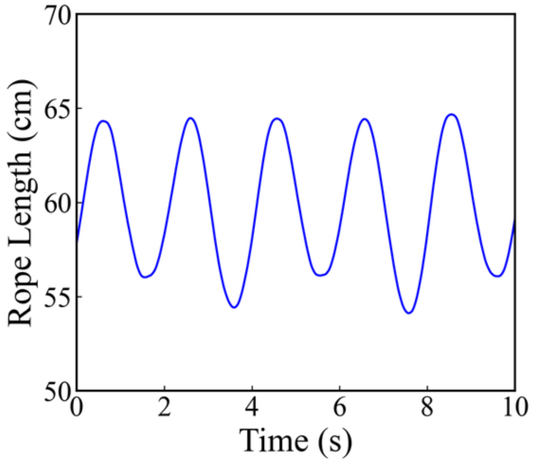

Rope Velocity

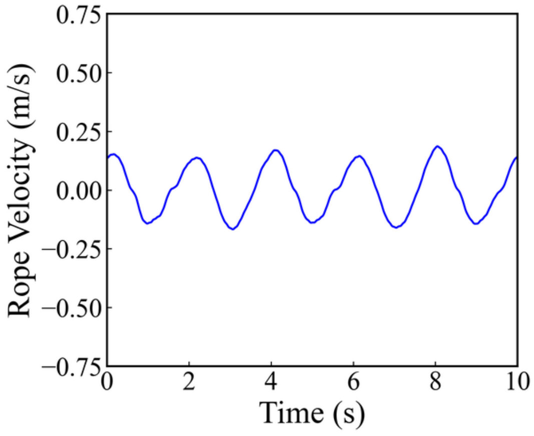

Regular wave: Period2.0s_Height10cm

Trajectory

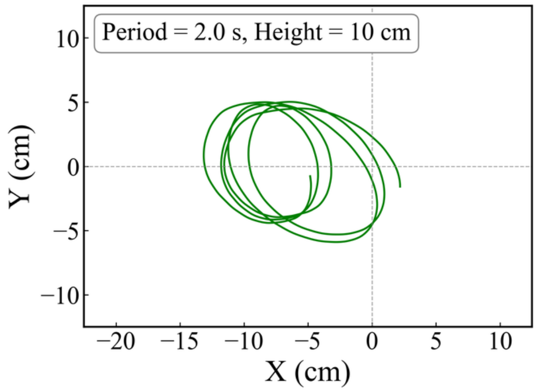

Displacement

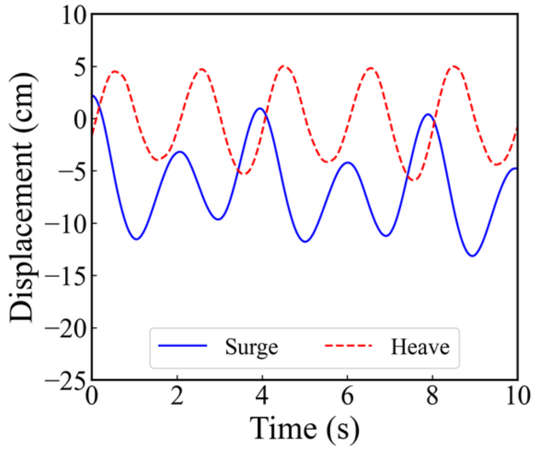

Velocity

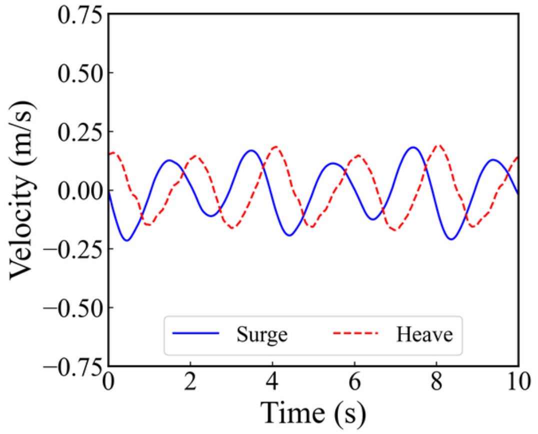

Rope Length

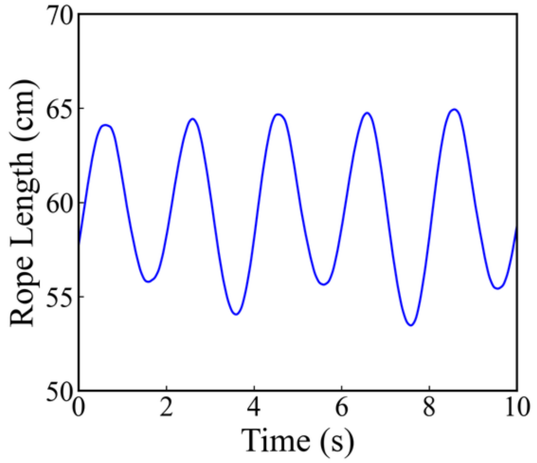

Rope Velocity

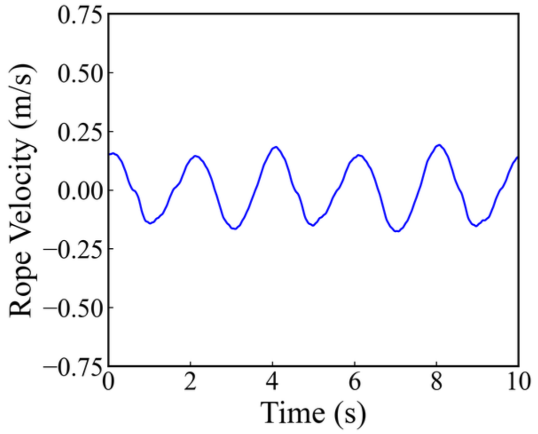

Regular wave: Period2.0s_Height11cm

Trajectory

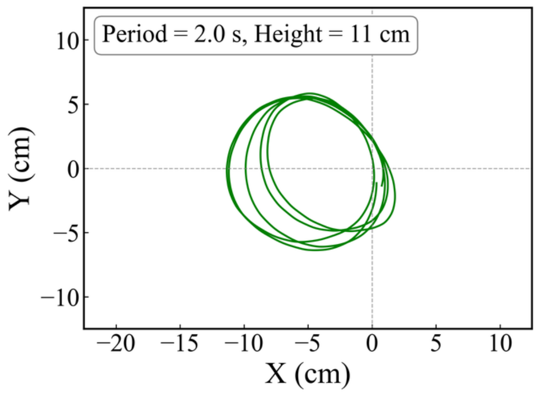

Displacement

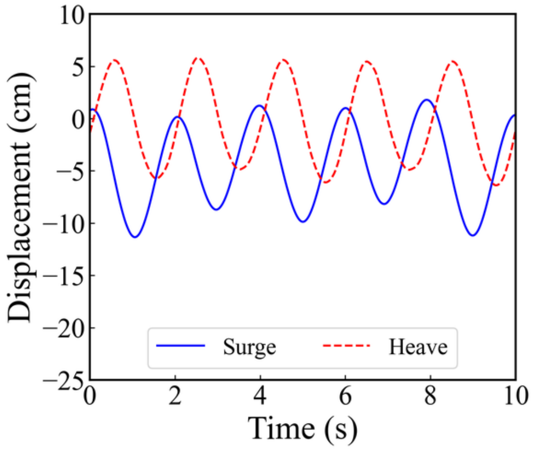

Velocity

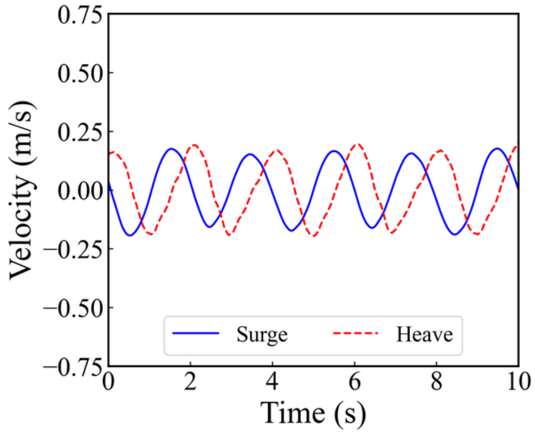

Rope Length

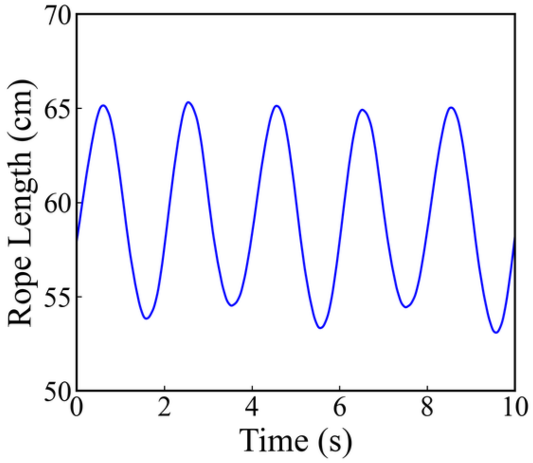

Rope Velocity

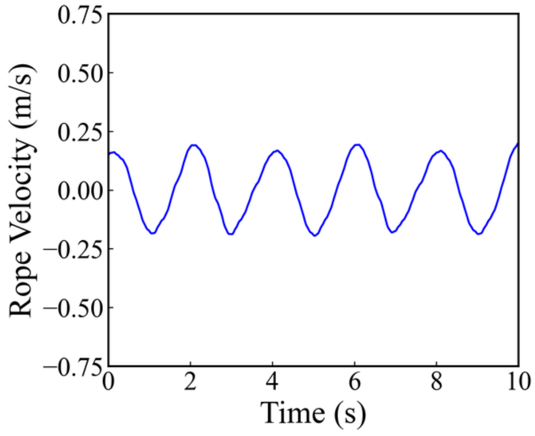

Regular wave: Period2.0s_Height12cm

Trajectory

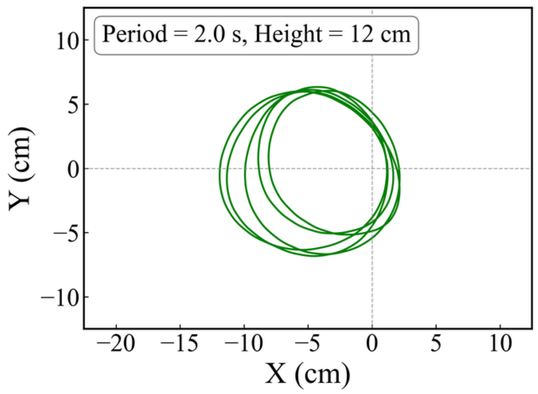

Displacement

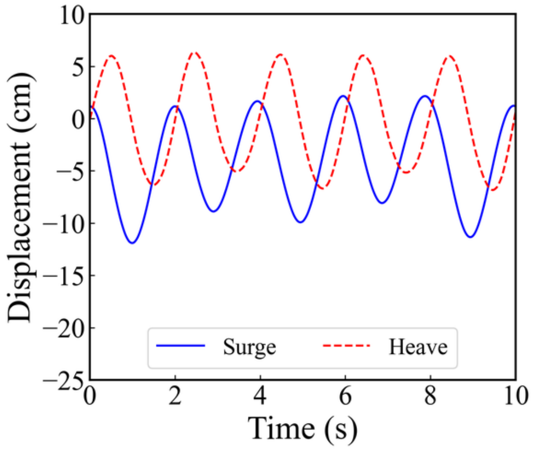

Velocity

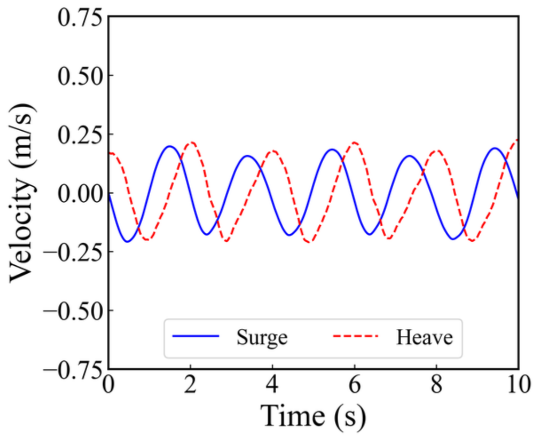

Rope Length

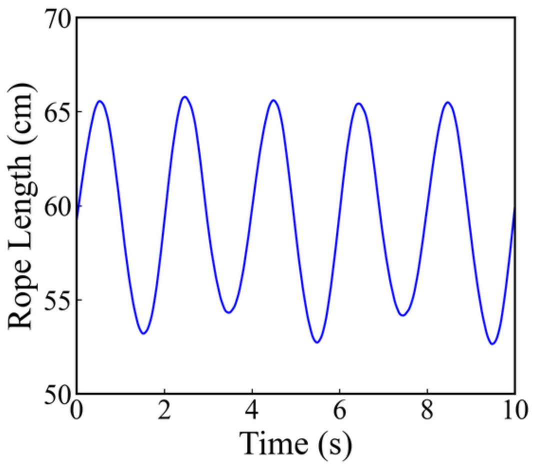

Rope Velocity

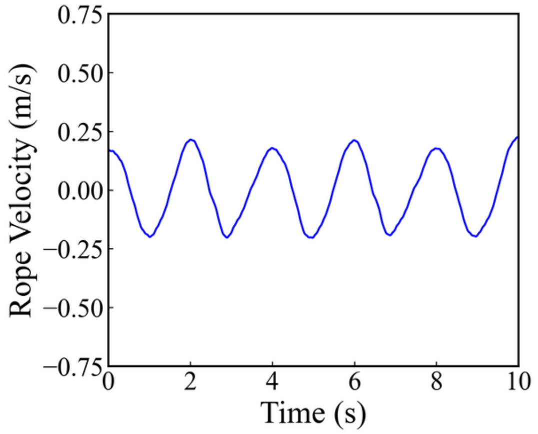

Regular wave: Period2.0s_Height13cm

Trajectory

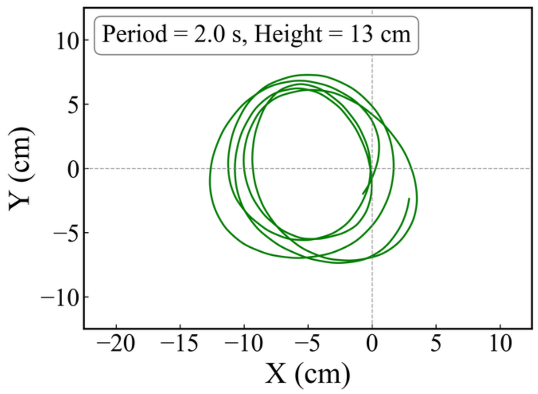

Displacement

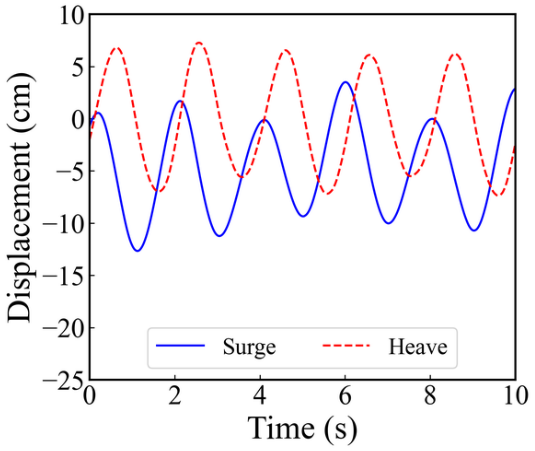

Velocity

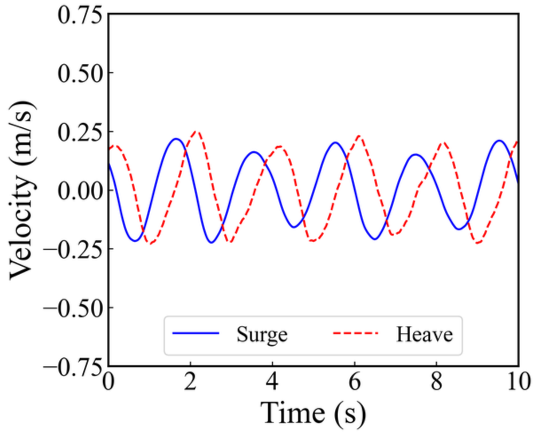

Rope Length

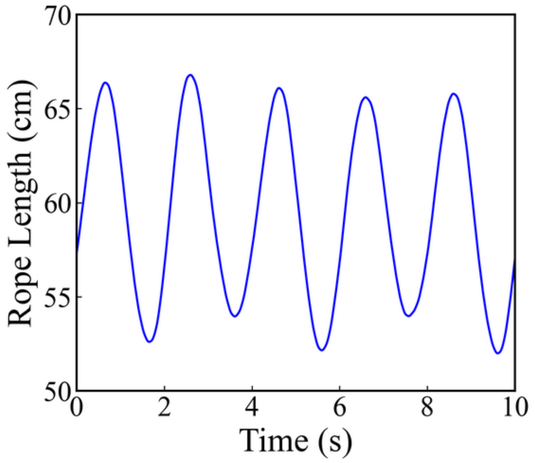

Rope Velocity

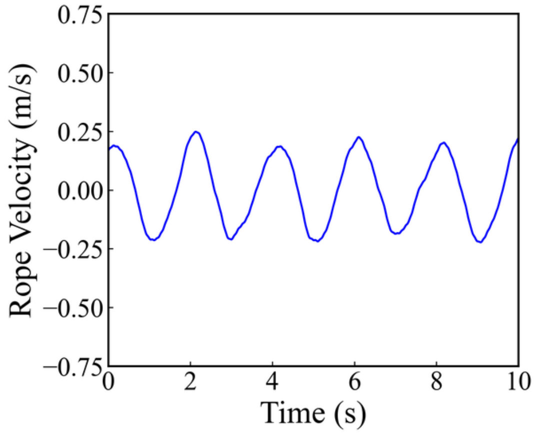

Regular wave: Period2.0s_Height14cm

Trajectory

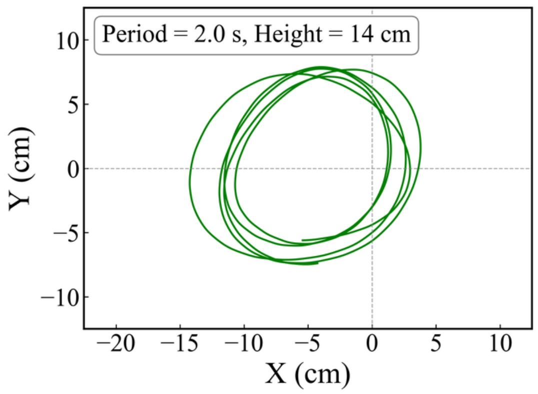

Displacement

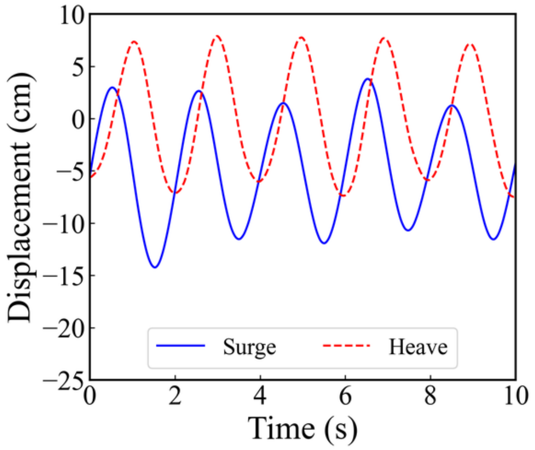

Velocity

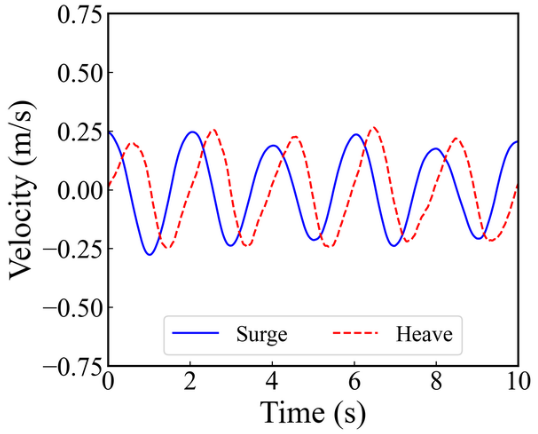

Rope Length

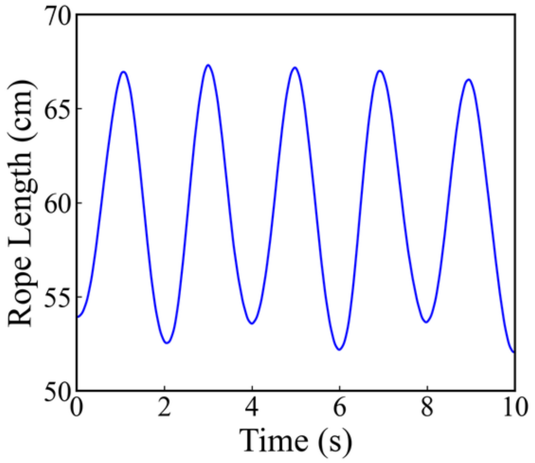

Rope Velocity

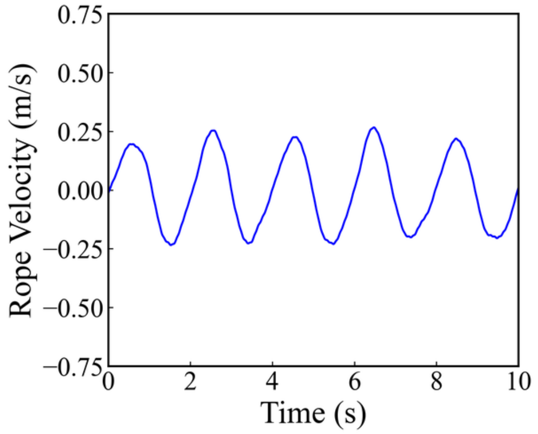

Regular wave: Period2.0s_Height15cm

Trajectory

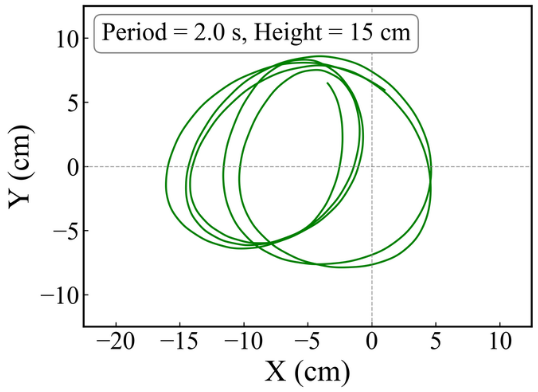

Displacement

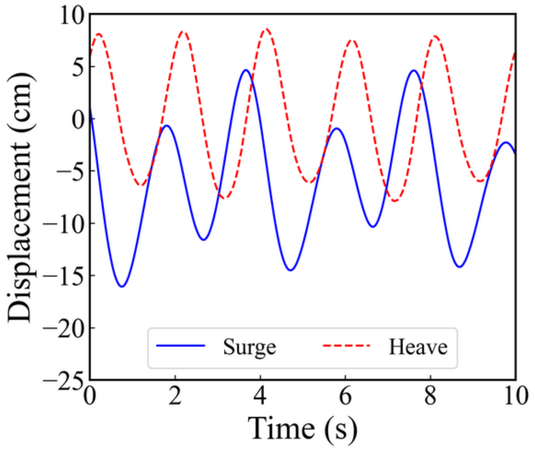

Velocity

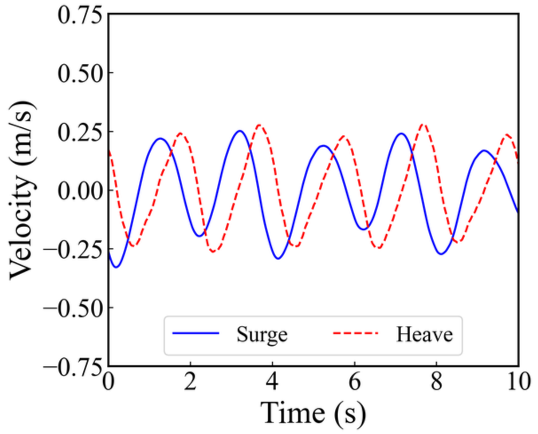

Rope Length

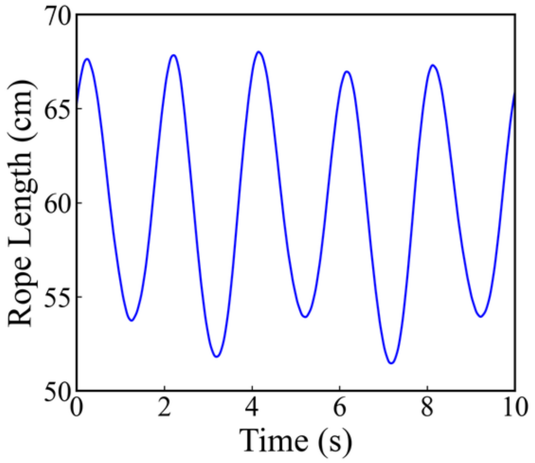

Rope Velocity

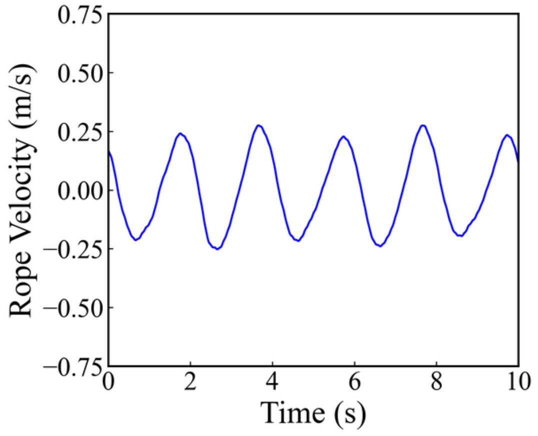

Supplement: Supplementary file 6 — Supplemental Data [file ADVS-13-e16945-s002.zip › Dataset S1.pdf]
