# Supplementary material for: A Pull‐Out Mooring Wave Energy Converter: Design, Analysis, and Application
Source: Adv Sci (Weinh). 2025 Nov 3;13(4):e16945. doi: 10.1002/advs.202516945 (PMC12822436; doi:10.1002/advs.202516945)

Period1.0s_Height5cm

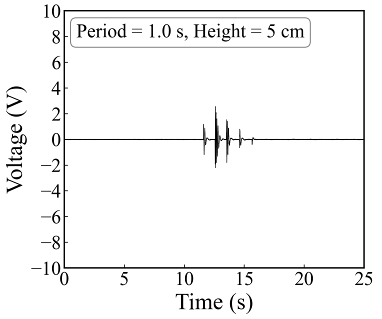

Period1.0s_Height6cm

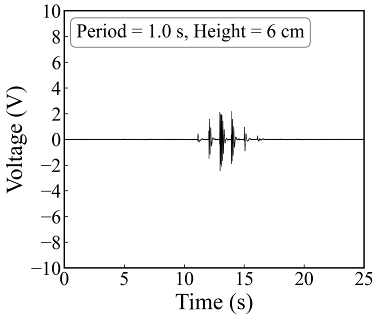

Period1.0s_Height7cm

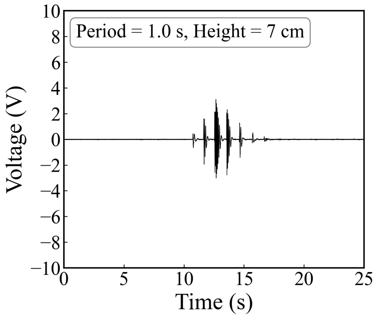

Period1.0s_Height8cm

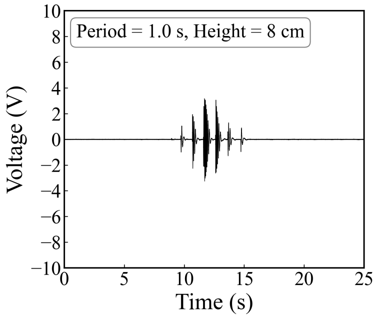

Period1.0s_Height9cm

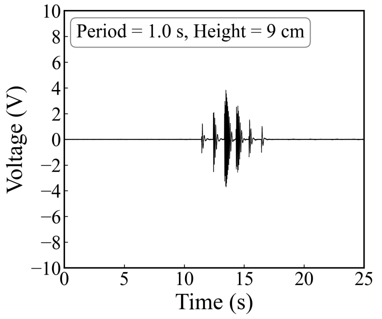

Period1.0s_Height10cm

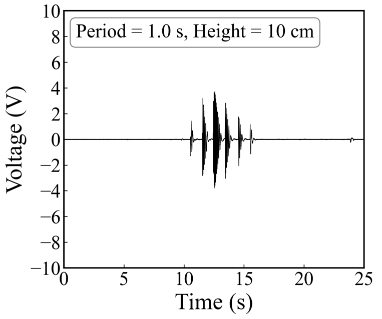

Period1.0s_Height11cm

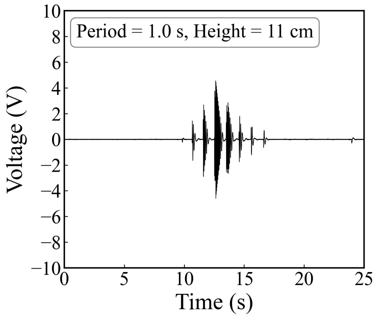

Period1.0s_Height12cm

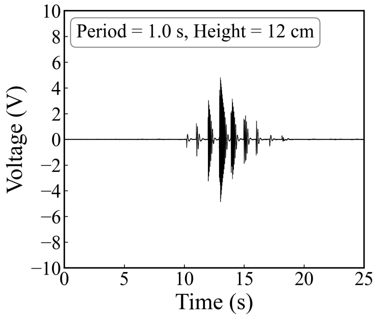

Period1.0s_Height13cm

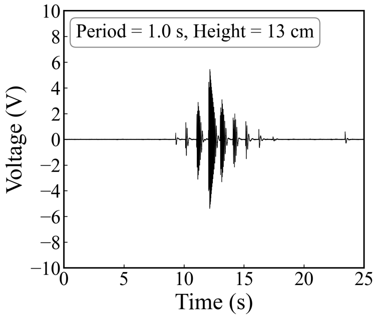

Period1.0s_Height14cm

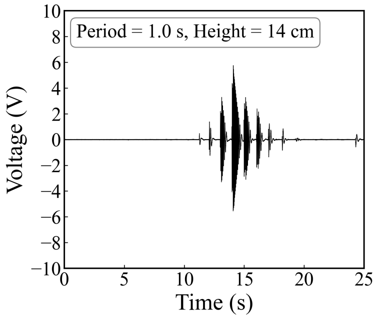

Period1.0s_Height15cm

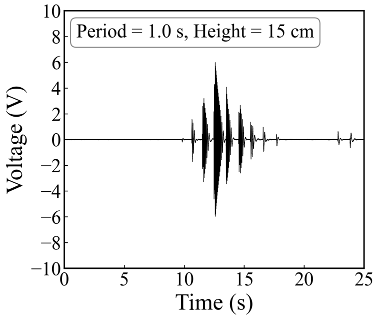

Period1.0s_Height10cm

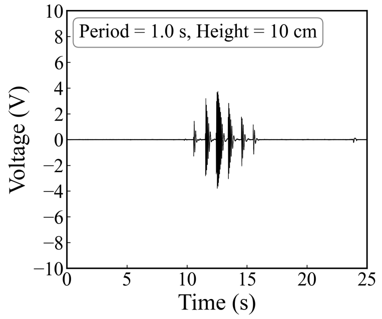

Period1.1s_Height10cm

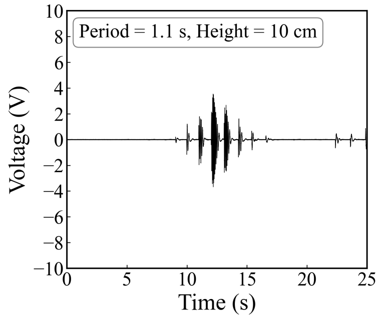

Period1.2s_Height10cm

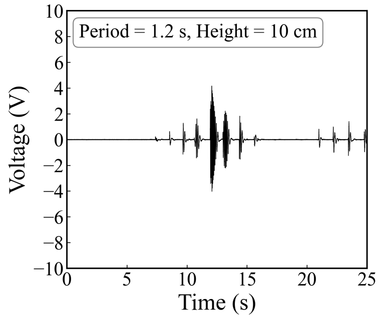

Period1.3s_Height10cm

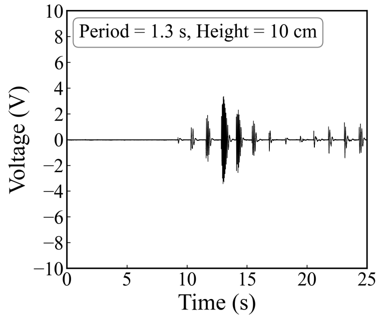

Period1.4s_Height10cm

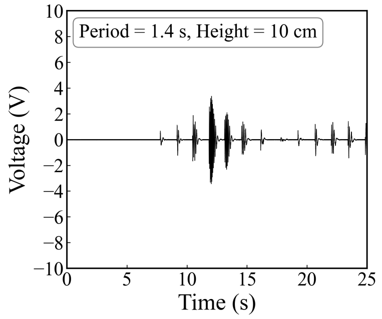

Period1.5s_Height10cm

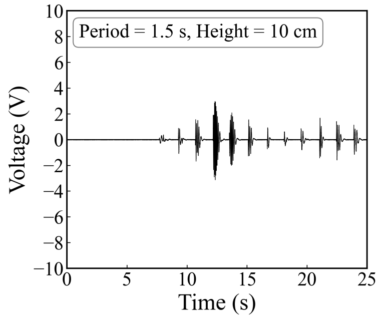

Period1.6s_Height10cm

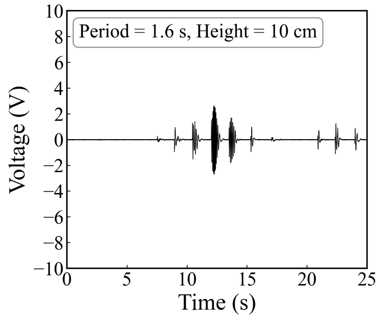

Period1.7s_Height10cm

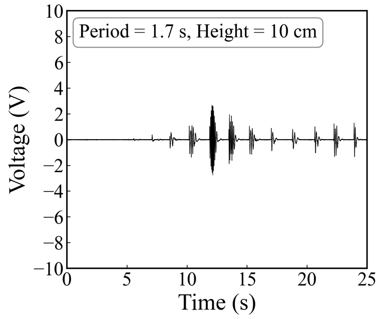

Period1.8s_Height10cm

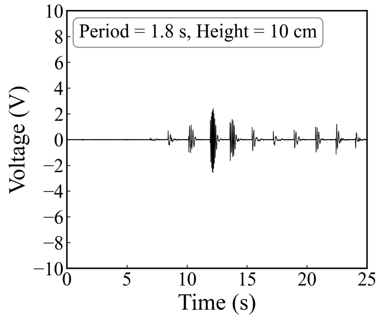

Period1.9s_Height10cm

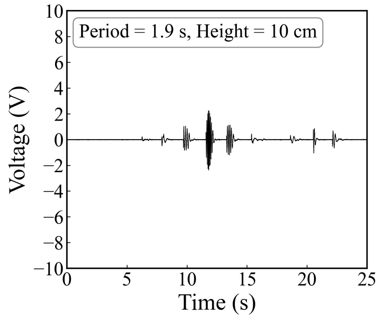

Period2.0s_Height10cm

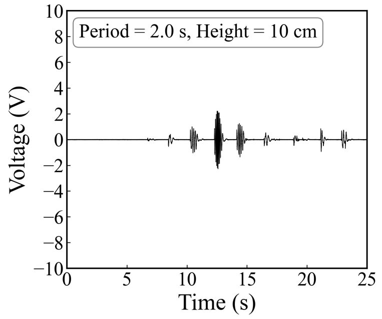

Supplement: Supplementary file 6 — Supplemental Data [file ADVS-13-e16945-s002.zip › Dataset S4.pdf]
